# Polymers Used in Transparent Face Masks—Characterization, Assessment, and Recommendations for Improvements Including Their Sustainability

**DOI:** 10.3390/polym17070937

**Published:** 2025-03-30

**Authors:** Katie E. Miller, Ann-Carolin Jahn, Brian M. Strohm, Shao M. Demyttenaere, Paul J. Nikolai, Byron D. Behm, Mariam S. Paracha, Massoud J. Miri

**Affiliations:** 1 School of Chemistry and Materials Science, Rochester Institute of Technology, Rochester, NY 14623, USA; 2 National Technical Institute for the Deaf, Rochester Institute of Technology, Rochester, NY 14623, USA

**Keywords:** epidemic, polymer applications, transparent face masks, materials characterization, sustainability

## Abstract

By 2050, 700 million people will have hearing loss, requiring rehabilitation services. For about 80% of deaf and hard-hearing individuals, face coverings hinders their ability to lip-read. Also, the normal hearing population experiences issues socializing when wearing face masks. Therefore, there is a need to evaluate and further develop transparent face masks. In this work, the properties of polymers used in ten commercial transparent face masks were determined. The chemical composition of the polymers including nose bridges and ear loops was determined by FTIR spectroscopy. The focus of the characterizations was on the polymers in the transparent portion of each face mask. In half of the masks, the transparent portion contained PET, while in the other masks it consisted of PETG, PC, iPP, PVC, or SR (silicone rubber). Most masks had been coated with anti-fog material, and a few with scratch-resistant compounds, as indicated by XRF/EDX, SEM/EDX, and contact angle measurements. Thermal, molecular weight, and mechanical properties were determined by TGA/DSC, SEC, and tensile tests, respectively. To measure optical properties, UV-Vis reflectance and UV-Vis haze were applied. An assessment of the ten masks and recommendations to develop better transparent face masks were made, including improvement of their sustainability.

## 1. Introduction

Face masks for the protection against respiratory diseases were used prior to the COVID-19 pandemic, particularly in Asia, to prevent airborne infections by humans. Face masks covering the mouth and nose were used during the Manchurian plague of 1910 and the swine flu pandemic of 1918 [1,2]. Face masks were also needed during the influenza pandemic of 2009 [3]. The high casualties since the COVID-19 epidemic have made the use of face masks critically important. During the peak of COVID-19, it was estimated that approximately 129 billion face masks were required [4]. However, even now, a few years after the peak of COVID-19 infections, still a small part of the population, which is more vulnerable, requires face mask protection. Furthermore, we need to be proactive and be prepared for the next respiratory epidemic, which may be transmitted by other organisms.

Most current face masks are made from melt-blown, nonwoven isotactic polypropylene (iPP) and other polymers based on petroleum as starting material [5,6,7]. A few suggestions to use natural fibers for face masks that can biodegrade have been reported [8,9]. Because of the high usage of face masks, their sustainability has become an important issue [10]. Their production requires much energy and contributes significantly to an increase in greenhouse gases, predominantly CO_2_ [11]. Disposable face masks contribute to medical waste that needs to be discarded in a safe and hygienic manner. However, an efficient waste management system for face masks still is lacking [12]. Most used face masks end up in landfills, where they accumulate, since they do not degrade over several centuries. It was estimated in 2020 that about 1.56 billion face masks ended up in the marine environment [13]. Waste from face masks can cause formation of more microplastics into both terrestrial and aquatic environment, including the atmosphere [14,15,16,17,18].

To improve the sustainability of face masks, many efforts have been made to reuse, recycle, and reprocess them. Reuse can involve, for example, washing cycles in a conventional washing machine [19], sterilization in autoclaves [20], or other sanitization methods, such as treatment with ozone or ethanol [5]. Efforts have also been made to recycle waste from face masks [21]. In addition, face masks can be repurposed, such as by being added to concrete for use in pavements and similar applications [22].

It has been forecasted that by 2050 there will be 2.5 billion people living with some kind of hearing loss, of whom 700 million will require rehabilitation services. Wearing masks poses additional challenges for people with certain disabilities. Survey studies conducted by organizations working with deaf and hard-of-hearing (DHH) individuals found that 85% of DHH respondents experienced face coverings hindering their ability to lip-read, while 72% of hard-of-hearing respondents reported that masks made it more difficult to comprehend speech using residual hearing [23].

Transparent (surgical) face masks, also called clear or see-through face masks, were initially designed to help the DHH population in better communicating among themselves or, for example, with educators or medical personnel. These masks are also beneficial for individuals with auditory processing issues and cognitively diverse people. The need for such masks was already realized in pre-COVID-19 times [24]. During the COVID-19 pandemic, the necessity for such masks significantly increased. However, as data showed, wearing solid face masks caused much emotional distress [25]. The lack of facial visibility not only reduced social interaction, but also negatively impacted relationships such as between parents and children [26]. Furthermore, if a larger portion of the population were to wear transparent face masks, it would likely reduce feelings of discrimination among DHH individuals [27]. Therefore, there is much higher demand for transparent masks than just for the DHH population.

To date, only a few articles have been published exclusively on new types of transparent face masks. It is important to distinguish these types of transparent masks from those designed for specific medical purposes, such as those for preventing infections in surgery or for anesthesia [28,29]. In one article, a new type of face mask was discussed with focus on a square-waveform design to increase air flow [30]. The title of the article includes that the face mask would be transparent, and it is mentioned briefly that the transparent portion would be made of clear epoxy. However, the images of the face mask show that the main part of the mask is opaque green, and it is not described how the special air flow design would allow for a clear view of the mouth area, which would be essential for communication. There are a few articles describing transparent air filters; however, they generally do not result in high visibility (max. 70%) and, having been conceptualized before the onset of the COVID-19 pandemic, they do not discuss the use of air filters for transparent face masks [31,32]. In several articles, the effects of transparent face masks influencing speech recognition were discussed [33,34]. The effect of transparent face masks on users’ emotions and other psychological aspects has also been studied [35,36]. However, notably, we could not find an article that discussed in a comprehensive manner the materials used in transparent face masks and their specific properties.

There is clearly a need to evaluate a sufficiently large number of existing types of transparent face masks and to develop improved types of such face masks. In this work, we first focus on determining the materials used in transparent face masks and their properties. We selected ten different commercially available transparent masks and one totally solid mask, the latter serving as a reference. Our measurements included the determination of the areal portion of the clear and solid portion in each mask. The chemical composition of the polymers was studied by FTIR (Fourier Transform Infrared) spectroscopy, because this also allows for distinguishing any coatings on the transparent portion. The material in the breathable portion of the masks represents typical polymer fibers, such as iPP, which have been already studied as described above. We therefore focused on determining the more detailed properties of the transparent portion of each transparent face mask. SEM (Scanning Electron Microscopy)/EDX (Energy-Dispersive X-Ray) and XRD (X-Ray Diffraction)/EDX measurements were conducted for elemental analyses of the coatings on the transparent portions. The wettability was measured by water contact angle goniometry. Specifically, for optical properties, we applied reflectance and haze measurements by UV-Vis spectrometry. Glass transition and melting temperatures were determined by DSC (Differential Scanning Calorimetry). Decomposition temperatures were measured by TGA (Thermal Gravimetric Analysis). Polymer densities were measured by the density gradient column technique, and polymer crystallinity was determined both from the densities, as well as from DSC data. SEC (Size Exclusion Chromatography) was applied to determine molecular weight properties. Subsequently, an assessment of the properties of the face masks is made, by ranking the properties, applying weights by relevance and determining a total score for each type of mask. Finally, recommendations on how to develop more efficient face masks are made. We particularly emphasized aspects regarding the sustainability of the face masks.

## 2. Materials and Methods

### 2.1. Materials

All investigated face masks were commercially available and easily purchasable, e.g., most through Amazon.com, Inc. We attempted to purchase additional face masks; however, a few companies were not willing to provide samples. We only investigated masks that were of adult size.

### 2.2. Basic Physical Properties

The transparent, structural, breathable, and filter portions of each mask were measured. The mask was flattened, and the contours of each portion were traced on a piece of uniform filter paper. The cutouts of each portion were weighed and compared to the total weight of the paper. The rigid JEM mask was unable to be flattened, so masking tape was applied directly to the mask and peeled off to create a stencil; this stencil was used to trace onto the paper, and the same method of weighing was followed.

### 2.3. Fourier Transform Infrared (FTIR) Spectroscopy

FTIR measurements were recorded using a Shimadzu IRPresige21 instrument (PIKE Technologies, Madison, WI, USA) with an accessory for ATR measurements by PIKE Technologies GladiATR instrument. The software Shimadzu IRsolution 1.60 was applied. Scans were taken of the side of the transparent portion that was closer to the user (“inside”), as well as the side that pointed away from the user (“outside”). Spectra were taken also from the structural, breathable, and filter portions and ear loops of each mask.

### 2.4. Water Contact Angle Goniometry

The wettability of the interior and exterior of the transparent portion of each mask was measured on a Rame-Hart Advanced Goniometer (Rame-Hart Instrument Co., Succasunna, NJ, USA). The software DROPimage 2.8.02 was applied. A 2 μL drop size was used. Typically, measurements were run as five replicates, unless the deviation was significant, and more reproducibility runs were necessary. For several samples, the polymer sample had to be flattened using adhesive tape on the edges to avoid changes in contact angle due to the convex or concave curvature of the samples.

### 2.5. Density Gradient Column

A density gradient was formed by mixing carbon tetrachloride and ethanol, using close to 30 mixtures with different compositions of the two solvents. A 500 mL graduated cylinder was placed in a large water bath at 25 °C. Samples were dropped into the cylinder, and the mL mark, at which they remained floating, was recorded. The readings were compared against a calibration curve based on densities of known polymer standards to determine density.

### 2.6. Differential Scanning Calorimetry (DSC)

DSC was run on a TA Instruments Discovery DSC 2500 with a TA Instruments RCS 90 cooling unit (TA Instruments, New Castle, DE, USA) using TRIOS software. Nitrogen was used for gas flow, and the flow rate was set to 40 mL/min. Samples were prepared using 10 mg of material in aluminum sample pans. The temperature ramp rate was 10 °C/min, and the bounds were set to 20 °C and 300 °C for most masks. RAN was run between 20 °C and 180 °C to avoid degradation and OPT was run between −50 °C and 250 °C to catch its lower anticipated glass transition. Each mask was run for three heating and cooling cycles with 5 min isothermals in between each heating or cooling step.

### 2.7. Size Exclusion Chromatography (SEC)

For the polymers soluble in chloroform (Jelly M1, Jelli Tech International, Dover, Delaware, Rafi Nova, Needham, Massachusetts), SEC measurements were recorded on a Shimadzu LC02030 plus instrument; an RID (Shimadzu RID-20A, Shimadzu, Kyoto, Japan) was used. A 10 mg quantity of each polymer sample was dissolved in HPLC-grade chloroform and filtered through syringe filters into GC vials. The flow rate was 1 mL per minute.

For the PET-type samples, SEC analysis was carried out in hexafluoroisopropanol (HFIP) with 0.05 M potassium trifluoroacetate (KTFAc) at 1 mL/min using two PSS (Agilent, Santa Clara, CA, USA) PFG linear M columns with a particle size of 7 μm and a matching guard column using a PSS SECcurity2 system, consisting of an isocratic pump, autosampler, column oven (30 °C), and refractive index detector (RID) (30 °C). Sample concentration was 3 g/L, and the injected sample amount was 50 μL. In order to obtain the molar mass distributions, a calibration curve with polymethyl methacrylate standards from PSS was created, using PSS WinGPC Unichrom 8.33 for data processing.

OPT was run in 1,2,4-trichlorobenzene at 160 °C at 1 mL/min on PSS—Polefin Q: 10 μm G, 3 × Linear XL columns. Sample concentration was 1 g/L, and the injected sample amount was 100 μL. Polystyrene from PSS were used as calibration standards.

SEU cannot be dissolved in a solvent to run SEC, since it is a crosslinked polymer.

### 2.8. Thermal Gravimetric Analysis (TGA)

TGA was run on a TA Instruments TGA Q500. Nitrogen was flown through the oven at a flow rate of 20 mL/min. Samples were prepared using 10 mg of the sample in a platinum pan. The sample was heated in the oven from 25 to 700°C at a rate of 10 °C/min. The degradation temperatures were determined from the derivative of the sample weight using TRIOS software.

### 2.9. UV-Vis Spectrometry

The relative reflectance and transmittance of the transparent portion of each mask was recorded on a Shimadzu UV-2600 instrument. The software program “Lab Solutions UV-Vis” was applied. An integrating sphere was used. The reflectance was measured between 190 and 800 nm and the haze was recorded between 350 and 700 nm. The film specimens were rectangular, at least 2.5 cm long and 1.2 cm wide. For the reflectance and haze measurements different configurations were applied according to the manufacturer’s instructions.

### 2.10. Stress Strain Measurements

Tensile tests were conducted with an Instron 34TM-50 (with a 1 kN load cell and 1 kN side action grips) using Bluehill Universal software. ASTM D638-14 (Standard Test Method for Tensile Properties of Plastics) was applied (Instron, Norwood, MA, USA). Most samples were prepared as rectangles with a width of 10 mm and a length between grips of 30 mm. The rectangles typically were cut in vertical direction of each face mask (for Safe ‘N’Clear there was insufficient length vertically and specimen had to be cut horizontally). Jelly M1, due to its greater thickness, was run in a dumbbell shape (ASTM D638, type V) with a width of 3.18 mm and a length of 9.53 mm cut from the thin middle section. Typically, 5 specimens of each polymer were tested (for Jelli M1 only samples for 4 dumbbells were available). The crosshead speed was 50 mm/min. The strain rate was 50 mm/min (except for BEclear and Rafi Nova, where the strain rate was reduced to 15 mm/min to avoid premature tearing).

### 2.11. X-Ray Fluorescence/Energy-Dispersive X-Ray (XRF/EDX) Spectroscopy

The XRF/EDX data were obtained with a Shimadzu EDX 8100 instrument. PCEDX 2.07 Navi software was used to process the data. The transparent polymer films were cut to pieces that were 1.5 cm × 1.5 cm. The measurements were performed under vacuum.

### 2.12. Scanning Electron Microscopy (SEM) and Energy-Dispersive X-Ray (SEM/EDX) Spectroscopy

A Tescan Vega 3 instrument (Tescan Orsay Holding, a.s., Brno, Czech Republic) was applied to take images of a few solid portions of the masks. The samples were directly placed on a stub with double-sided carbon tape and coated with Pd/Au; 10 kV was applied.

For EDX measurements of elements, a Bruker EDX 630 M (Bruker, Billerica, MA, USA) was used as accessory. The samples were coated with carbon for conductivity because only elements of lower atomic mass were of interest; 20 kV was applied.

## 3. Results and Discussion

**Figure 1:**
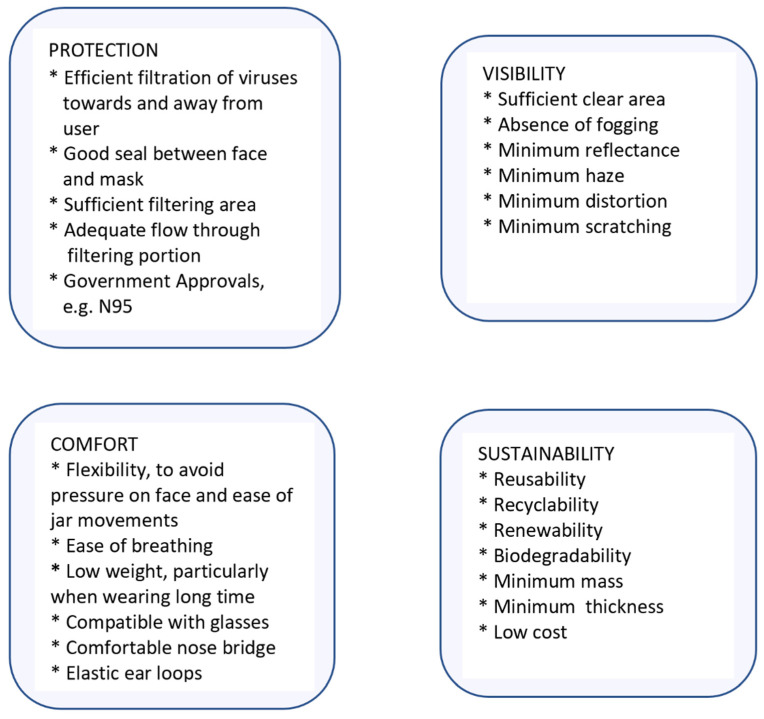
Relevant criteria for the effectiveness of transparent face masks.

In addition to the requirements of efficient face masks, transparent face masks must meet further criteria for their effectiveness. In the case of transparent face masks, visibility in particular is highly relevant. The criteria are shown in Figure 1.

In Table 1, eleven characterized face masks—of which ten were transparent (Entries 1 to 10) and one was solid and served as a reference (Entry 11)—are listed, including the websites for each mask. In Table 2, general and relevant information from the face mask manufacturers is given. In Table 3, the standards that are applied to some of the face masks are explained.

As shown in Table 2, only one of the transparent face masks can be considered fully N95-approved, OPT. SEM has a filter, which is N95-approved. Many of the masks are latex-free regarding the material used for the ear loops. Several masks also have anti-fog coatings. For most masks, a complete and specific description of the applied materials was not provided by the manufacturers.

Table 3 shows the differences between the three standards applied for face masks mentioned in Table 2.

### 3.1. Determination of Basic Physical Properties, Including Mass and Sizes of Different Portions of Masks

**Figure 2:**
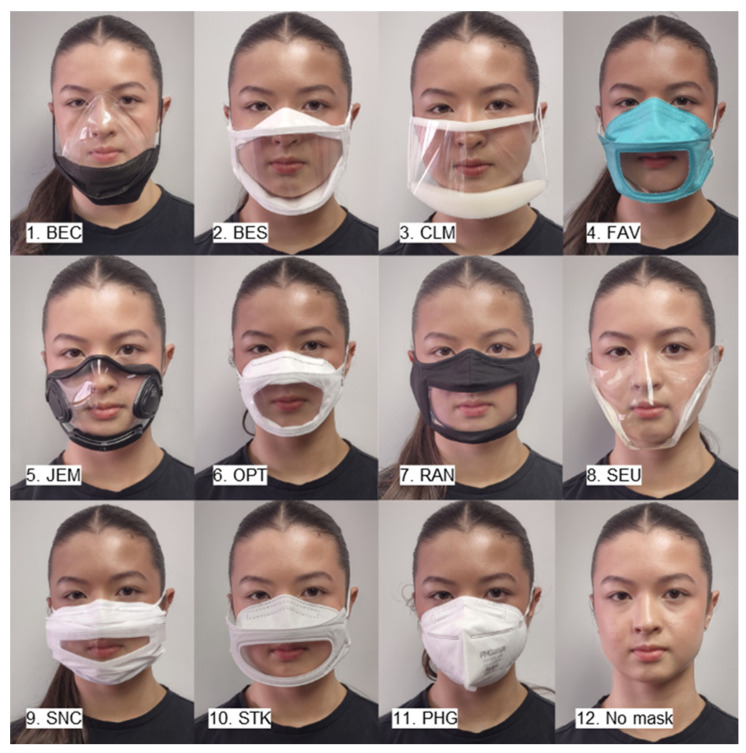
Photos of 11 face masks and one without mask (showing co-author Katie Miller, photographed by co-author Brian Strohm).

In Figure 2, photos of the eleven investigated masks are shown. The views from the side or profile are given in the Supplementary Materials.

From the photos in Figure 2, some distinctions on the face masks already become apparent. The visible area of the mouth and its vicinity, which is critical for lip reading, is seen best with CLM and worst with SNC. Visual distortions are observed most for BEC and SEU, and the least for OPT and RAN. Reflections are observed the most for BEC and SEU and the least for OPT and RAN. Further distinctions are described in the Supplementary Materials.

**Figure 3:**
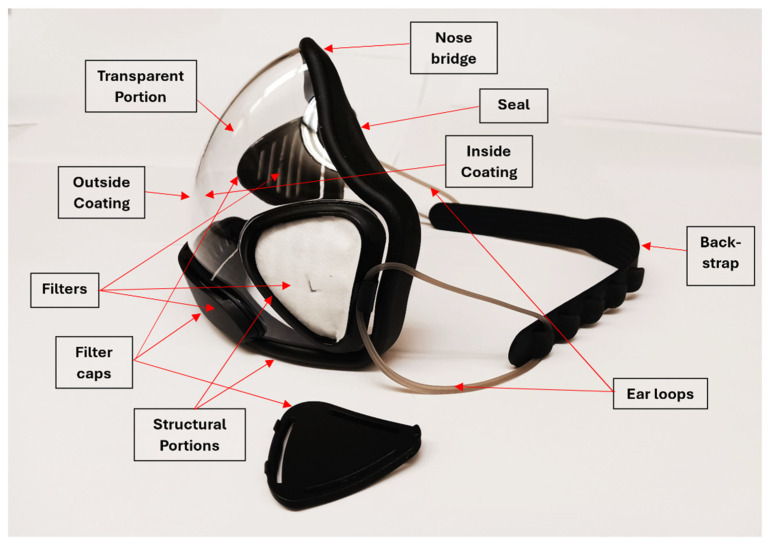
Components of a more complex transparent face mask using the example of JEM.

In Figure 3, the different parts of a more complex transparent face mask (JEM) are identified. In most masks, several of the shown parts, such as a filter or backstrap, are not included. There is also no separate nose bridge in JEM, unlike in many other masks (in JEM, the nose bridge is part of the rubber seal).

Among the components of the face masks, we mainly investigated the transparent portions, and any coatings on these. We also characterized any major structural portions, any sealing portion, any filters, and nose bridges and ear loops, the latter affecting comfort significantly. We verified that the breathable portions were mostly made of melt-blown, nonwoven polypropylene and did not study this further, since this has been discussed thoroughly in the literature [5,6,7,19,41]. The polypropylene typically contains several layers and may be charged to produce an electrostatic layer. We also did not investigate stitches (most typically made of cotton) or staples (typically made of steel).

**Figure 4:**
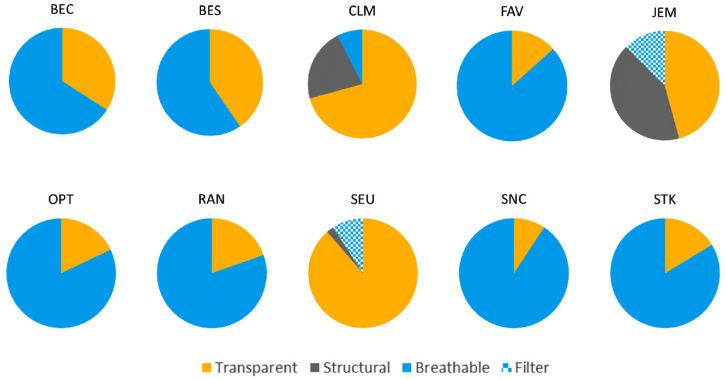
Areal proportions of transparent, structural, breathable, and filter portion for each transparent face mask.

We measured the transparent and remaining areas for each mask and arrived at percentages for each mask, as shown in Table 4 and Figure 4. Hereby, the solid area was distinguished between structural, breathable, and additional filter areas. In Table 4, the mass and total area of each mask are also given.

As mentioned above, when looking directly at the face of the user, CLM appears to have the most visible area followed by SEU and BES. However, by the measured areas of the entire mask, SEU has a more transparent portion than CLM and BES. This is due to the construction of the mask. In the case of SEU, the mask sits relatively tight on the face, and the area on the sides, i.e., the cheeks, is transparent. In case of CLM, the mask has more open areas on the sides, and in case of BES, there is more of the non-transparent portion on the sides. Besides some gaps between mask and face, a couple of masks had wider gaps due to the profile and contours of the masks, such as BEC and CLM.

### 3.2. Determination of Polymer Types

The materials in the face masks were mainly characterized by FTIR spectroscopy, since this was the most efficient method to characterize all parts of the masks, including the transparent portion, the breathable portion, any structural components, the nose bridges, and ear loops. NMR spectroscopy was not applied, since the thin coating on the inside and the outside of the transparent portion would have resulted in low signal–noise ratio and could not have been differentiated. In addition, one face mask, SEU, and several rubbery materials used in the ear loops would not have been soluble for regular NMR spectroscopy. As mentioned earlier, our emphasis was on the characterization of the transparent portions, since these distinguish the masks from already well-studied non-transparent face masks. Therefore, PHG is not included in the following figures.

In Table 5, the polymers found in the transparent portion of the face masks are shown with their repeat units.

**Figure 5:**
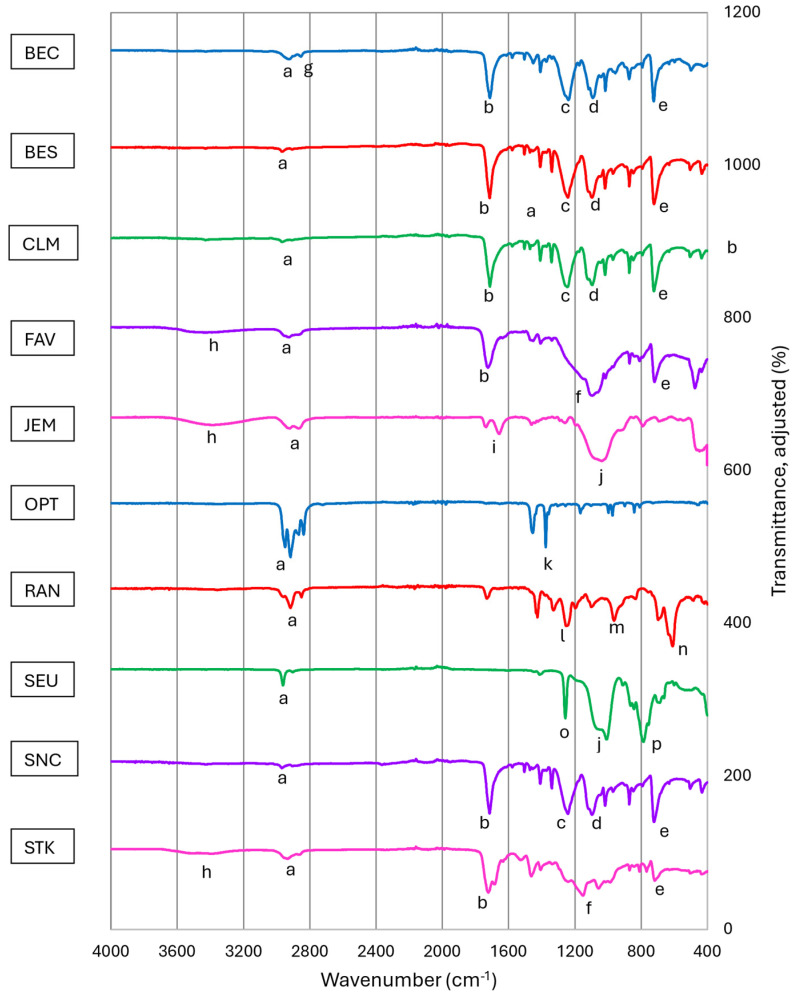
IR spectra of polymers found in transparent portions of face masks (outside layer; labels with letters are referred to in the text).

The IR spectra shown in Figure 5 were obtained by letting the outside (less close to the face) of the transparent material be closer to the ATR crystal. The IR spectra of the inside and outside of the transparent portions of the masks were similar except for nuances due to differences in the coatings, which may have been applied on the inside and outside of the transparent portions (details of IR spectra are shown in the Supplementary Materials). The differences between the inside and outside coatings on the transparent portions of the masks are further discussed in the subsequent section.

The masks BEC to FAV, STK, and SNC show the characteristic peaks for PET between 2800 and 3100 cm^−1^. (a) From aliphatic and aromatic C-H stretching vibrations, 1715 cm^−1^. (b) Due to C=O stretching of the ester group, as well as peaks at about 1240 cm^−1^. (c) At close to 1100 cm^−1^. (d) Due to C-O stretching of the ester group at 720 cm^−1^. (e) Due to the CH_2_ rocking peak of its methylene groups. For FAV and STK, also some overlapping peaks 1200 and 1100 cm^−1^. (f) Observed due to coatings further explained in the subsequent section. BEC shows peaks between 2920 cm^−1^. (g) Due to aliphatic C-H stretching caused by cyclohexanedimethanol and shifted to lower wavenumbers than for PET—the very broad and shallow peak between 3200 and 3500 cm^−1^. (h) In FAV, SEU, and STK the peak is due to stretching vibrations of O-H groups in hydrophilic coatings or absorbed water by silica. JEM shows peaks at about 2950 cm^−1^. (a) Due to C-H stretching vibrations of CH_3_ groups, which are caused by polycarbonate, the peaks being at close to 1640 cm^−1^. (i) From bending vibrations of absorbed water by silica, and the characteristic broad and intense peak between 1100 cm^−1^ and 1000 cm^−1^. (j) Mainly due to the stretching vibrations of Si-O-Si from the silica. OPT shows characteristic peaks between 2870 and 2950 cm^−1^. (a) Due to stretching of C-H bonds in the methylene and methine groups and a sharp peak at 1375 cm^−1^. (k) Due to the symmetric bending of the methyl group in iPP. The PVC in RAN shows additional peaks around 2920 cm^−1^. (a) Due to C-H stretching vibrations of its methylene group, has characteristic peaks at close to 1250 cm^−1^. (l) Due to bending vibrations in -CHCl-. PVC also shows peaks at about 965 cm^−1^. (m) Due to rocking vibrations of its methylene group and peaks between 610 and 700 cm^−1^. (n) Due to C-Cl stretching vibrations. The spectrum of SR in SEU shows a peak at about 2950 cm^−1^. (a) Due to C-H stretching vibrations in its methyl groups and one sharp peak at 1260 cm^−1^. (o) Due to bending vibrations of Si-CH_3_ and a large and broader peak between 1000 and 1100 cm^−1^. (j) Due to Si-O-Si stretching vibrations. (p) The peaks between 760 and 820 cm^−1^ are due to the combination of stretching vibration of Si-C and rocking vibration of the methyl group.

The main findings on the materials’ characterizations are shown in Table 6.

As given in Table 6, the material used in the transparent portion of half of the face masks was PET, while in the remaining half other polymers were used, namely, PETG, PC, iPP, PVC, or SR (silicone rubber). The dominant material in the breathable portion was iPP. The identity of the other portions of the face masks were also determined by FTIR spectroscopy (see spectra in Supplementary Materials). Polymers used in the nose bridge are polyurethane foam, and iPP or PVC as coatings on metal wires. For the ear loops, mostly either nylon 6, polyurethane elastomer (spandex), or PET was used.

### 3.3. Characterization of Coatings on Transparent Portions of Face Masks

The transparent portions of the face masks are typically coated with different types of materials. The inside preferably should be hydrophilic to absorb moisture exhaled by the user and thereby to reduce or eliminate fogging [42]. This can be achieved by adding materials such as polyethylene glycol, polyvinyl alcohol, or polyacrylic acid [43]. Frequently nanoparticles, such as silica or titanium dioxide, are used to make the surface super-hydrophilic. Low-volatility non-ionic surfactants, such as esters of sorbitan, may be added to lower the surface tension of the water and spread it further out [42]. The applied materials also need to show good adhesion to the main transparent portion of the face masks and be themselves transparent [44]. Crosslinked polymers may be used for higher durability [43]. These materials often are applied as multilayer coatings [45].

On the outside of the mask, scratch-resistant coatings are typically applied. In contrast to the anti-fog coatings, these are generally more hydrophobic. Examples are polyurethanes, polyacrylics, or silicon polymers [46]. Similarly to anti-fog coatings, nanoparticles, such as TiO_2_, SiO_2_, or ZrO_2_, may be included. Anti-scratch coatings also typically consist of several layers [47]. It is further possible that the surfaces of the transparent portions are grafted with a polymer [48]. Alternatively, the transparent portions are treated with plasma to introduce very thin layers of other groups, such as carbonyl groups, to enhance adhesion [49]. Furthermore, other materials such as UV stabilizers may be added [50].

Because five or more materials are typically applied in blends or different layers of the coatings and their amounts are relatively small, it would be beyond the scope of this work to exactly identify each of these materials. Instead, we tried to find some elements in the coatings beside those expected from the main elements in the transparent portion. This was carried out at two different depths. With XRF/EDX, elements that are a few micrometers and up to a millimeter deep into the material can be measured. With SEM/EDX, elements in the range from to 100 nm up to 5 μm can be detected, i.e., the surface is measured more than the bulk of the material. The exact depth depends on the type of element and electron beam energy.

**Figure 6:**
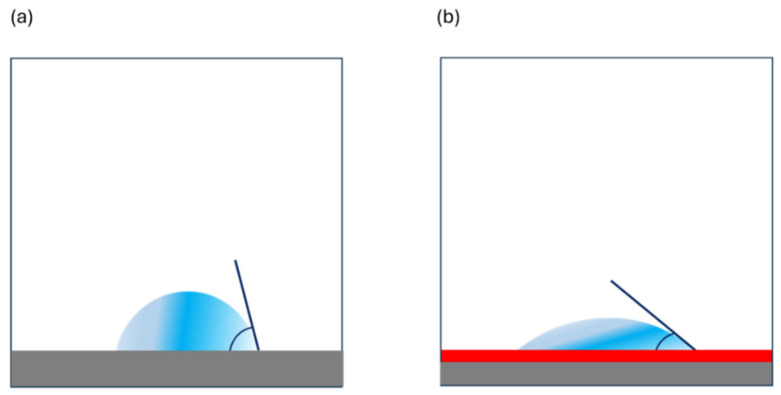
Schematic effect of anti-fog coating causing a decrease in the water contact angle: (**a**) droplet (blue) on less hydrophilic transparent polymer (grey) without coating and (**b**) droplet on more hydrophilic coating (red layer) on top of transparent polymer.

Furthermore, the water contact angles of the inside and the outside of the masks were determined. Water contact angle measurements directly allow for the determination of the wettability of the surfaces and the hydrophilicity of the coatings, as illustrated in Figure 6.

The results from the XRF/EDX and SEM/EDX measurements and water contact angle goniometry of the main substances in the coatings are given in Table 7.

The presence of silicon in FAV is most likely due to silicon dioxide used as nanoparticles. The nitrogen found in the outside layer of STK most likely is caused by the antimicrobial coating, mentioned by the manufacturer (3-(trimethoxysilyl) propyldimethyloctadecyl ammonium chloride).

With SEM/EDX, the obtained weight percentages shown in Table 7 are slightly higher for carbon and lower for oxygen than actual values, because the polymer samples were coated with a thin layer (5–20 nm) of carbon for the required conductivity. However, even if Pd/Au had been used for conductivity, the carbon and oxygen contents would not have been accurate because of interference of the signals of the heavier elements with those of the lighter elements. In addition, with both EDX methods, matrix effects can occur that would give slightly incorrect values for C and O.

The contact angles for the inside coating with the average of the bulk material for each mask were compared. For the following masks the difference between these contact angles were more than 10%, given in the following in increasing order of hydrophilicity of the inside coating: SEU, SNC, OPT, CLM, JEM, FAV, and STK. For the following masks, there was only a small difference (less than 10%) between the contact angles of the inside coating and the bulk (given in decreasing order): BES, RAN, and BEC. For BEC, the manufacturer stated that it had been coated inside with “Nerdwax”, which is most likely a blend of hydrophobic materials.

**Figure 7:**
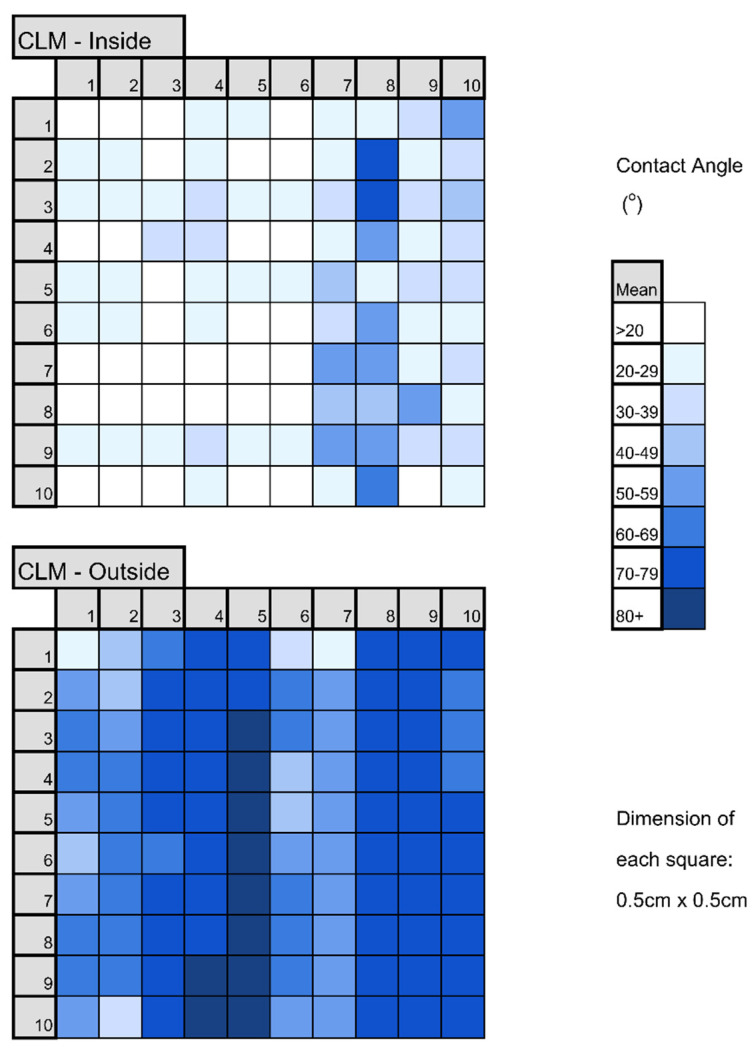
Non-uniform pattern with different contact angles (^o^) for CLM.

Not all the transparent portions of the face masks were uniform. Particularly, BEC had a non-uniform coating, as could be simply observed as a large uneven spot in the center of the mask. In the case of CLM, a great variation of the contact angle was observed, as shown in Figure 7. The inside surface may have been treated with an anti-fog agent; however, it appears that it was not spread uniformly. Also, the outside, untreated surface had varying contact angles. These variations in contact angles may arise due to different degrees of roughness on the surface, which is known to influence the contact angle.

### 3.4. Quantitative Optical Properties—Reflectance and Haze

Some of the visual properties could be directly obtained by viewing the photos in Figure 2 and were already commented on. In the following, two quantitative properties that are related to the visibility of the transparent face masks, reflectance and haze, are eluded to. The reflectance indicates how much light is reflected by the material at different wavelengths compared to the total incident light. The haze is determined as the amount of scattered light divided by the transmitted light, in %.

In Table 8, the data of reflectance and haze of the transparent portion of the face masks are shown.

Generally, the lower the reflectance or the haze are, the better. RAN has the lowest UV-Vis reflectance, while OPT and SNC have the highest reflectance. Hereby, the reflectance within the material is measured. Therefore, there is no strong correlation with the reflections when looking like a viewer at a face mask. The latter depends much on the curvatures of the mask, as shown in Figure 2. The UV-Vis-based haze correlates to the haziness of the transparent portions of the face masks. The mask with the haziest transparent portion is OPT. In addition, the film of OPT is soft, does not stay flat, and rather tends to form waves. RAN had the least haze and looked relatively clear, also in its photo, shown in Figure 2. The wave patterns in some of UV-Vis transmittance graphs are due to the presence of coatings. Graphs for the UV-Vis reflectance and UV-Vis haze are provided in the Supplementary Materials.

### 3.5. Thermal Properties, Density, and Degree of Crystallinity of Transparent Portions in Face Masks

Knowing the described chemical compositions of each of the transparent face masks is critically important, and they are all composed of polymeric materials (with the exception of some metal in the nose clips). However, any polymer of a certain type can still vary in several of its properties. For example, a polymer of a given type can have relatively low or high crystallinity, or low versus a high molecular weight. Its other macroscopic properties such as strength or transparency will then also vary. We therefore found it necessary to determine these specific properties for each of the polymers, particularly in the transparent portions of the face masks.

**Figure 8:**
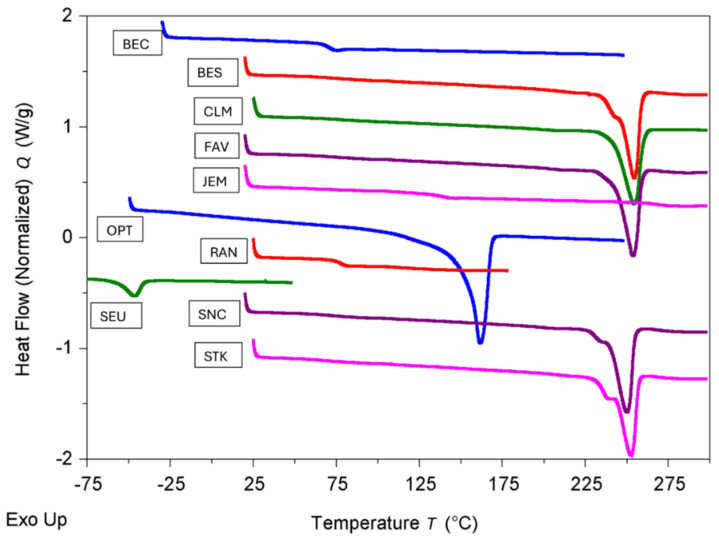
DSC traces for 3rd heating of the polymers in the transparent potions of face masks.

**Figure 9:**
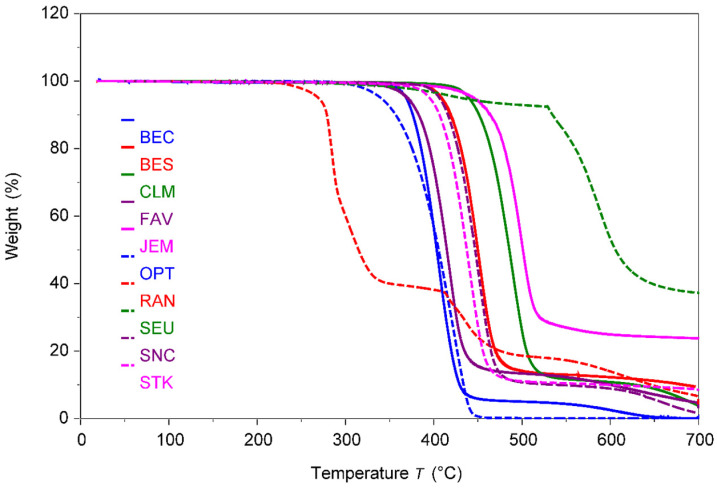
Thermogravimetric curves for transparent portions of polymers.

In Figure 8, the DSC traces for the third heating of the polymers in the transparent portions are shown. The third heating is to eliminate the processing dependent thermal history of the polymer. The TGA curves are shown in Figure 9.

In Figure 9, the curves for the Thermal Gravimetric Analysis (TGA) of the polymers in the transparent portions of the face masks are shown.

In Table 9, the densities, thermal properties, and degrees of crystallinities of the polymers in the transparent face masks are given.

As is apparent from Figure 8 and Table 9, about half of the polymers have a melting peak indicated by a large endotherm and are semi-crystalline, i.e., the five PET samples and iPP. The values of the degrees of crystallinities obtained from the density differ from those measured by DSC, because of the measurement method, as has been observed previously [57]. However, the trend of the crystallinities from the density and the melting peaks are identical. The polymer with the highest degree of crystallinity is iPP used in OPT. Typically, a higher degree of crystallinity causes more opaqueness in a polymer.

The remaining polymers only show inflections at the glass transition temperature (T_g_) and are amorphous (SEU, which shows some hysteresis peak at T_g_). Generally, the higher the value of T_g_ compared to the ambient application temperature, the more rigid the polymer is. BEC, JEM, and RAN contained amorphous polymers in their transparent portions, which only produced glass transition temperatures. The glass transition temperature usually indicates the rigidity of the polymer. Therefore, JEM containing SiO_2_ and PC in its transparent portion is the most rigid. In the case of SEU, a glass transition could not be observed.

Though the application temperature for the face masks is expected to be in the range of ambient temperatures (−20 to 40 °C), the TGA curves, shown in Figure 9 and Table 9, still help to distinguish the materials thermal degradability. From the thermal degradation, properties such as general stability, presence of blends, ease of processing, sterilizability, presence of by-products or volatiles, and performance in pyrolysis or incineration can be inferred. Interestingly, the four PETE-based masks show some distinction. While CLM is the most heat-stable (solid green), FAV is the least heat-stable (solid violet). The latter may be because of the coatings in FAV. Overall, the most heat-stable polymer is SEU, while RAN is the least heat-stable. Also, JEM is with T_dec_ of close to 500 °C relatively stable and more stable than pure polycarbonate due to the presence of a high amount of silica gel. As is typical of the PVC in RAN (red, dashed), there are two decomposition steps, the first being due to the release in HCl and its reaction with the aluminum pan used in the DSC instrument [58]. At 700 °C, SEU resulted in the highest residue with close to one-third of polymer remaining non-decomposed, while PP in OPT resulted in no residue at all. All polymers are sterilizable under regular steam or in an autoclave up to ca. 150 °C.

### 3.6. Molecular Weight Properties

In Table 10 the molecular weight properties of the polymers are shown.

The molecular weight averages lie in the expected range of each polymer. All PET-type polymers have very similar molecular weight properties. Based on the previous findings through FTIR spectroscopy and the thermal properties, one can assume that the PET in FAV has also similar molecular weight properties to the other four PET-based polymers. The lowest molecular weight is that of PC in JEM. The highest molecular weight is that of PP in OPT, which is not a typical for this chain-addition polymer. The polydispersity indices of most of the polymers lie between 1.32 and 1.99. The high PDI of OPT indicates that it is made with a heterogeneous Ziegler/Natta catalyst and not a homogenous single-site catalyst, the latter having PDIs of close to 2.0. For SR in SEU, no molecular weight properties could be measured since it is a crosslinked polymer.

### 3.7. Mechanical Properties

**Figure 10:**
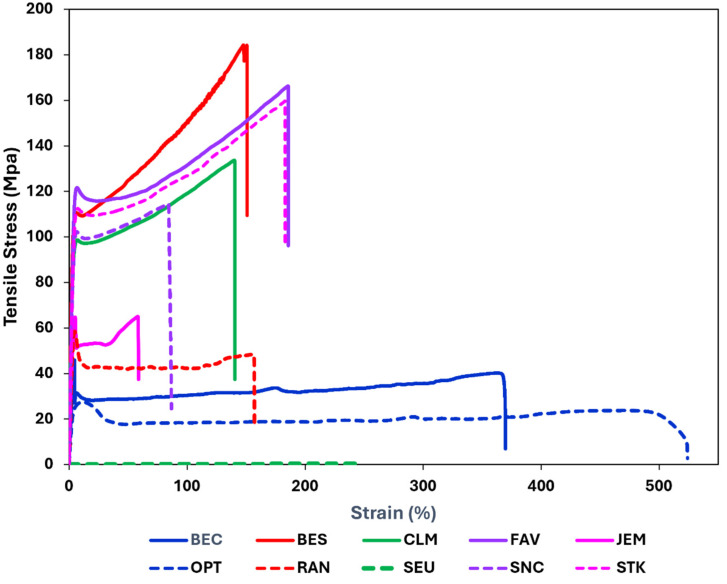
Representative curves for stress versus nominal strain of polymers in transparent portions of face masks (comments in footnote of Table 11).

In Figure 10, the stress/strain curves of the transparent portions of the face masks are shown, and in Table 11, the values of the tensile strength, elongation, and moduli are listed.

Some of the PET-based materials were the strongest, namely, those in BES and FAV, whereby the weakest material in this category was in SNC, most likely because it could not be measured in vertical direction. The weakest material overall was SR used in SEU. The materials with the highest elongations were the PP in OPT, PETG in BEC, and SR in SEU. Based on the moduli, the stiffest material is the PET in BES, and the most flexible the SR in SEU. The softest material is SR in SEU. Also, still relatively soft are PP in OPT and PETG in BEC. PVC in RAN and PC in JEM can be considered tough. The PET in BES, FAV, SNC, and STK may be considered relatively hard and tough [59].

### 3.8. Overall Assessment

To assess the determined properties of the 10 different transparent face masks, those properties were grouped by the main criteria that were described in Figure 1, i.e., protection, visibility, comfort, and sustainability. Each main criterion was divided into subcategories, shown further below and in the Supplementary Materials. Altogether, 28 properties were assessed, each of which was ranked typically from 0 to 10, with 10 being the most favorable. The properties were also weighted between 0.7 and 1.0. The weights were primarily assigned based on the relevance of a property and its reliability, i.e., the weights were reduced when the assessment was not quantifiable or less clear, which is explained for each property in the Supplementary Materials. The weight (W) was the product of the relevance (Rv) multiplied with the reliability (Rb), i.e., W = Rv × Rb. The points per property (PpP) was the product of rank multiplied with the weight, i.e., (PpP = Rk × W).

Two examples of how the assessment was applied are shown in Table 12 and Table 13. All 33 tables are included in the Supplementary Materials, with comments on the properties when feasible, their relevance, and their reliability. The reason there are 33 tables and only 28 properties is that some properties were evaluated under two or more of the main categories. These are the type of “polymer in the transparent portion” under three subcategories of sustainability: reusability, recyclability, and biodegradability. In addition, the “mass” was evaluated both under comfort and sustainability, and the “nose bridge material” under protection and comfort.

Table 12 is an example of applying the assessment for a non-quantitative property, while Table 13 serves as an example of applying a quantitative property. More than half of the properties were assessed based on quantitative or Boolean comparisons. In Table 12, the qualitative property (presence of a filter) was ranked manually between 0 and 10, with 10 being the highest or best. In this case, a simple Boolean comparison could be made, i.e., either a filter was present with a rank of 10, or not with a rank of zero. A weight of 100% was applied, because a filter is highly relevant, and the original data were highly reliable. In cases where a larger property had a negative effect, the opposite ranking was applied (i.e., ranking by a descending instead of ascending order). Any partially subjectively appearing properties were estimated upon consensus within our research group and are further explained in the Supplementary Materials. The criteria for ranking more complex properties, such as recyclability under the category of sustainability, also are alluded to in the Supplementary Materials.

For Table 13, the measured data for the Thickness under the category of sustainability was assessed. Generally, a thinner material for the same target application (here, the transparent portion of the face mask) is preferred since it is more economical and produces less material waste when it must be discarded.

**Figure 11:**
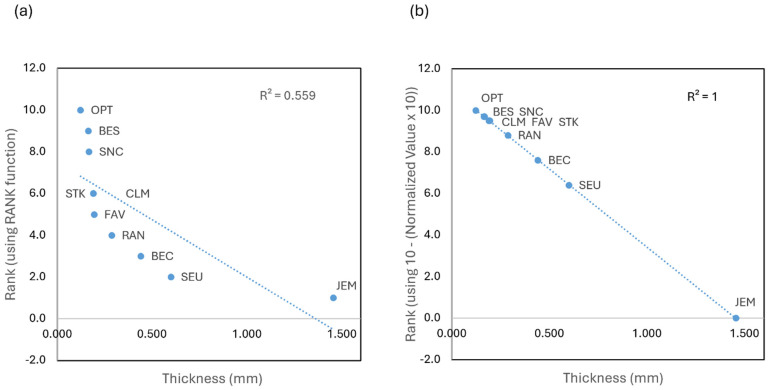
Exemplary graphs illustrating ranking methods for the assessment: (**a**) using the RANK function and (**b**) based on normalized values and applied for the actual assessment.

One way to rank the value for the thickness (in mm) is by using MS Excel’s “Rank” function. Because in this case the ranking increases with decreasing thickness, an order of 0 (descending) was applied instead of 1 (ascending). The resulting ranks are shown in the third column of Table 13. However, as shown in Figure 11a, this ranking function would result in different ranks, even when the numbers for the thicknesses are relatively close, i.e., not significantly different. For the specific example, the thickness of nine of the masks only increased by less than 0.5 mm or 38% of the entire range but would have caused a change in rank from 10 to 2. That this direct ranking method is poor (using MS Excel’s RANK function or its other direct RANK functions) is also indicated by the small coefficient of determination, R^2^., e.g., for Figure 11a being only 0.559.

Therefore, we used a different, more meaningful method of ranking, resulting in the ranks shown in the fifth column of Table 13. We preferred calculations rather than adjusting each ranking graphically (e.g., using Figure 11a as a starting point) due to the higher degree of objectivity using calculations. The following method is also simpler than the alternative of ranking using IF statements.

The values first were normalized, i.e., the minimum value Min and the maximum value Max, and then the ranges as (Max–Min) were calculated. Then, for each of the 10 masks, the individual value, V, was applied using the formula for the rank in ascending order: Rank_asc_ = (V-Min)/(Max − Min) × 10. The result was rounded to the first decimal to have more differentiation for values between zero and 10 than with just the integers. This way the mask with the minimum value ends up with a rank of zero, and the one with the maximum value with 10, as in most of our assessments.

For quantitative data, which needed to be ranked in descending order as Rank_desc_, (like in the shown case for the thickness), the same method for obtaining the rank in ascending order, Rank_asc_, was applied; however, the initially calculated Rank_asc_ was subtracted from 10, i.e., Rank_desc_ = 10 − Rank_asc_. As shown in Figure 11b, due to the normalization, one obtains a coefficient of determination R^2^ of 1. Also, the thicknesses, which are closer to each other, are now at the same rank (i.e., for BES and SNC at 9.7, and for CLM, FAV, and STK at 9.5).

The results of the overall assessment for all properties is shown in Table 14.

The assessment resulted in STK being the best mask and CLM being the worst mask overall. No single mask was rated highly on all or most of the properties, and for many masks, the high ratings for some properties were compensated by low ratings in other properties. This is due to several of the properties being opposed to each other. For example, larger visibility goes on cost of less efficient filtering through the breathable portion, unless a specific filter is present. It should be noted that of the 271 total points that were available, the score for best mask (STK) was 62%, and for the worst mask (CLM) 51%. These results underscore the need for a superior transparent face mask as mentioned in the Introduction. The assessment also showed that not only a few main properties were critical. For example, both the highest- and lowest-ranked mask contained PET as the main material in the transparent portion. Within each category of the main criterion, the following masks were rated best: protection: SEU; visibility: STK; comfort: OPT; sustainability: RAN.

**Figure 12:**
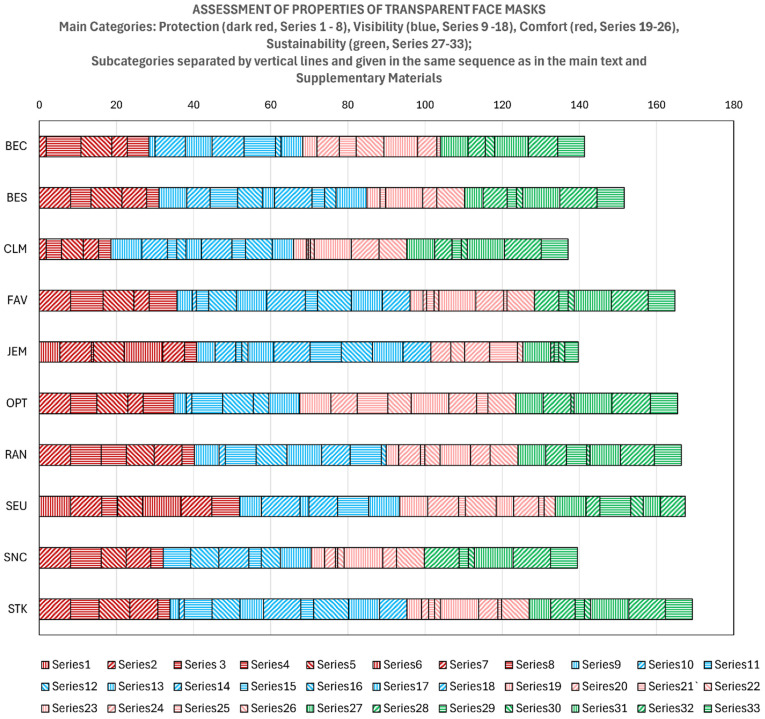
Assessment of properties of transparent face masks.

The graphic results of the assessment are shown in Figure 12.

For each main criterion, the following measured properties were applied (number in brackets is equal to number of the series in Figure 12). Protection was based on (1) seal, (2) major gaps, (3) availability of sizes, (4) breathable area, (5) breathable material, (6) filter presence, (7) nose bridge material, and (8) types of governmental approvals. For visibility, the considered properties were (9) clear area around mouth, (10) transparent area, (11) apparent distortion, (12) apparent reflectance, (13) UV-Vis reflectance, (14) UV-Vis haze, (15) degree of crystallinity, (16) contact angle, (17) inside (anti-fog) coating, (18) outside (anti-scratch) coating. Comfort was based on (19) glass transition temperature, (20) stress, (21) strain, (22) modulus, (23) mass, (24) nose bridge material, (25) ear loop material, and (26) ease of assembly. For sustainability, the subcategories were (27) reusability, (28) recyclability, (29) renewability/petroleum, (30) biodegradability, (31) mass, (32) thickness, and (33) price. More details on all assessment criteria and data are shown in the Supplementary Materials. If for a designated property no bar segment is shown in Figure 12, it means the value of that property is zero.

### 3.9. A Closer Look at the Assessment of Sustainability-Related Aspects

Among the sustainable subcategories, most preferrable is reusability, before recyclability, because of the lower cost of the former. Regarding reusability, two masks were advertised as “disposable” (FAV and SNC), which is a less sustainable option than the other masks, which were labeled as “reusable”. In some cases, it was indicated by the manufacturer how often the masks could be reused before being disposed. For example, BEC can be used up to ten times or 80 h, and STK is reusable for a week. In case of SEU, the mask can be washed with soap and water. One issue with cleaning the masks with water or some disinfectants, such as isopropanol or ethanol, is that the anti-fog coating may be removed or impaired.

In a comparative study from 2018, the U.S. Department of Energy reported that PET is with 15% the most recycled polymer within its type, whereas only 3% of PP is recycled [60]. The recycling rate of PET bottles in 2023 was with 33% the highest among polymer products [61]. However, OPT and SNC are easiest to recycle, since both the transparent as well as the solid portion are made of the same material, iPP or PET, respectively. For several masks, the parts made of different polymers need to be separated before recycling, which, particularly in case of JEM, is more of a challenge. Polymers, which are not specifically recycled polymers, such as PC in JEM or SR in SEU, can be viewed as having a disadvantage. It is preferable that the coating on the transparent portion does not represent a significant mass, since it could have an impact on the properties of the mainly recycled polymer. Any polymers in the nose bridges and ear loops were generally different from the polymers in the transparent or breathable portions, and should be separated for efficient and specific recycling.

Another important criterion regarding sustainability is renewability, i.e., the use of plant based or other renewable resources instead of fossil fuels. The renewability of polymers can be expressed as kg polymer/L petroleum or kg polymer/kg petroleum (petroleum has a density of about 0.8 kg/L). Among the polymers used most in the transparent face masks, several sources estimate that the production of PET requires more petroleum than that of PP (some stating that 1 kg of PET requires 1.9 kg petroleum, whereas 1 kg PP requires 1.7 kg petroleum) [62,63]. Among the other applied polymers, it takes the least amount of petroleum to produce PVC and the most to produce PC (data for PETG are close to those of PET; no data for SR are available) [64,65].

None of the face masks were made to a larger extent from a highly sustainable polymer. Only one mask (SEU) contained 6.2 weight % of polylactic acid (PLA), a renewable and biodegradable polymer, in its filter portion. In RAN, cotton is used for the main solid portion. However, cotton fiber lacks some of the advantages a fiber made from iPP, such as higher efficiency in filtration. Aliphatic polyesters, such as polyhydroxy alkanoates (PHAs), PLA, polybutylene succinate (PBS), polycaprolactone (PCL), and polyglycolic acid (PGA), are biodegradable [66]. PC (in JEM) or PET or PETG (in the remaining five polymers), are not considered biodegradable; however, they have a potential to degrade under conditions that could cause hydrolysis of their carbonate or ester groups, respectively. Generally, pure chain addition polymers, such as PP (in OPT), PVC (in RAN), or SR (in SEU), are not biodegradable and require additives for their degradation [67].

It is more sustainable to produce any plastic product with the lowest possible mass for its application, a trend that has been applied much in packaging for the last decades. This is collectively often summarized under “reduce”. In this regard, the most sustainable mask was SNC, and the least sustainable JEM. For a similar reason, a thinner mask is preferable, with OPT being the most sustainable and JEM again the least. Since sustainability depends on three components, that is, environmental, economic, and social, one also needs to include the price of the masks as well. A lower price is preferable because of the economic aspect of sustainability, i.e., lower production cost, as well as the social aspect of sustainability, i.e., making the mask more affordable for a larger population. SEU is by far the most expensive and therefore should be considered least sustainable, and BEC and SNC are the most sustainable in these regards.

### 3.10. Recommendations for Improvements

The recommendations made here go beyond those made for solid face masks, mentioned in the literature, some of which are referenced earlier in this article.

To achieve high protection, N95-approved masks should also be a requirement for transparent face masks. It is highly unlikely to create a polymer that would have excellent optical properties and be efficient in filtering viruses at the same time. For the filtering, polymer fibers with a high degree of crystallinity are typically preferable, whereas optical clarity is obtained with polymers with a lower degree of crystallinity. Therefore, high protection can only be achieved by the presence of a sufficient breathable portion or a designated filter. The transparent portion should have a shape that closely matches an average face to avoid any significant gaps. The mask should be offered in three different sizes: large, medium, and small. A more flexible polymer in the transparent portion with a relatively low glass transition temperature is preferable to better adjust while speaking, while staying tight on the face. The mask also should contain a soft seal at the edges of the mask, which better adjusts to contours of a face. Nose bridges should be preferably plastic-coated aluminum wire. Ear loops should be made of a stretchable rubber with a soft surface, preferably silicone rubber.

Because several important properties influencing the visibility are contrary to each other, they need to be optimized. For example, the visible area needs to be maximized, so it is large enough to see the moving mouth including its surroundings, as well as having sufficient solid portions to allow for efficient filtering. At least one-fifth of the mask should be transparent, allowing for sufficient visibility. For better visibility and a larger transparent area, a separate filter would be more suitable. Optical properties of polymers with lower crystallinity and low haze are preferred. For example, syndiotactic polypropylene may be preferable to isotactic polypropylene. The shape of the transparent portion should be low in curvature to avoid glare and distortions. To reduce fogging use of a thin and uniform coating of a hydrophilic material on the side facing the mouth is recommended. The coating should contain a combination of materials to be sufficiently transparent, adhere to the main transparent material and be crosslinked so it is more durable. The material should contain nanoparticles such as silica to render the coating super-hydrophilic. On the outside of the mask, the transparent portion should be coated with a scratch-resistant coating, containing, for example, polyurethane.

For more comfort, the transparent polymer should have a low mass and density to reduce user fatigue. It should have a low glass transition temperature so the entire mask can move easily with jar movements. Better candidates would be polyolefins such as PP (T_g_ = −20 to −10 °C) or certain step-growth polymers, such as polycaprolactone (T_g_ = −62 °C). The lowering of the glass transition temperature may be also achieved by adding a plasticizer, such as triethyl citrate. The mask should be easily and swiftly be placed on the face, not requiring assembly of several parts. The transparent portion of the mask also needs to have optimal mechanical properties. Its tensile strength should be sufficiently high to maintain durability, but low enough to provide flexibility for comfortable speaking. Also, the transparent material needs to be sufficiently strong so it does not deform to a level that would impair visibility. Similarly, the modulus should have an optimized value. The mask material should allow for sufficient elongation so it slightly stretches during speaking, allowing for speech clarity and less user fatigue.

A reusable mask is generally preferable to a disposable mask based on the economics as part of sustainability. The polymers must withstand disinfecting agents, such as mild detergents or alcohol. However, a reusable polymer will also eventually reach its “grave” at the end of its life cycle, and the polymers used in it need to be recyclable or biodegradable. With recycling rates being generally relatively low, the applied plastics preferably should be thermomechanically recyclable. One also needs to bear in mind that most of the discussed masks are based on multi-component polymers, i.e., contain a solid portion that is made of a different plastic than the transparent portion and other parts, such as filters or ear loops. To recycle the materials in most of the masks with high efficiency, those parts would need to be separated and sorted to end up in a specific recycling stream. Alternatively, a mask consisting of only one major polymer or two easily separable components should be considered. If a disposable mask is offered, it needs to be biodegradable to avoid pollution in landfills or in the marine environment.

PET has been frequently used for the transparent portion since it is highly recyclable. However, polyethylene furanoate (PEF) has very similar properties to PET and additionally is renewable [68]. PEF can be obtained from glucose and fructose originating from lignocellulose. Alternatively, bio-based PET, which is synthesized, for example, from bio-based isobutanol and bio-based para-xylene, could also be used as starting materials [69]. A sustainable counterpart for the frequently used iPP (isotactic polypropylene) in face masks, including transparent types, would be biobased PP obtained from sugarcane [70]. Another more sustainable alternative to iPP is polybutylene succinate (PBS). The mechanical and processing properties of PBS and PP are very similar [71,72]. PBS can be obtained from biobased starting compounds, such as biobased 1,4-butanediol and bio-succinic acid, which can be produced by microbial processes. PBS and PEF are biodegradable, but at a rate that allows for them to stay intact during the lifetime of the mask, in contrast to, for example, polyhydroxyalkanoates (PHAs), which degrade at a much higher rate. Furthermore, one could use copolymers containing renewable and stable antibacterial moieties such as eugenol [73] or antiviral agents such as copper [74].

In general, polymers that produce optimal target properties at the lowest possible mass (below 10 g) and thickness (below 0.3 mm) need to be selected. In addition, the polymers need to be inexpensive, i.e., most of the polymers should cost less than USD 5/lb (e.g., PBS and PEF fall in this category). Also, the packaging of the mask should be minimal. Besides injection molding and thermoforming, masks could be produced by additive manufacturing. Preferably, the energy used for the manufacturing process should be green, e.g., based on solar power.

In special instances, a “smart transparent mask” may be desired, which could include a fan, a microphone to improve speech quality, or diagnostic tools, such as a colored fever thermometer or a COVID-19 sensor, as well as Bluetooth connectivity.

## 4. Conclusions

Altogether, we measured 28 properties of 10 different transparent face masks. Several properties varied over a wide range. For example, the percentage of the transparent to solid area differed between 8 to 89%. Tensile strengths of the transparent portions also varied over a wide range, from 0.248 to 186 MPa. Our focus was on the characterization of the transparent portion of each mask, since the solid portions were made of commonly used and well-studied iPP. In half of the masks, PET was used as material for the transparent portion. The inside of most of the transparent portions showed low water contact angles due to hydrophilic coatings.

A perfect transparent face mask is not commercially available, yet. STK was the highest-scoring mask. However, based on our assessment, it scored only 62% of all available points. The abovementioned findings should be useful to develop a transparent face mask that will fulfill the most important requirements. A preferable mask needs to perform well in all of the major criteria: protection, visibility, comfort, and sustainability. Six of the ten masks were lacking greatly in protection. Only two of the masks included designated filters (JEM and SEU). Only one of the ten masks was N95-approved (OPT). Furthermore, none of the masks investigated were highly sustainable. It would be most preferable to produce reusable masks mainly from renewable and biodegradable polymers.

It will be also important to directly determine the flow properties of the masks, which will be discussed by one of our team members, Jennifer Schneider, in a future publication. Additionally, a user-focused marketing study will be helpful to evaluate insights into perceived visibility, comfort, acoustics, and aesthetics, which some of our team members have already initiated.

## Figures and Tables

**Table 1:** Face masks characterized in this work, including manufacturers’ website addresses.

Entry No.	Brand Name	Abbreviation	Approx. Price Range per Mask in USD	Information at Amazon or Specific Website, as of 30 November 2024
1	BEclear	BEC	n. a.	https://www.facebook.com/BEmask.org/
2	Bendshape	BES	5–10	https://kangaroo-ruby-a78y.squarespace.com/shop
3	ClearMask	CLM	5–10	https://www.theclearmask.com
4	FaceView	FAV	2–5	https://faceviewmask.com
5	Jelli M1	JEM	30–40	https://jellim.com
6	Optrel	OPT	1–2	https://optrel.us
7	RafiNova	RAN	3–7	https://rafinova.com
8	SeeUS95	SEU	48–85	https://seeus-95.com
9	SafeN’Clear	SNC	1–1.5	https://safenclear.com
10	Stek	STK	3–4	https://stekcare.com
11	Protective Health Gear	PHG	0.6–1	https://protectivehealthgear.com

**Table 2:** General information provided by manufacturers.

Abbreviation	General Comments Provided by the Manufacturers from Their Websites and on Packaging
BEC	Lightweight, transparent, reusable, recyclable, inexpensive, KF94 mask + BeClear Adapter; “nerd wax antifog” is pre-applied.
BES	Anti-fog transparent front. ≥98% BFE, ≥98% PFE @ 0.1 micron (“Highest filtration”; reusable—up to 10X or 80 hours; remark on sizes mentioned, but not posted.
CLM	FDA cleared (class II), CE marked (class I); applicable ASTM level 3 and EN 14683 standards; anti-fog technology, latex-free; in package: polyurethane foam; adult and child size.
FAV	This product meets requirements for KN95 and effectively filters at least 95% of particles; latex-free; Nonwoven complex fiber, melt-blown nonwoven fiber, hydrophilic Toray film, nylon and spandex ear loops, polyurethane sponge nose strip, aluminum nose strip; disposable.
JEM	Comes with replaceable filters (20 sets of three-piece filters that last from 2 to 3 weeks with regular wear, USD 5 ea.); anti-fog surface; breakdown: 29.92% PC, 31.93% nylon, 37.94% silica gel, 0.21% melt-blown
OPT	The P.Air Clear N95 Respirator is the world’s first NIOSH-approved and FDA-cleared face mask with a transparent window; latex-free; anti-fog coating. In package: straps: latex-free elastic; nose clip: metal; nose foam: polyurethane; window: polypropylene; filter: polypropylene.
RAN	BPA-free clear panel; tie-behind and ear loop styles; adjustable nose bridge; no anti-fog coating (recommended to obtain); four mask sizes (adult, teen, kids, little kids).
SEU	Adhesive on perimeter, gentle to the skin. Will adhere directly to face for better seal; reusable/washable with soap and water; BIO-FILTER 95% filtration efficacy; Washable filter with no rating, comparable to cloth mask; main part made of “silicon”; filters: PLA, bamboo. Anti-fog coating. The filter is N95-approved; large and small size.
SNC	Single-use, disposable; Level 1: FDA-registered and approved device meets ASTM Level 1 surgical mask standards; Level 3: ASTM Level 3, fog-resistant; “Fog resistant” window.
STK	Reusable for one week, anti-fog-coated; antimicrobial cover (3-(trimethoxysilyl) propyldimethyloctadecyl ammonium chloride); nonwoven fabric (exterior, lining, filter), polyester (nose wire), spandex (string), PET film; anti-fog; scratch resistance.
PHG	Completely solid (used as reference); NIOSH-approved N95 respirator; adult and child sizes.

**Table 3:** Explanations of standards used for face masks mentioned in Table 2.

Standard	N95	KN95	KF94
Country	United States	China	South Korea
Standard	NIOSH	GB2626-2019	KMOEL-2017-64
Filtration Efficiency	≥95% of particles 0.3 microns or larger	≥95% of particles 0.3 microns or larger	≥94% of particles 0.3 microns or larger
Typical shape	E.g., cup style or duckbill style	E.g., flat-fold or tent like style	E.g., larger cup or boat style
Fit	Tight	Less tight than N95	Less tight than KN95
Fastenings	Two headbands	Two ear loops	Two ear loops
Applications	Healthcare, other industries	Public, healthcare	Public
Other categorizations	RS2: reusable DS2: disposable	-	-
References	[37]	[38]	[39]

N in N95 and KN95 stands for “Not” approved for oil mists or oily environments.; in the United States, there are also masks with higher efficiency, such as N99, which filters at least 99% of particles, but is not required for face masks against COVID-19; FDA approvals on the masks or packaging [40] can vary and potentially present a red flag, meaning that the masks may not adhere to any of the three defined standards.

**Table 4:** Basic physical properties of the transparent face masks.

Mask ID	Mass (g)	Total Area (cm^2^)	Transparent Area (cm^2^)	Thickness of Transparent Part (mm)	Functionality of Portion of Total Area (%)				Seal	Wider Gaps Between Mask and Face
					Trans-paent	Structural	Breathable	Filter		
BEC	15.30	444.37	151.49	0.441	34.09	0.00	65.91	0.00	No	Top
BES	8.75	301.64	121.83	0.165	40.39	0.00	59.61	0.00	No	No
CLM	9.07	192.69	131.28	0.l90	68.13	21.03 *	7.22 *	0.00	No	Sides
FAV	8.72	317.88	42.44	0.195	13.35	0.00	86.65	0.00	No	No
JEM	78.31	242.67	111.07	1.456	45.77	41.67	0.00	12.55	Yes	No
OPT	7.36	274.97	49.33	0.123	17.94	0.00	82.06	0.00	No	No
RAN	21.45	268.74	52.81	0.288	19.65	0.00	80.35	0.00	No	No
SEU	45.68	208.33	184.62	0.600	88.62	2.15	0.00	9.23	Yes	No
SNC	5.94	288.76	26.91	0.168	9.32	0.00	90.68	0.00	No	No
STK	6.90	290.96	47.57	0.190	16.35	0.00	83.65	0.00	No	No

*: sponge area considered 66.7% structural and 33.3% breathable; nose bridges and ear loops had a negligible effect on main area.

**Table 5:** Polymer types with their repeat units found in the transparent portion of face masks.

Entry No.	Mask ID	Polymer Name	Polymer Acronym	Polymer’s Repeat Unit
1	BEC	Polyethylene terephthalate glycol (with 1,4-cyclohexane-dimethanol)	PETG	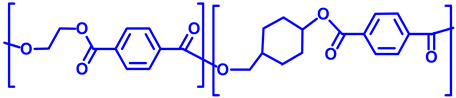
2 3 4 9 10	BES CLM FAV SNC STK	Polyethylene terephthalate	PET	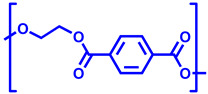
5	JEM	Polycarbonate	PC	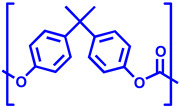
6	OPT	Isotactic polypropylene	iPP	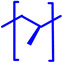
7	RAN	Polyvinyl chloride	PVC	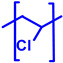
8	SEU	Silicon rubber (crosslinked)	SR	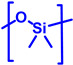

**Table 6:** Materials used in different portions of face masks.

Mask ID	Transparent Portion	Breath-Able Portion	Filter	Structural Portion	Nose Bridge	Ear Loop ^a^	Seal or Adhesive
BEC	PETG	iPP	-	-	iPP-coated wire ^b^	Nylon 6	(Chin gel: PU strip: PMMA^c^)
BES	PET	iPP	-	-	Al strip	PET	-
CLM	PET	PU foam	-	PU foam	PU foam	PET	-
FAV	PET	iPP	-	-	PU foam ^d^	Nylon 6/spandex	-
JEM	SiO_2_/PC	-	iPP	Nylon	SR	SR ^e^	SR
OPT	iPP	iPP	-	-	PU foam	NBR	-
RAN	PVC	Cotton/PET	-	-	PVC-coated wire	PET	-
SEU	SR	-	PLA	PLA	(Sil. adhes. strip)	-	Sil. adhes. strips for sides
SNC	PET	PET	-	-	Al strip	PET	-
STK	PET	iPP	-	-	iPP-coated wire	Nylon 6/spandex	-
PHG	-	iPP	-	-	Al strip	Nylon 6	-

Polymers identified by FTIR spectroscopy; a: may contain cotton; b: nose bridge not used, since on chin; c: strip connects transparent portion to solid portion; d: combined with Al (aluminum); e: includes wider band made of SR on back of neck (see Figure 3); iPP: isotactic polypropylene; NBR: acrylonitrile–butadiene rubber; PC: polycarbonate; PET: polyethylene terephthalate; PETG: polyethylene terephthalate glycol; PLA: polylactic acid; PU: polyurethane; PVC: polyvinyl chloride; SR: silicon rubber; Sil. adhes.: silicon adhesive.

**Table 7:** Elemental composition and contact angles of coatings on the transparent portions of face masks.

Mask Type	Contact Angle of Main Portion ^a^	Inside Coating				Outside Coating			
(Main Material in Transparent Portion)		Element (wt.%) by XRF /EDX ^b^	Element (wt.%) by SEM /EDX ^c^	Contact Angle of Coating ^d^	Contact Angle, Std. Dev. ^e^	Element (wt.%) by XRF /EDX ^b^	Element (wt.%) by SEM /EDX ^c^	Contact Angle of Coating ^d^	Contact Angle, Std. Dev. ^e^
BEC (PETG)	65–86 [51]	C: 67.5 O: 32.5	C: 76.3 O: 23.7	74.9	4.18	C: 70.2 O: 29.7	C: 77.5 O: 22.5	73.3	1.89
BES (PET)	66–81 [52]	C: 71.3 O: 28.5	C: 77.4 O: 22.6	62.5	8.99	C: 71.0 O: 28.8	C: 77.8 O: 22.2	60.0	8.75
CLM (PET)	66–81 [52]	C: 70.5 O: 29.2	C: 77.5 O: 22.5	28.0	13.0 ^f^	C: 71.7 O: 28.1	C: 76.7 O: 23.3	67.1	22.0 ^f^
FAV (PET)	66–81 [52]	C: 55.8 O: 40.9 Si: 2.97	C: 66.1 O: 32.2 Si: 1.59	11.7	0.65	C: 54.3 O: 42.1 Si: 3.09	C: 76.4 O: 23.6	72.6	0.15
JEM (/SiO_2_/ PC)	82–84 [53]	C: 53.9 Si: 43.8	C: 62.9 O: 29.3 Si: 7.87	18.2	2.08	O: 53.2 Si: 44.4	C: 62.8 O: 29.7 Si: 7.44	19.7	1.43
OPT (iPP)	99–102 [54]	C: 99.9	C: 98.8 O: 1.24	53.2	2.71	C: 98.7 O: 1.20	C: 100	53.6	3.88
RAN (PVC)	76–87 [55]	Cl: 99.7	C: 79.7 Cl: 20.3	76.6	2.61	Cl: 99.7	C: 79.6 Cl: 20.4	78.8	2.23
SEU (SR)	112 [56]	Si: 77.5 O: 22.0	C: 56.1 O: 24.0 Si: 19.9	87.2	0.07	Si: 77.7 O: 21.8 Al: 0.48	C: 57.7 O: 22.5 Si:19.8	88.2	4.80
SNC (PET)	66–81 [52]	C: 71.4 O: 28.3	C: 75.5 O: 24.5	44.7	2.33	C: 77.2 O: 22.7	C: 75.9 O: 24.1	51.2	3.33
STK (PET)	66–81 [52]	C: 68.1 O: 31.6	C: 75.8 O: 24.2	10.1	0.40	C: 70.4 O: 29.3	C: 72.0 O: 25.3 N: 2.72	74.9	4.08

a: contact angles for each main transparent material obtained from literature; b: determined by XRS/EDX; c: determined by SEM/EDX; d: determined by water contact angle goniometry; e: Std. Dev.: standard deviation; f: see Figure 7.

**Table 8:** Quantitative optical properties of transparent portions in face masks.

Mask ID	UV-Vis Reflectance (%)	UV-Vis Haze (%)
BEC	3.0	1.82
BES	4.3	0.71
CLM	4.0	2.06
FAV	2.7	0.51
JEM	3.1	0.97
OPT	5.4	8.04
RAN	2.3	2.46
SEU	4.6	2.44
SNC	5.4	2.15
STK	3.3	0.73

**Table 9:** Thermal properties and degrees of crystallinities of polymers in transparent portion of face masks.

Mask	Density (g/mL)	Crystallinity from Density	Glass Transition Temp.(°C)	Melting Temp. (°C)	Degree of Crystallinity (°C)	Decom- Position Onset Temp. (°C)	Decom- Position Temp. (°C)	TGA Residue % at 700 °C
BEC	1.3332	-	67.9	-	-	391	408	5.8
BES	1.3880	44.17	73.3	254.5	55.70	422	451	11.3
CLM	1.3900	45.83	74.1	254.6	50.67	454	491	11.2
FAV	1.3932	48.50	75.0	252.9	55.88	376	419	13.9
JEM	1.2024	-	138.0	-	-	451	500	23.8
OPT	0.9180	76.92	−14.9	162.6	93.53	352	425	0
RAN	1.3700	-	77.0	-	-	275/411	285/416	7.0
SEU	1.2388	-	Not observed	-	-	540	588	34.6
SNC	1.3916	47.17	72.9	250.2	54.55	408	452	5.3
STK	1.3900	45.83	66.2	252.6	54.22	408	441	8.8

**Table 10:** Molecular weight properties of the polymers in the transparent portions of the face masks.

Mask ID	M_w_ (g/mol)	M_n_ (g/mol)	PDI
BEC	58,500	29,300	1.99
BES	45,400	25,000	1.82
CLM	44,900	24,900	1.80
FAV	n.d.	n.d.	n.d.
JEM	19,667	13,400	1.64
OPT	338,000	54,100	6.26
RAN	188,000	141,000	1.33
SEU	n.a.	n.a.	n.a.
SNC	43,100	26,200	1.64
STK	45,000	26,500	1.70

n.d.: not determined, n.a.: not available.

**Table 11:** Mechanical and viscoelastic properties of transparent portions of face masks.

Mask	Tensile Strength ^a^ (MPa)	Std. Dev.	Strain (%)	Std. Dev.	Modulus (MPa)	Std. Dev
BEC	51.7	6.23	371.3	30.15	1390	121.0
BES	189.0	13.2	172.7	39.26	4060 ^b^	288.9 ^b^
CLM	179.0	8.56	100.6	21.54	3570	168.0
FAV	166.0	4.17	200.0	21.85	3450	116.0
JEM	68.2	4.09	60.6	13.54	2230	91.1
OPT	28.5	1.77	636.9	107.07	1000	44.3
RAN	57.5	1.34	139.3	47.94	2040	36.9
SEU	0.248	0.07	186.6	41.03	0.391	0.1
SNC	122.0	14.33	99.0	32.69	3190	35.4
STK	148.0	19.67	170.0 ^b^	24.74 ^b^	3280	51.4

a: highest tensile strength at yield or break; Std. Dev.: standard deviation (referring to value on left); b: eliminating one outlier; sample shape: 10 mm, length 30 mm, cut in vertical direction for each face mask (except SNC, due to insufficient length; for JEM, due to its greater thickness and dumbbell shape, ASTM D638 type V was used); strain rate: 50 mm/min (except BEC and RAN, with 15 mm/min to avoid premature tearing); number of specimens per mask: 5 (except for JEM with 4, due to limited availability).

**Table 12:** Example of assessment of a non-quantitative Boolean property.

Mask ID	Protection-Specific Filter	Rank	Weight	Points per Property
BEC	No	0	1.0	0
BES	No	0	1.0	0
CLM	No	0	1.0	0
FAV	No	0	1.0	0
JEM	Yes	10	1.0	10.0
OPT	No	0	1.0	0
RAN	No	0	1.0	0
SEU	Yes	10	1.0	10.0
SNC	No	0	1.0	0
STK	No	0	1.0	0

**Table 13:** Example of assessment of a quantitative property.

Mask Type	Sustainability Thickness	Rank (1) Using RANK Function, for Descending Order	Rank (2a) from Normalized Value, for Ascending Order	Rank (2b) = 10 − Rank (2a), for Descending Order	Weight	Points per Property
**BEC**	**0.441**	3	2.4	**7.6**	**1.0**	**7.6**
**BES**	**0.165**	9	0.3	**9.7**	**1.0**	**9.7**
**CLM**	**0.190**	6	0.5	**9.5**	**1.0**	**9.5**
**FAV**	**0.195**	5	0.5	**9.5**	**1.0**	**9.5**
**JEM**	**1.456**	1	10.0	**0.0**	**1.0**	**0**
**OPT**	**0.123**	10	0.0	**10.0**	**1.0**	**10**
**RAN**	**0.288**	4	1.2	**8.8**	**1.0**	**8.8**
**SEU**	**0.600**	2	3.6	**6.4**	**1.0**	**6.4**
**SNC**	**0.168**	8	0.3	**9.7**	**1.0**	**9.7**
**STK**	**0.190**	6	0.5	**9.5**	**1.0**	**9.5**

Text in bold print is applied for the example of sustainability—thickness; text in regular print is only used for explanations.

**Table 14:** Overall assessment of transparent face masks.

Mask ID	Protection	Visibility	Comfort	Sustainability	Total
	(pts)	(pts)	(pts)	(pts)	(pts)
BEC	28.4	39.9	35.7	37.4	141.1
BES	31.1	53.9	25.3	41.4	152.7
CLM	18.6	47.4	29.3	41.8	137.1
FAV	35.7	60.4	32.3	36.4	164.8
JEM	40.8	60.7	23.8	14.3	139.6
OPT	34.9	32.6	56.0	42.0	165.5
RAN	40.2	49.7	34.1	42.4	166.4
SEU	52.0	41.4	40.3	33.7	167.4
SNC	32.1	38.4	29.3	39.7	139.5
STK	33.9	61.4	31.7	42.3	169.3

Highest value is best.

## Data Availability

The original contributions presented in this study are included in the article/Supplementary Materials. Further inquiries can be directed to the corresponding author.

## Supplementary Materials

The following supporting information can be downloaded at: https://www.mdpi.com/article/10.3390/polym17070937/s1. More detailed information can be found in the Supplementary Materials, available in a separate file. The Supplementary Materials contain the following: (1) Figure of side view of each face mask, (2) Figures of FTIR spectra of the transparent portion distinguishing inside and outside, the breathable portion, any filter, and any ear loops, etc., (3) Figures of selected SEM images of breathable portions, (4) Figures of XRF/EDX diagrams for each mask, (5) Figures of SEM/EDX diagrams for each mask, (6) Tables of contact angle data of (the transparent portion of) each mask, (7) Figures of UV-Vis Reflectance graphs of (the transparent portion of) each mask, (8) Figures of UV-Vis Transmittance graphs of (the transparent portion of) each mask, (9) Tables of the Assessment of Protection, (10) Tables of the Assessment of Visibility, (11) Tables of the Assessment of Comfort, and (12) Tables of the Assessment of Sustainability.
