# Supplementary material for: Polymers Used in Transparent Face Masks—Characterization, Assessment, and Recommendations for Improvements Including Their Sustainability
Source: Polymers (Basel). 2025 Mar 30;17(7):937. doi: 10.3390/polym17070937 (PMC11990979; doi:10.3390/polym17070937)
Supplement: Supplementary file 1 [file polymers-17-00937-s001.zip › polymers-3537115-supplementary.pdf]

SUPPLEMENTARY MATERIALS

Polymers used in Transparent Face Masks – Characterization, Assessment and Recommendations for Improvements including their Sustainability

Katie E. Miller[1,2], Ann-Carolin Jahn[1,2], Brian M. Strohm[1], Shao M. Demyttenaere[1], Paul J. Nikolai[1], Byron Behm[2], Mariam Paracha[2], Massoud J. Miri*[1]

1: School of Chemistry and Materials Science, Rochester Institute of Technology, Rochester, NY 14623

2: National Technical Institute for the Deaf, Rochester Institute of Technology, Rochester, NY

Table of Contents

Fig. S-1. Photos of Facemasks – Side view ……………………….……………….………...… S-7

Fig. S-2.1.1. FTIR spectrum of BEC – Transparent portion, inside…………...…….…….... S-8

Fig. S-2.1.2. FTIR spectrum of BEC – Transparent portion, outside……………….…......... S-9

Fig. S-2.1.3. FTIR spectrum of BEC – Breathable portion ..……………………………… S-10

Fig. S-2.1.4. FTIR spectrum of BEC – Gel strip for chin ………………………………… S-11

Fig. S-2.1.5. FTIR spectrum of BEC – Coating on nose bridge wire …………………… S-12

Fig. S-2.1.6. FTIR spectrum of BEC – Ear loop……………...……………………………S-13

Fig. S-2.2.1. FTIR spectrum of BES – Transparent portion, inside….……………………..S-14

Fig. S-2.2.2. FTIR spectrum of BES – Transparent portion, outside………………………S-15

Fig. S-2.2.3. FTIR spectrum of BES – Breathable portion ………………………...…… S-16

Fig. S-2.2.4. FTIR spectrum of BES – Ear loop ………………………………..…..……S-17

Fig. S-2.3.1. FTIR spectrum of CLM – Transparent portion, inside……………….……. S-18

Fig. S-2.3.2. FTIR spectrum of CLM – Transparent portion, outside…………………… S-19

Fig. S-2.3.3. FTIR spectrum of CLM – Breathable/Structural portion …………………….S-20

Fig. S-2.3.4. FTIR spectrum of CLM – Nose bridge………..………………………… S-21

Fig. S-2.3.5. FTIR spectrum of CLM – Ear loop …. ……. …...…………………………S-22

Fig. S-2.4.1. FTIR spectrum of FAV – Transparent portion, inside ……………….……S-23

Fig. S-2.4.2. FTIR spectrum of FAV – Transparent portion, outside …..……….….…… S-24

Fig. S-2.4.3. FTIR spectrum of FAV – Breathable portion…..…………………………… S-25

Fig. S-2.4.4. FTIR spectrum of FAV – Nose bridge………...……………………………S-26

Fig. S-2.4.5. FTIR spectrum of FAV – Ear loop ………………………………………...S-27

Fig. S-2.5.1. FTIR spectrum of JEM – Transparent portion (heated to 150 ^{o}C), inside….... S-28

Fig. S-2.5.2. FTIR spectrum of JEM – Transparent portion, (heated to 150 ^{o}C), outside... S-29

Fig. S-2.5.3. FTIR spectrum of JEM – Transparent portion, fraction extracted with Chloroform ……………………………………………………………………………S-30

Fig. S-2.5.4. FTIR spectrum of JEM – Structural portion (Filter cap)……………….…… S-31

Fig. S-2.5.5. FTIR spectrum of JEM – Filter ……………………………………………S-32

Fig. S-2.5.6. FTIR spectrum of JEM – Seal……………………..……………………..S-33

Fig. S-2.5.7. FTIR spectrum of JEM – Ear loop……………………………….……..…S-34

Fig. S-2.6.1. FTIR spectrum of OPT – Transparent portion, inside……………………….. S-35

Fig. S-2.6.2. FTIR spectrum of OPT – Transparent portion, outside……………………… S-36

Fig. S-2.6.3. FTIR spectrum of OPT – Breathable portion ………………………...……S-37

Fig. S-2.6.3. FTIR spectrum of OPT – Nose bridge……. ………………………...…… S-38

Fig. S-2.6.4. FTIR spectrum of OPT – Ear loop …….…………………….……………S-39

Fig. S-2.7.1. FTIR spectrum of RAN – Transparent portion, inside……………….……… S-40

Fig. S-2.7.2. FTIR spectrum of RAN – Transparent portion, outside……………………... S-41

Fig. S-2.7.3. FTIR spectrum of RAN – Breathable portion …………………………..…...S-42

Fig. S-2.7.4. FTIR spectrum of RAN – Coating on nose bridge wire ………………...…... S-43

Fig. S-2.7.4. FTIR spectrum of RAN – Ear loop …………….………………….……... S-44

Fig. S-2.8.1. FTIR spectrum of SEU – Transparent portion, inside ………………………. S-45

Fig. S-2.8.2. FTIR spectrum of SEU – Transparent portion, outside ……………………... S-46

Fig. S-2.8.3. FTIR spectrum of SEU – Structural portion ……………………….……... S-47

Fig. S-2.8.4. FTIR spectrum of SEU – Filter ……………………………………………S-48

Fig. S-2.8.5. FTIR spectrum of SEU – Adhesive …….……………………………….... S-49

Fig. S-2.9.1. FTIR spectrum of SNC – Transparent portion, inside……………….………S-50

Fig. S-2.9.2. FTIR spectrum of SNC – Transparent portion, outside……………….…………...S-51

Fig. S-2.9.3. FTIR spectrum of SNC – Breathable portion ……………………......…… S-52

Fig. S-2.9.4. FTIR spectrum of SNC – Ear loop ………………………….………….... S-53

Fig. S-2.10.1. FTIR spectrum of STK – Transparent portion, inside ………………………S-54

Fig. S-2.10.2. FTIR spectrum of STK – Transparent portion, outside ...…………………...S-55

Fig. S-2.10.3. FTIR spectrum STK – Breathable portion …………….……………………S-56

Fig. S-2.10.4. FTIR spectrum STK – Coating on nose bridge wire ….…………………… S-57

Fig. S-2.10.5. FTIR spectrum of STK – Ear loop …………….…………...………… S-58

Fig. S-2.11. FTIR spectrum of PHG (main, breathable portion of mask)…………………. S-59

Fig. S-3. SEM images of breathable portions of examples of masks …..………..……….. S-60

Fig. S-4.1.1. XRF/EDX of BEC - Transparent portion, inside …………………………… S-61

Fig. S-4.1.2. XRF/EDX of BEC - Transparent portion, outside …………………………...S-62

Fig. S-4.1.1. XRF/EDX of BES - Transparent portion, inside ……………………………..S-63

Fig. S-4.1.2. XRF/EDX of BES - Transparent portion, outside ………………………….. .S-64

Fig. S-4.1.1. XRF/EDX of CLM - Transparent portion, inside ……………………………S-65

Fig. S-4.1.2. XRF/EDX of CLM - Transparent portion, outside …………………………..S-66

Fig. S-4.1.1. XRF/EDX of FAV - Transparent portion, inside …………………………….S-67

Fig. S-4.1.2. XRF/EDX of FAV - Transparent portion, outside ………………………….. S-68

Fig. S-4.1.1. XRF/EDX of JEM - Transparent portion, inside …………………………….S-69

Fig. S-4.1.2. XRF/EDX of JEM- Transparent portion, outside ……………………………S-70

Fig. S-4.1.1. XRF/EDX of OPT - Transparent portion, inside …………………………….S-71

Fig. S-4.1.2. XRF/EDX of OPT - Transparent portion, outside …………………………...S-72

Fig. S-4.1.1. XRF/EDX of RAN - Transparent portion, inside …………………………....S-73

Fig. S-4.1.2. XRF/EDX of RAN - Transparent portion, outside …………………………..S-74

Fig. S-4.1.1. XRF/EDX of SEU - Transparent portion, inside …………………………….S-75

Fig. S-4.1.2. XRF/EDX of SEU - Transparent portion, outside …………………………...S-76

Fig. S-4.1.1. XRF/EDX of SNC - Transparent portion, inside …………………………….S-77

Fig. S-4.1.2. XRF/EDX of SNC - Transparent portion, outside ………………………… S-78

Fig. S-4.1.1. XRF/EDX of STK - Transparent portion, inside ……………………………...S-79

Fig. S-4.1.2. XRF/EDX of STK - Transparent portion, outside …………………………... S-80

Fig. S-5.1.1. SEM/EDX diagram for BEC - Transparent portion, inside …………………...S-81

Fig. S-5.1.2. SEM/EDX diagram for BEC - Transparent portion, outside ………………….S-82

Fig. S-5.1.1. SEM/EDX diagram for BES - Transparent portion, inside …………………...S-83

Fig. S-5.1.2. SEM/EDX diagram for BES - Transparent portion, outside ………………….S-84

Fig. S-5.1.1. SEM/EDX diagram for CLM - Transparent portion, inside …………………..S-85

Fig. S-5.1.2. SEM/EDX diagram for CLM- Transparent portion, outside ………………….S-86

Fig. S-5.1.1. SEM/EDX diagram for FAV - Transparent portion, inside …………………...S-87

Fig. S-5.1.2. SEM/EDX diagram for FAV - Transparent portion, outside ………………....S-88

Fig. S-5.1.1. SEM/EDX diagram for JEM - Transparent portion, inside …………………...S-89

Fig. S-5.1.2. SEM/EDX diagram for JEM - Transparent portion, outside ………………….S-90

Fig. S-5.1.1. SEM/EDX diagram for OPT - Transparent portion, inside …………………...S-91

Fig. S-5.1.2. SEM/EDX diagram for OPT - Transparent portion, outside ………………….S-92

Fig. S-5.1.1. SEM/EDX diagram for RAN - Transparent portion, inside ………………… .S-93

Fig. S-5.1.2. SEM/EDX diagram for RAN - Transparent portion, outside …………………S-94

Fig. S-5.1.1. SEM/EDX diagram for SEU - Transparent portion, inside …………………...S-95

Fig. S-5.1.2. SEM/EDX diagram for SEU - Transparent portion, outside ………………….S-96

Fig. S-5.1.1. SEM/EDX diagram for SNC - Transparent portion, inside …………………...S-97

Fig. S-5.1.2. SEM/EDX diagram for SNC - Transparent portion, outside ………………….S-98

Fig. S-5.1.1. SEM/EDX diagram for STK - Transparent portion, inside …………………...S-99

Fig. S-5.1.2. SEM/EDX diagram for STK - Transparent portion, outside …………………S-100

Fig. S-6. Photo of a contact angle measurement of STK - Transparent portion, inside …... S-101

Table S-1.1. Contact Angle Data – BEC and BES (CLM not included, see main article)…S-102

Table S-1.2. Contact Angle Data – FAV ……...…………………………………………... S-103

Table S-1.3. Contact Angle Data – JEM and OPT …………...…………………………… S-104

Table S-1.4. Contact Angle Data – RAN and SEU ……………...…………………...…… S-105

Table S-1.5. Contact Angle Data – SNC and STK ………...……………………………… S-106

Fig. S-7.1. UV-Vis Reflectance Graph of BEC……….……………………….....…… S-107
Fig. S-7.2. UV-Vis Reflectance Graph of BES…………..……..………………………… S-107
Fig. S-7.3. UV-Vis Reflectance Graph of CLM……...………………………………… S-108
Fig. S-7.4. UV-Vis Reflectance Graph of FAV ……….………………………………… S-108
Fig. S-7.5. UV-Vis Reflectance Graph of JEM …………..…...………………………… S-109
Fig. S-7.6. UV-Vis Reflectance Graph of OPT ………..……...………………………… S-109
Fig. S-7.7. UV-Vis Reflectance Graph of RAN ….….…...…………………….....….... S-110
Fig. S-7.8. UV-Vis Reflectance Graph of SEU …………………………….……………S-110
Fig. S-7.9. UV-Vis Reflectance Graph of SNC ……………………………………….S-111
Fig. S-7.10. UV-Vis Reflectance Graph of STK ……………………………………...S-111
Fig. S-8.1. UV-Vis Transmittance Graph of BEC ……………………………………….S-112
Fig. S-8.2. UV-Vis Transmittance Graph of BES ……………………………………....S-112
Fig. S-8.3. UV-Vis Transmittance Graph of CLM …………………………………….…...S-113
Fig. S-8.4. UV-Vis Transmittance Graph of FAV.……………………………………….S-113
Fig. S-8.5. UV-Vis Transmittance Graph of JEM ……………………………………… S-114
Fig. S-8.6. UV-Vis Transmittance Graph of OPT ……………………………………….S-114
Fig. S-8.7. UV-Vis Transmittance Graph of RAN ………………………………………S-115
Fig. S-8.8. UV-Vis Transmittance Graph of SEU ……………………………………… S-115
Fig. S-8.9. UV-Vis Transmittance Graph of SNC ……………………………………… S-116
Fig. S-8.10. UV-Vis Transmittance Graph of STK ……………………………………….. S-116
Table S-2.1.1. Assessment of PROTECTION – Seal …...………………………………S-117
Table S-2.1.2. Assessment of PROTECTION – Major gaps ……………...……………….S-118
Table S-2.1.3. Assessment of PROTECTION – Available Sizes…………………………..S-119
Table S-2.1.4. Assessment of PROTECTION – Breathable area …………………………S-120
Table S-2.1.5. Assessment of PROTECTION – Breathable Material……...……………... S-121
Table S-2.1.6. Assessment of PROTECTION – Filter presence …………...……………... S-122
Table S-2.1.7. Assessment of PROTECTION – Nose bridge material……………………S-123
Table S-2.1.8. Assessment of PROTECTION – Types of approvals and Subtotal ……...... S-124

Table S-2.2.1. Assessment of VISIBILITY – Clear area around mouth (based on photos)..S-125

Table S-2.2.2. Assessment of VISIBILITY – Transparent area (cm^2) ……………………. S-126

Table S-2.2.3. Assessment of VISIBILITY – Apparent distortion (based on photos)......… S-127

Table S-2.2.4. Assessment of VISIBILITY – Apparent reflections (based on photos)…... S-128

Table S-2.2.5. Assessment of VISIBILITY – Reflectance by UV-Vis..……………………S-129

Table S-2.2.6. Assessment of VISIBILITY – Haze by UV-Vis……………………………S-130

Table S-2.2.7. Assessment of VISIBILITY – Crystallinity from Density and DSC………..S-131

Table S-2.2.8. Assessment of VISIBILITY – Contact Angle ………..……………………..S-132

Table S-2.2.9. Assessment of VISIBILITY – Inside (anti-fog) coating……….…………S-133

Table S-2.2.10. Assessment of VISIBILITY – Outside (anti-scratch) coating and Subtotal.S-134

Table S-2.3.1. Assessment of COMFORT – Flexibility from T_g ……………….……… S-135

Table S-2.3.2. Assessment of COMFORT – Flexibility from Tensile Stress………………S-136

Table S-2.3.3. Assessment of COMFORT – Flexibility from Elongation ……………...… S-137

Table S-2.3.4. Assessment of COMFORT – Flexibility from Modulus ………………… S-138

Table S-2.3.5. Assessment of COMFORT – Mass ………………...…………………… S-139

Table S-2.3.6. Assessment of COMFORT – Nose bridge material ………………………..S-140

Table S-2.3.7. Assessment of COMFORT – Ear loops ………………...……………..... S-141

Table S-2.3.8. Assessment of COMFORT – Ease of Assembly and Subtotal……...………S-142

Table S-2.4.1. Assessment of SUSTAINABILITY – Reusability ……………………....S-143

Table S-2.4.2. Assessment of SUSTAINABILITY – Recyclability……………….…....S-144

Table S-2.4.3. Assessment of SUSTAINABILITY – Renewability…………………….... S-145

Table S-2.4.4. Assessment of SUSTAINABILITY – Biodegradability…………………... S-146

Table S-2.4.5. Assessment of SUSTAINABILITY – Mass ……………….…………….... S-147

Table S-2.4.6. Assessment of SUSTAINABILITY – Thickness ………………….……... S-148

Table S-2.4.7. Assessment of SUSTAINABILITY – Price ……………………............. S-149

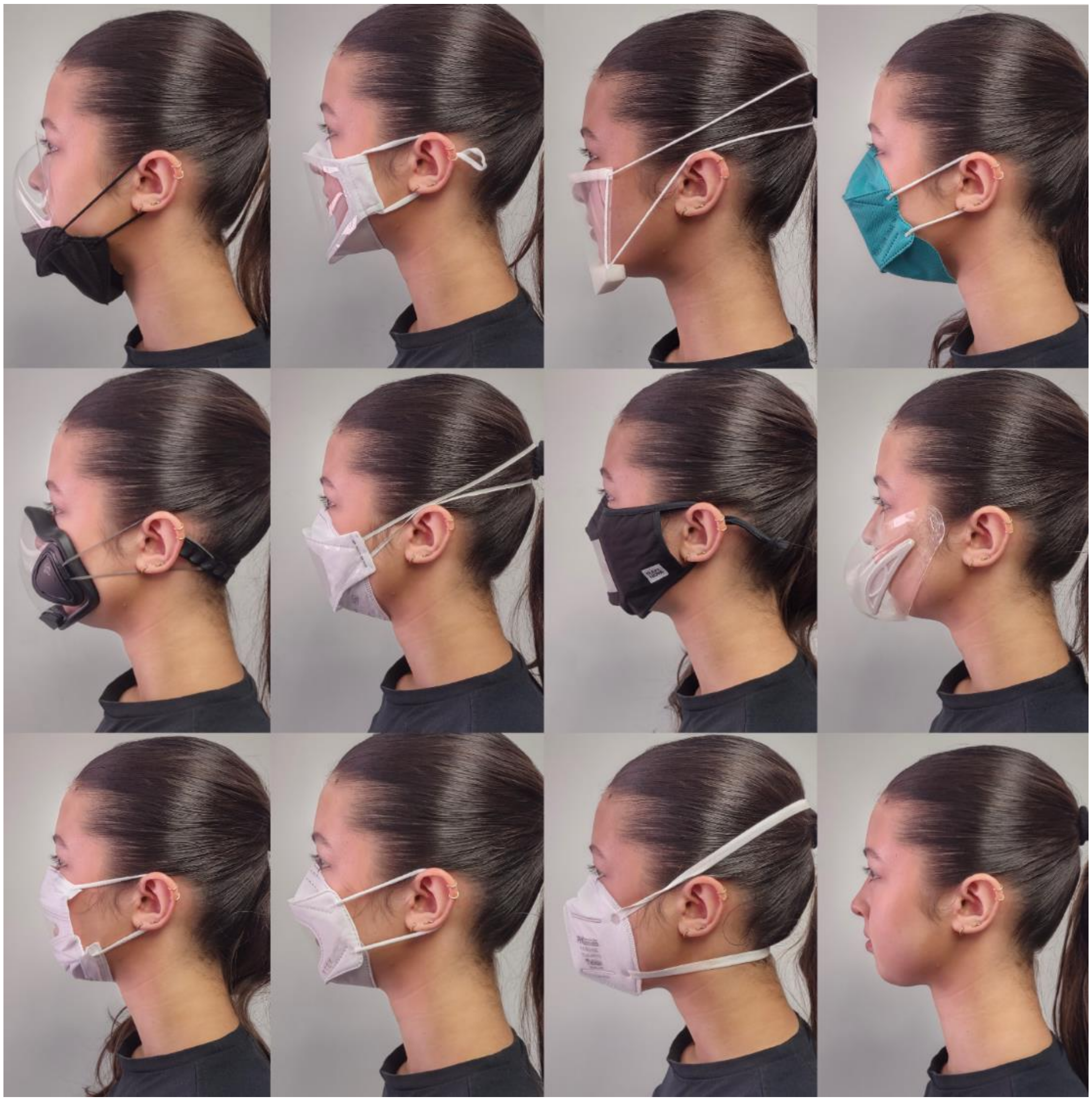

Fig. S-1. Photos of Facemasks – Side view

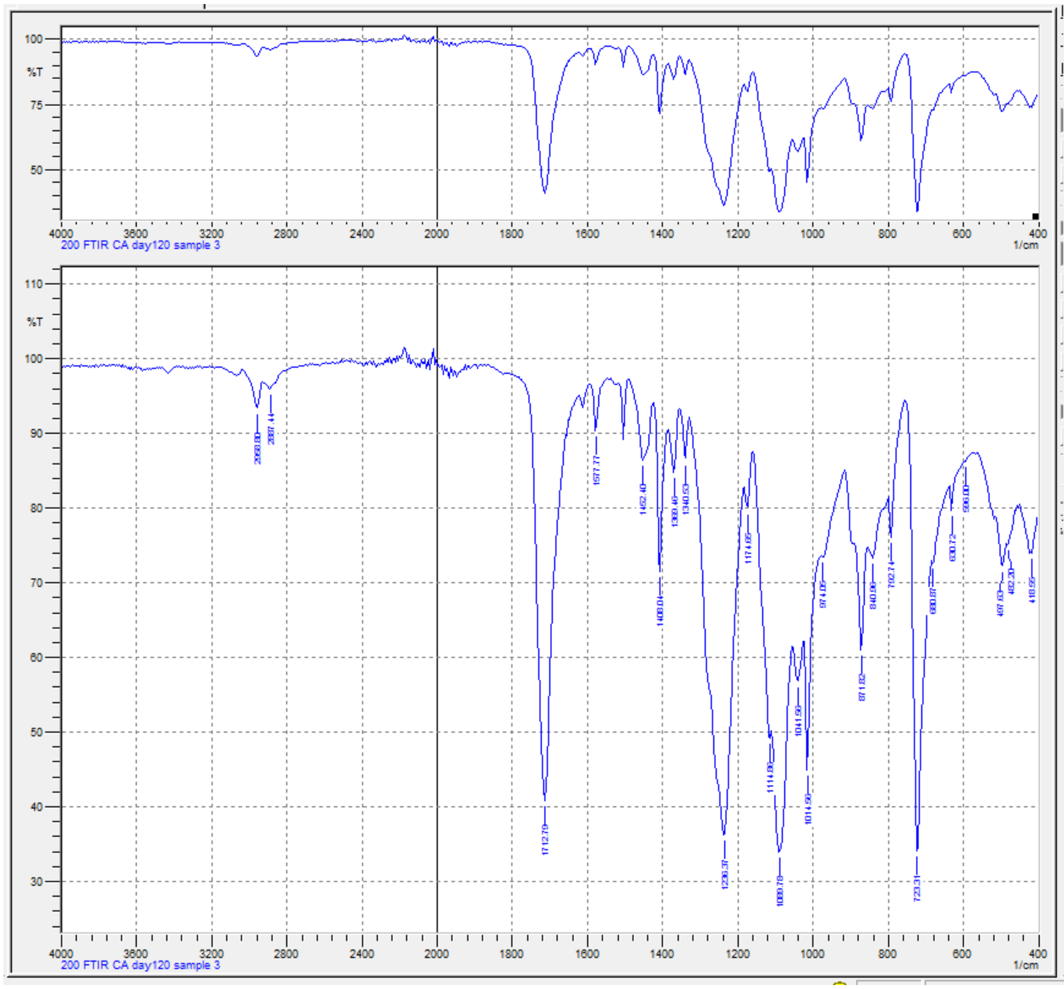

No.	Peak	Intensity
1	418.55	73.898
2	482.20	75.103
3	497.63	72.137
4	596.00	86.199
5	630.72	79.642
6	680.87	72.702
7	723.31	34.061
8	792.74	76.114
9	840.96	73.266
10	871.82	61.026
11	974.05	73.456
12	1014.56	44.996
13	1041.56	56.770
14	1089.78	33.925
15	1114.86	49.227
16	1174.65	79.934
17	1236.37	36.187
18	1340.53	86.415
19	1369.46	84.745
20	1408.04	71.524
21	1452.40	86.364
22	1577.77	90.345
23	1712.79	40.897
24	2887.44	95.925
25	2958.80	93.361

Calculate...

Fig. S-2.1.1. FTIR spectrum of BEC – Transparent portion, inside.

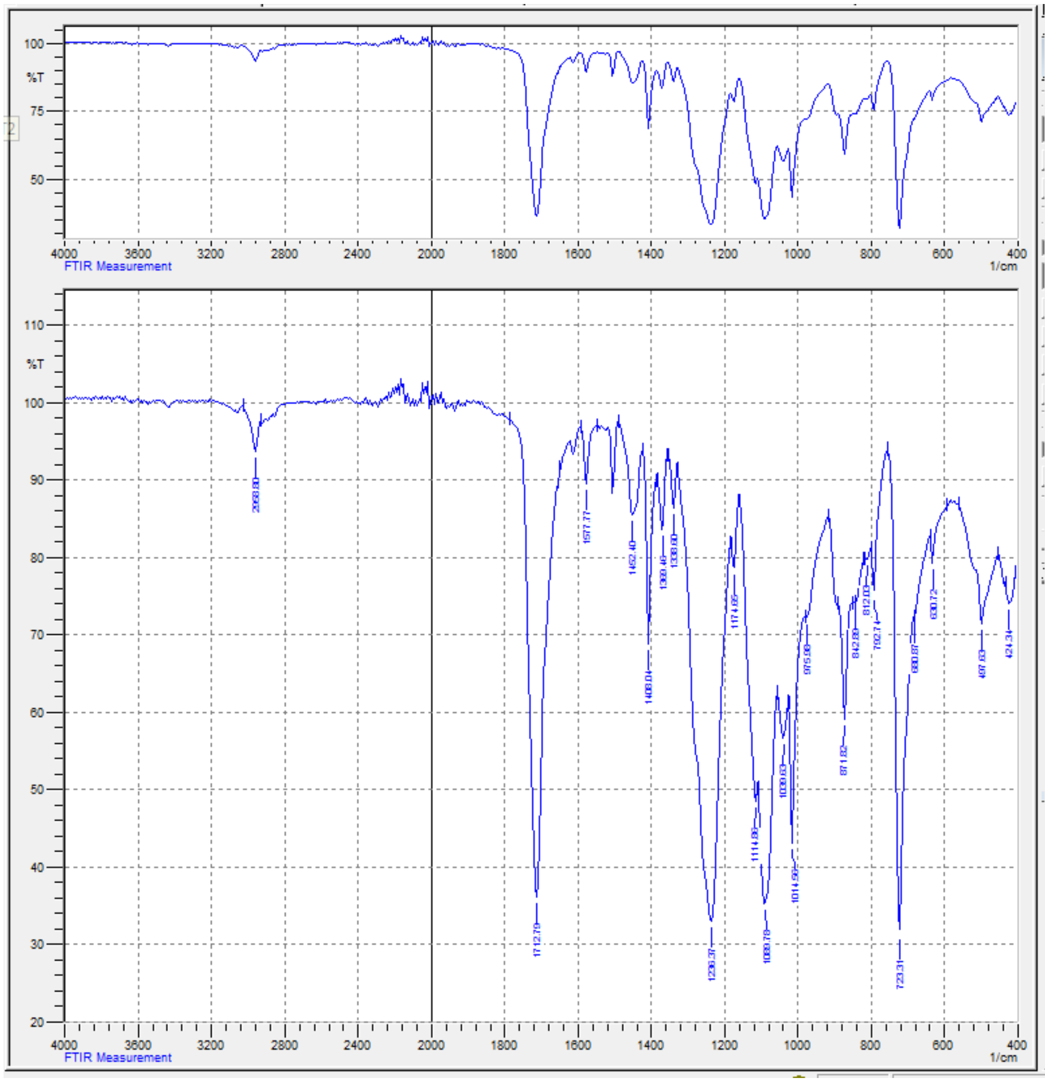

No.	Peak	Intensity
1	424.34	73.962
2	497.63	71.430
3	630.72	79.210
4	680.87	72.240
5	723.31	31.435
6	792.74	75.172
7	812.03	79.709
8	842.89	74.234
9	871.82	59.159
10	975.98	72.192
11	1014.56	43.124
12	1039.63	56.591
13	1089.78	35.327
14	1114.86	48.487
15	1174.65	78.509
16	1236.37	33.091
17	1338.60	86.336
18	1369.46	83.587
19	1408.04	68.893
20	1452.40	85.456
21	1577.77	89.541
22	1712.79	36.199
23	2958.80	93.645

Calculate...

Fig. S-2.1.2. FTIR spectrum of BEC – Transparent portion, outside.

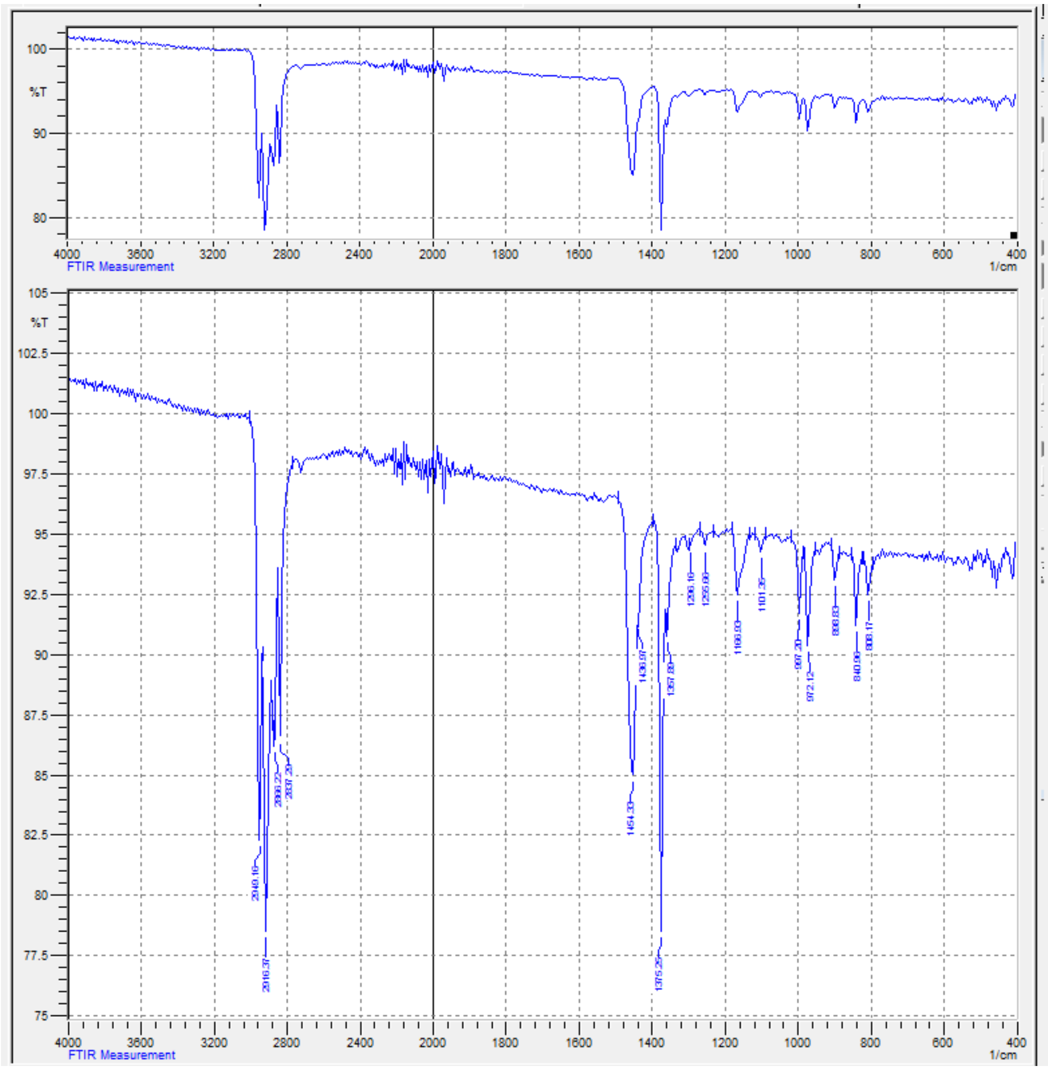

No.	Peak	Intensity
1	808.17	92.501
2	840.96	91.191
3	898.83	93.085
4	972.12	90.376
5	997.20	91.689
6	1101.35	94.286
7	1166.93	92.457
8	1255.66	94.480
9	1296.16	94.489
10	1357.89	90.772
11	1375.25	78.524
12	1436.97	91.336
13	1454.33	84.949
14	2837.29	86.505
15	2866.22	86.186
16	2916.37	78.520
17	2949.16	82.305

Calculate...

Fig. S-2.1.3. FTIR spectrum BEC – Breathable portion.

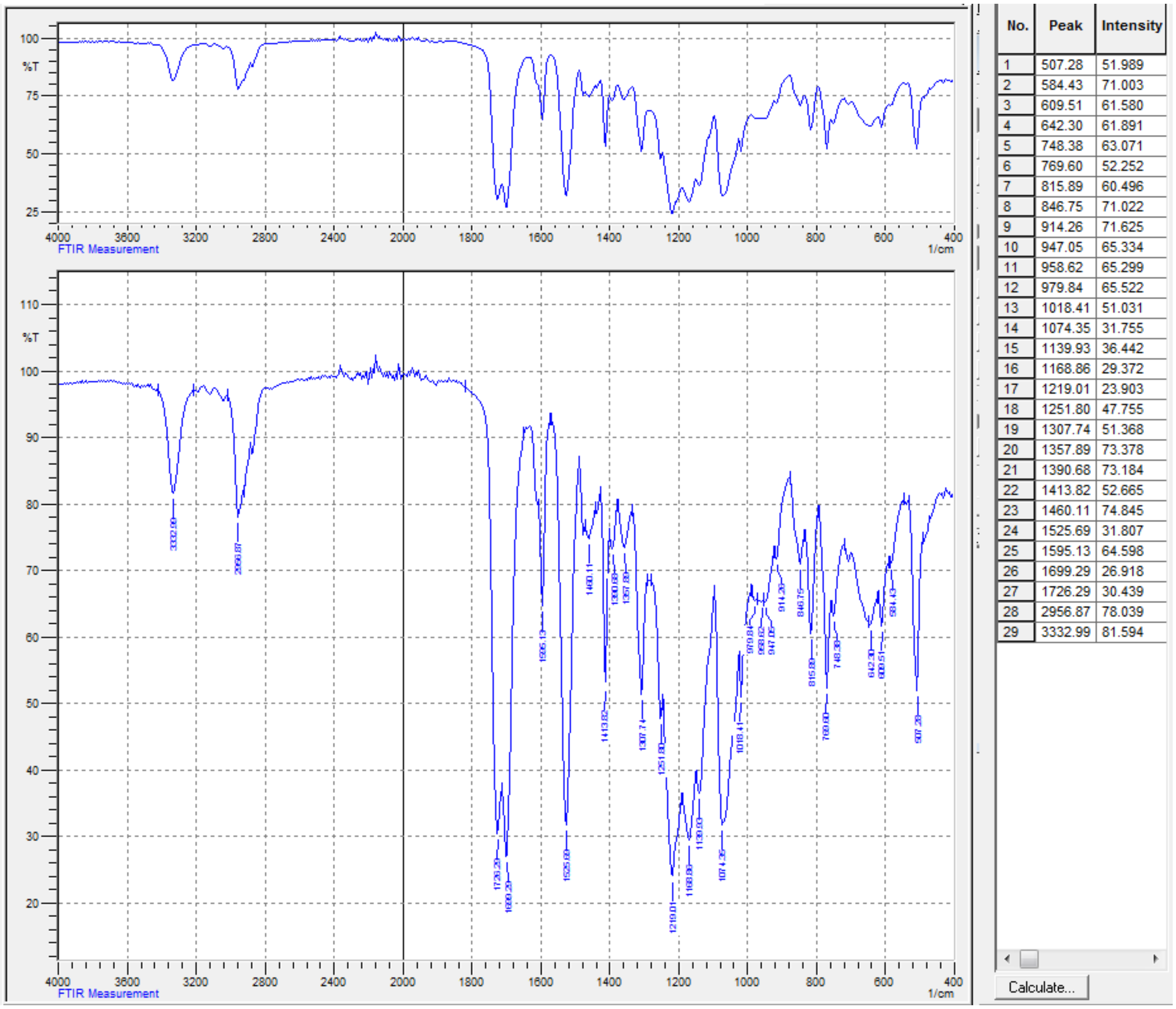

No.	Peak	Intensity
1	507.28	51.989
2	584.43	71.003
3	609.51	61.580
4	642.30	61.891
5	748.38	63.071
6	769.60	52.252
7	815.89	60.496
8	846.75	71.022
9	914.26	71.625
10	947.05	65.334
11	958.62	65.299
12	979.84	65.522
13	1018.41	51.031
14	1074.35	31.755
15	1139.93	36.442
16	1168.86	29.372
17	1219.01	23.903
18	1251.80	47.755
19	1307.74	51.368
20	1357.89	73.378
21	1390.68	73.184
22	1413.82	52.665
23	1460.11	74.845
24	1525.69	31.807
25	1595.13	64.598
26	1699.29	26.918
27	1726.29	30.439
28	2956.87	78.039
29	3332.99	81.594

Fig. S-2.1.4 FTIR spectrum of BEC – Gel Strip for Chin.

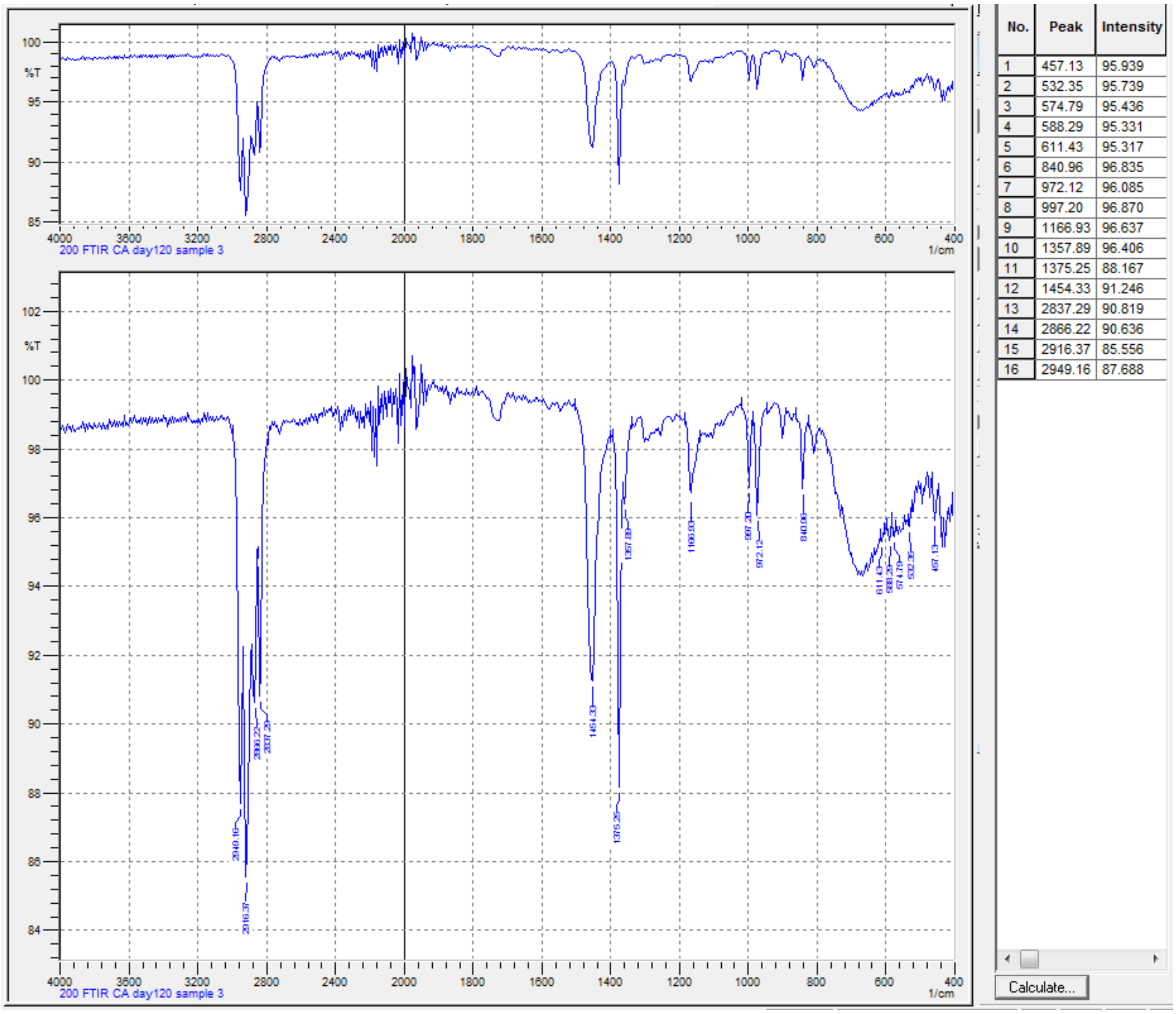

No.	Peak	Intensity
1	457.13	95.939
2	532.35	95.739
3	574.79	95.436
4	588.29	95.331
5	611.43	95.317
6	840.96	96.835
7	972.12	96.085
8	997.20	96.870
9	1166.93	96.637
10	1357.89	96.406
11	1375.25	88.167
12	1454.33	91.246
13	2837.29	90.819
14	2866.22	90.636
15	2916.37	85.556
16	2949.16	87.688

Fig. S-2.1.6. FTIR spectrum BEC – Coating on nose bridge wire.

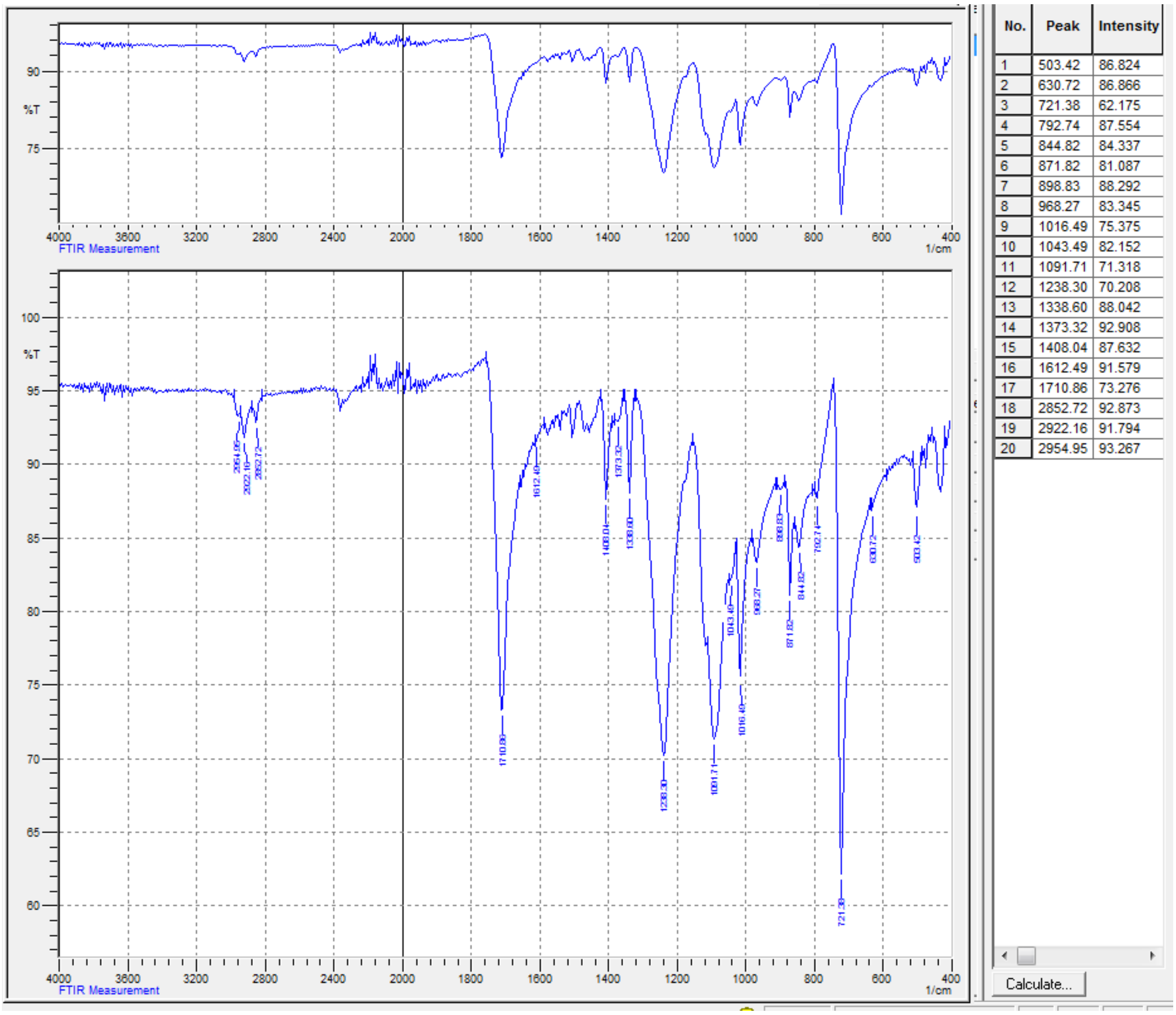

No.	Peak	Intensity
1	503.42	86.824
2	630.72	86.866
3	721.38	62.175
4	792.74	87.554
5	844.82	84.337
6	871.82	81.087
7	898.83	88.292
8	968.27	83.345
9	1016.49	75.375
10	1043.49	82.152
11	1091.71	71.318
12	1238.30	70.208
13	1338.60	88.042
14	1373.32	92.908
15	1408.04	87.632
16	1612.49	91.579
17	1710.86	73.276
18	2852.72	92.873
19	2922.16	91.794
20	2954.95	93.267

Calculate...

Fig. S-2.1.7. FTIR spectrum BEC – Ear loop.

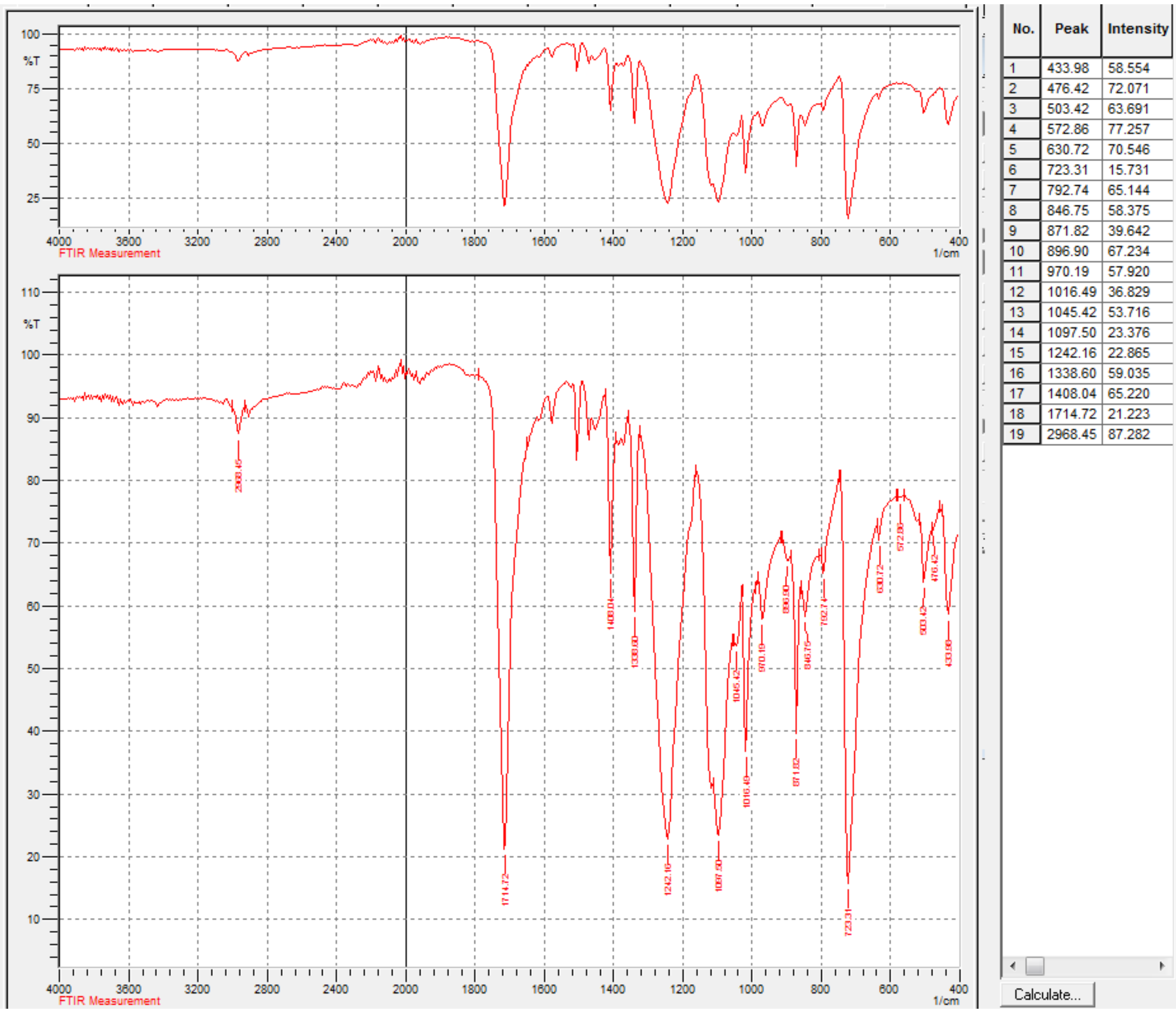

No.	Peak	Intensity
1	433.98	58.554
2	476.42	72.071
3	503.42	63.691
4	572.86	77.257
5	630.72	70.546
6	723.31	15.731
7	792.74	65.144
8	846.75	58.375
9	871.82	39.642
10	896.90	67.234
11	970.19	57.920
12	1016.49	36.829
13	1045.42	53.716
14	1097.50	23.376
15	1242.16	22.865
16	1338.60	59.035
17	1408.04	65.220
18	1714.72	21.223
19	2968.45	87.282

Calculate...

Fig. S-2.2.1. FTIR spectrum of BES – Transparent portion, inside.

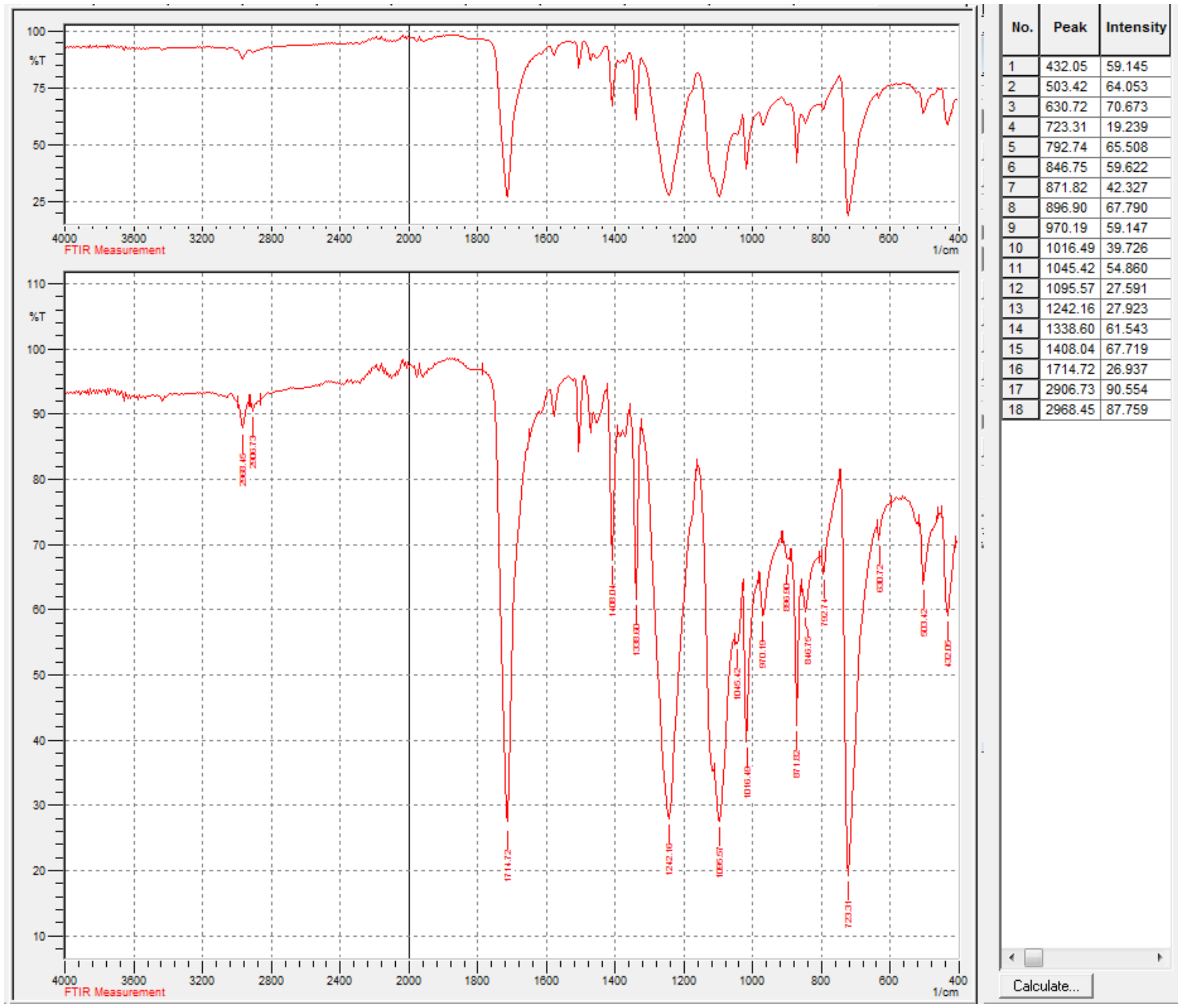

No.	Peak	Intensity
1	432.05	59.145
2	503.42	64.053
3	630.72	70.673
4	723.31	19.239
5	792.74	65.508
6	846.75	59.622
7	871.82	42.327
8	896.90	67.790
9	970.19	59.147
10	1016.49	39.726
11	1045.42	54.860
12	1095.57	27.591
13	1242.16	27.923
14	1338.60	61.543
15	1408.04	67.719
16	1714.72	26.937
17	2906.73	90.554
18	2968.45	87.759

Calculate...

Fig. S-2.2.2. FTIR spectrum of BES – Transparent portion, outside.

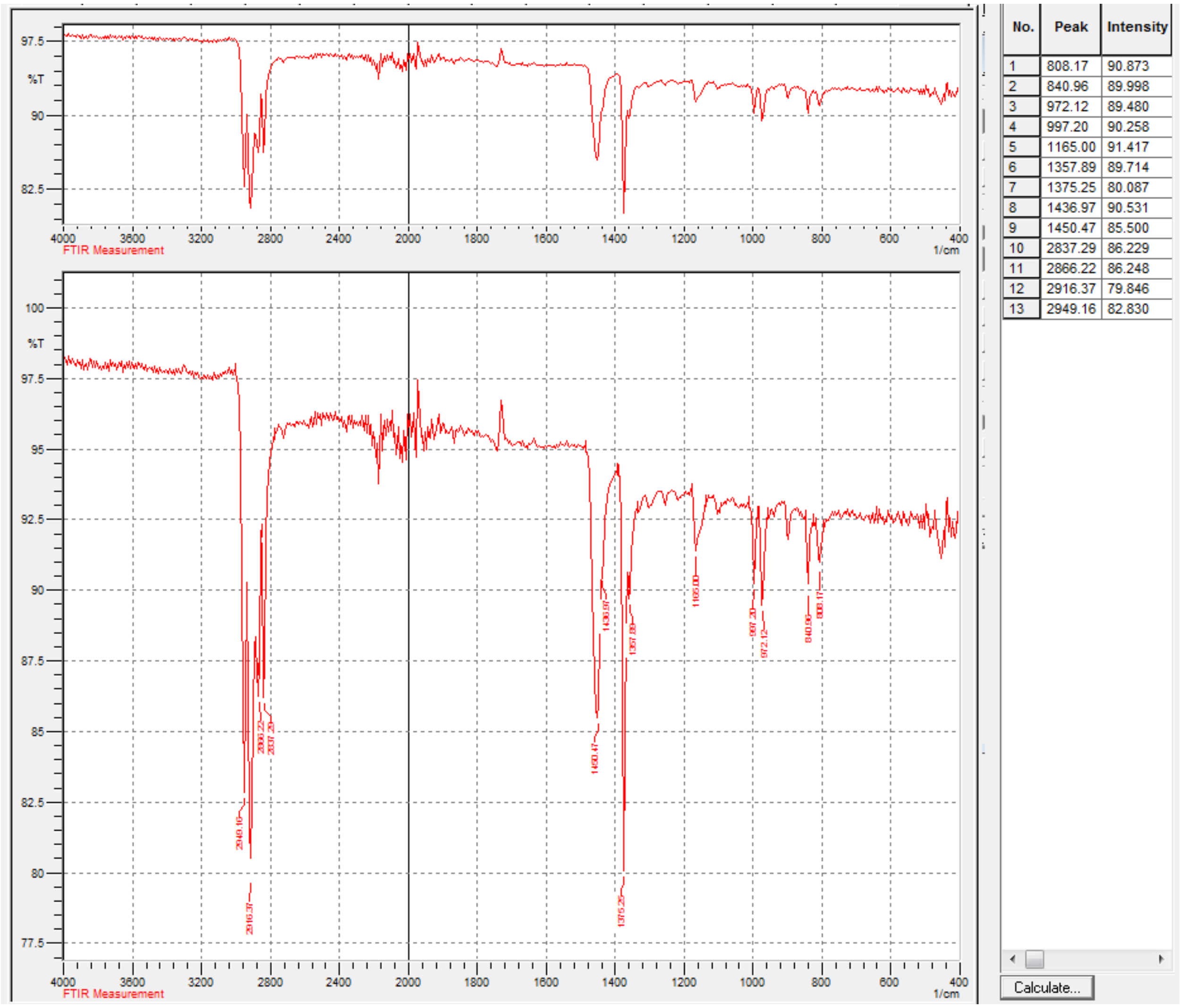

Fig. S-2.2.3. FTIR spectrum of BES – Breathable portion.

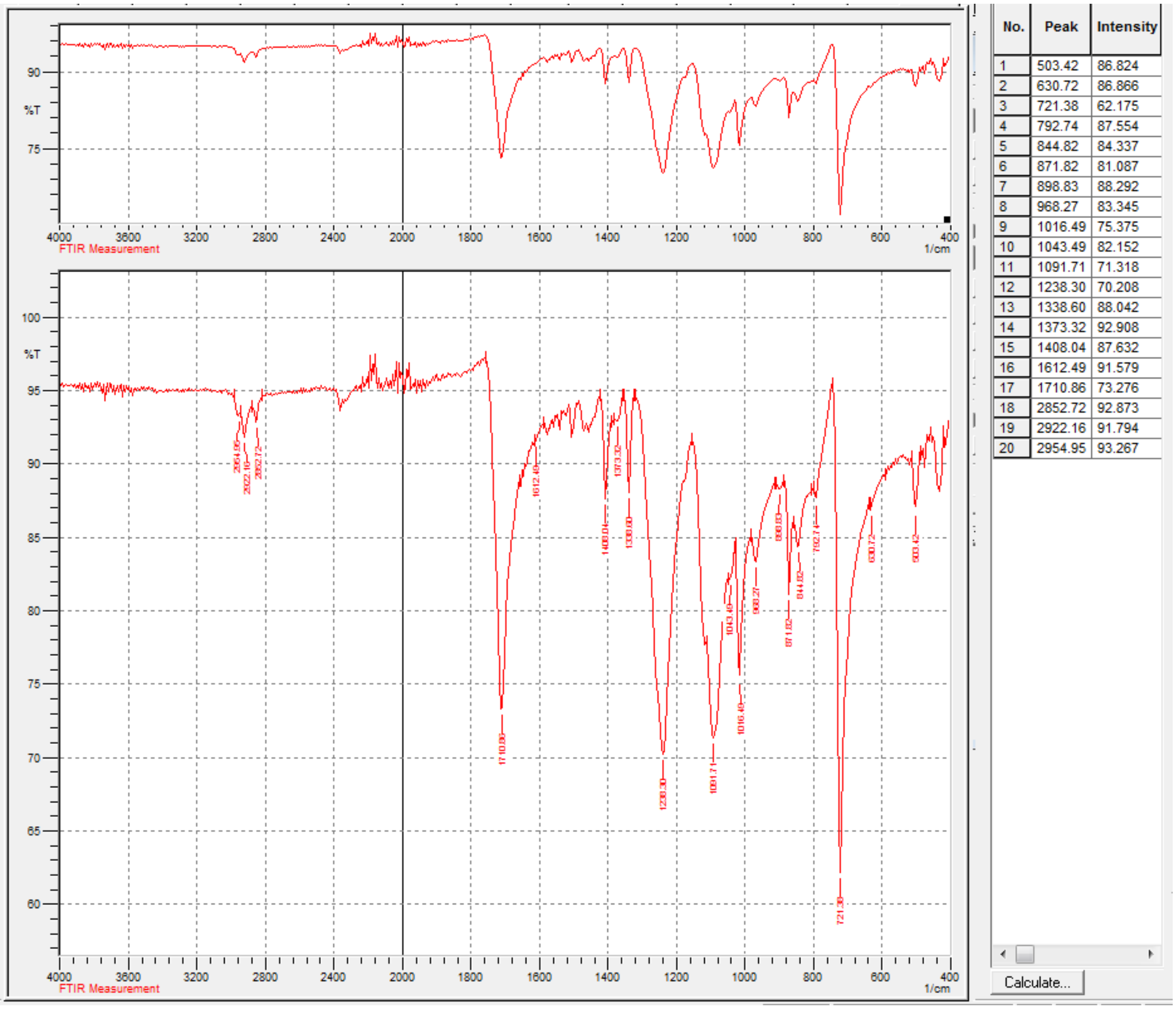

No.	Peak	Intensity
1	503.42	86.824
2	630.72	86.866
3	721.38	62.175
4	792.74	87.554
5	844.82	84.337
6	871.82	81.087
7	898.83	88.292
8	968.27	83.345
9	1016.49	75.375
10	1043.49	82.152
11	1091.71	71.318
12	1238.30	70.208
13	1338.60	88.042
14	1373.32	92.908
15	1408.04	87.632
16	1612.49	91.579
17	1710.86	73.276
18	2852.72	92.873
19	2922.16	91.794
20	2954.95	93.267

Fig. S-2.2.4. FTIR spectrum of BES – Ear loop.

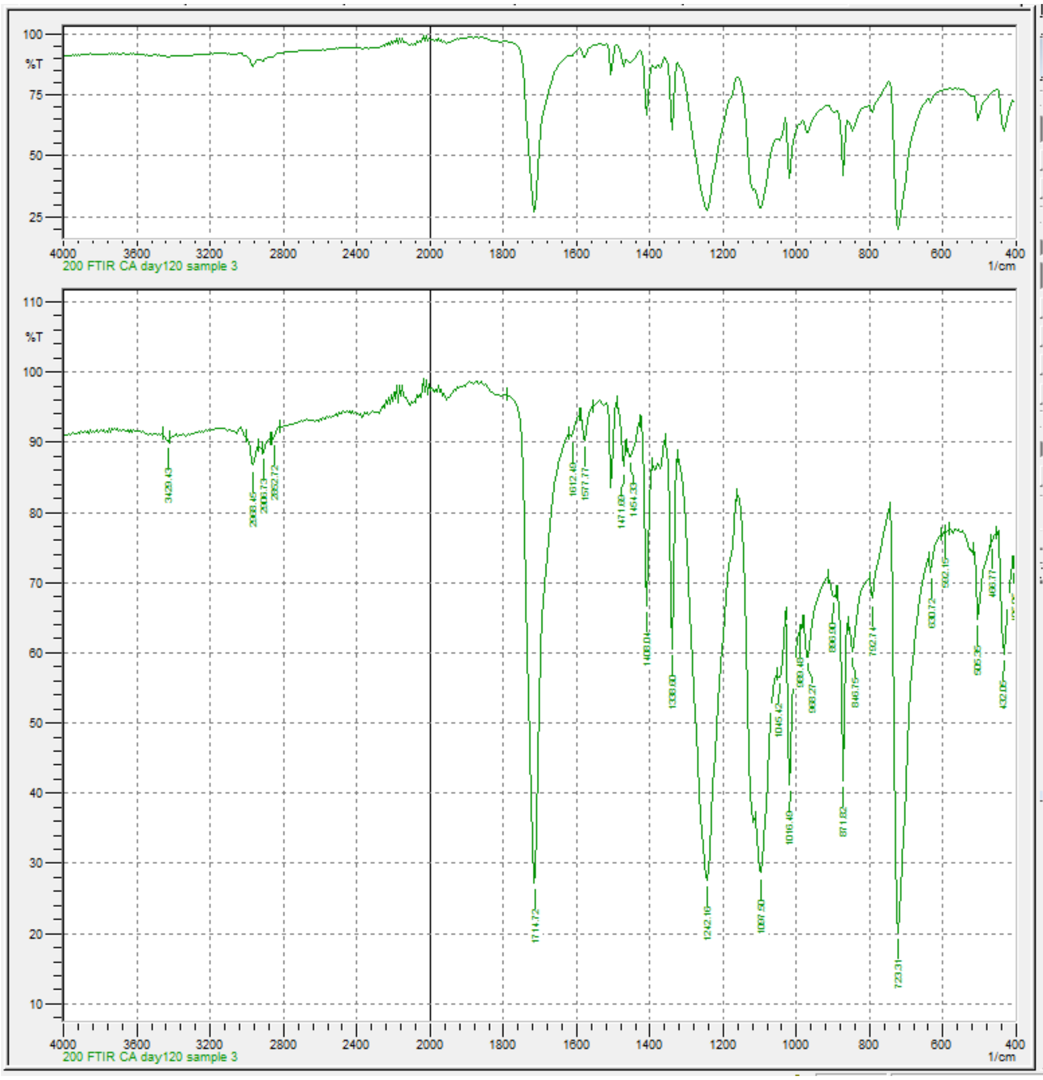

No.	Peak	Intensity
1	405.05	72.305
2	432.05	59.876
3	466.77	75.692
4	505.35	64.684
5	592.15	77.237
6	630.72	71.417
7	723.31	20.114
8	792.74	67.451
9	846.75	60.141
10	871.82	41.764
11	896.90	67.982
12	968.27	59.451
13	989.48	63.006
14	1016.49	41.126
15	1045.42	56.527
16	1097.50	28.541
17	1242.16	27.610
18	1338.60	60.550
19	1408.04	66.716
20	1454.33	87.923
21	1471.69	86.198
22	1577.77	90.177
23	1612.49	90.915
24	1714.72	27.198
25	2852.72	90.364
26	2906.73	88.556
27	2968.45	86.563
28	3429.43	90.102

Calculate...

Fig. S-2.3.1. FTIR spectrum of CLM – Transparent portion, inside.

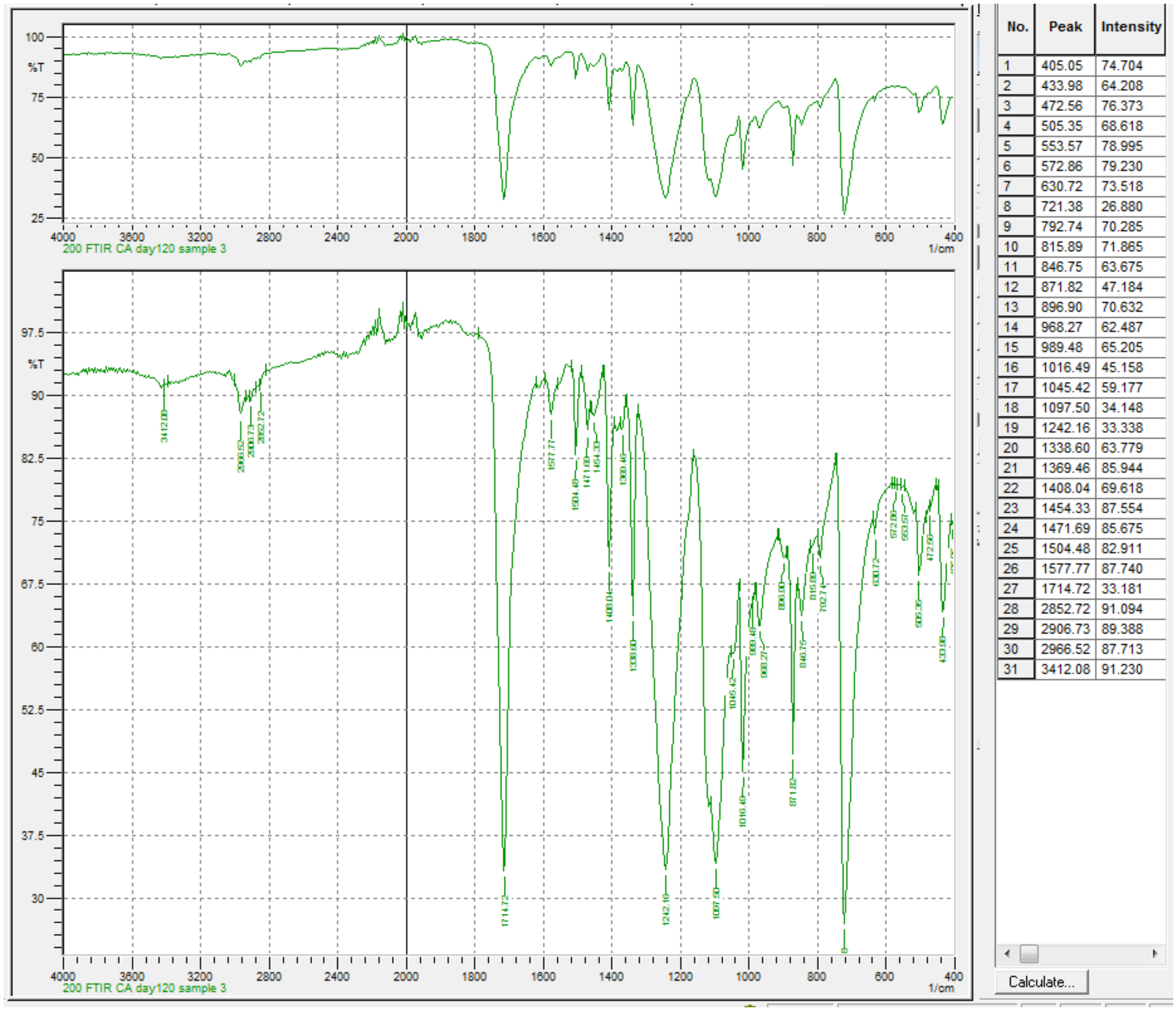

No.	Peak	Intensity
1	405.05	74.704
2	433.98	64.208
3	472.56	76.373
4	505.35	68.618
5	553.57	78.995
6	572.86	79.230
7	630.72	73.518
8	721.38	26.880
9	792.74	70.285
10	815.89	71.865
11	846.75	63.675
12	871.82	47.184
13	896.90	70.632
14	968.27	62.487
15	989.48	65.205
16	1016.49	45.158
17	1045.42	59.177
18	1097.50	34.148
19	1242.16	33.338
20	1338.60	63.779
21	1369.46	85.944
22	1408.04	69.618
23	1454.33	87.554
24	1471.69	85.675
25	1504.48	82.911
26	1577.77	87.740
27	1714.72	33.181
28	2852.72	91.094
29	2906.73	89.388
30	2966.52	87.713
31	3412.08	91.230

Calculate...

Fig. S-2.3.2. FTIR spectrum of CLM – Transparent portion, outside.

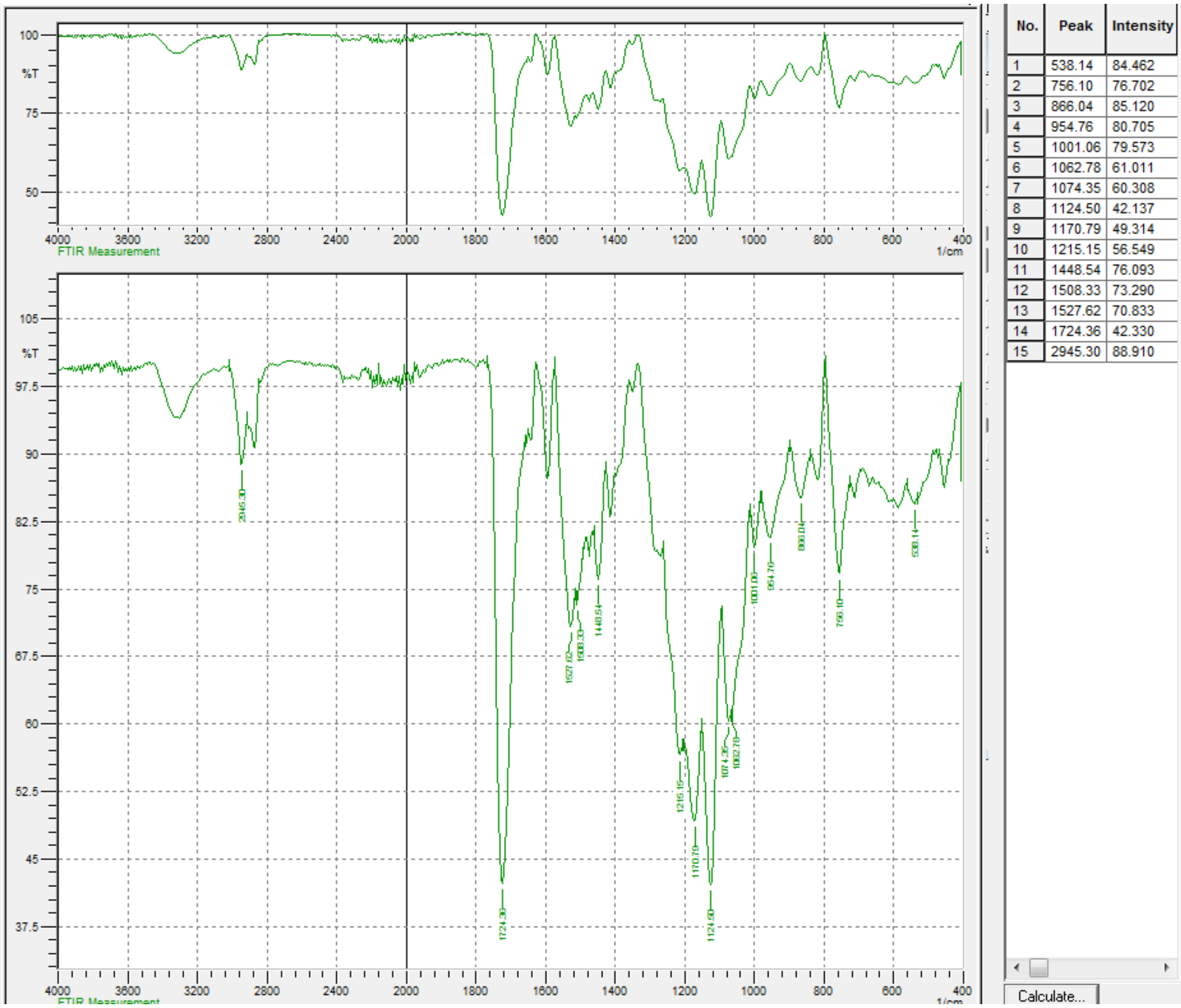

No.	Peak	Intensity
1	538.14	84.462
2	756.10	76.702
3	866.04	85.120
4	954.76	80.705
5	1001.06	79.573
6	1062.78	61.011
7	1074.35	60.308
8	1124.50	42.137
9	1170.79	49.314
10	1215.15	56.549
11	1448.54	76.093
12	1508.33	73.290
13	1527.62	70.833
14	1724.36	42.330
15	2945.30	88.910

Calculate...

Fig. S-2.3.3. FTIR spectrum of CLM – Breathable/Structural portion.

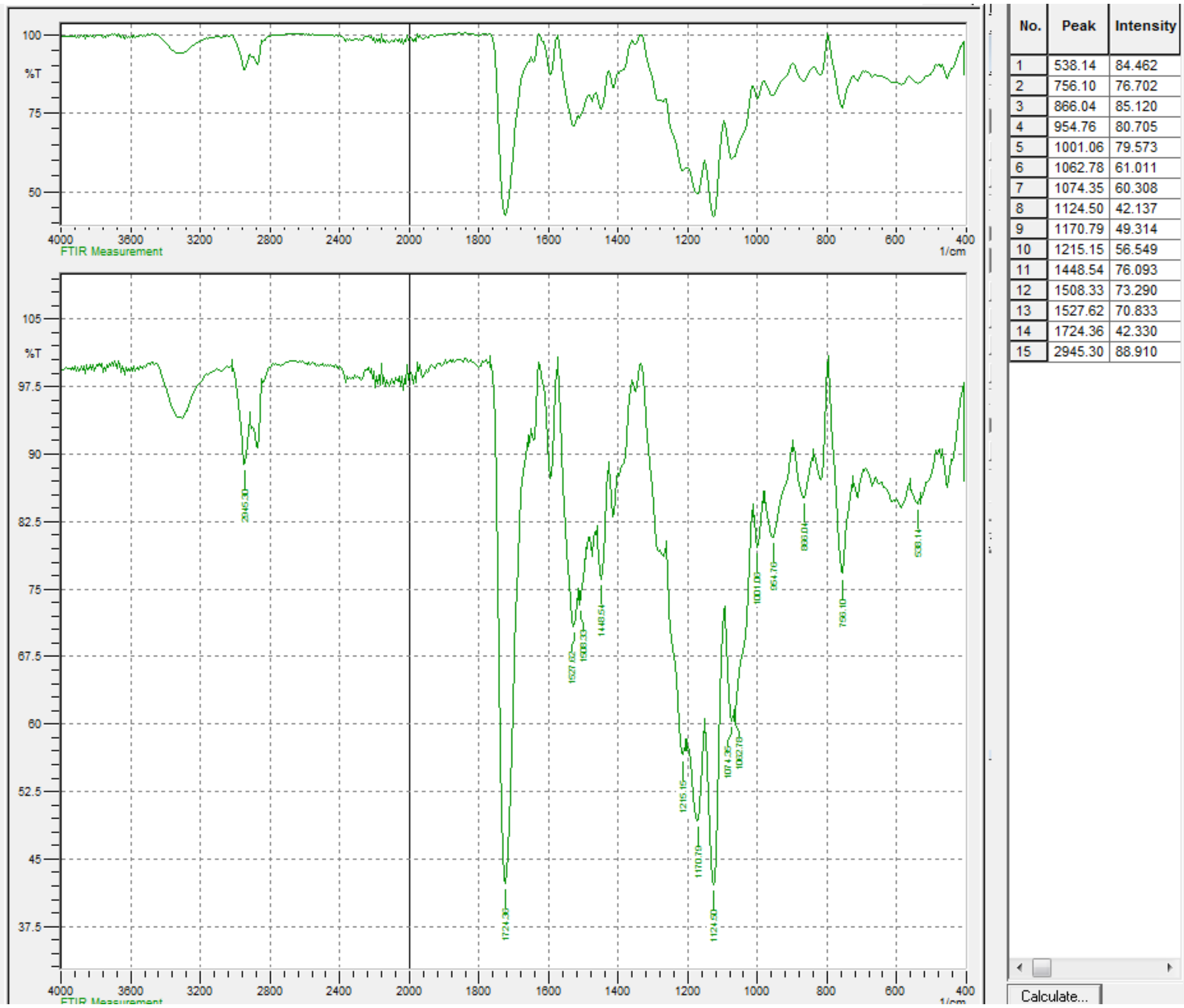

No.	Peak	Intensity
1	538.14	84.462
2	756.10	76.702
3	866.04	85.120
4	954.76	80.705
5	1001.06	79.573
6	1062.78	61.011
7	1074.35	60.308
8	1124.50	42.137
9	1170.79	49.314
10	1215.15	56.549
11	1448.54	76.093
12	1508.33	73.290
13	1527.62	70.833
14	1724.36	42.330
15	2945.30	88.910

Calculate...

Fig. S-2.3.4. FTIR spectrum of CLM – Nose bridge.

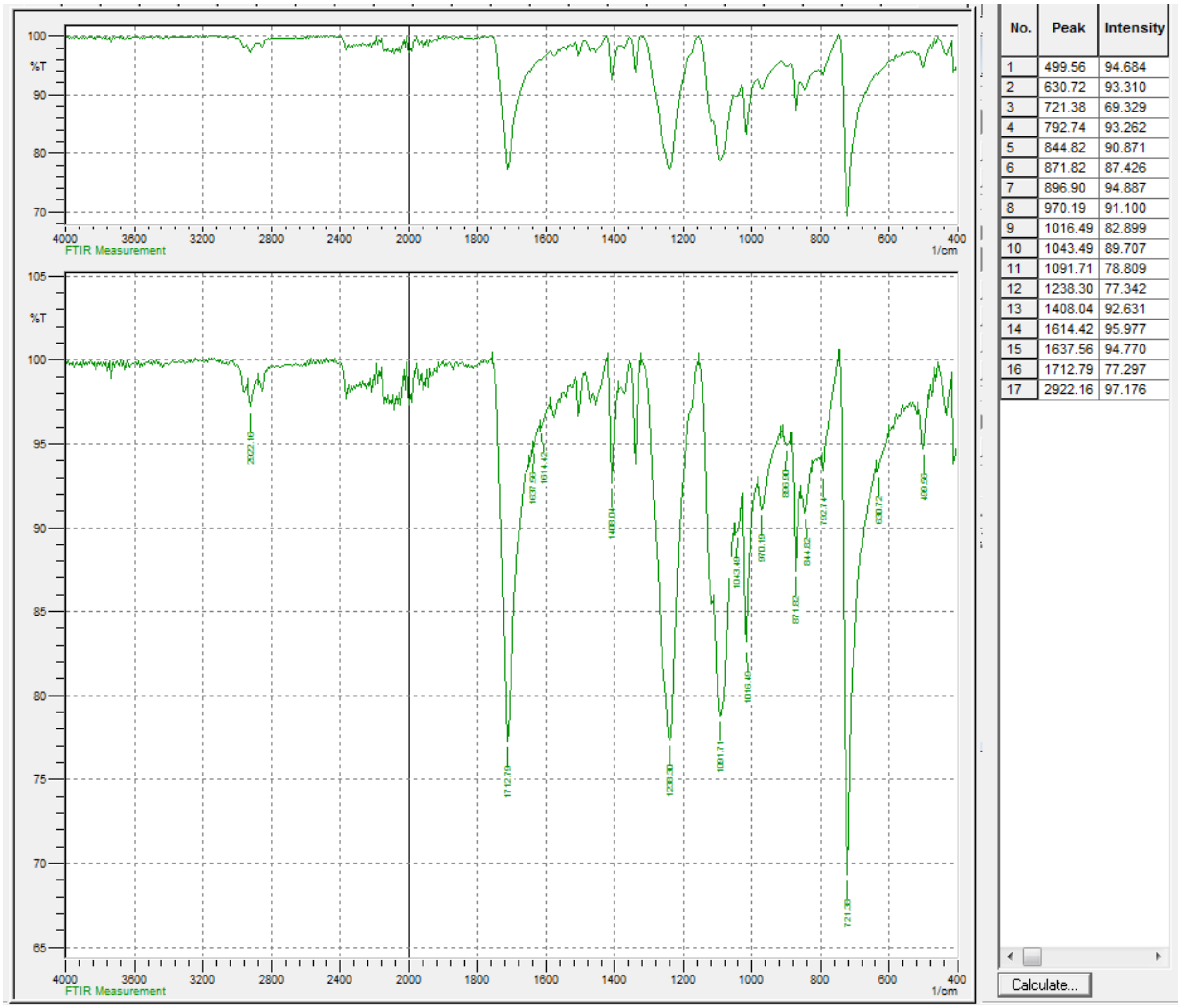

No.	Peak	Intensity
1	499.56	94.684
2	630.72	93.310
3	721.38	69.329
4	792.74	93.262
5	844.82	90.871
6	871.82	87.426
7	896.90	94.887
8	970.19	91.100
9	1016.49	82.899
10	1043.49	89.707
11	1091.71	78.809
12	1238.30	77.342
13	1408.04	92.631
14	1614.42	95.977
15	1637.56	94.770
16	1712.79	77.297
17	2922.16	97.176

Fig. S-2.3.4. FTIR spectrum of CLM – Ear loop.

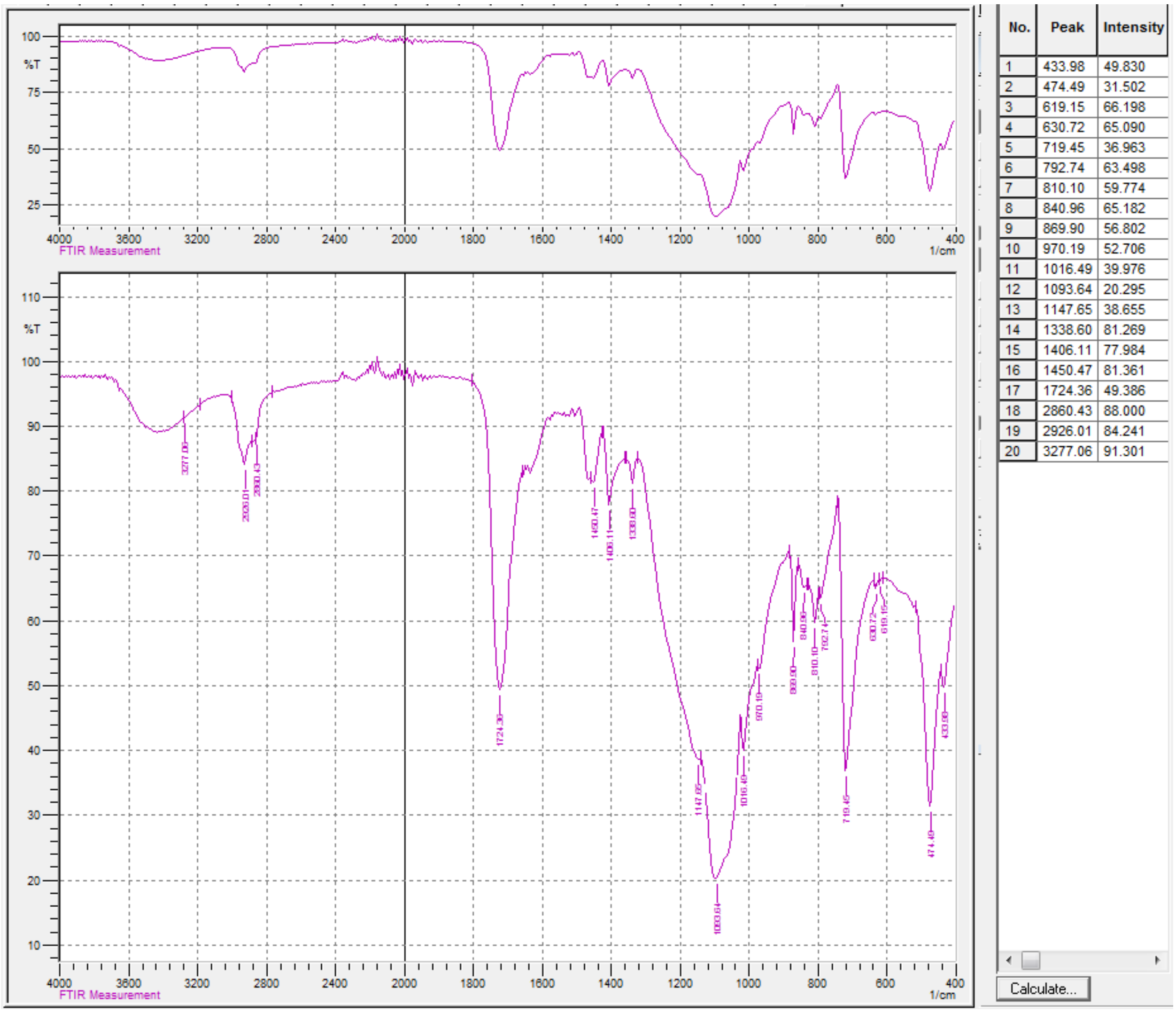

No.	Peak	Intensity
1	433.98	49.830
2	474.49	31.502
3	619.15	66.198
4	630.72	65.090
5	719.45	36.963
6	792.74	63.498
7	810.10	59.774
8	840.96	65.182
9	869.90	56.802
10	970.19	52.706
11	1016.49	39.976
12	1093.64	20.295
13	1147.65	38.655
14	1338.60	81.269
15	1406.11	77.984
16	1450.47	81.361
17	1724.36	49.386
18	2860.43	88.000
19	2926.01	84.241
20	3277.06	91.301

Calculate...

Fig. S-2.4.1. FTIR spectrum of FAV – Transparent portion, inside.

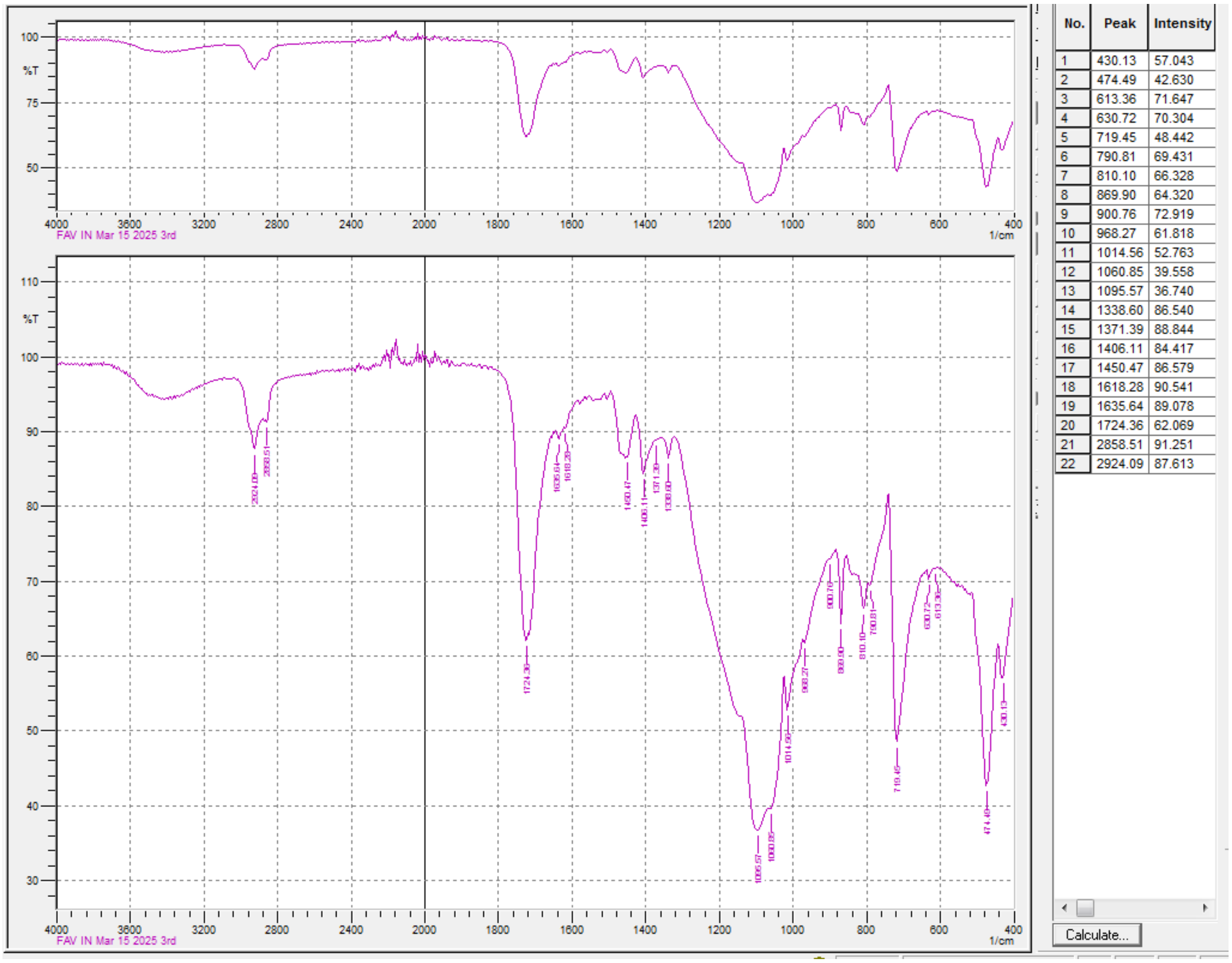

No.	Peak	Intensity
1	430.13	57.043
2	474.49	42.630
3	613.36	71.647
4	630.72	70.304
5	719.45	48.442
6	790.81	69.431
7	810.10	66.328
8	869.90	64.320
9	900.76	72.919
10	968.27	61.818
11	1014.56	52.763
12	1060.85	39.558
13	1095.57	36.740
14	1338.60	86.540
15	1371.39	88.844
16	1406.11	84.417
17	1450.47	86.579
18	1618.28	90.541
19	1635.64	89.078
20	1724.36	62.069
21	2858.51	91.251
22	2924.09	87.613

Fig. S-2.4.3. FTIR spectrum of FAV – Transparent portion, outside.

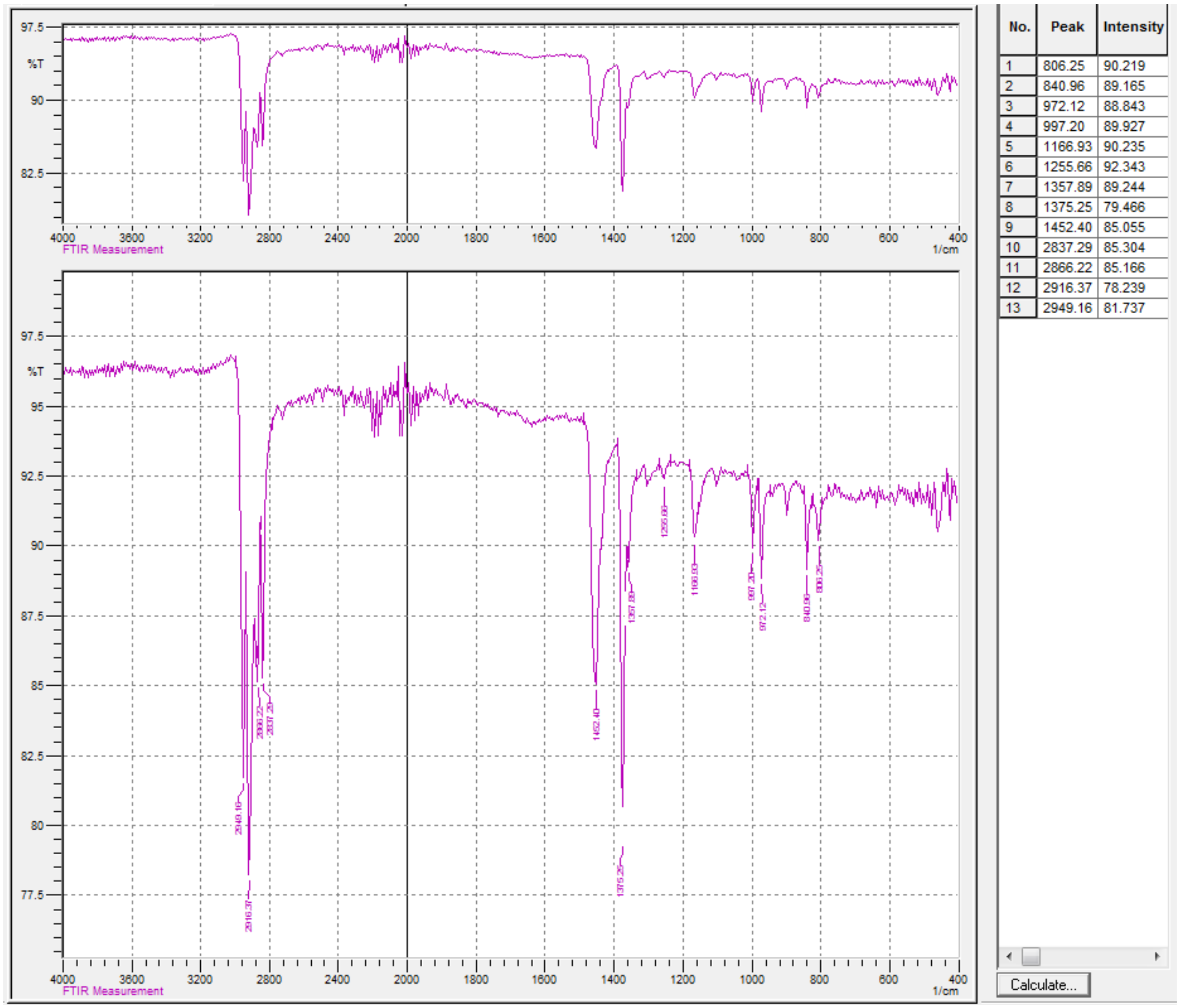

No.	Peak	Intensity
1	806.25	90.219
2	840.96	89.165
3	972.12	88.843
4	997.20	89.927
5	1166.93	90.235
6	1255.66	92.343
7	1357.89	89.244
8	1375.25	79.466
9	1452.40	85.055
10	2837.29	85.304
11	2866.22	85.166
12	2916.37	78.239
13	2949.16	81.737

Fig. S-2.4.3. FTIR spectrum of FAV – Breathable portion.

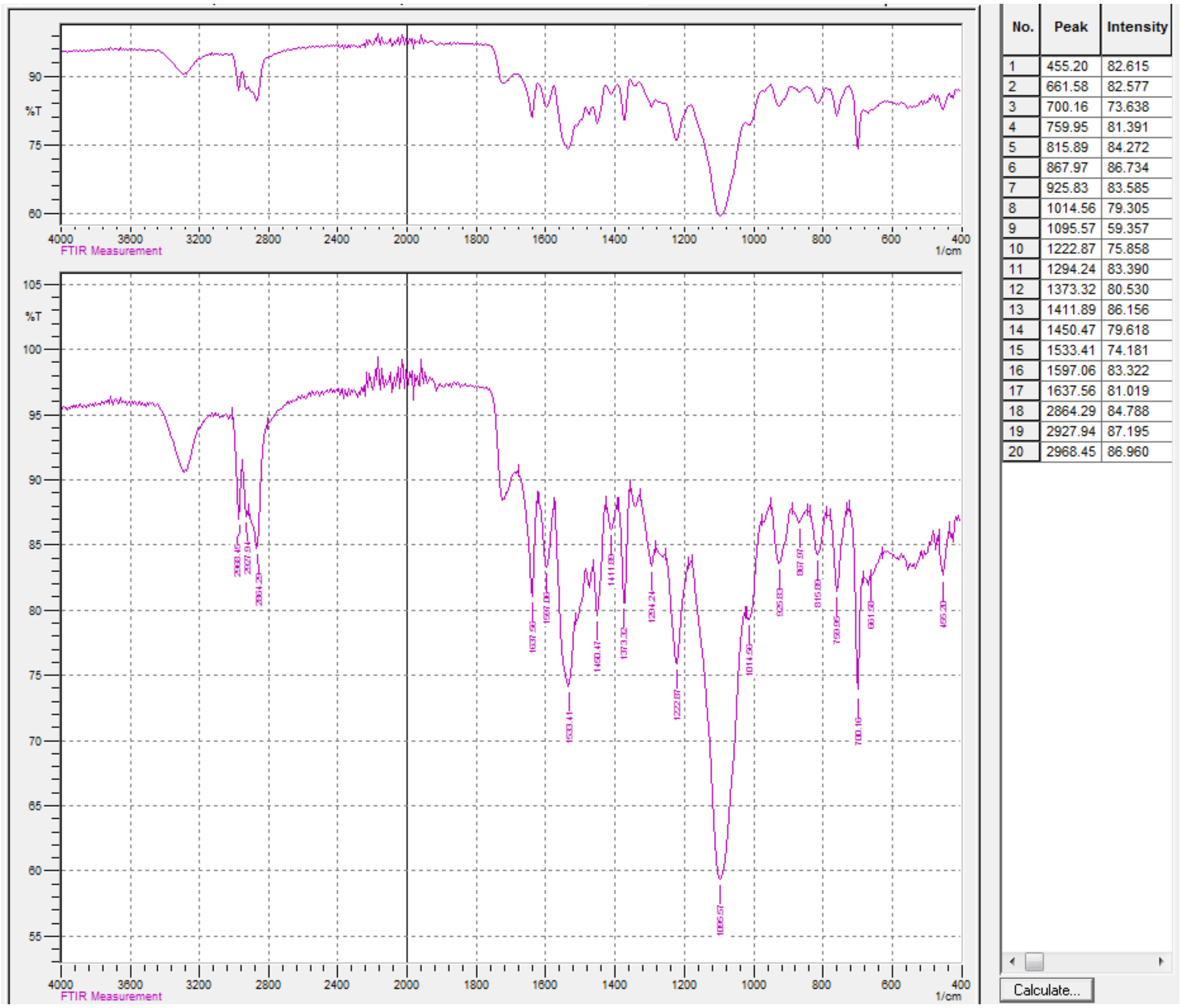

No.	Peak	Intensity
1	455.20	82.615
2	661.58	82.577
3	700.16	73.638
4	759.95	81.391
5	815.89	84.272
6	867.97	86.734
7	925.83	83.585
8	1014.56	79.305
9	1095.57	59.357
10	1222.87	75.858
11	1294.24	83.390
12	1373.32	80.530
13	1411.89	86.156
14	1450.47	79.618
15	1533.41	74.181
16	1597.06	83.322
17	1637.56	81.019
18	2864.29	84.788
19	2927.94	87.195
20	2968.45	86.960

Fig. S-2.4.4. FTIR spectrum of FAV – Nose bridge.

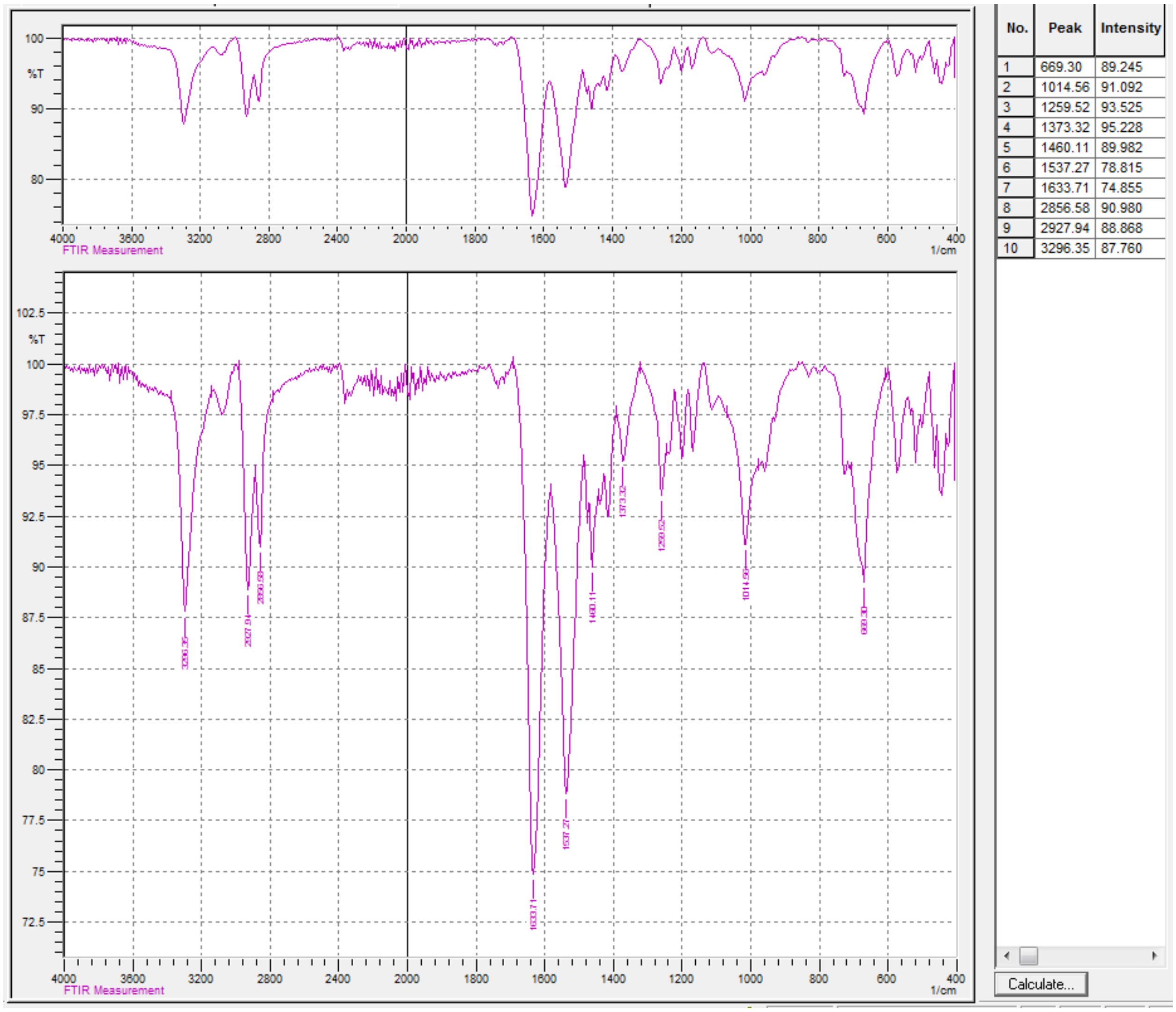

No.	Peak	Intensity
1	669.30	89.245
2	1014.56	91.092
3	1259.52	93.525
4	1373.32	95.228
5	1460.11	89.982
6	1537.27	78.815
7	1633.71	74.855
8	2856.58	90.980
9	2927.94	88.868
10	3296.35	87.760

Fig. S-2.4.5. FTIR spectrum of FAV – Ear loop.

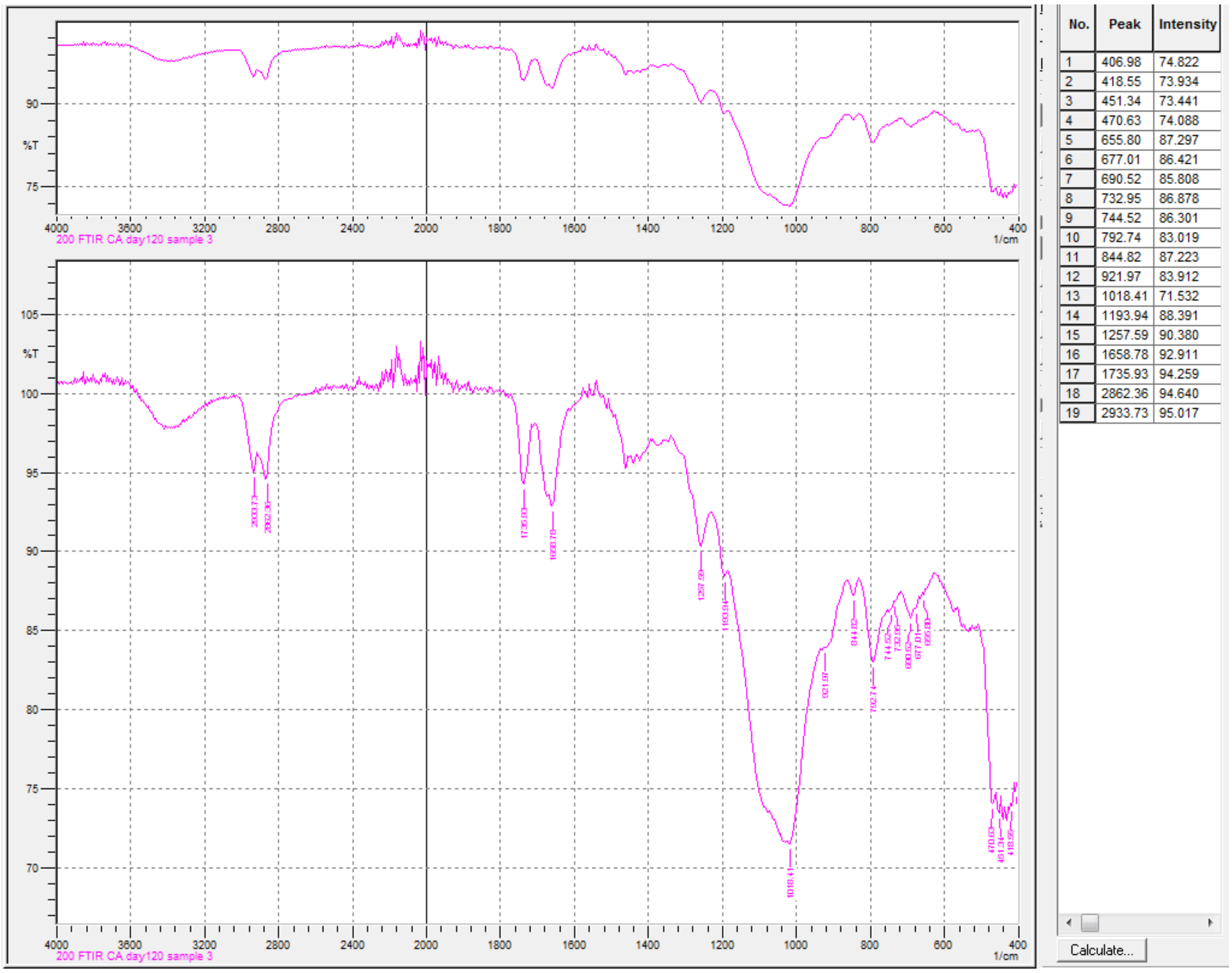

No.	Peak	Intensity
1	406.98	74.822
2	418.55	73.934
3	451.34	73.441
4	470.63	74.088
5	655.80	87.297
6	677.01	86.421
7	690.52	85.808
8	732.95	86.878
9	744.52	86.301
10	792.74	83.019
11	844.82	87.223
12	921.97	83.912
13	1018.41	71.532
14	1193.94	88.391
15	1257.59	90.380
16	1658.78	92.911
17	1735.93	94.259
18	2862.36	94.640
19	2933.73	95.017

Calculate...

Fig. S-2.5.1. FTIR spectrum of JEM – Transparent portion (heated to 150 °C), inside.

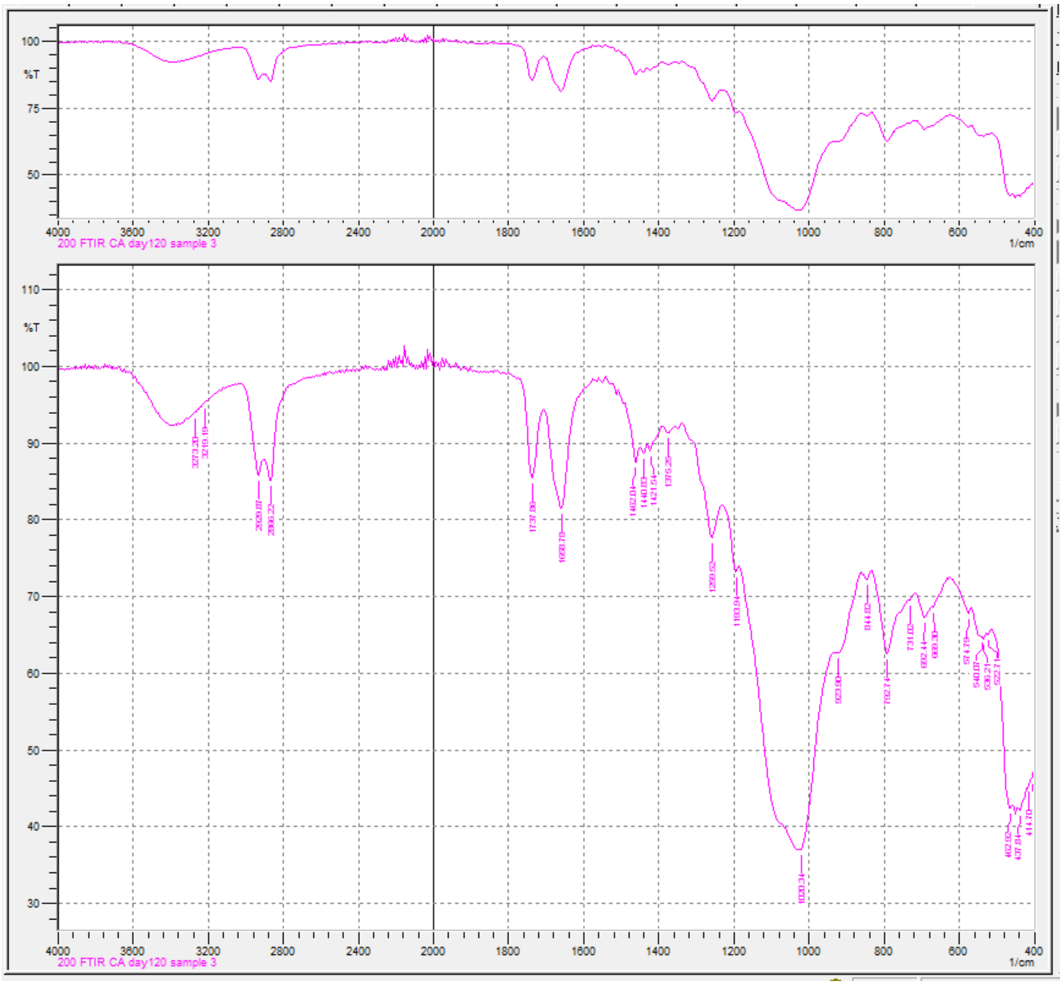

No.	Peak	Intensity
1	406.98	46.349
2	414.70	45.051
3	437.84	42.130
4	462.92	42.495
5	522.71	65.068
6	536.21	64.468
7	540.07	64.722
8	574.79	67.829
9	669.30	68.639
10	692.44	67.234
11	731.02	69.474
12	792.74	62.585
13	844.82	72.200
14	923.90	62.652
15	1020.34	37.025
16	1193.94	73.211
17	1259.52	77.719
18	1375.25	91.353
19	1421.54	89.032
20	1440.83	88.610
21	1462.04	87.568
22	1658.78	81.436
23	1737.86	85.513
24	2866.22	85.124
25	2929.87	85.755
26	3219.19	95.262
27	3273.20	93.892

Fig. S-2.5.2. FTIR spectrum of JEM – Transparent portion (heated to 150 ^{o}C), outside.

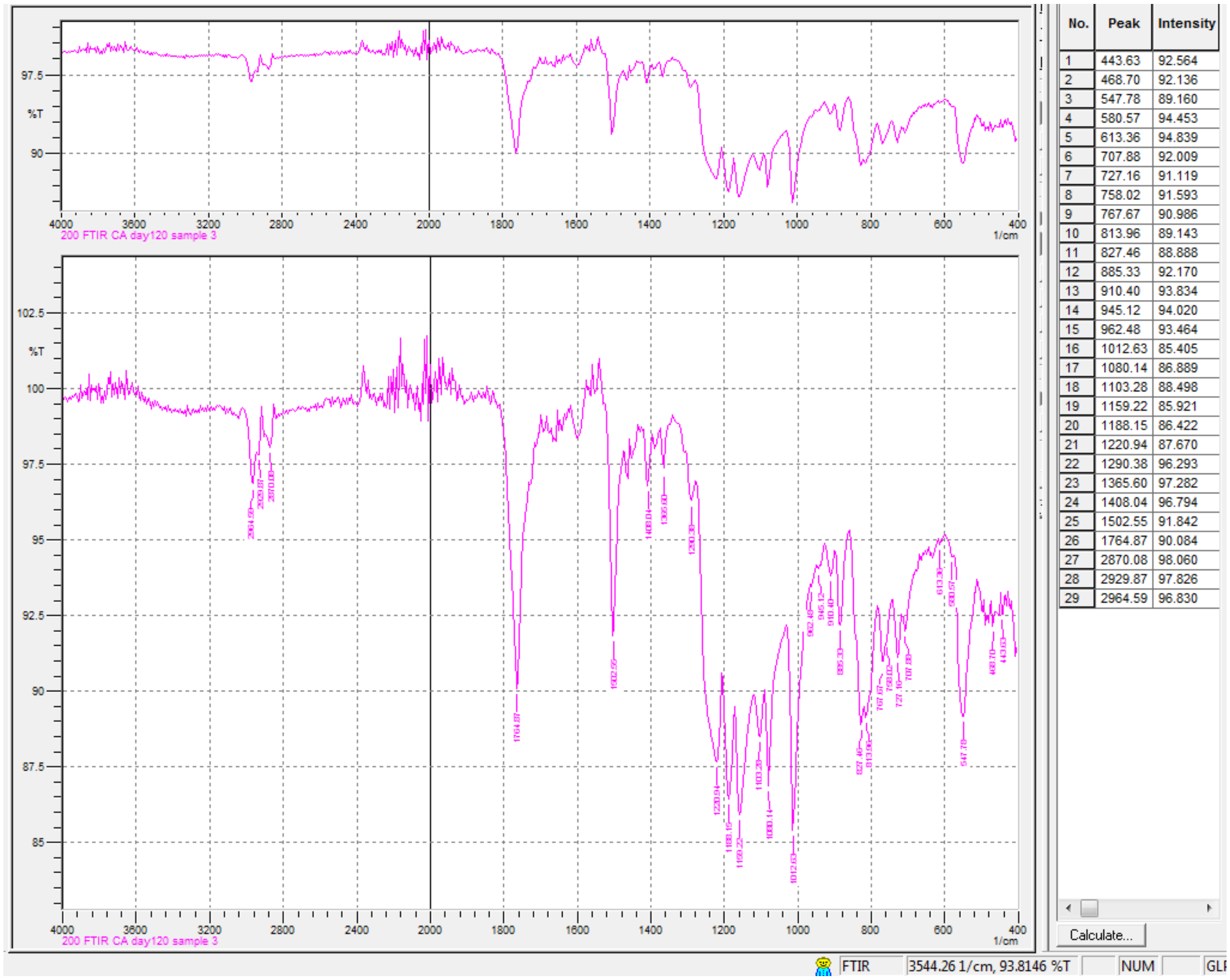

No.	Peak	Intensity
1	443.63	92.564
2	468.70	92.136
3	547.78	89.160
4	580.57	94.453
5	613.36	94.839
6	707.88	92.009
7	727.16	91.119
8	758.02	91.593
9	767.67	90.986
10	813.96	89.143
11	827.46	88.888
12	885.33	92.170
13	910.40	93.834
14	945.12	94.020
15	962.48	93.464
16	1012.63	85.405
17	1080.14	86.889
18	1103.28	88.498
19	1159.22	85.921
20	1188.15	86.422
21	1220.94	87.670
22	1290.38	96.293
23	1365.60	97.282
24	1408.04	96.794
25	1502.55	91.842
26	1764.87	90.084
27	2870.08	98.060
28	2929.87	97.826
29	2964.59	96.830

Fig. S-2.5.3. FTIR spectrum of JEM – Transparent portion, fraction extracted with chloroform.

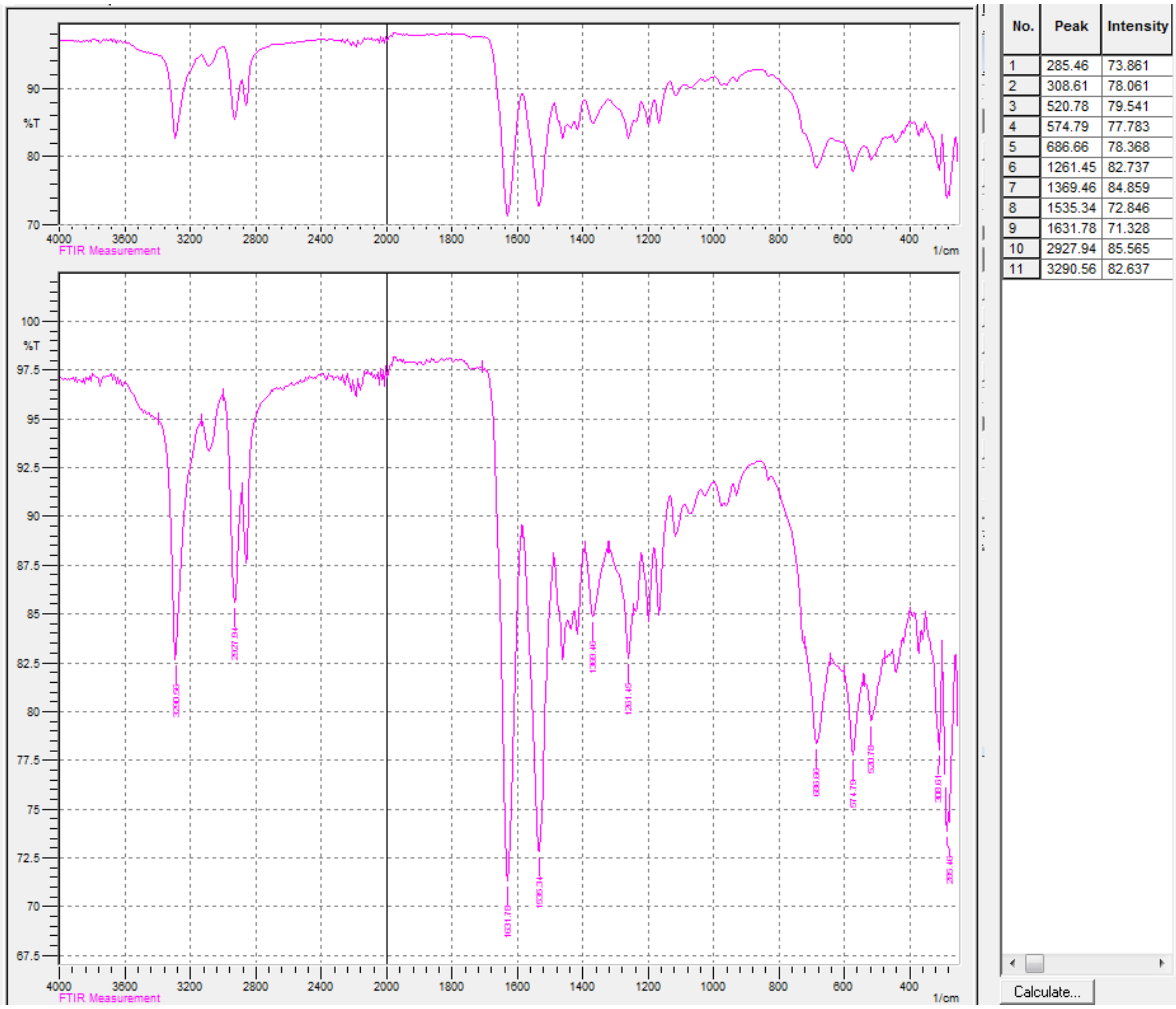

No.	Peak	Intensity
1	285.46	73.861
2	308.61	78.061
3	520.78	79.541
4	574.79	77.783
5	686.66	78.368
6	1261.45	82.737
7	1369.46	84.859
8	1535.34	72.846
9	1631.78	71.328
10	2927.94	85.565
11	3290.56	82.637

Calculate...

Fig. S-2.5.4. FTIR spectrum of JEM – Structural portion (Filter cap).

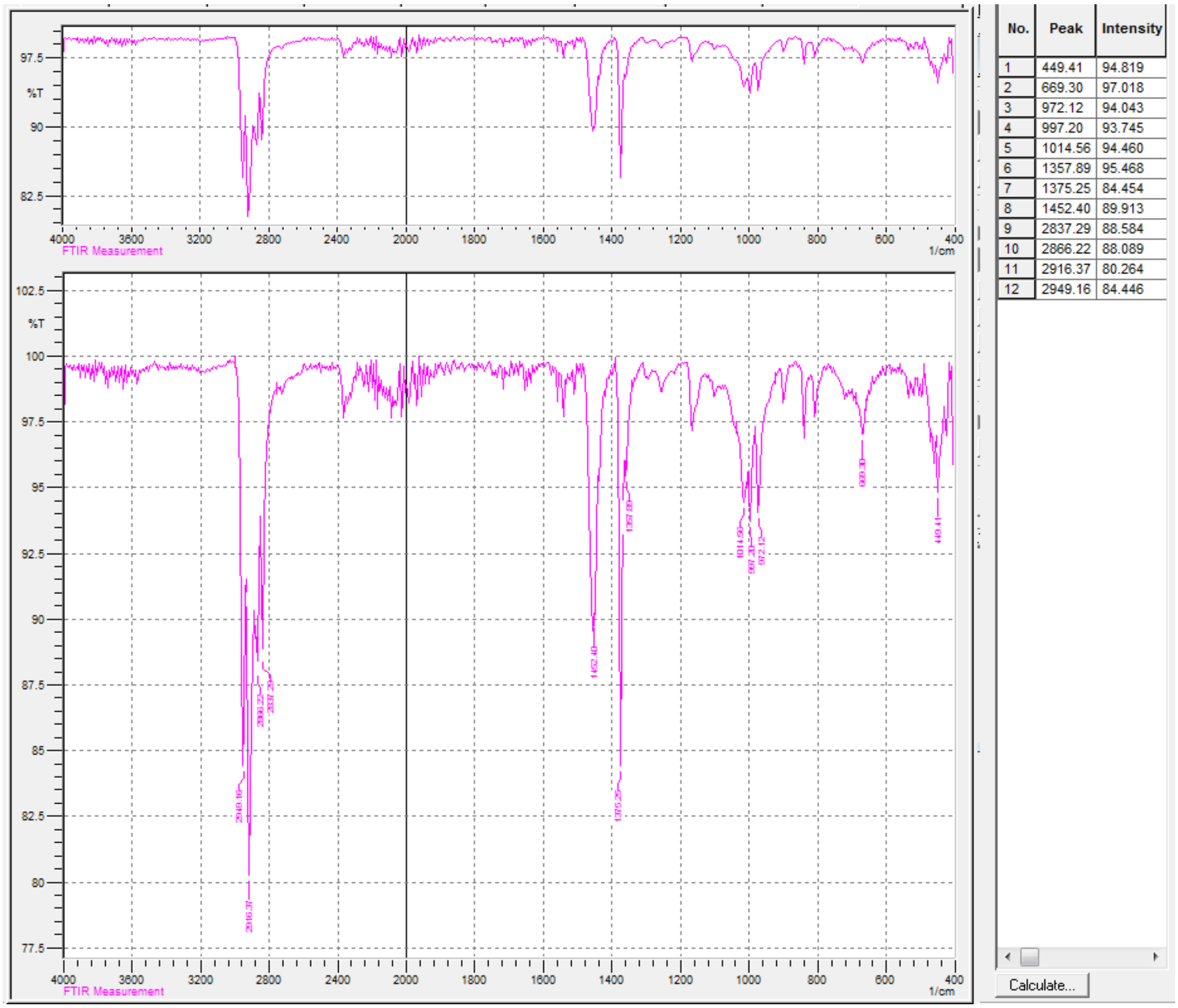

No.	Peak	Intensity
1	449.41	94.819
2	669.30	97.018
3	972.12	94.043
4	997.20	93.745
5	1014.56	94.460
6	1357.89	95.468
7	1375.25	84.454
8	1452.40	89.913
9	2837.29	88.584
10	2866.22	88.089
11	2916.37	80.264
12	2949.16	84.446

Calculate...

Fig. S-2.5.5. FTIR spectrum of JEM – Filter.

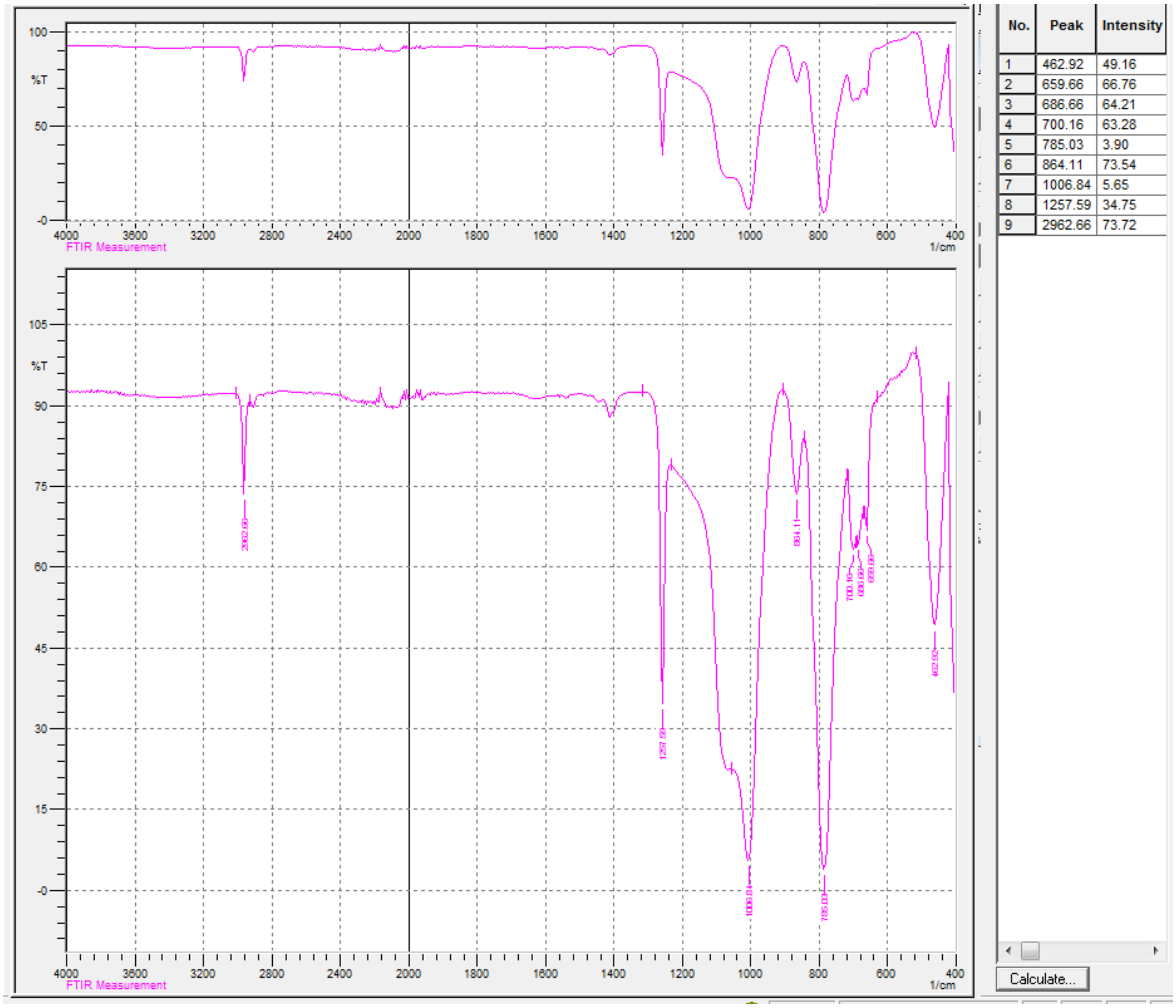

Fig. S-2.5.6. FTIR spectrum of JEM – Seal.

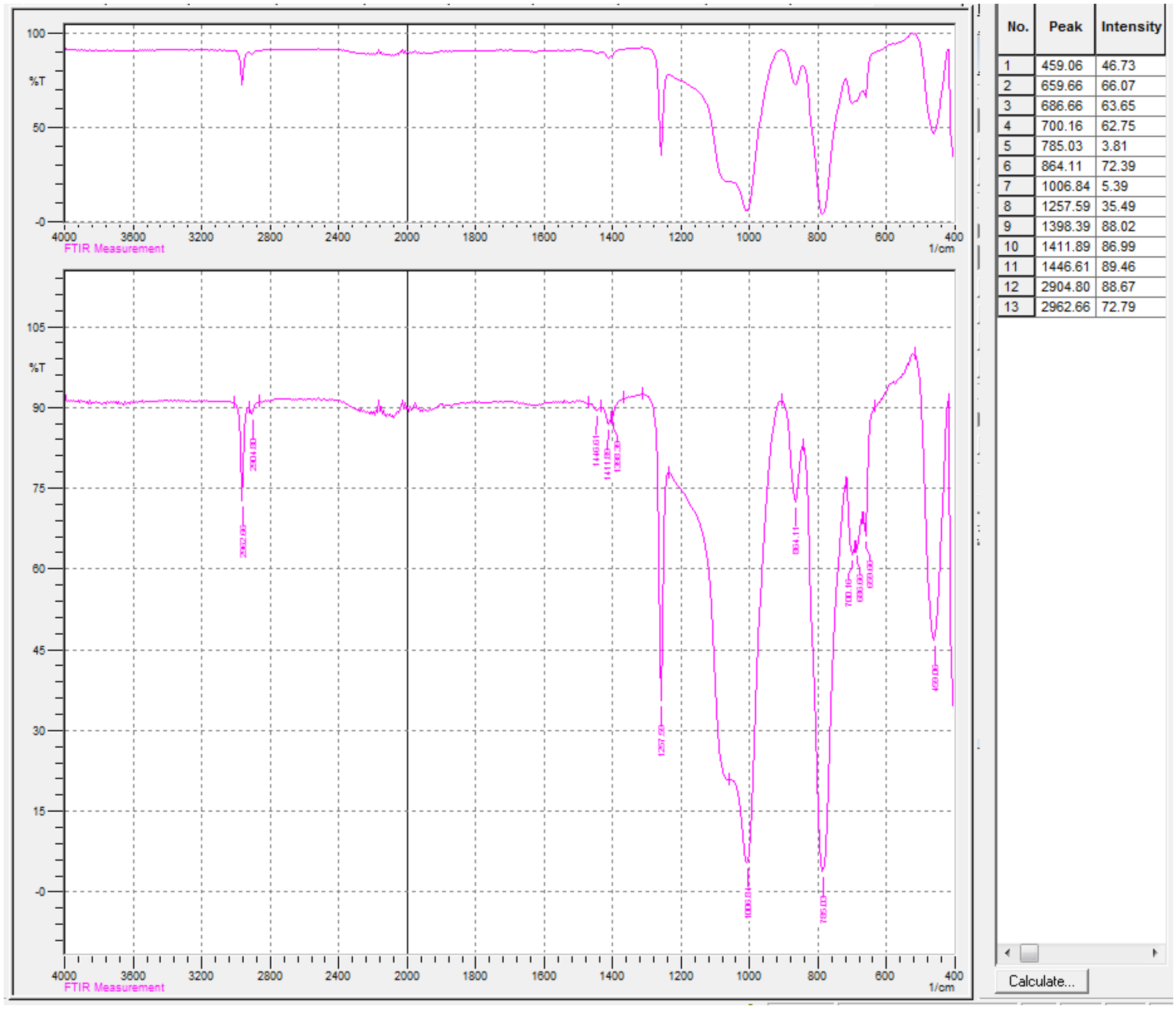

No.	Peak	Intensity
1	459.06	46.73
2	659.66	66.07
3	686.66	63.65
4	700.16	62.75
5	785.03	3.81
6	864.11	72.39
7	1006.84	5.39
8	1257.59	35.49
9	1398.39	88.02
10	1411.89	86.99
11	1446.61	89.46
12	2904.80	88.67
13	2962.66	72.79

Fig. S-2.5.7. FTIR spectrum of JEM – Ear loop.

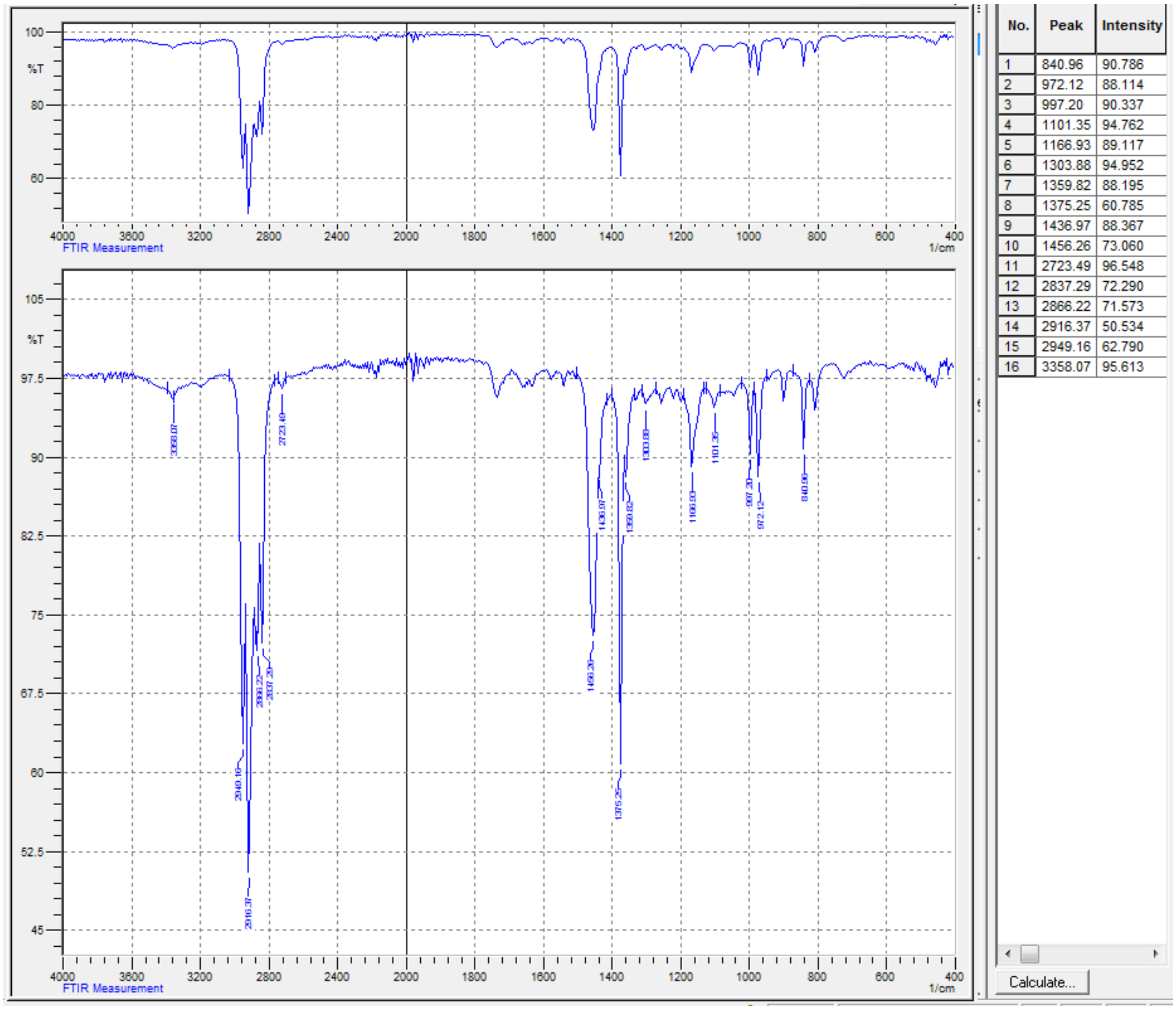

Fig. S-2.6.1. FTIR spectrum of OPT – Transparent portion, inside.

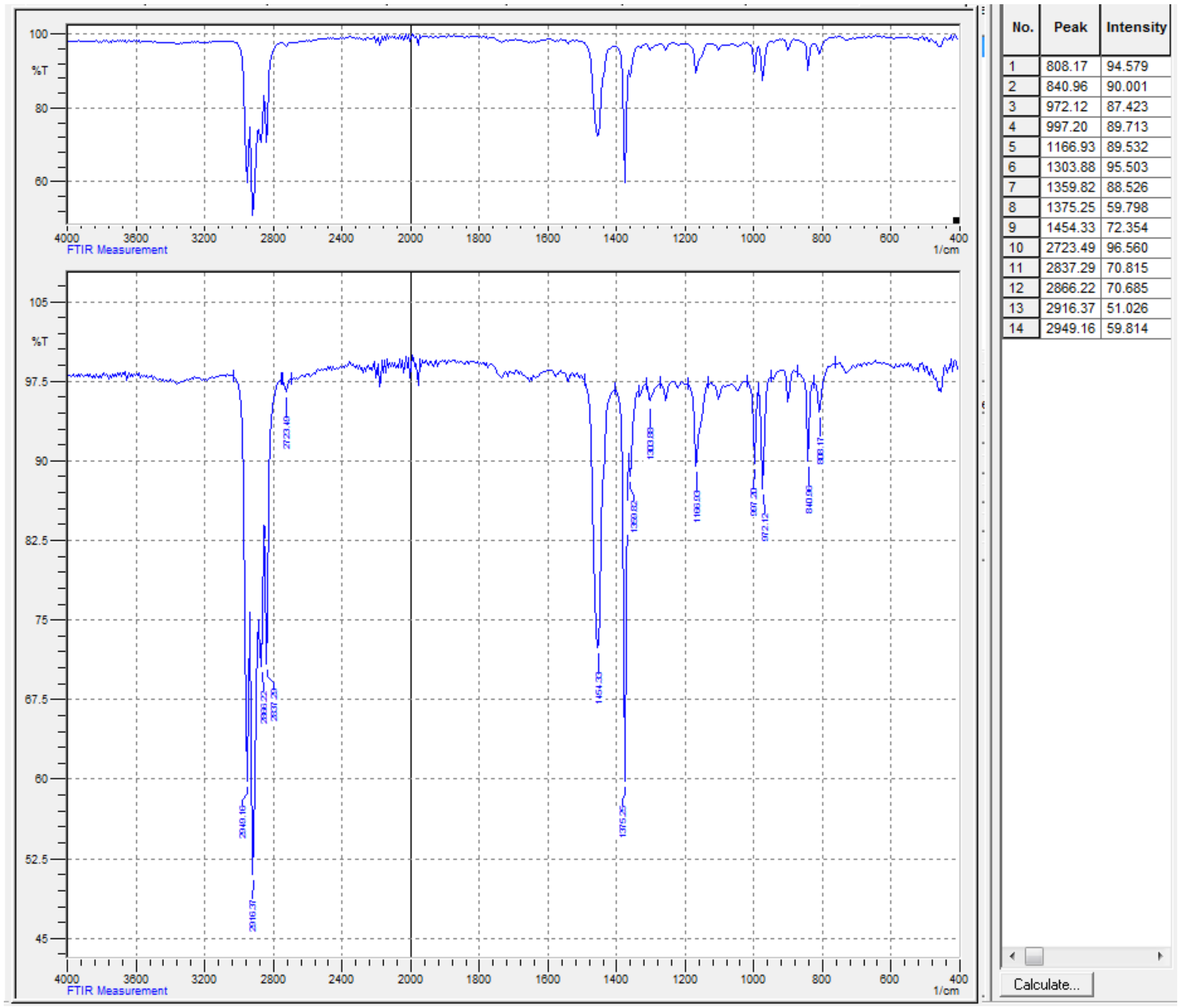

No.	Peak	Intensity
1	808.17	94.579
2	840.96	90.001
3	972.12	87.423
4	997.20	89.713
5	1166.93	89.532
6	1303.88	95.503
7	1359.82	88.526
8	1375.25	59.798
9	1454.33	72.354
10	2723.49	96.560
11	2837.29	70.815
12	2866.22	70.685
13	2916.37	51.026
14	2949.16	59.814

Calculate...

Fig. S-2.6.2. FTIR spectrum of OPT – Transparent portion, outside.

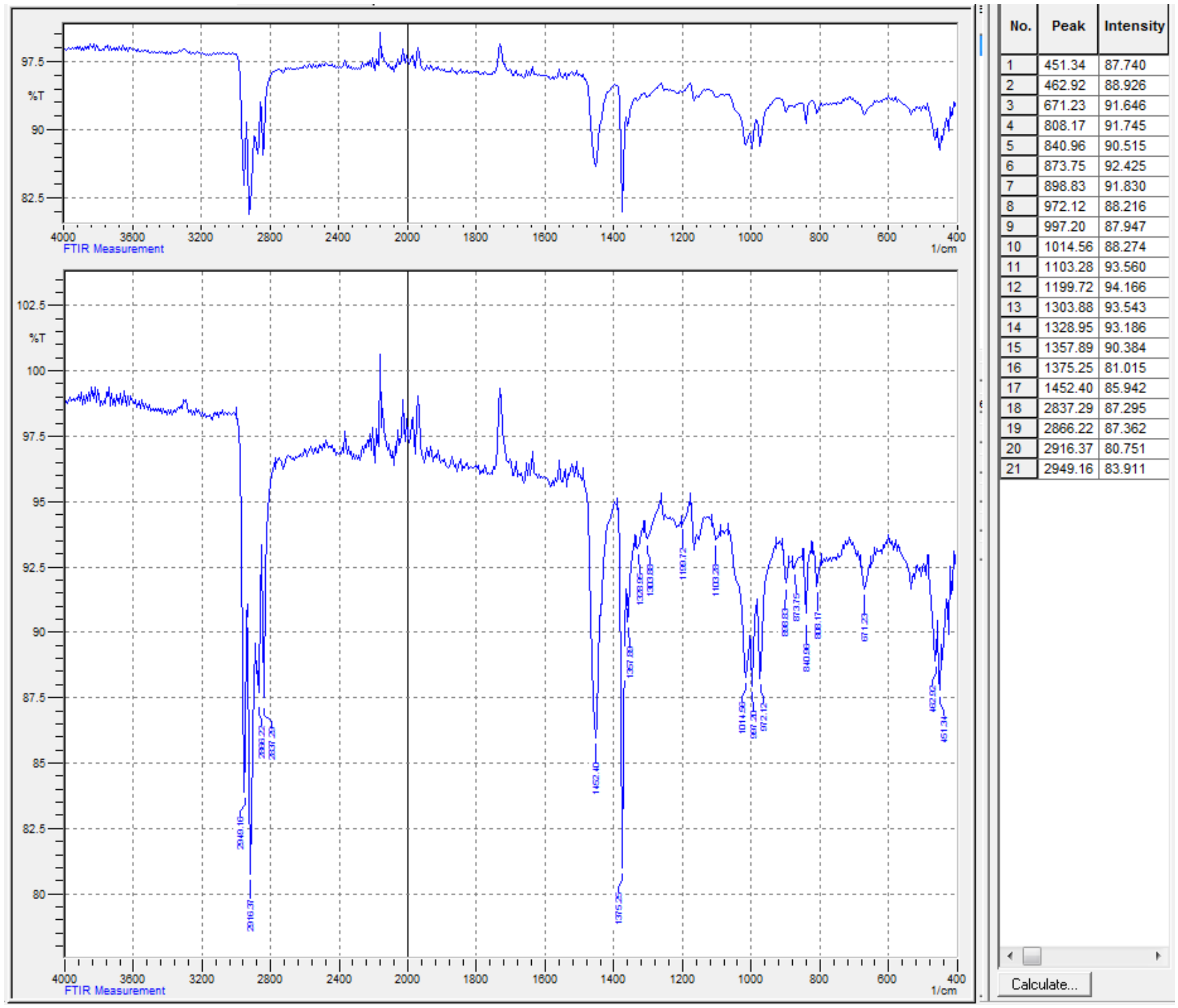

No.	Peak	Intensity
1	451.34	87.740
2	462.92	88.926
3	671.23	91.646
4	808.17	91.745
5	840.96	90.515
6	873.75	92.425
7	898.83	91.830
8	972.12	88.216
9	997.20	87.947
10	1014.56	88.274
11	1103.28	93.560
12	1199.72	94.166
13	1303.88	93.543
14	1328.95	93.186
15	1357.89	90.384
16	1375.25	81.015
17	1452.40	85.942
18	2837.29	87.295
19	2866.22	87.362
20	2916.37	80.751
21	2949.16	83.911

Fig. S-2.6.3. FTIR spectrum of OPT – Breathable portion.

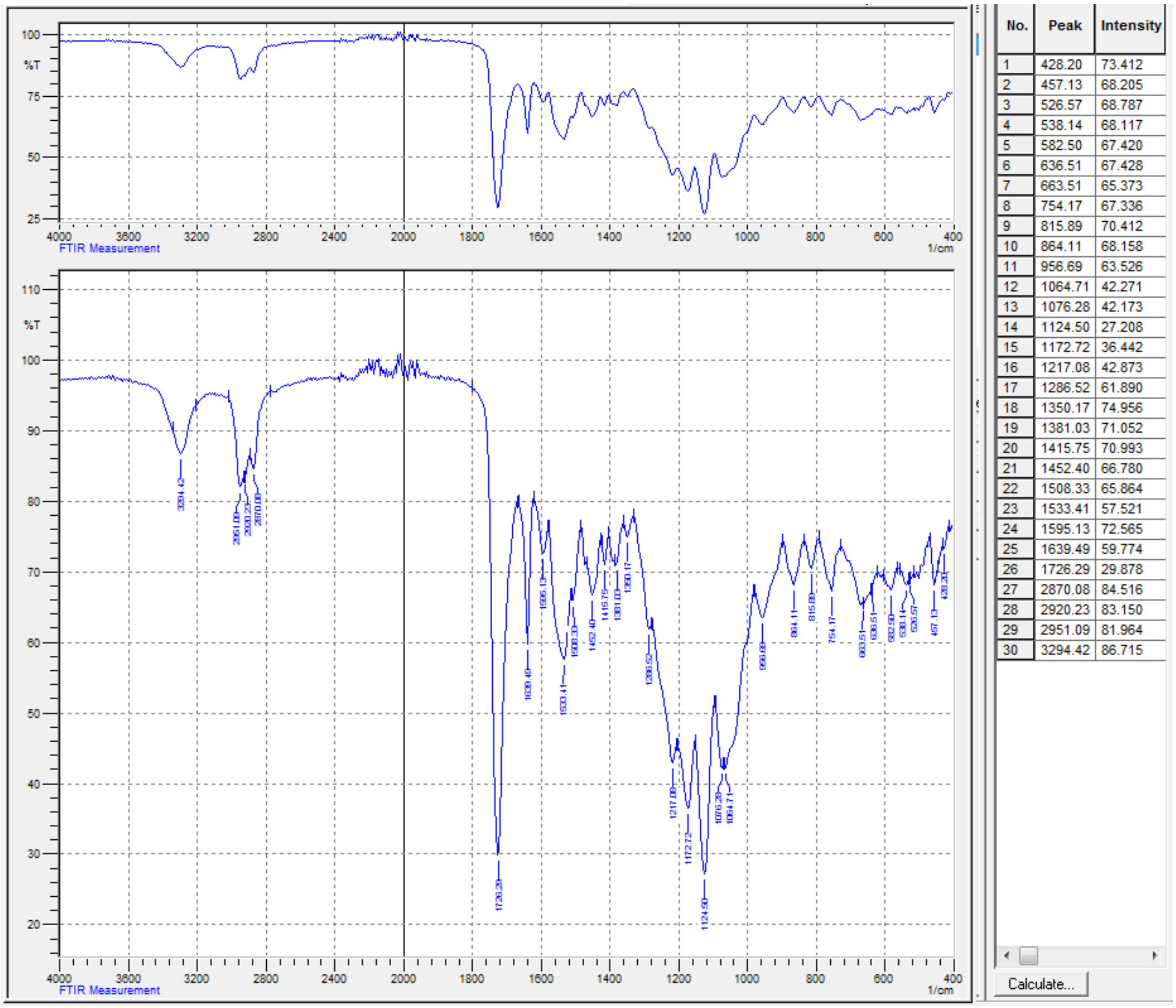

No.	Peak	Intensity
1	428.20	73.412
2	457.13	68.205
3	526.57	68.787
4	538.14	68.117
5	582.50	67.420
6	636.51	67.428
7	663.51	65.373
8	754.17	67.336
9	815.89	70.412
10	864.11	68.158
11	956.69	63.526
12	1064.71	42.271
13	1076.28	42.173
14	1124.50	27.208
15	1172.72	36.442
16	1217.08	42.873
17	1286.52	61.890
18	1350.17	74.956
19	1381.03	71.052
20	1415.75	70.993
21	1452.40	66.780
22	1508.33	65.864
23	1533.41	57.521
24	1595.13	72.565
25	1639.49	59.774
26	1726.29	29.878
27	2870.08	84.516
28	2920.23	83.150
29	2951.09	81.964
30	3294.42	86.715

Calculate...

Fig. S-2.6.4. FTIR spectrum of OPT – Nose bridge.

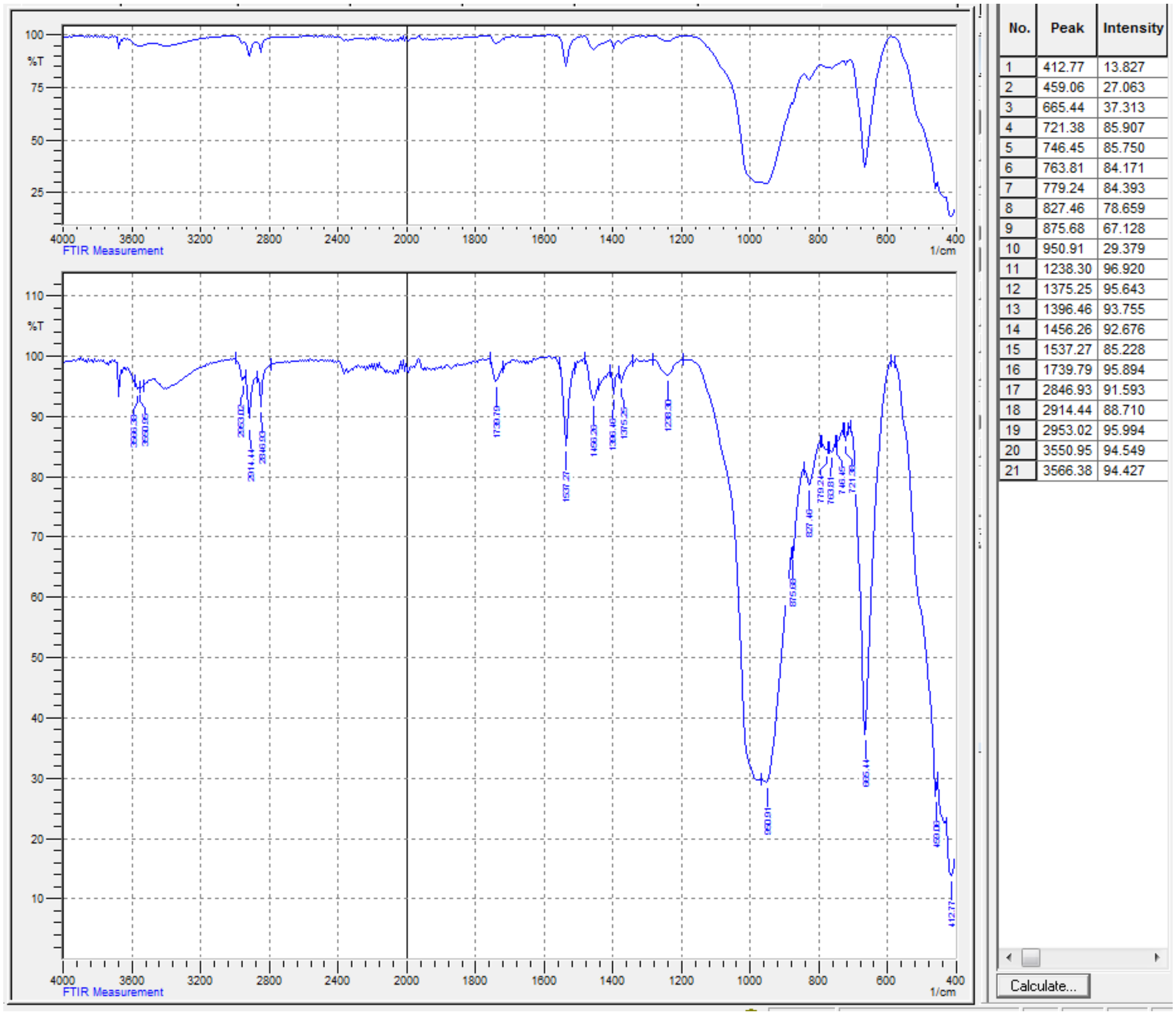

No.	Peak	Intensity
1	412.77	13.827
2	459.06	27.063
3	665.44	37.313
4	721.38	85.907
5	746.45	85.750
6	763.81	84.171
7	779.24	84.393
8	827.46	78.659
9	875.68	67.128
10	950.91	29.379
11	1238.30	96.920
12	1375.25	95.643
13	1396.46	93.755
14	1456.26	92.676
15	1537.27	85.228
16	1739.79	95.894
17	2846.93	91.593
18	2914.44	88.710
19	2953.02	95.994
20	3550.95	94.549
21	3566.38	94.427

Fig. S-2.6.5. FTIR spectrum of OPT – Ear loop.

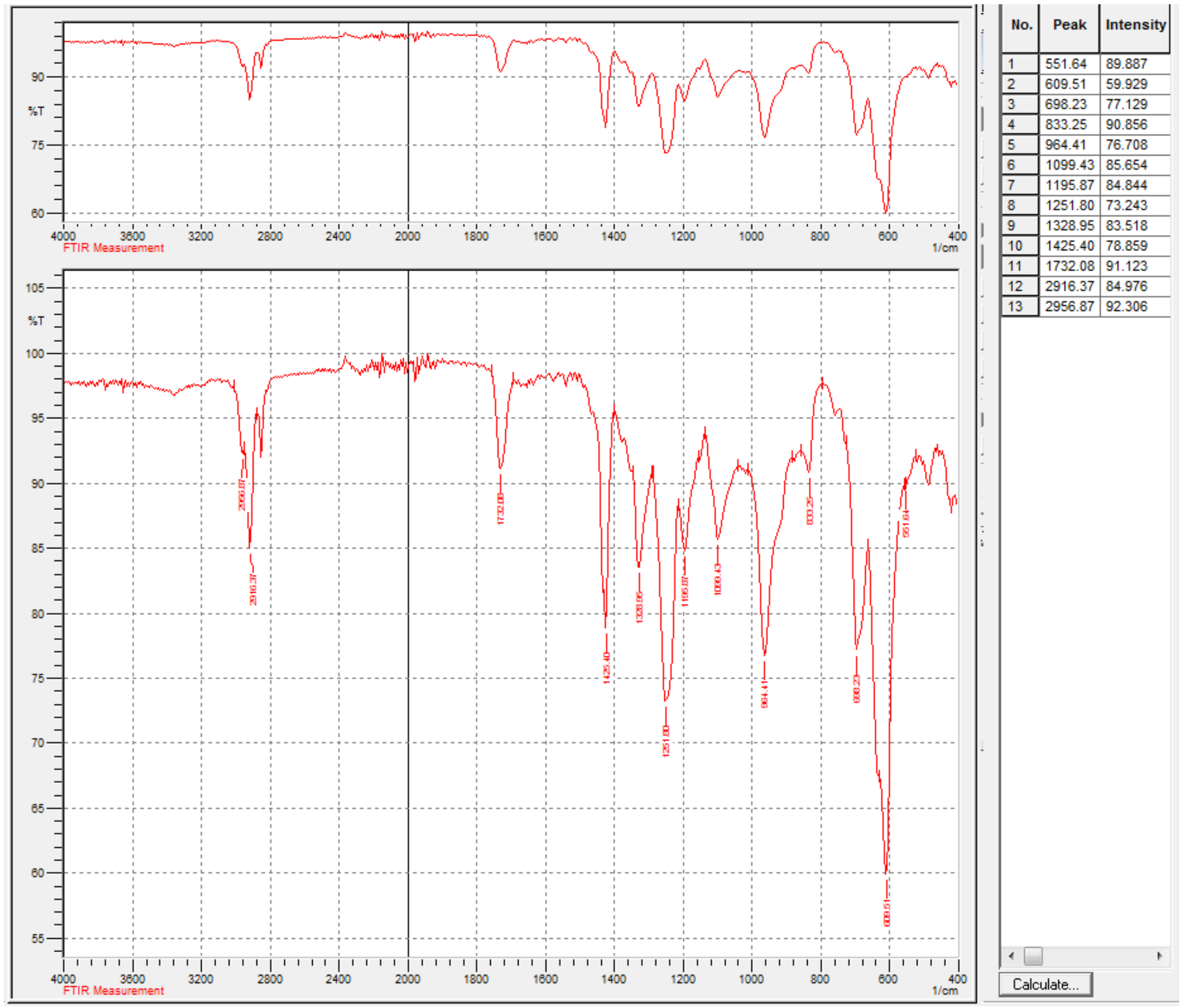

No.	Peak	Intensity
1	551.64	89.887
2	609.51	59.929
3	698.23	77.129
4	833.25	90.856
5	964.41	76.708
6	1099.43	85.654
7	1195.87	84.844
8	1251.80	73.243
9	1328.95	83.518
10	1425.40	78.859
11	1732.08	91.123
12	2916.37	84.976
13	2956.87	92.306

Calculate...

Fig. S-2.7.1. FTIR spectrum of RAN – Transparent portion, inside.

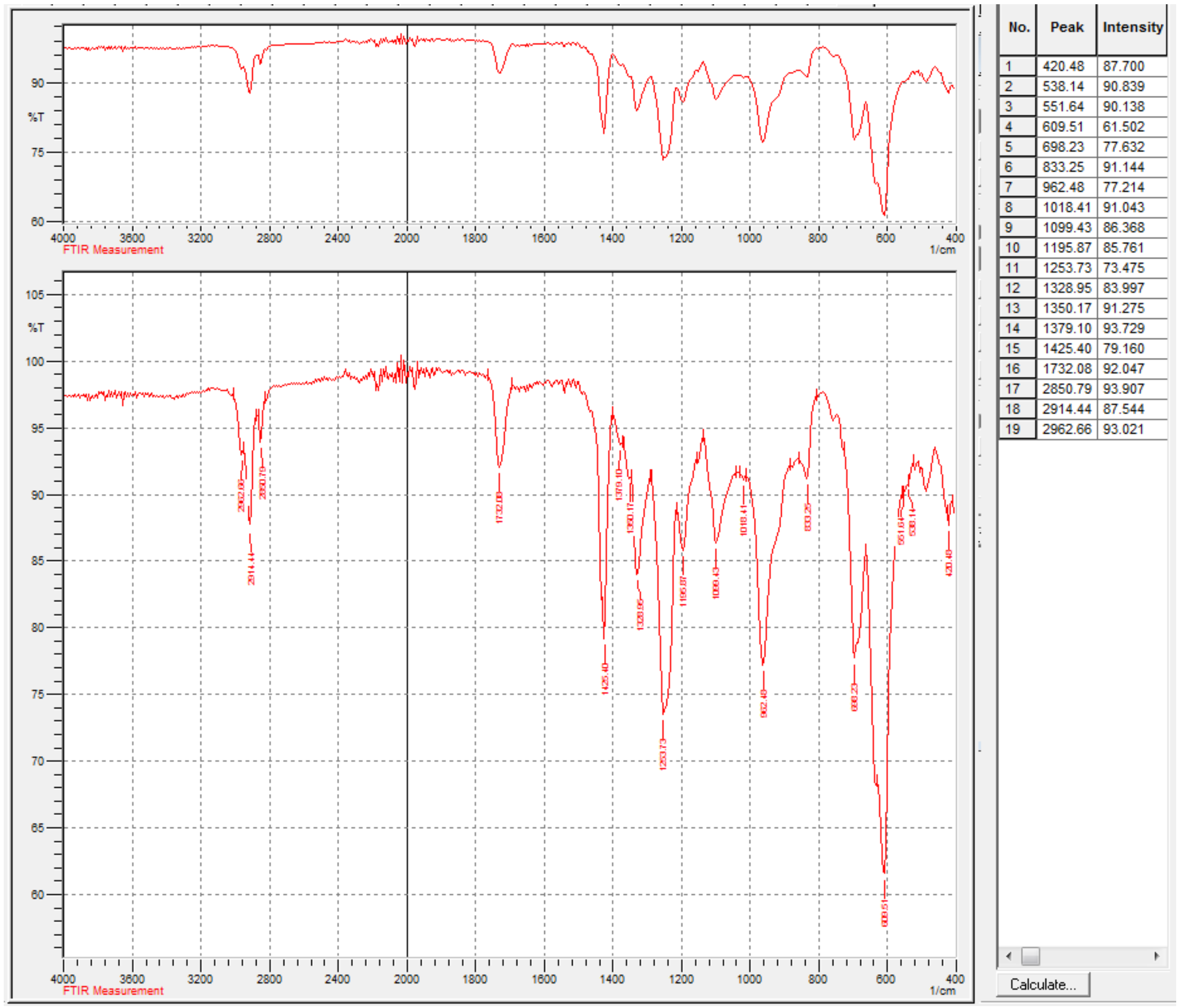

No.	Peak	Intensity
1	420.48	87.700
2	538.14	90.839
3	551.64	90.138
4	609.51	61.502
5	698.23	77.632
6	833.25	91.144
7	962.48	77.214
8	1018.41	91.043
9	1099.43	86.368
10	1195.87	85.761
11	1253.73	73.475
12	1328.95	83.997
13	1350.17	91.275
14	1379.10	93.729
15	1425.40	79.160
16	1732.08	92.047
17	2850.79	93.907
18	2914.44	87.544
19	2962.66	93.021

Calculate...

Fig. S-2.7.2. FTIR spectrum of RAN – Transparent portion, outside.

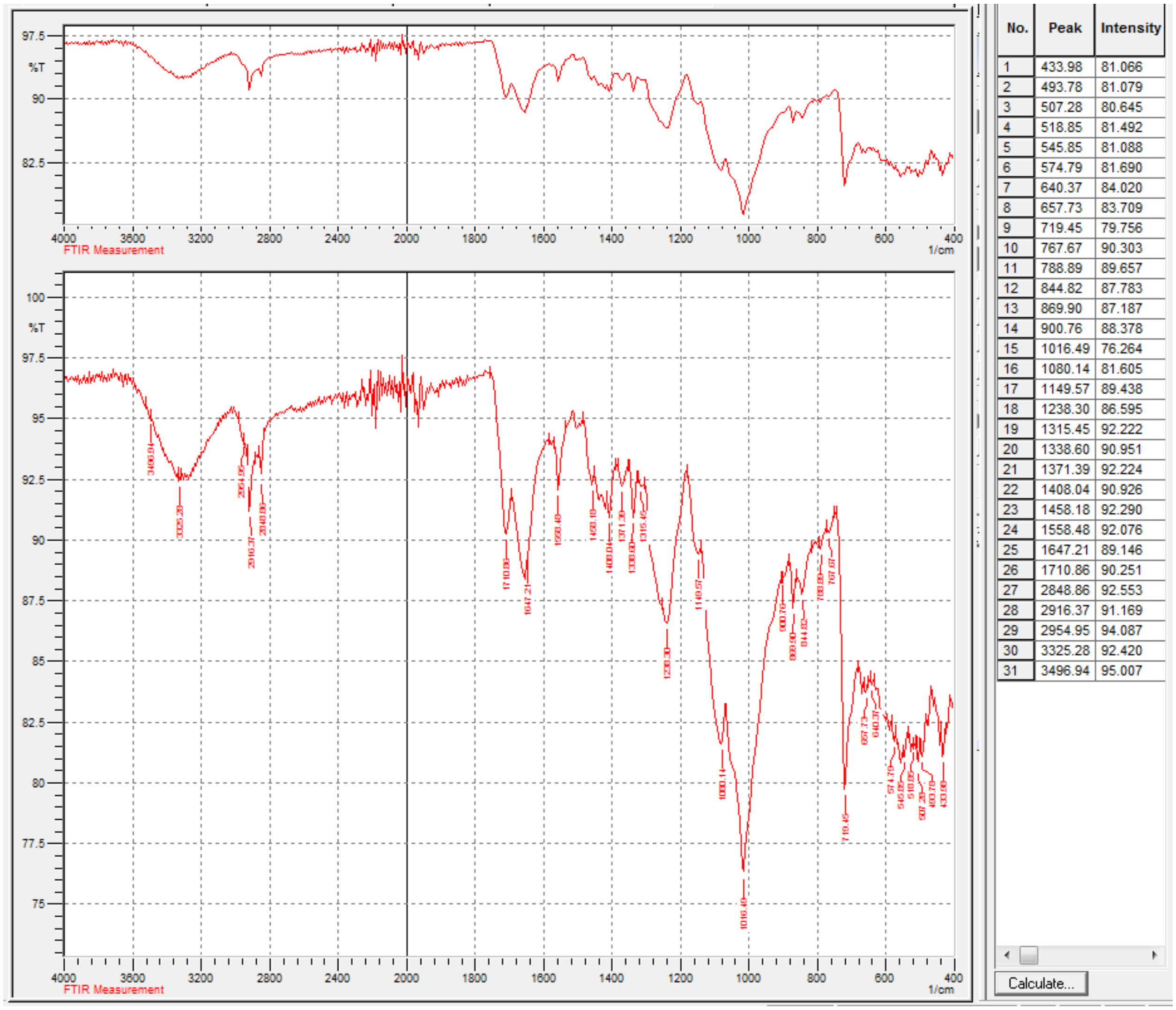

Fig. S-2.7.3. FTIR spectrum of RAN – Breathable portion.

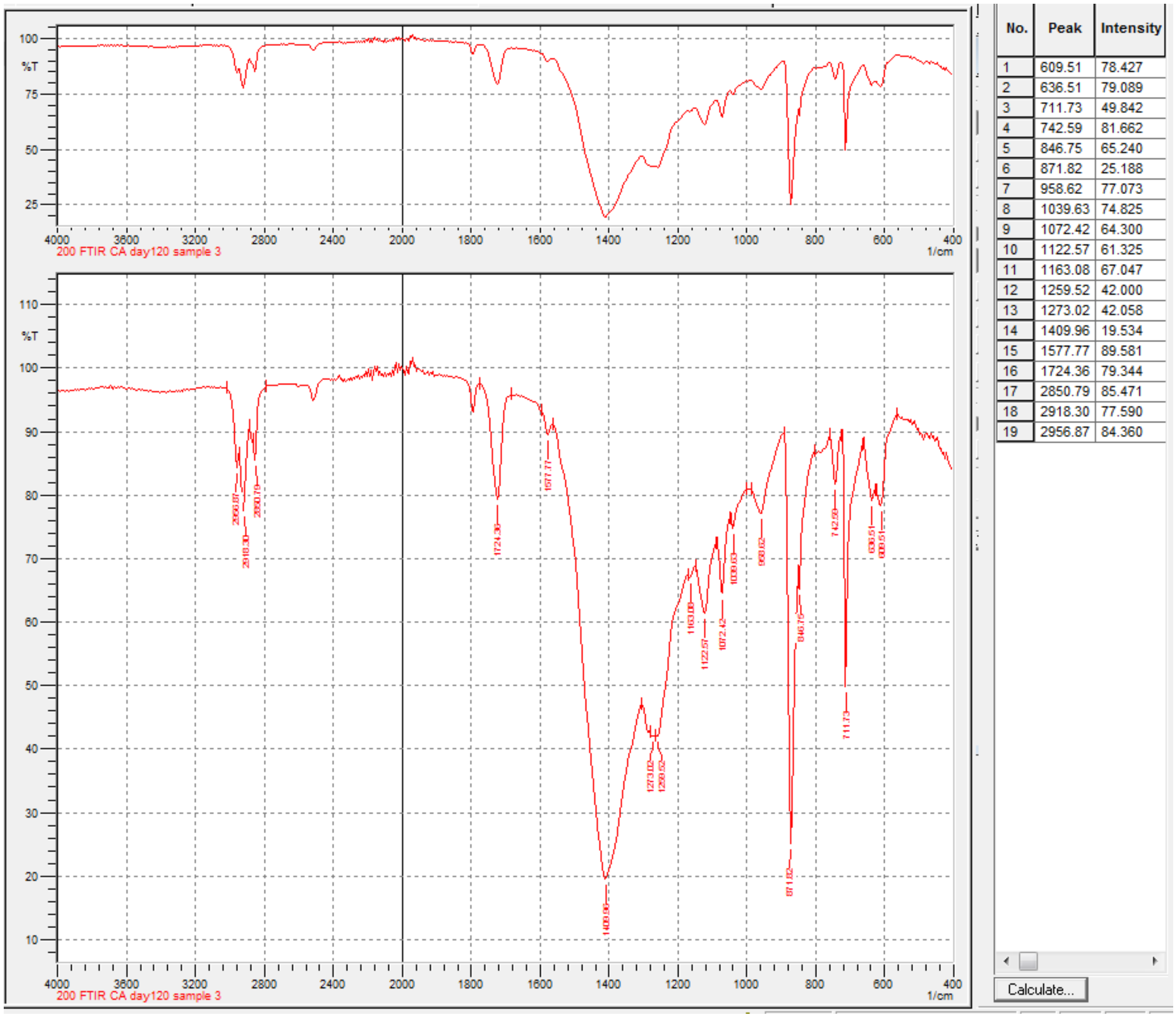

No.	Peak	Intensity
1	609.51	78.427
2	636.51	79.089
3	711.73	49.842
4	742.59	81.662
5	846.75	65.240
6	871.82	25.188
7	958.62	77.073
8	1039.63	74.825
9	1072.42	64.300
10	1122.57	61.325
11	1163.08	67.047
12	1259.52	42.000
13	1273.02	42.058
14	1409.96	19.534
15	1577.77	89.581
16	1724.36	79.344
17	2850.79	85.471
18	2918.30	77.590
19	2956.87	84.360

Calculate...

Fig. S-2.7.4. FTIR spectrum of RAN – Coating on nose bridge wire.

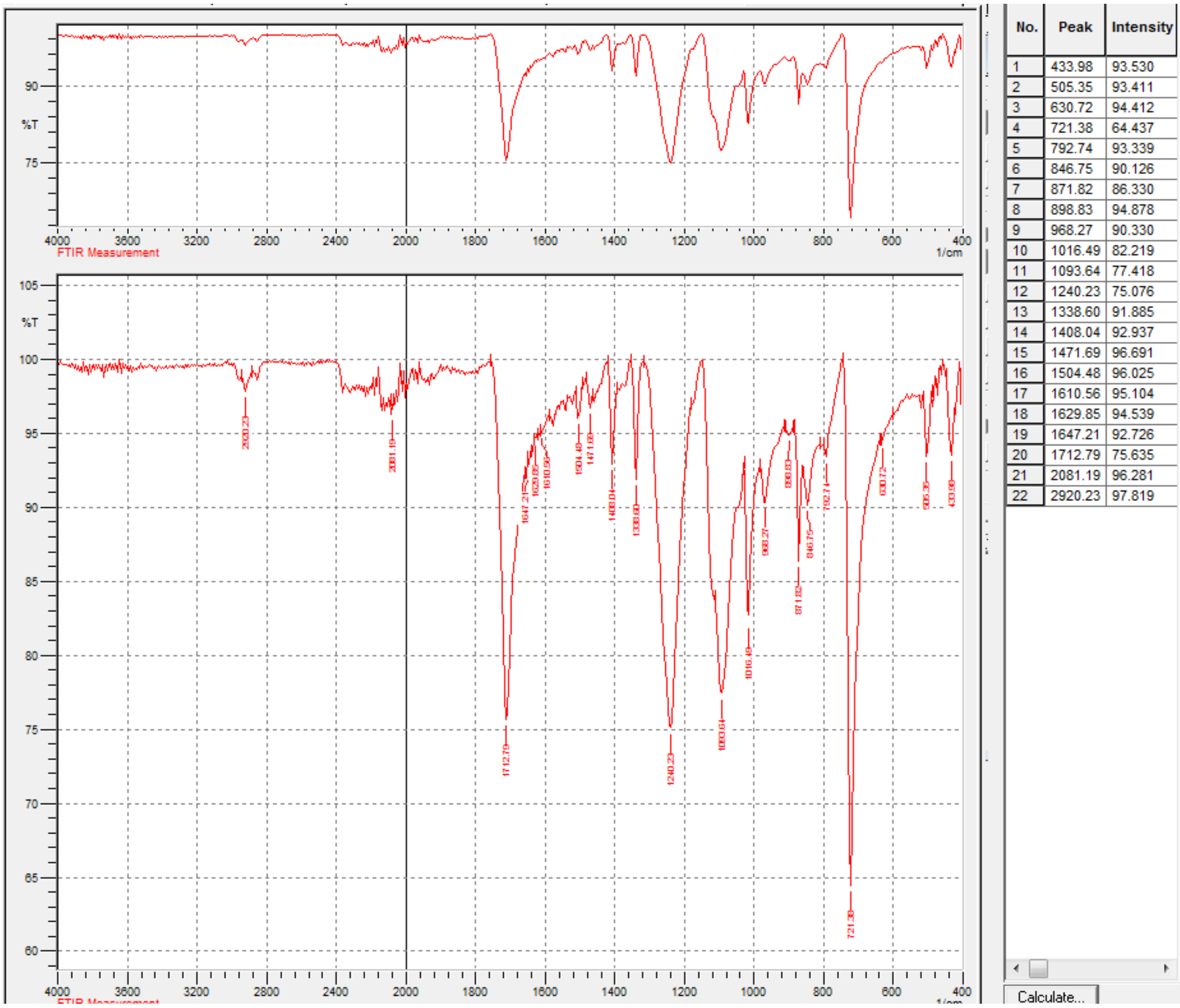

No.	Peak	Intensity
1	433.98	93.530
2	505.35	93.411
3	630.72	94.412
4	721.38	64.437
5	792.74	93.339
6	846.75	90.126
7	871.82	86.330
8	898.83	94.878
9	968.27	90.330
10	1016.49	82.219
11	1093.64	77.418
12	1240.23	75.076
13	1338.60	91.885
14	1408.04	92.937
15	1471.69	96.691
16	1504.48	96.025
17	1610.56	95.104
18	1629.85	94.539
19	1647.21	92.726
20	1712.79	75.635
21	2081.19	96.281
22	2920.23	97.819

Calculate...

Fig. S-2.7.5. FTIR spectrum of RAN – Ear loop.

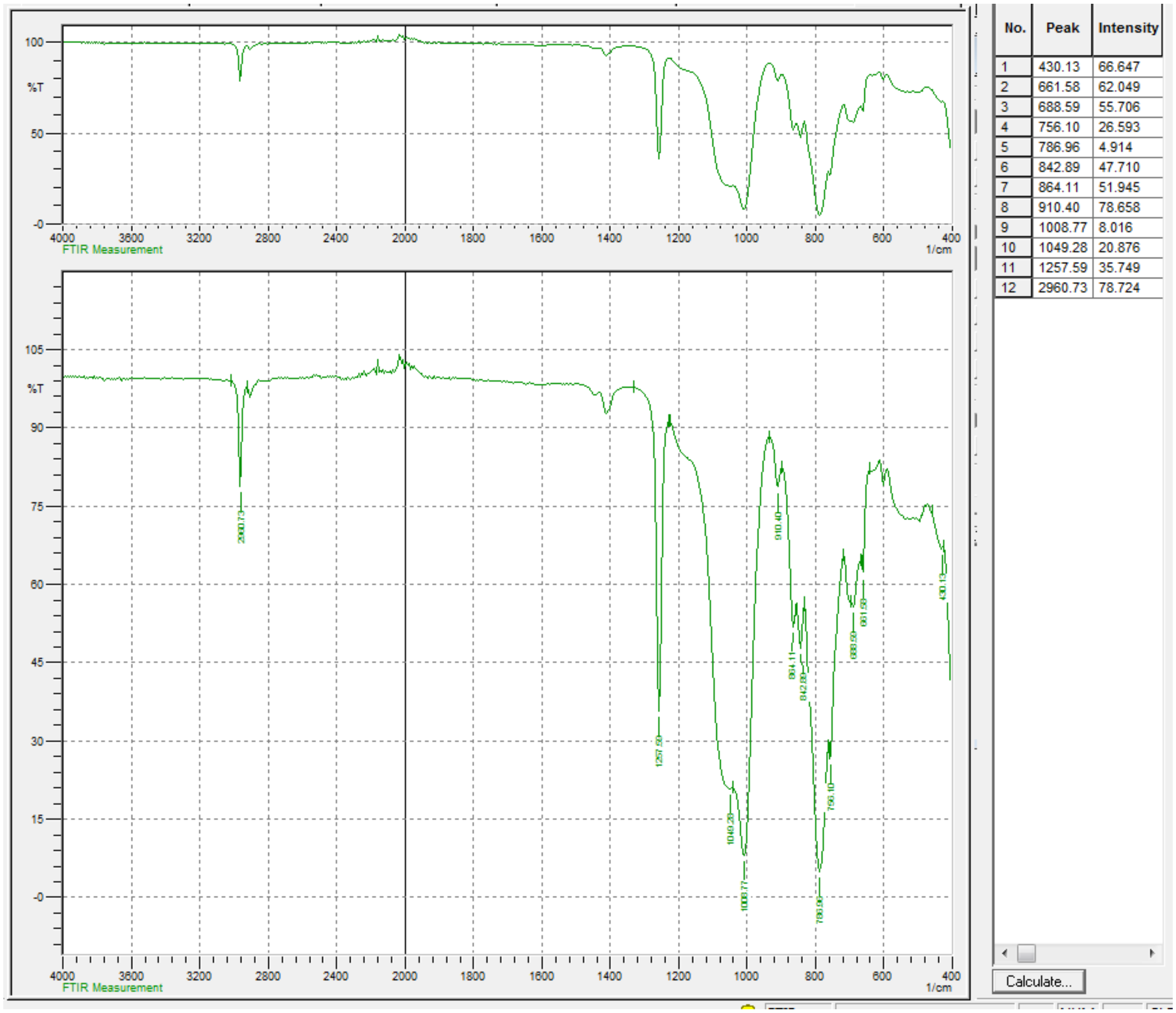

Fig. S-2.8.1. FTIR spectrum of SEU – Transparent portion, inside.

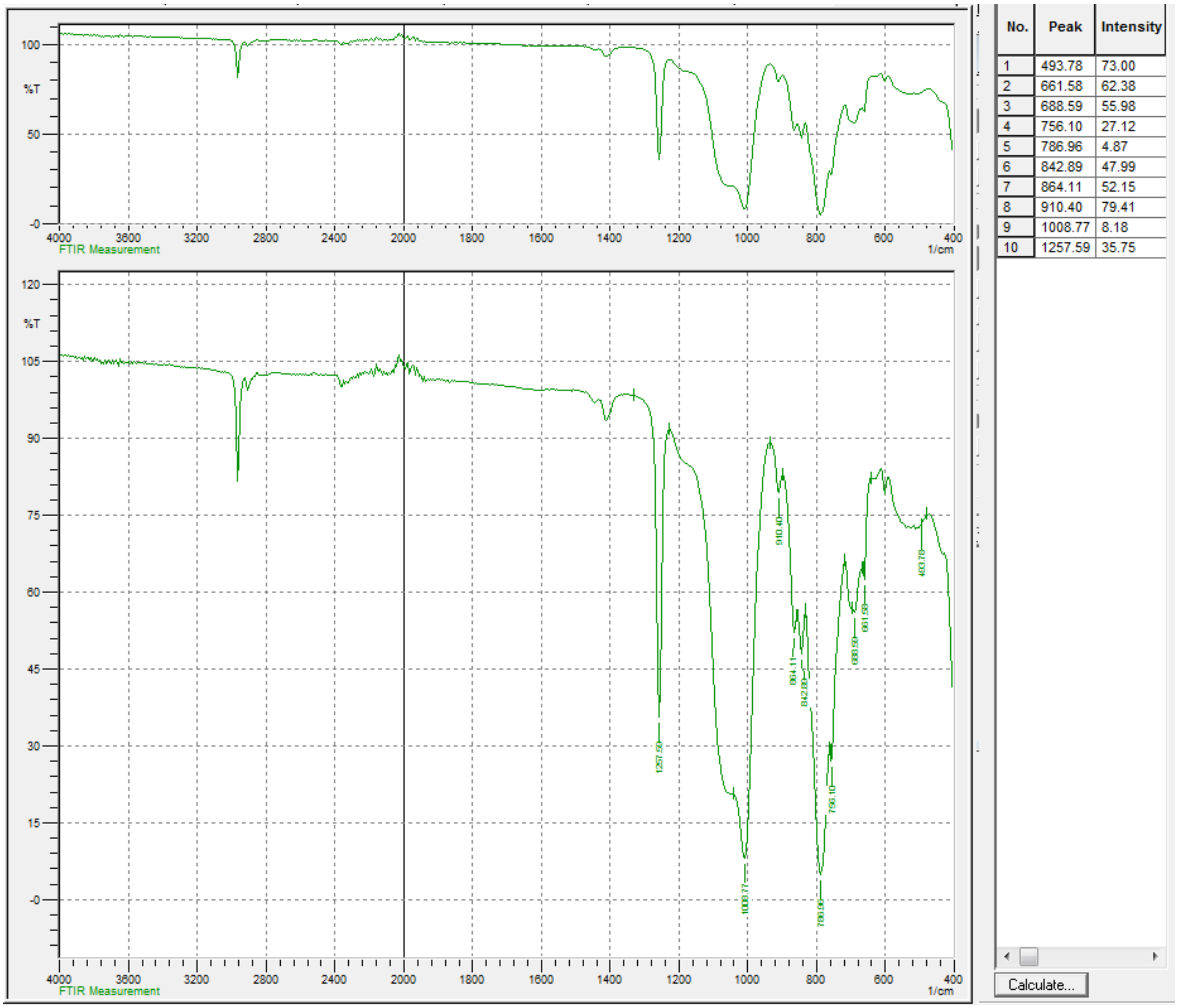

Fig. S-2.8.2. FTIR spectrum of SEU – Transparent portion, outside.

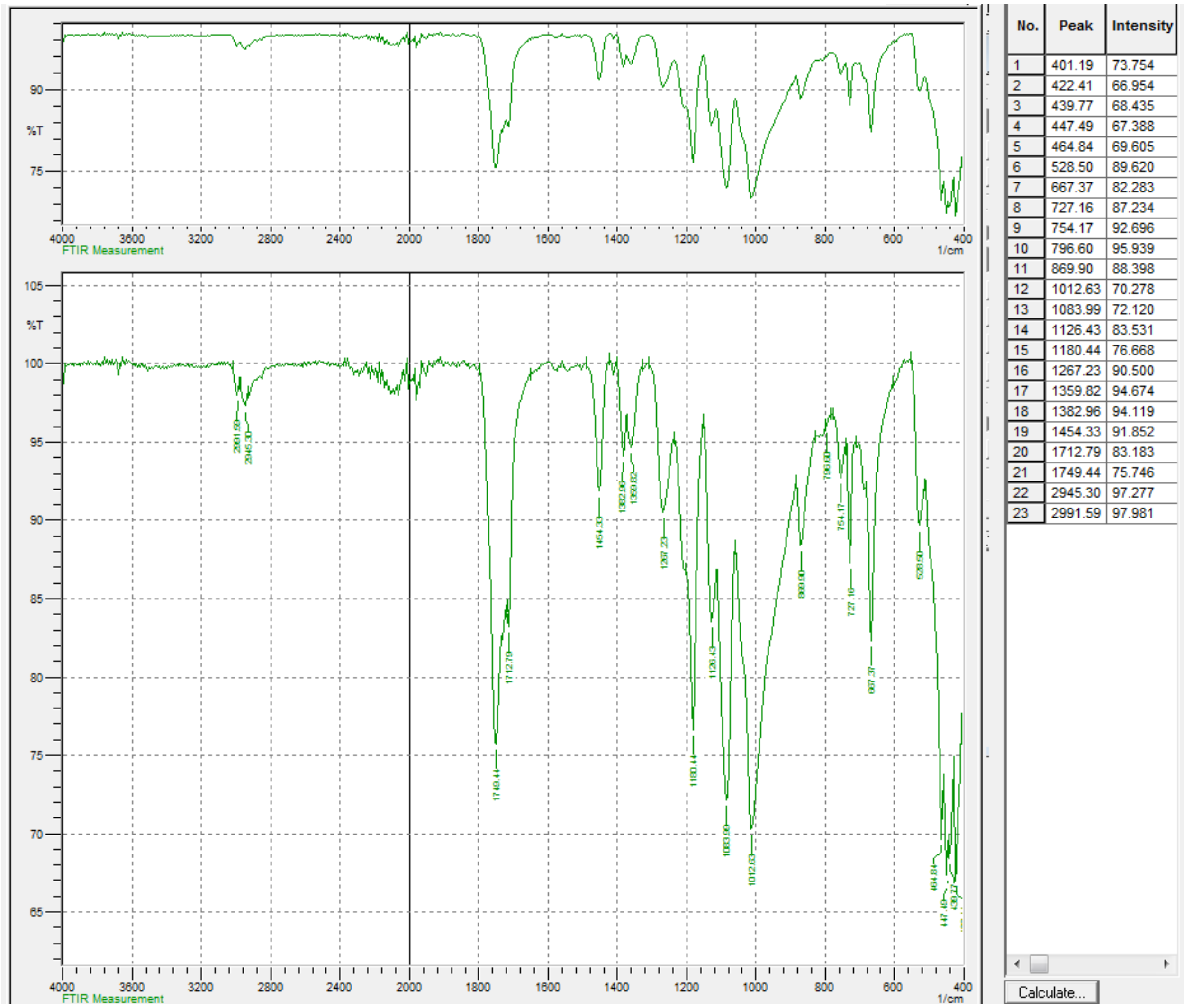

No.	Peak	Intensity
1	401.19	73.754
2	422.41	66.954
3	439.77	68.435
4	447.49	67.388
5	464.84	69.605
6	528.50	89.620
7	667.37	82.283
8	727.16	87.234
9	754.17	92.696
10	796.60	95.939
11	869.90	88.398
12	1012.63	70.278
13	1083.99	72.120
14	1126.43	83.531
15	1180.44	76.668
16	1267.23	90.500
17	1359.82	94.674
18	1382.96	94.119
19	1454.33	91.852
20	1712.79	83.183
21	1749.44	75.746
22	2945.30	97.277
23	2991.59	97.981

Fig. S-2.8.3. FTIR spectrum of SEU – Structural portion.

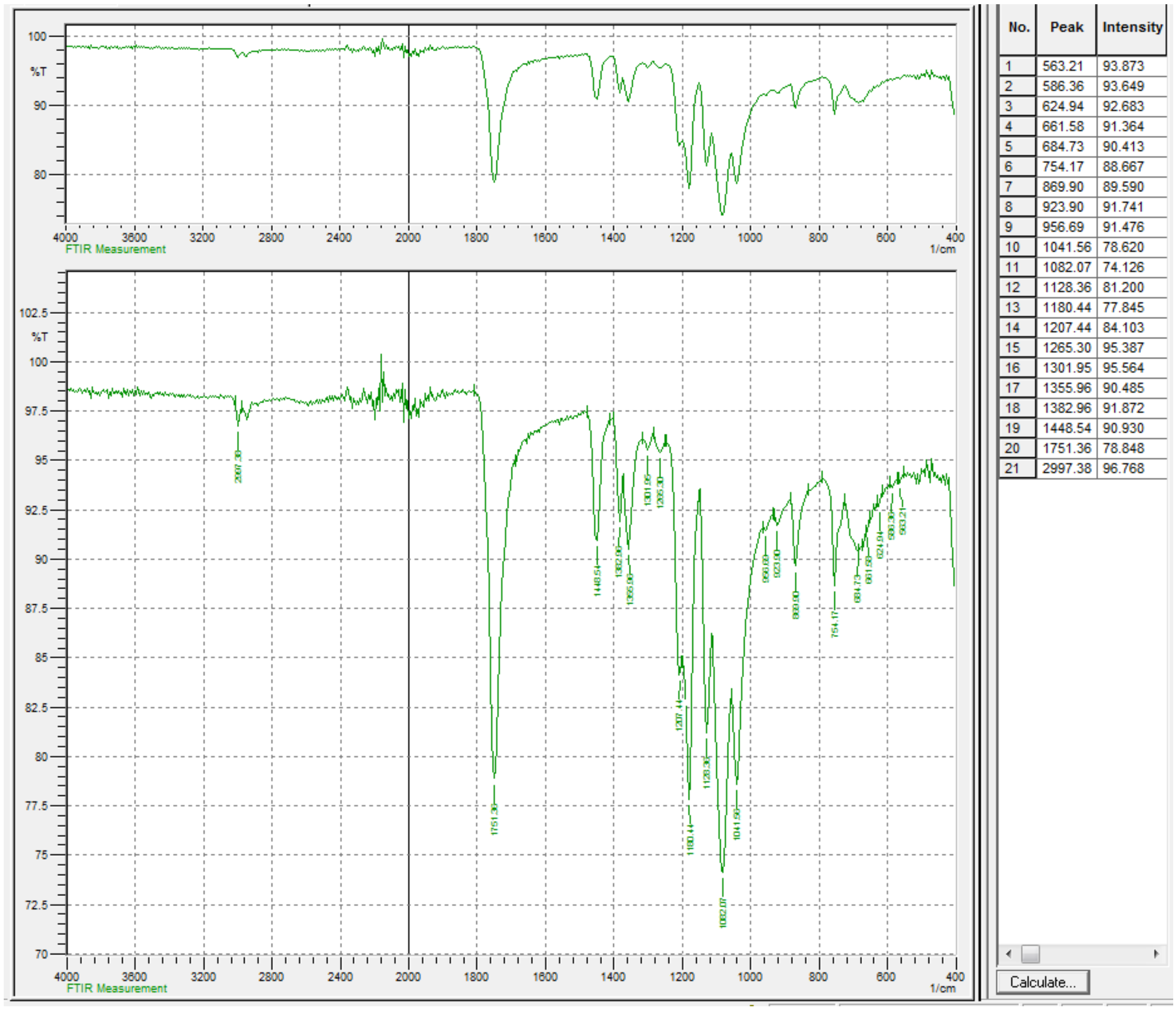

No.	Peak	Intensity
1	563.21	93.873
2	586.36	93.649
3	624.94	92.683
4	661.58	91.364
5	684.73	90.413
6	754.17	88.667
7	869.90	89.590
8	923.90	91.741
9	956.69	91.476
10	1041.56	78.620
11	1082.07	74.126
12	1128.36	81.200
13	1180.44	77.845
14	1207.44	84.103
15	1265.30	95.387
16	1301.95	95.564
17	1355.96	90.485
18	1382.96	91.872
19	1448.54	90.930
20	1751.36	78.848
21	2997.38	96.768

Calculate...

Fig. S-2.8.4. FTIR spectrum of SEU – Filter.

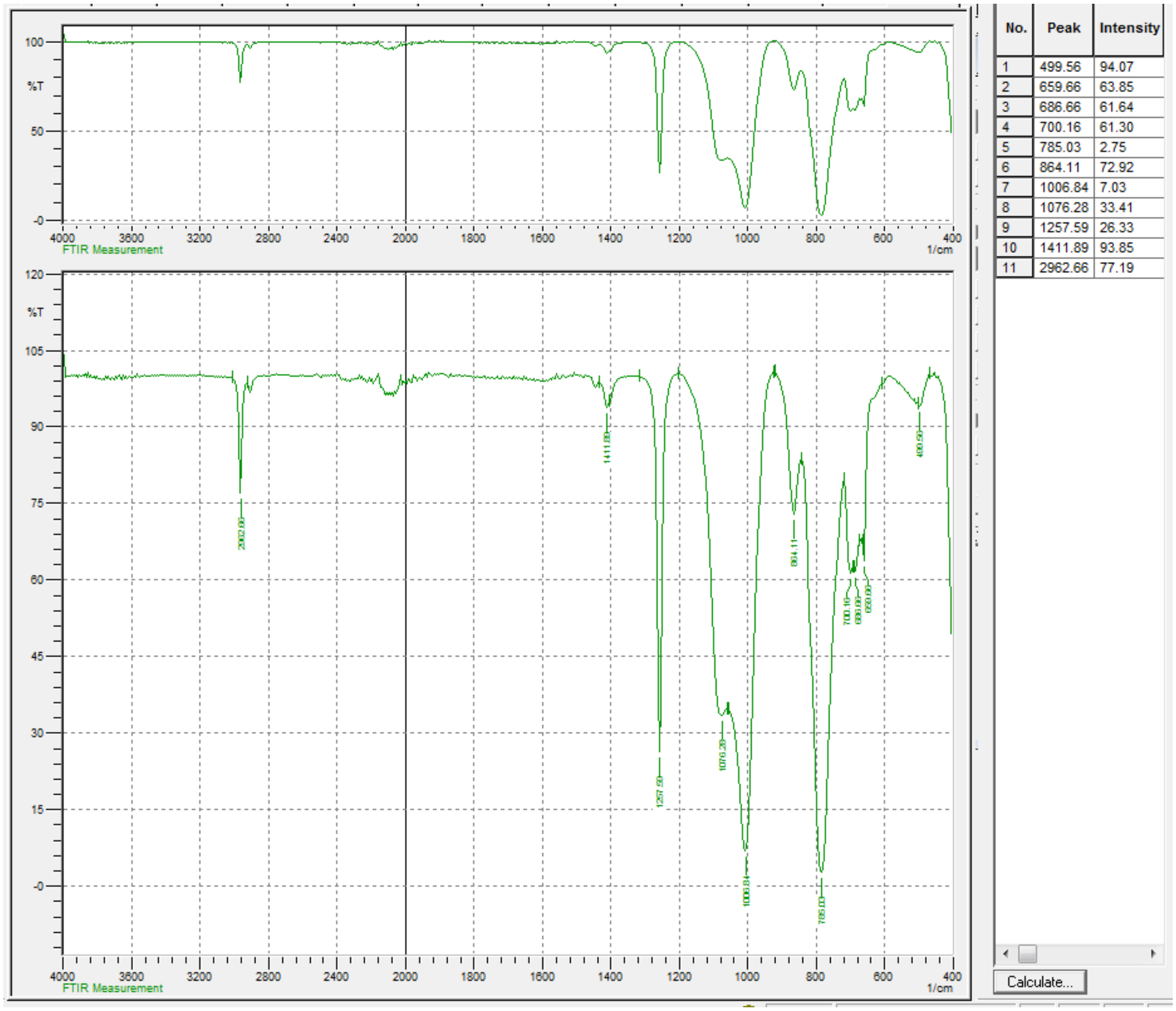

No.	Peak	Intensity
1	499.56	94.07
2	659.66	63.85
3	686.66	61.64
4	700.16	61.30
5	785.03	2.75
6	864.11	72.92
7	1006.84	7.03
8	1076.28	33.41
9	1257.59	26.33
10	1411.89	93.85
11	2962.66	77.19

Fig. S-2.8.5. FTIR spectrum of SEU – Adhesive.

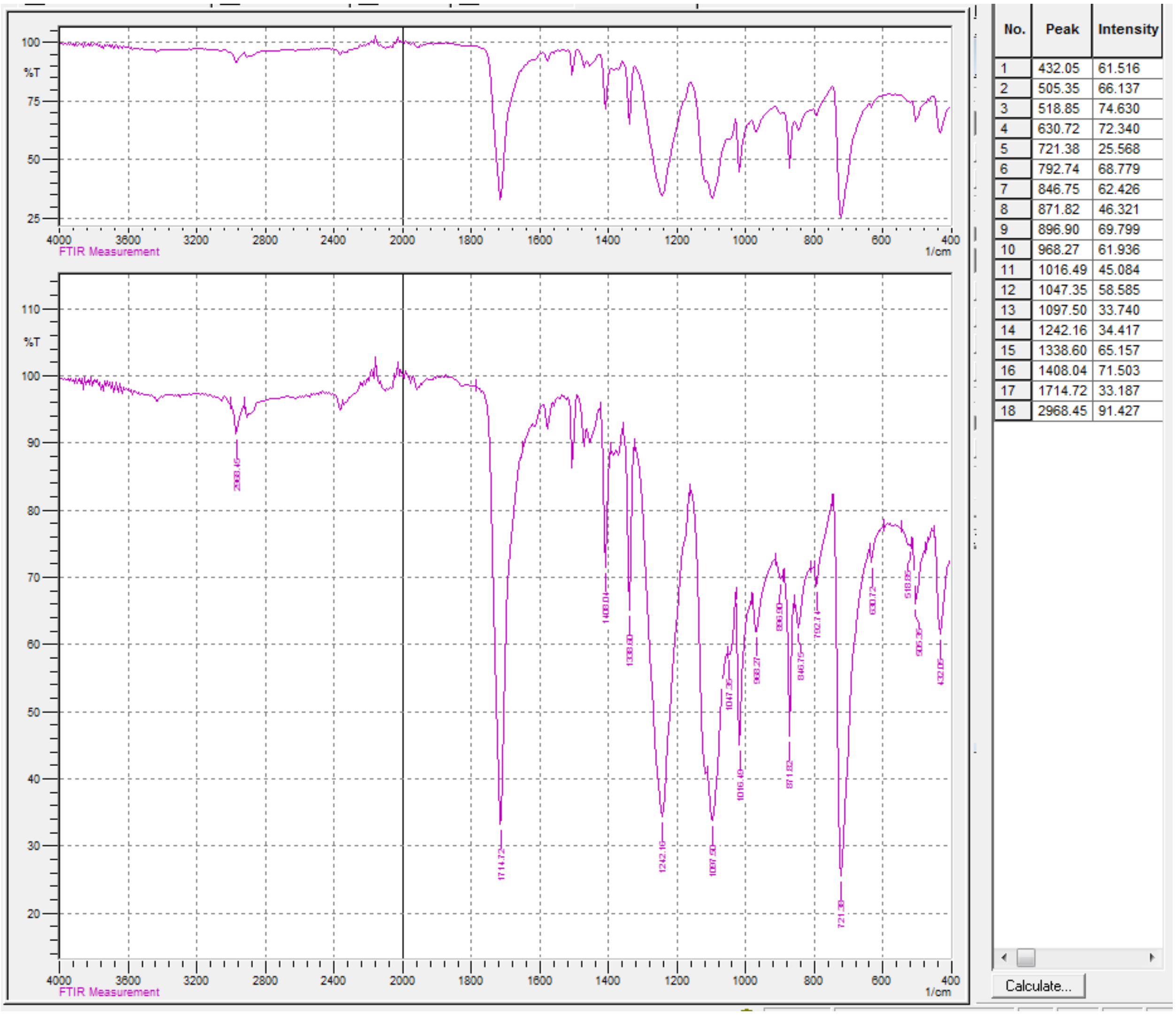

No.	Peak	Intensity
1	432.05	61.516
2	505.35	66.137
3	518.85	74.630
4	630.72	72.340
5	721.38	25.568
6	792.74	68.779
7	846.75	62.426
8	871.82	46.321
9	896.90	69.799
10	968.27	61.936
11	1016.49	45.084
12	1047.35	58.585
13	1097.50	33.740
14	1242.16	34.417
15	1338.60	65.157
16	1408.04	71.503
17	1714.72	33.187
18	2968.45	91.427

Fig. S-2.9.1. FTIR spectrum of SNC – Transparent portion, inside.

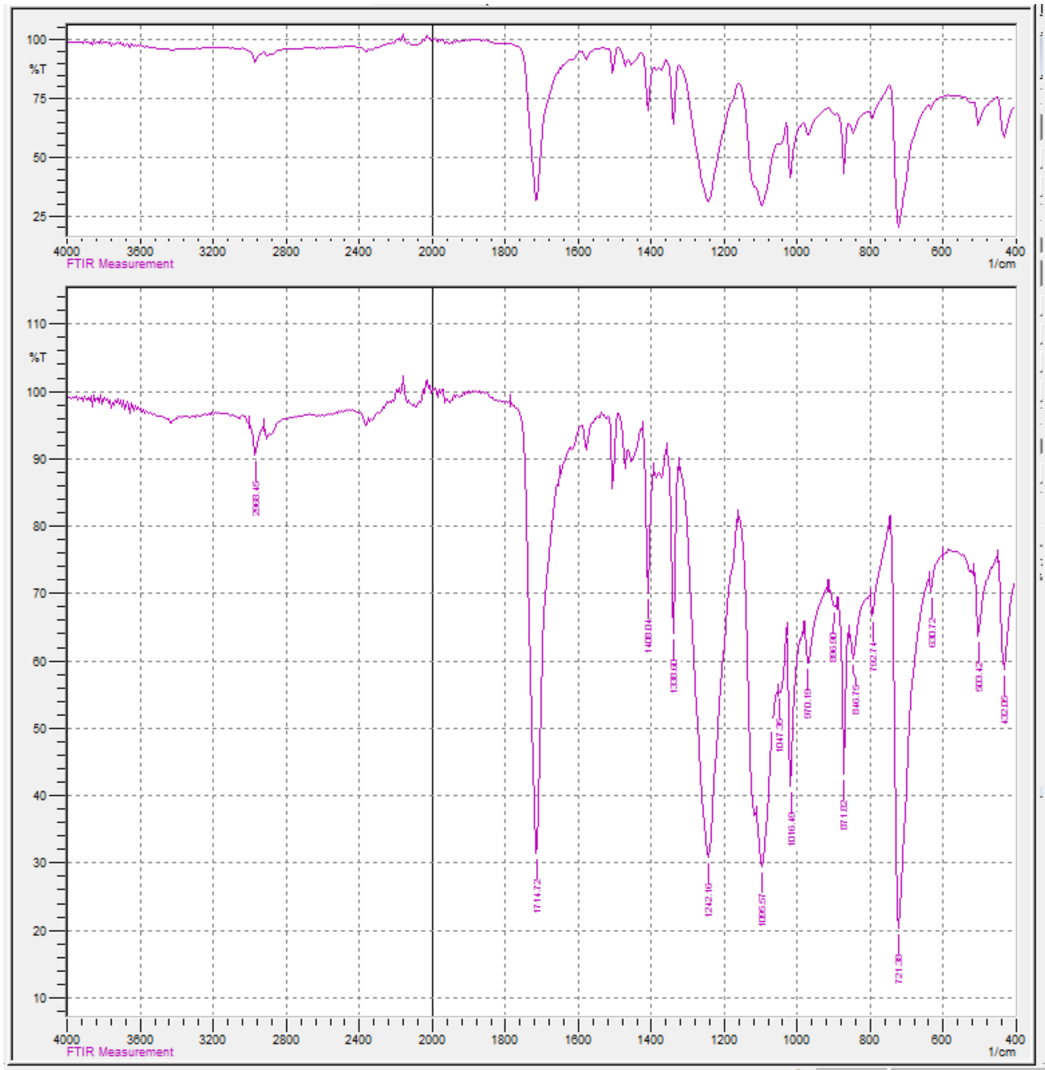

No.	Peak	Intensity
1	432.05	58.690
2	503.42	63.791
3	630.72	70.282
4	721.38	20.309
5	792.74	66.651
6	846.75	60.276
7	871.82	43.241
8	896.90	68.053
9	970.19	59.797
10	1016.49	41.446
11	1047.35	55.336
12	1095.57	29.509
13	1242.16	30.865
14	1338.60	64.089
15	1408.04	70.053
16	1714.72	31.409
17	2968.45	90.617

Calculate...

Fig. S-2.9.2. FTIR spectrum of SNC – Transparent portion, outside.

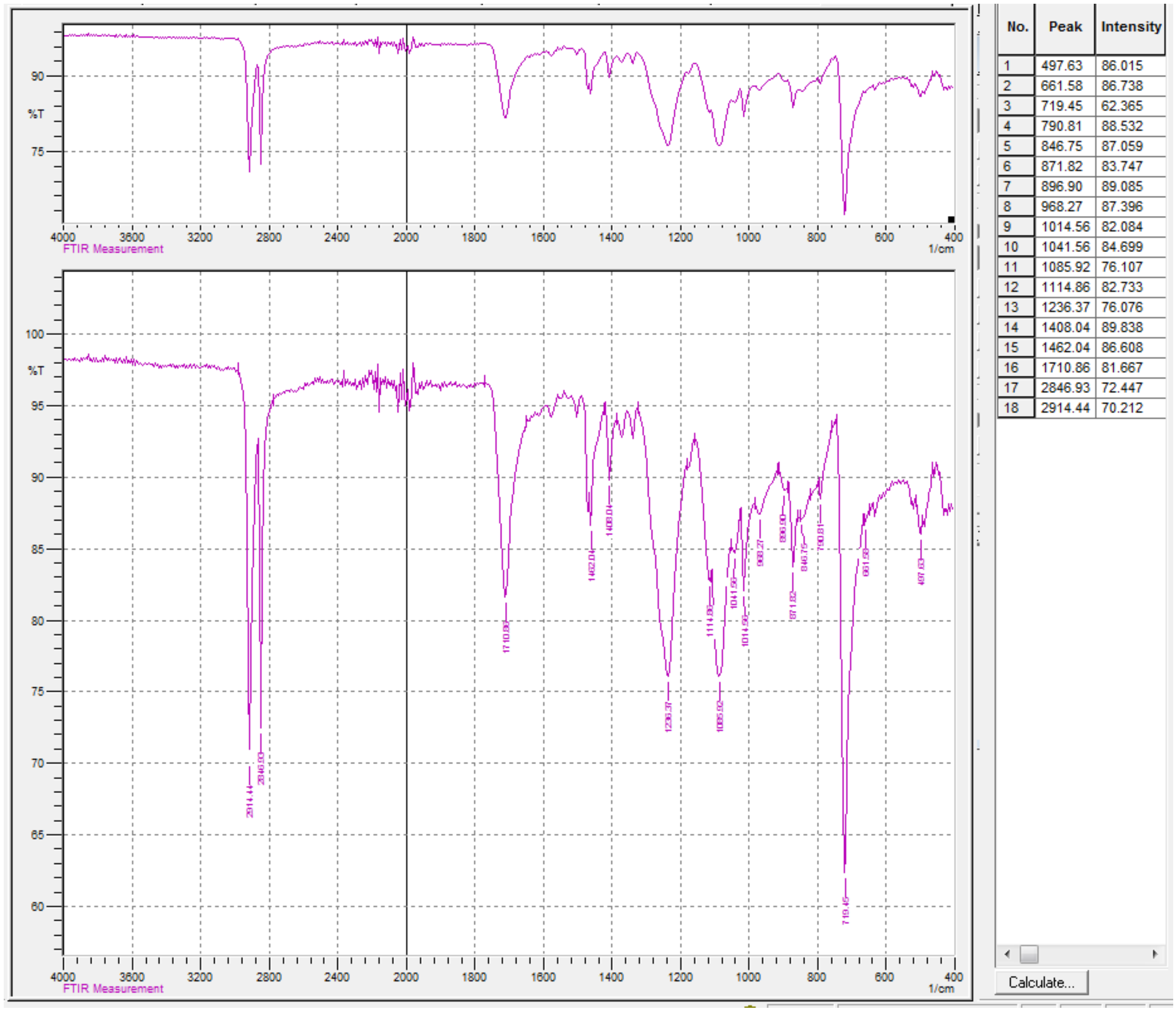

No.	Peak	Intensity
1	497.63	86.015
2	661.58	86.738
3	719.45	62.365
4	790.81	88.532
5	846.75	87.059
6	871.82	83.747
7	896.90	89.085
8	968.27	87.396
9	1014.56	82.084
10	1041.56	84.699
11	1085.92	76.107
12	1114.86	82.733
13	1236.37	76.076
14	1408.04	89.838
15	1462.04	86.608
16	1710.86	81.667
17	2846.93	72.447
18	2914.44	70.212

Calculate...

Fig. S-2.9.3. FTIR spectrum of SNC – Breathable portion.

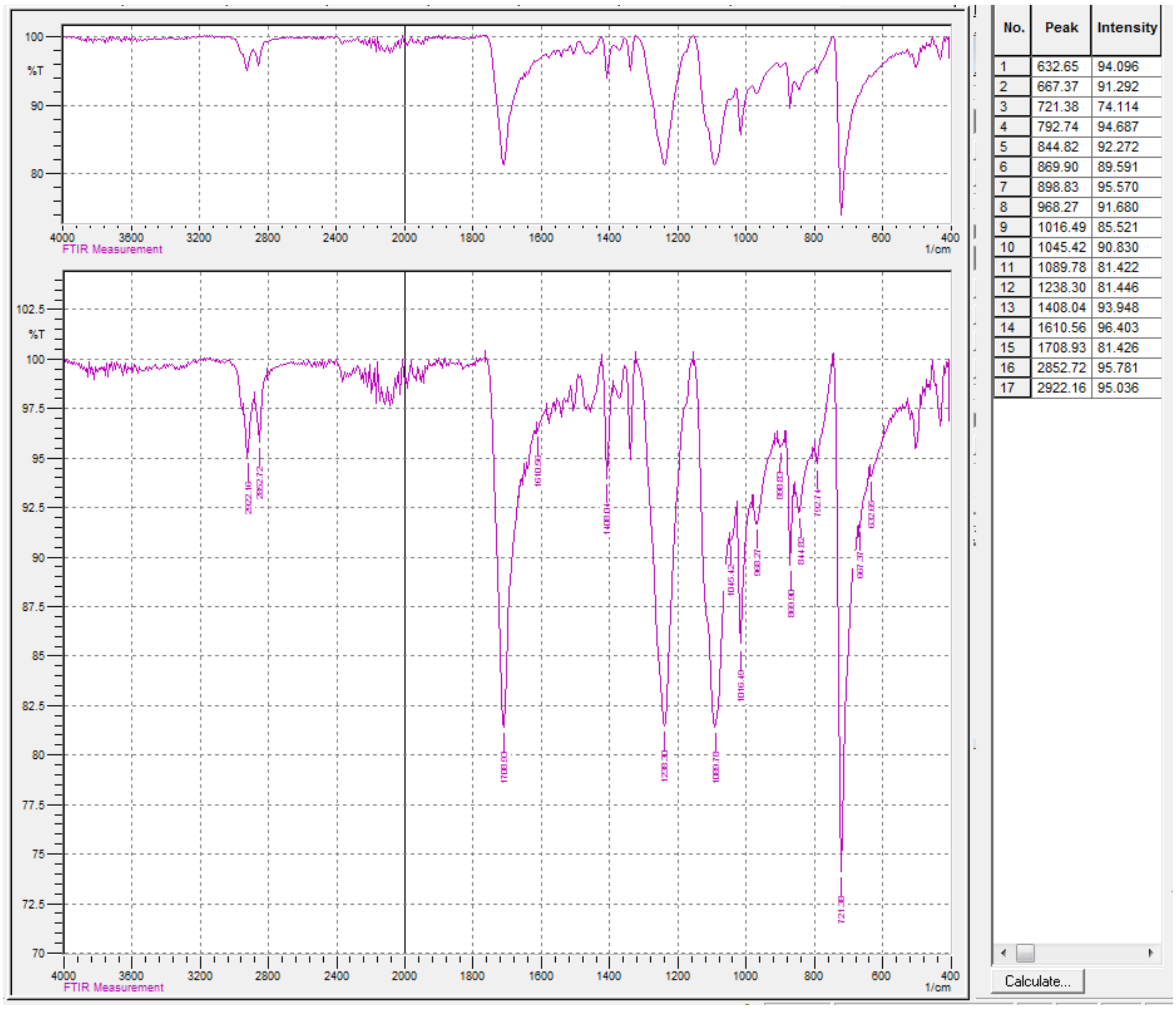

Fig. S-2.9.4. FTIR spectrum of SNC – Ear loop.

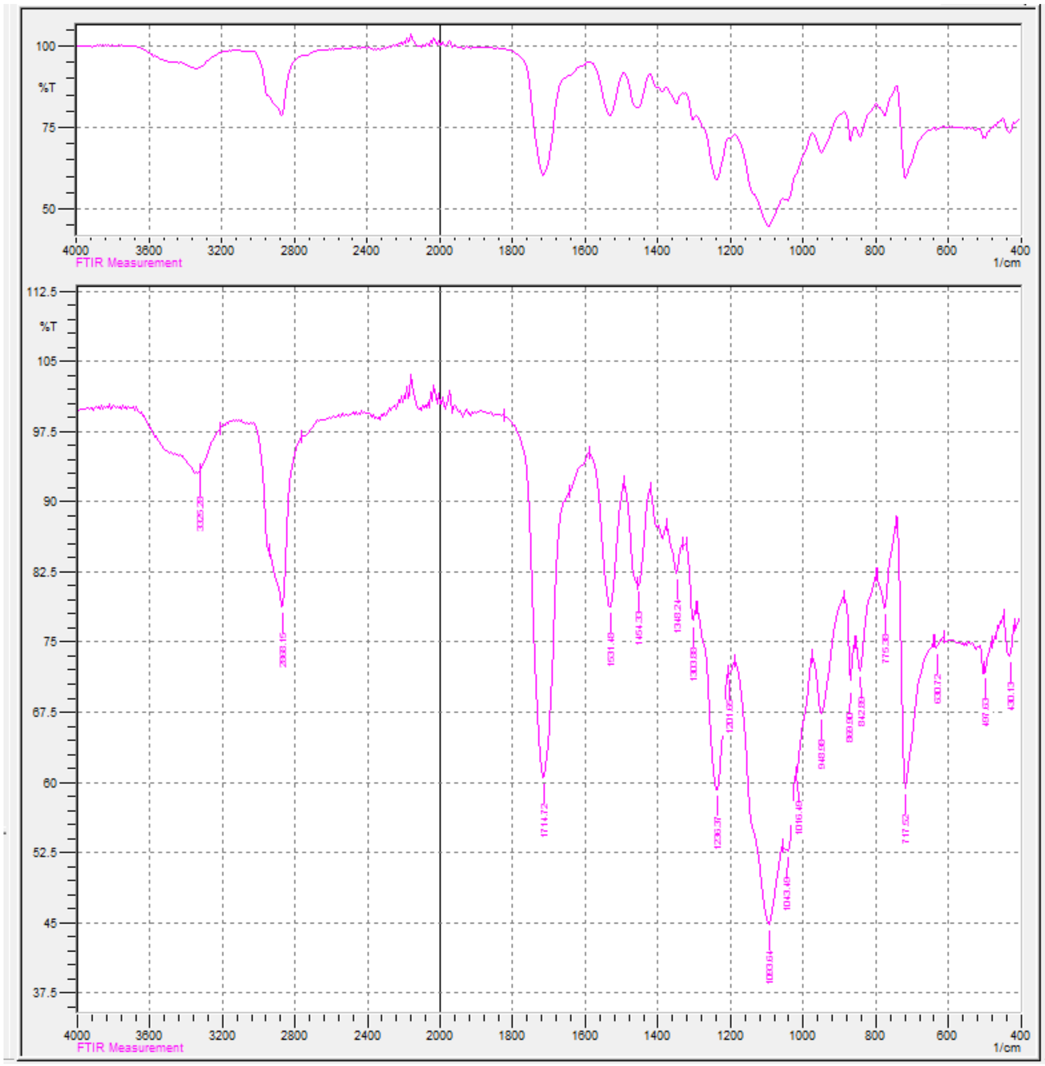

No.	Peak	Intensity
1	430.13	73.434
2	497.63	71.743
3	630.72	74.412
4	717.52	59.402
5	775.38	78.610
6	842.89	71.980
7	869.90	70.220
8	948.98	67.307
9	1016.49	60.859
10	1043.49	52.660
11	1093.64	44.785
12	1201.65	71.679
13	1236.37	59.169
14	1303.88	77.274
15	1348.24	82.373
16	1454.33	81.017
17	1531.48	78.723
18	1714.72	60.443
19	2868.15	78.779
20	3325.28	93.286

Calculate...

Fig. S-2.10.1. FTIR spectrum of STK – Transparent portion, inside.

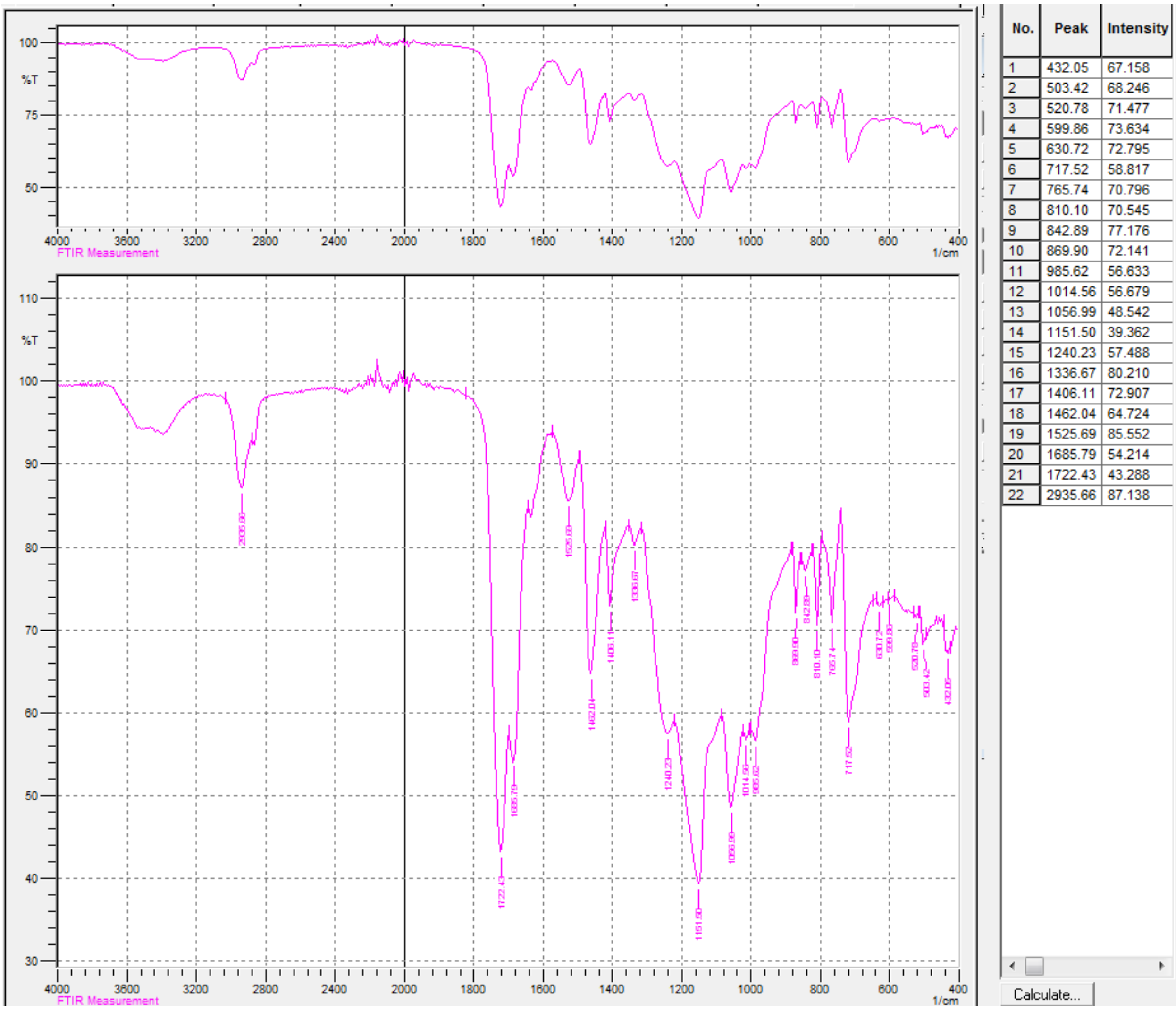

No.	Peak	Intensity
1	432.05	67.158
2	503.42	68.246
3	520.78	71.477
4	599.86	73.634
5	630.72	72.795
6	717.52	58.817
7	765.74	70.796
8	810.10	70.545
9	842.89	77.176
10	869.90	72.141
11	985.62	56.633
12	1014.56	56.679
13	1056.99	48.542
14	1151.50	39.362
15	1240.23	57.488
16	1336.67	80.210
17	1406.11	72.907
18	1462.04	64.724
19	1525.69	85.552
20	1685.79	54.214
21	1722.43	43.288
22	2935.66	87.138

Calculate...

Fig. S-2.10.2. FTIR spectrum of STK – Transparent portion, outside.

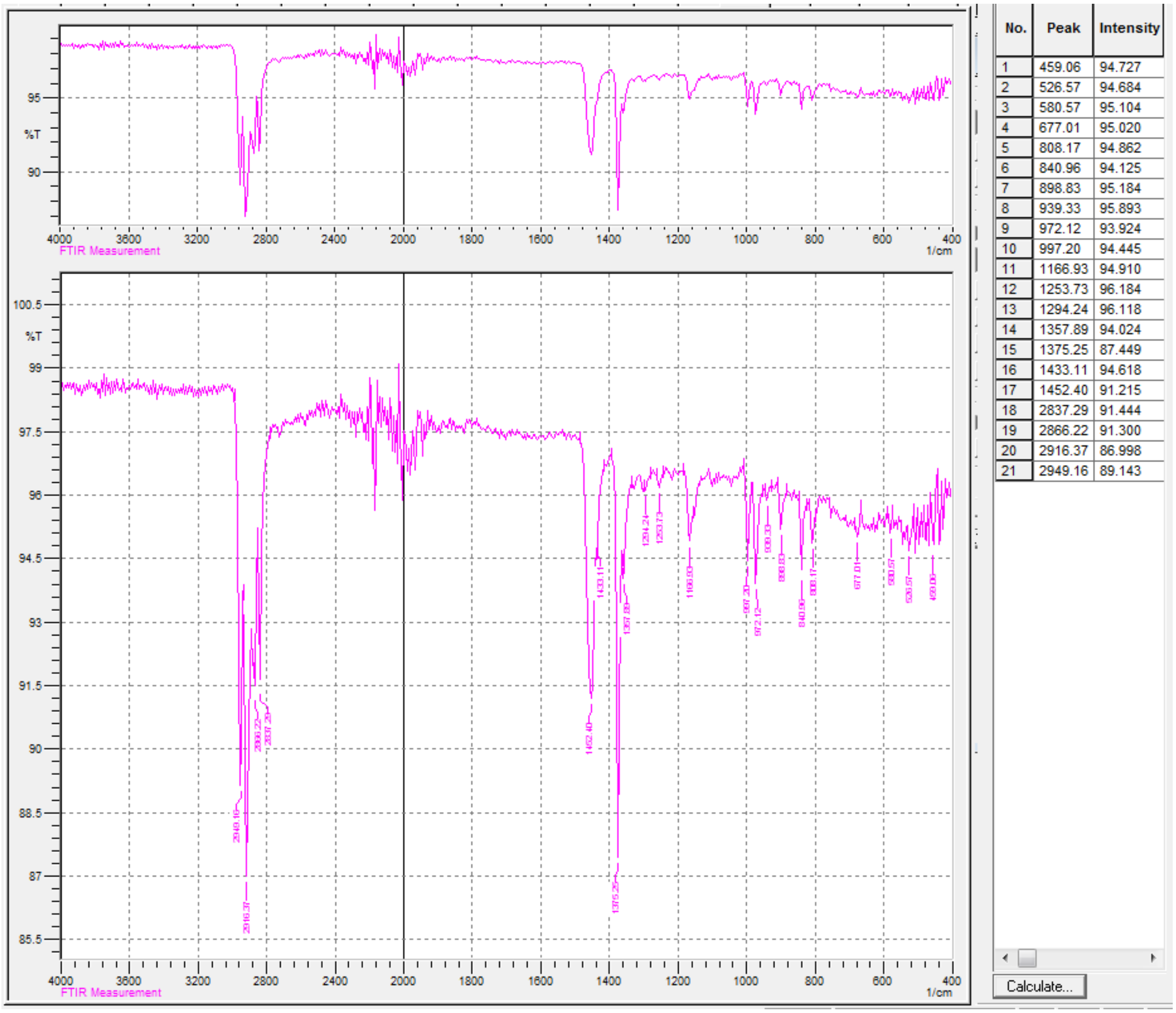

No.	Peak	Intensity
1	459.06	94.727
2	526.57	94.684
3	580.57	95.104
4	677.01	95.020
5	808.17	94.862
6	840.96	94.125
7	898.83	95.184
8	939.33	95.893
9	972.12	93.924
10	997.20	94.445
11	1166.93	94.910
12	1253.73	96.184
13	1294.24	96.118
14	1357.89	94.024
15	1375.25	87.449
16	1433.11	94.618
17	1452.40	91.215
18	2837.29	91.444
19	2866.22	91.300
20	2916.37	86.998
21	2949.16	89.143

Fig. S-2.10.3. FTIR spectrum STK – Breathable portion.

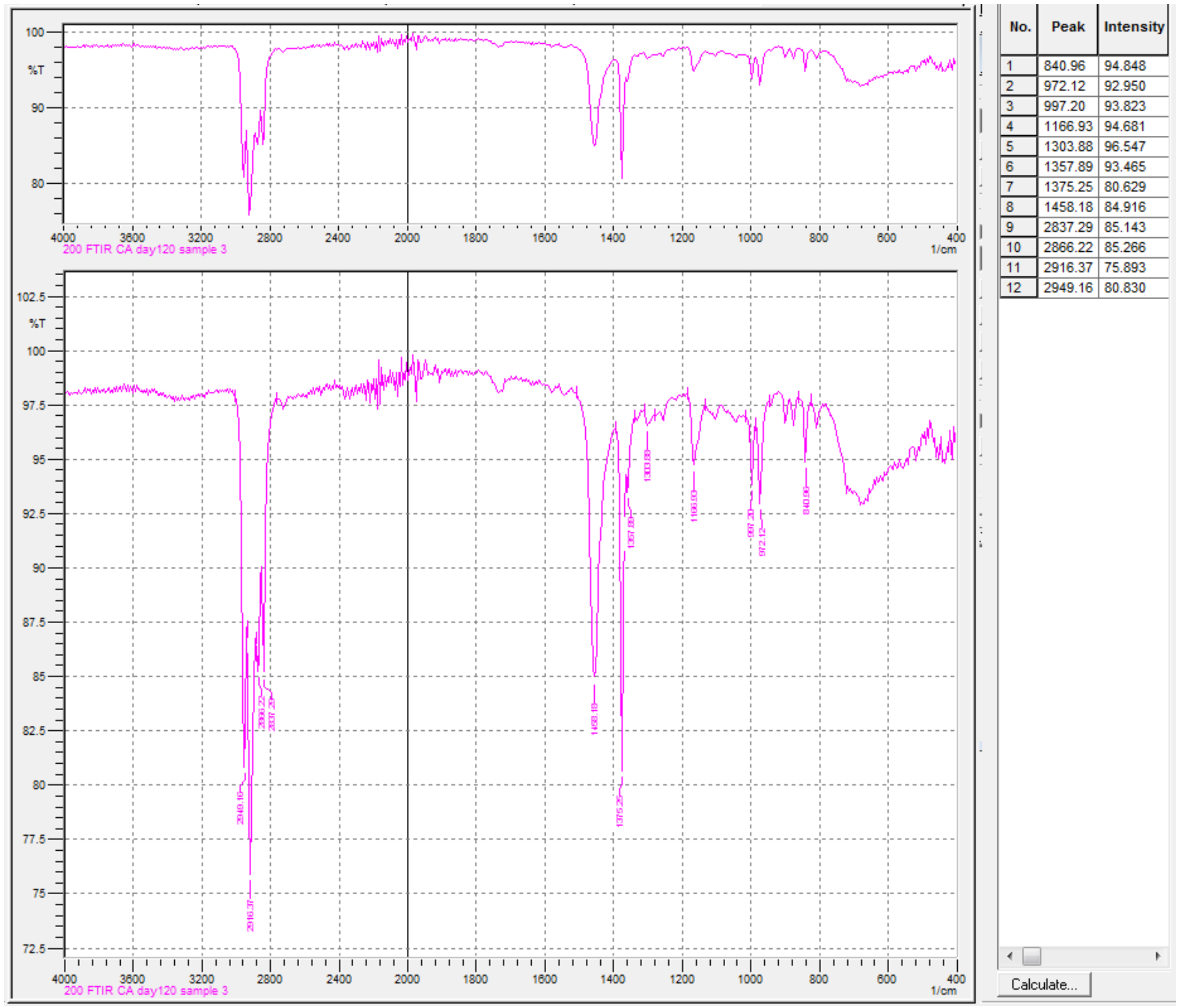

No.	Peak	Intensity
1	840.96	94.848
2	972.12	92.950
3	997.20	93.823
4	1166.93	94.681
5	1303.88	96.547
6	1357.89	93.465
7	1375.25	80.629
8	1458.18	84.916
9	2837.29	85.143
10	2866.22	85.266
11	2916.37	75.893
12	2949.16	80.830

Calculate...

Fig. S-2.10.4. FTIR spectrum STK – Coating on nose bridge wire.

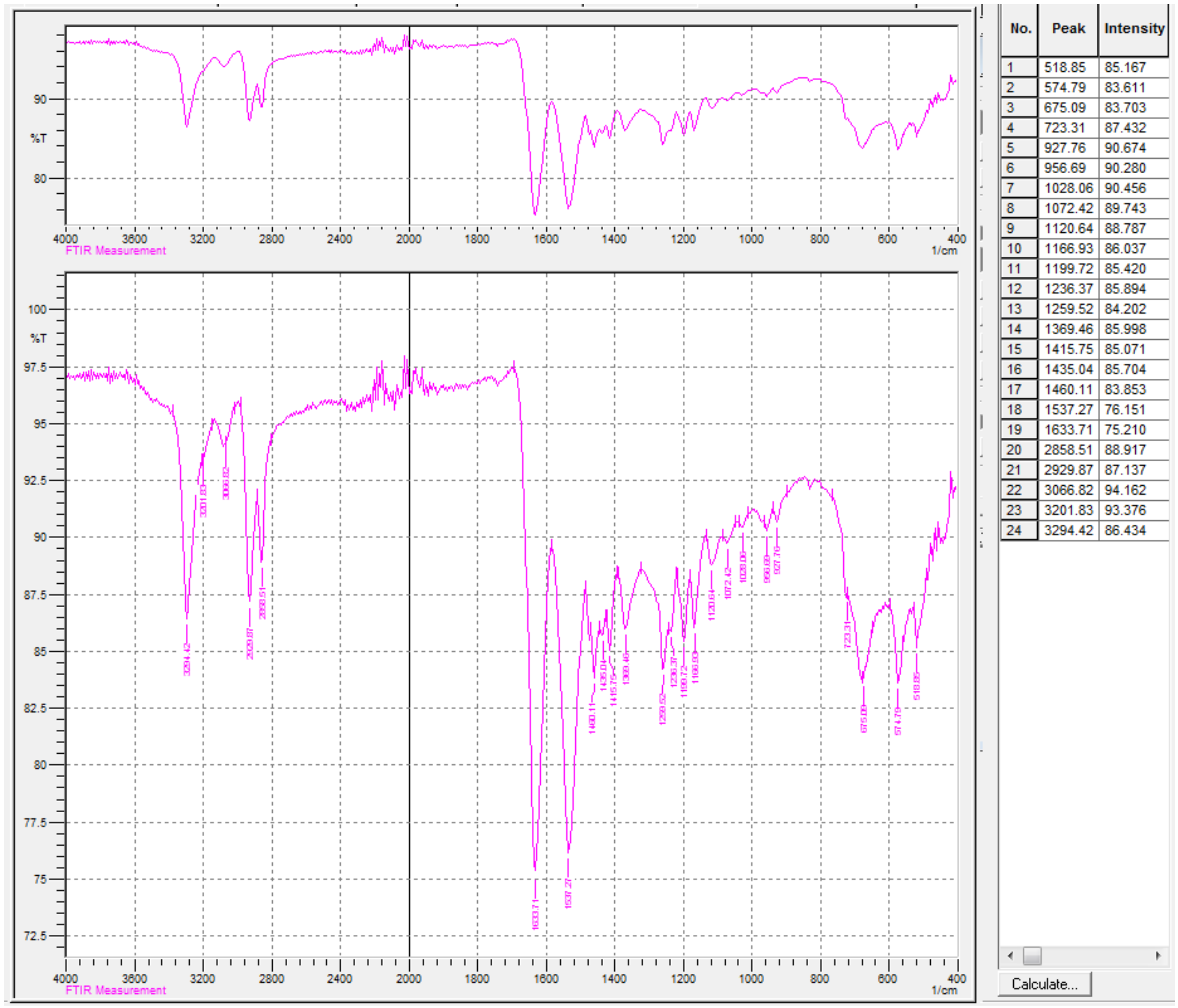

No.	Peak	Intensity
1	518.85	85.167
2	574.79	83.611
3	675.09	83.703
4	723.31	87.432
5	927.76	90.674
6	956.69	90.280
7	1028.06	90.456
8	1072.42	89.743
9	1120.64	88.787
10	1166.93	86.037
11	1199.72	85.420
12	1236.37	85.894
13	1259.52	84.202
14	1369.46	85.998
15	1415.75	85.071
16	1435.04	85.704
17	1460.11	83.853
18	1537.27	76.151
19	1633.71	75.210
20	2858.51	88.917
21	2929.87	87.137
22	3066.82	94.162
23	3201.83	93.376
24	3294.42	86.434

Calculate...

Fig. S-2.10.5. FTIR spectrum of STK – Ear loop.

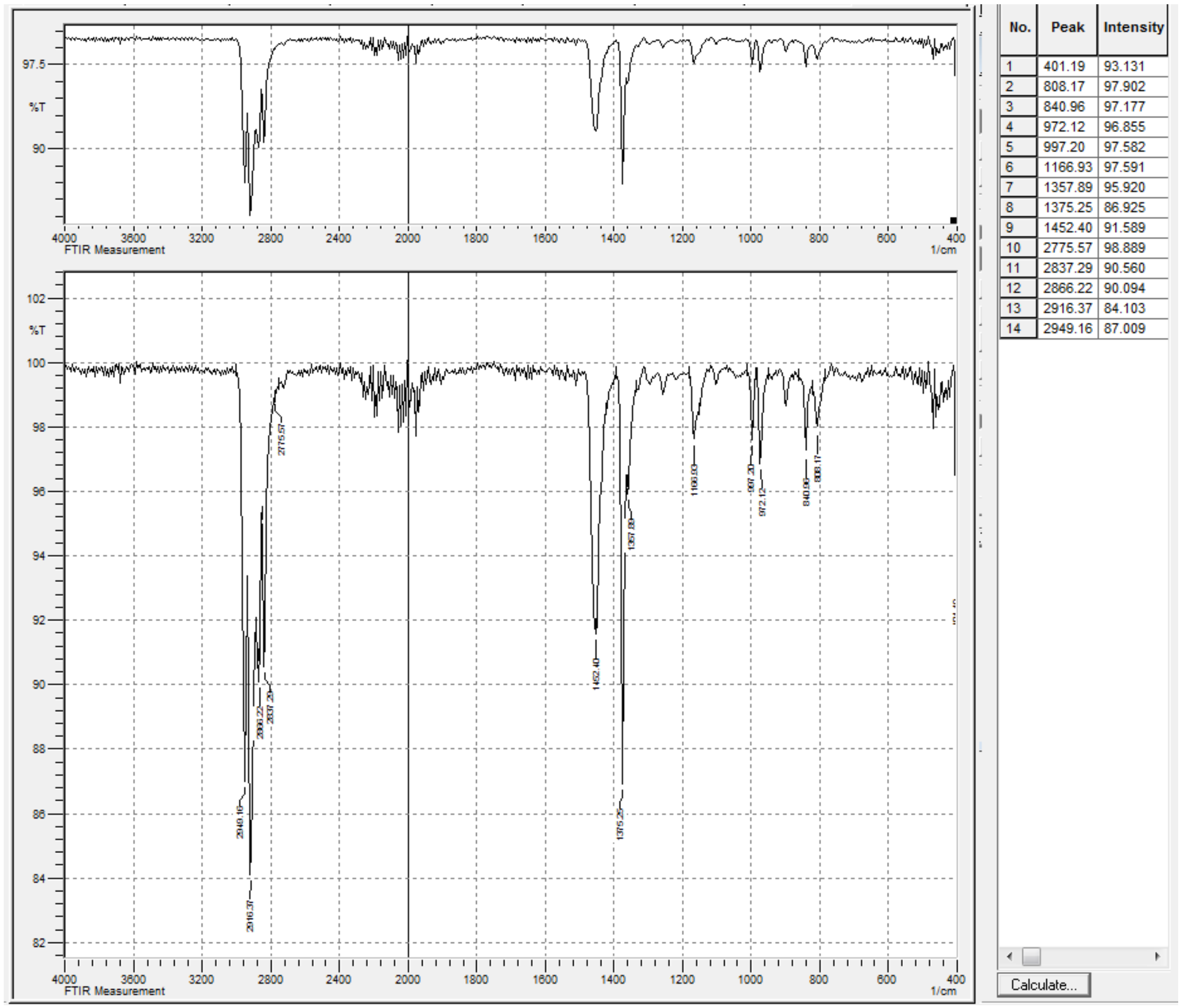

No.	Peak	Intensity
1	401.19	93.131
2	808.17	97.902
3	840.96	97.177
4	972.12	96.855
5	997.20	97.582
6	1166.93	97.591
7	1357.89	95.920
8	1375.25	86.925
9	1452.40	91.589
10	2775.57	98.889
11	2837.29	90.560
12	2866.22	90.094
13	2916.37	84.103
14	2949.16	87.009

Calculate...

Fig. S-2.11. FTIR spectrum of PHG (main, breathable portion of mask).

FAV

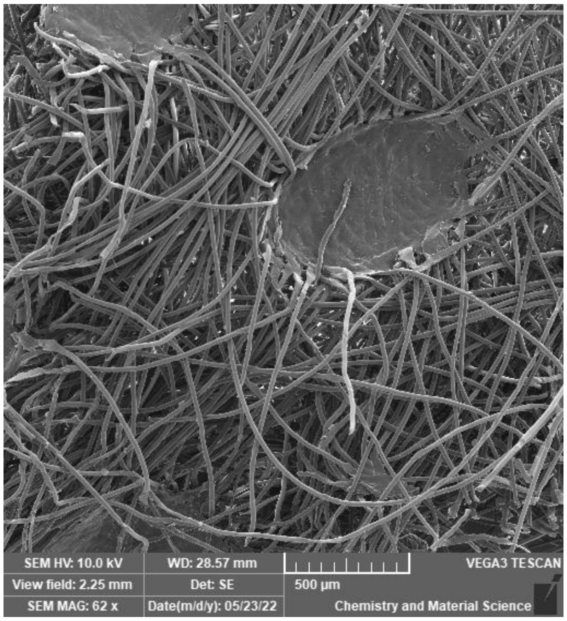

RAN

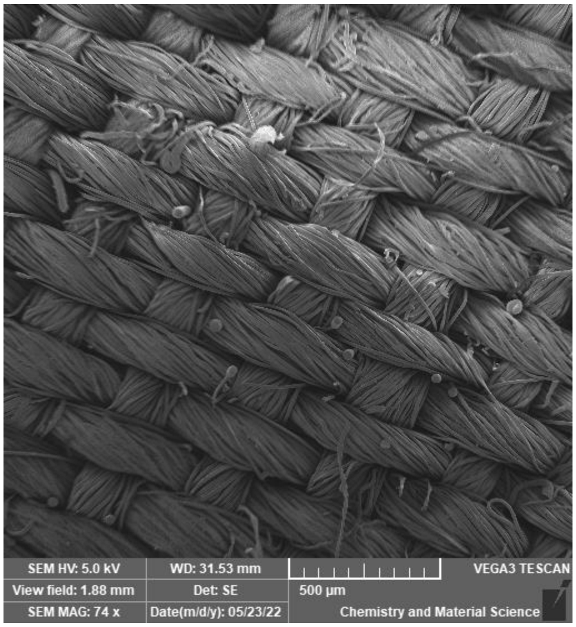

SNC

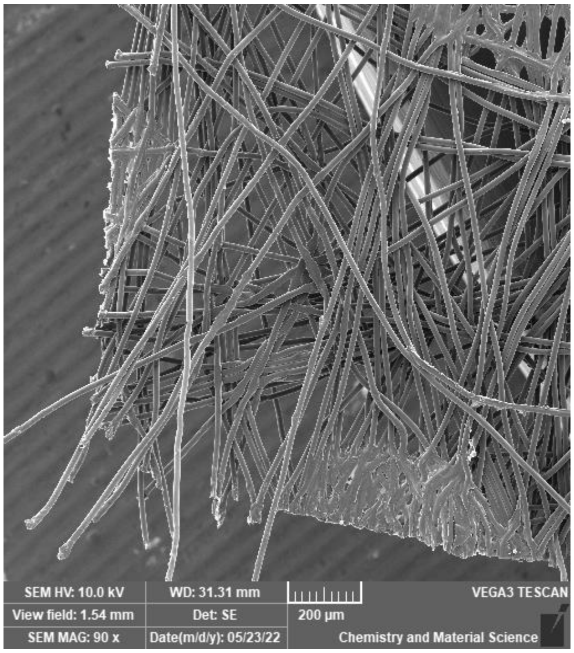

Fig. S-3. SEM images of breathable portions of examples of masks.

Quantitative Result

Analyte	Result	[3-sigma]	Proc.-Calc.	Line	Int.(cps/uA)
C	67.508 %	[28.658]	Quan-FP	C Ka	0.0174
O	32.455 %	[0.671]	Quan-FP	O Ka	0.2775
Si	0.018 %	[0.005]	Quan-FP	SiKa	0.0711
Al	0.014 %	[0.006]	Quan-FP	AlKa	0.0321
P	0.005 %	[0.004]	Quan-FP	P Ka	0.0400

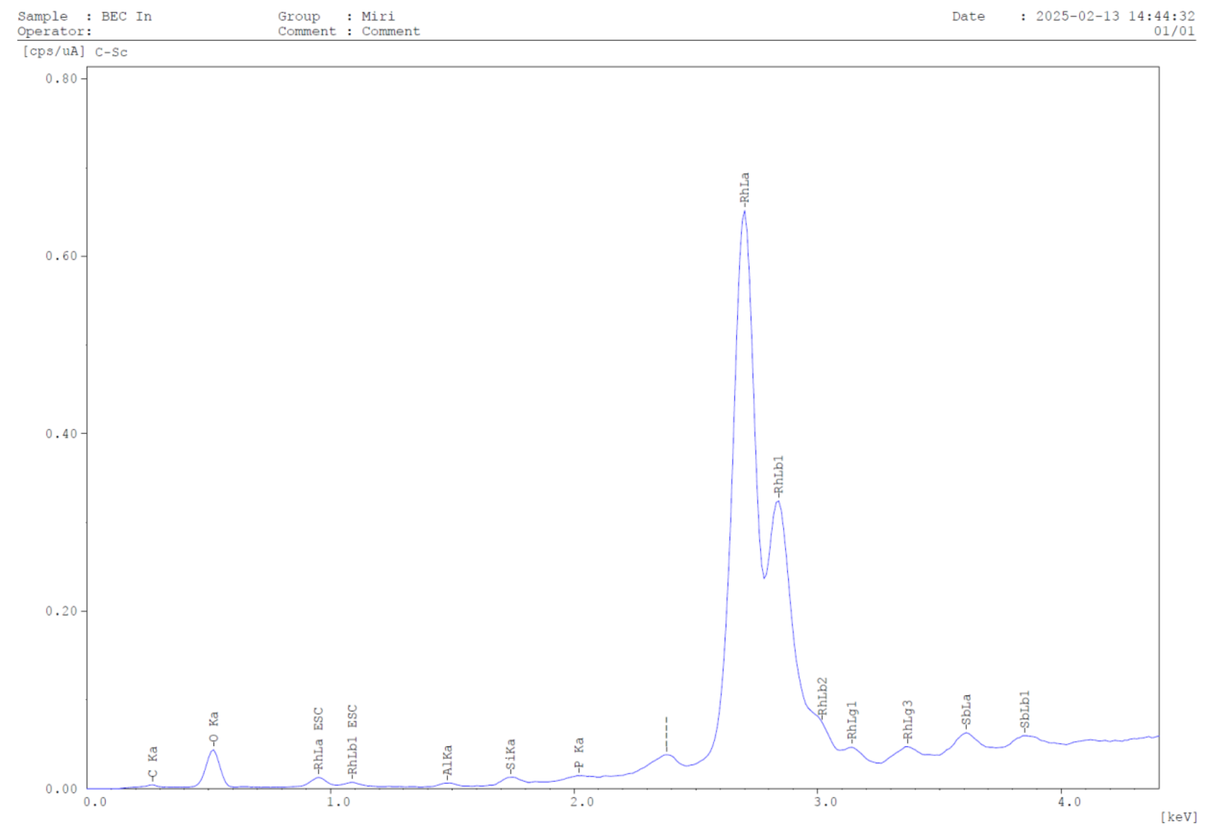

Fig. S-4.1.1. XRF/EDX of BEC - Transparent portion, inside.

Quantitative Result

Analyte	Result	[3-sigma]	Proc.-Calc.	Line	Int.(cps/uA)
C	70.229 %	[29.690]	Quan-FP	C Ka	0.0301
O	29.739 %	[0.608]	Quan-FP	O Ka	0.4075
Si	0.022 %	[0.005]	Quan-FP	SiKa	0.1478
Al	0.007 %	[0.005]	Quan-FP	AlKa	0.0267
P	0.004 %	[0.003]	Quan-FP	P Ka	0.0482

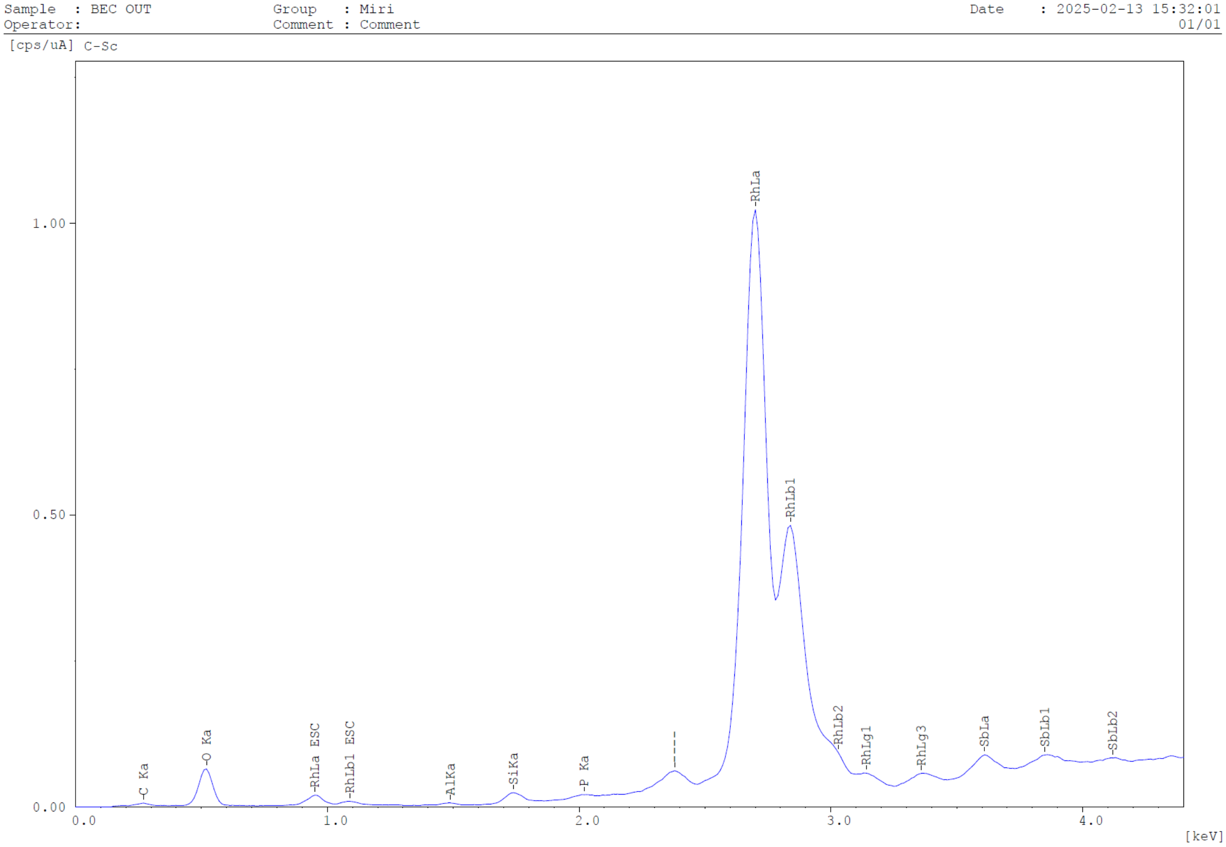

Fig. S-4.1.2 XRF/EDX of BEC - Transparent portion, outside.

Quantitative Result

Analyte	Result	[3-sigma]	Proc.-Calc.	Line	Int.(cps/uA)
C	71.255 %	[29.806]	Quan-FP	C Ka	0.0347
O	28.506 %	[0.521]	Quan-FP	O Ka	0.4400
S	0.116 %	[0.001]	Quan-FP	S Ka	3.4660
Cs	0.077 %	[0.011]	Quan-FP	CsLa	1.1496
Si	0.024 %	[0.006]	Quan-FP	SiKa	0.1844
Al	0.011 %	[0.005]	Quan-FP	AlKa	0.0501
Sc	0.011 %	[0.002]	Quan-FP	ScKa	0.6548

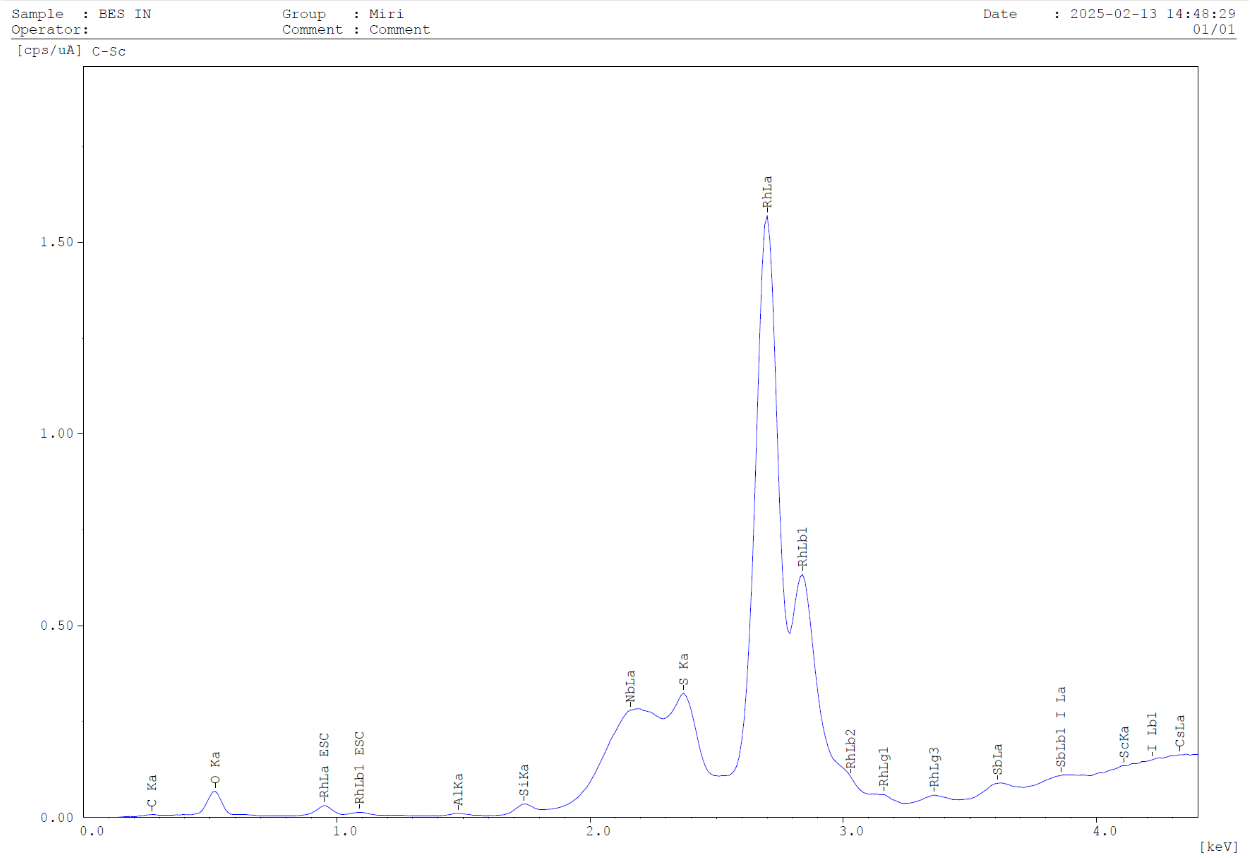

Fig. S-4.2.1. XRF/EDX of BES - Transparent portion, inside.

Quantitative Result

Analyte	Result	[3-sigma]	Proc.-Calc.	Line	Int.(cps/uA)
C	71.021 %	[30.304]	Quan-FP	C Ka	0.0320
O	28.750 %	[0.534]	Quan-FP	O Ka	0.4118
S	0.117 %	[0.001]	Quan-FP	S Ka	3.2136
Cs	0.077 %	[0.011]	Quan-FP	CsLa	1.0638
Si	0.023 %	[0.006]	Quan-FP	SiKa	0.1644
Al	0.013 %	[0.006]	Quan-FP	AlKa	0.0517

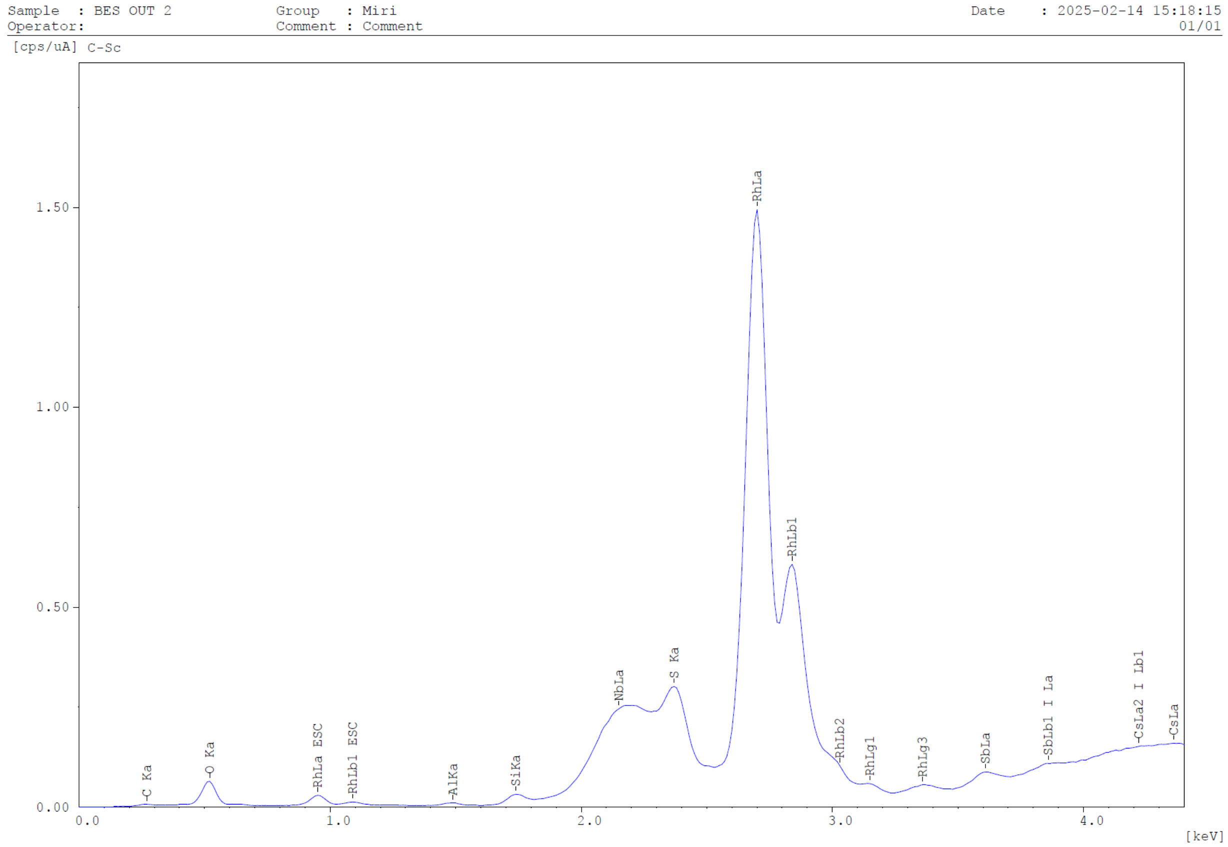

Fig. S-4.2.2. XRF/EDX of BES - Transparent portion, outside.

Quantitative Result

Analyte	Result	[3-sigma]	Proc.-Calc.	Line	Int.(cps/uA)
C	70.540 %	[30.558]	Quan-FP	C Ka	0.0355
O	29.234 %	[0.545]	Quan-FP	O Ka	0.4726
S	0.181 %	[0.001]	Quan-FP	S Ka	5.5735
Si	0.032 %	[0.006]	Quan-FP	SiKa	0.2605
Al	0.012 %	[0.006]	Quan-FP	AlKa	0.0571

Sample : CLM IN Group : Miri Date : 2025-02-13 14:51:12
Operator: Comment : Comment 01/01

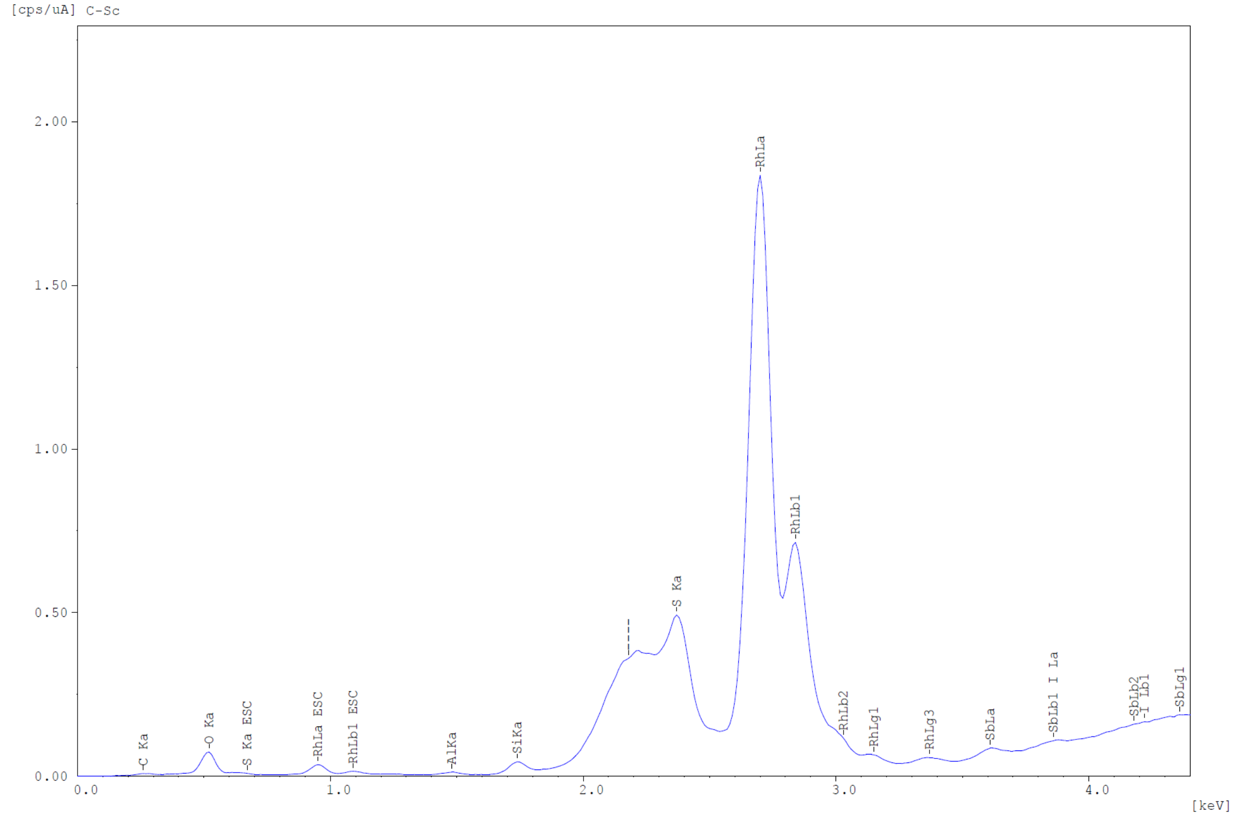

Fig. S-4.3.1. XRF/EDX of CLM - Transparent portion, inside.

Quantitative Result

Analyte	Result	[3-sigma]	Proc.-Calc.	Line	Int.(cps/uA)
C	71.719 %	[31.095]	Quan-FP	C Ka	0.0365
O	28.104 %	[0.530]	Quan-FP	O Ka	0.4511
S	0.112 %	[0.001]	Quan-FP	S Ka	3.5138
Si	0.039 %	[0.006]	Quan-FP	SiKa	0.3164
Sc	0.014 %	[0.003]	Quan-FP	ScKa	0.9258
Al	0.011 %	[0.006]	Quan-FP	AlKa	0.0529

Sample : CLM OUT Group : Miri Date : 2025-02-13 15:38:40
Operator: Comment : Comment 01/01

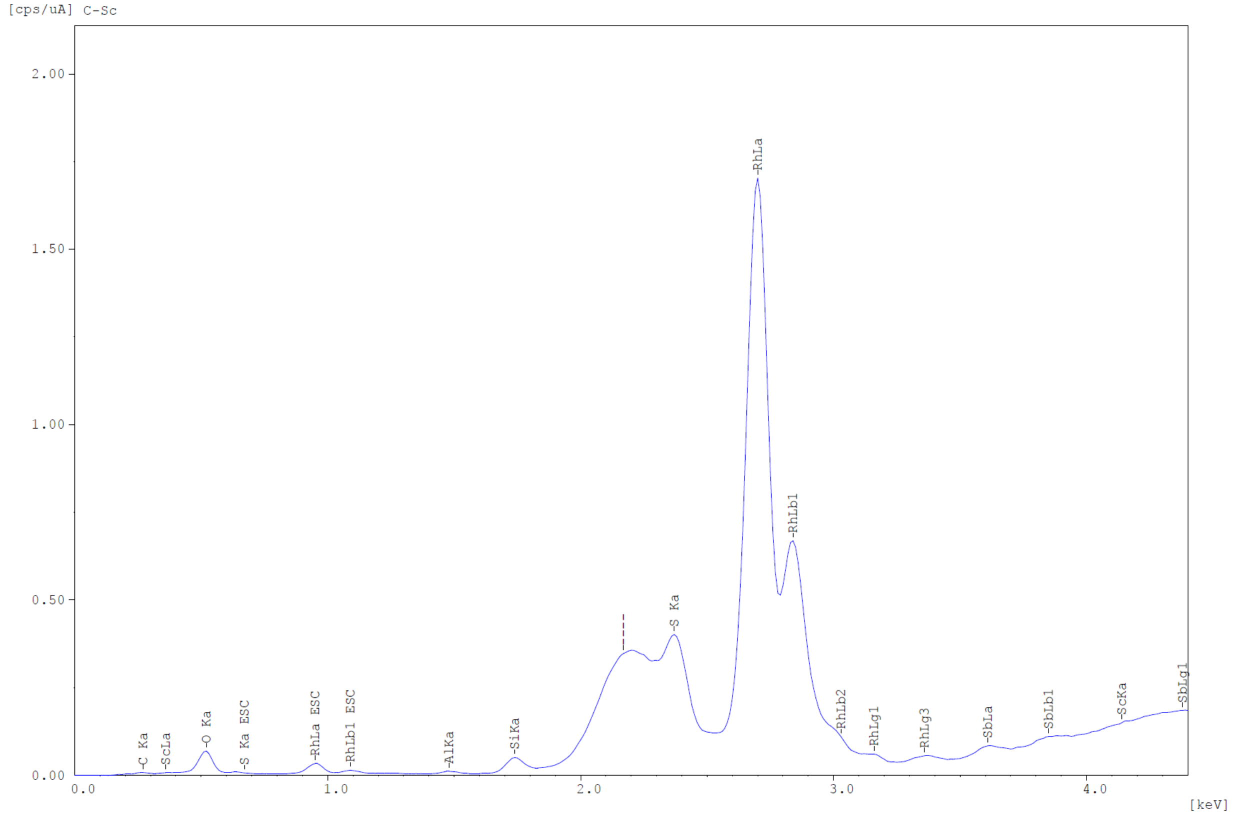

Fig. S-4.3.2. XRF/EDX of CLM - Transparent portion, outside.

Quantitative Result

Analyte	Result	[3-sigma]	Proc.-Calc.	Line	Int.(cps/uA)
C	55.846 %	[26.580]	Quan-FP	C Ka	0.0196
O	40.937 %	[0.719]	Quan-FP	O Ka	0.6157
Si	2.965 %	[0.010]	Quan-FP	SiKa	17.3338
S	0.217 %	[0.001]	Quan-FP	S Ka	4.2359
Al	0.024 %	[0.006]	Quan-FP	AlKa	0.0813
Ca	0.011 %	[0.003]	Quan-FP	CaKa	0.2413

Sample : FAV IN 2 Group : Miri Date : 2025-02-14 15:22:08
Operator: Comment : Comment 01/01

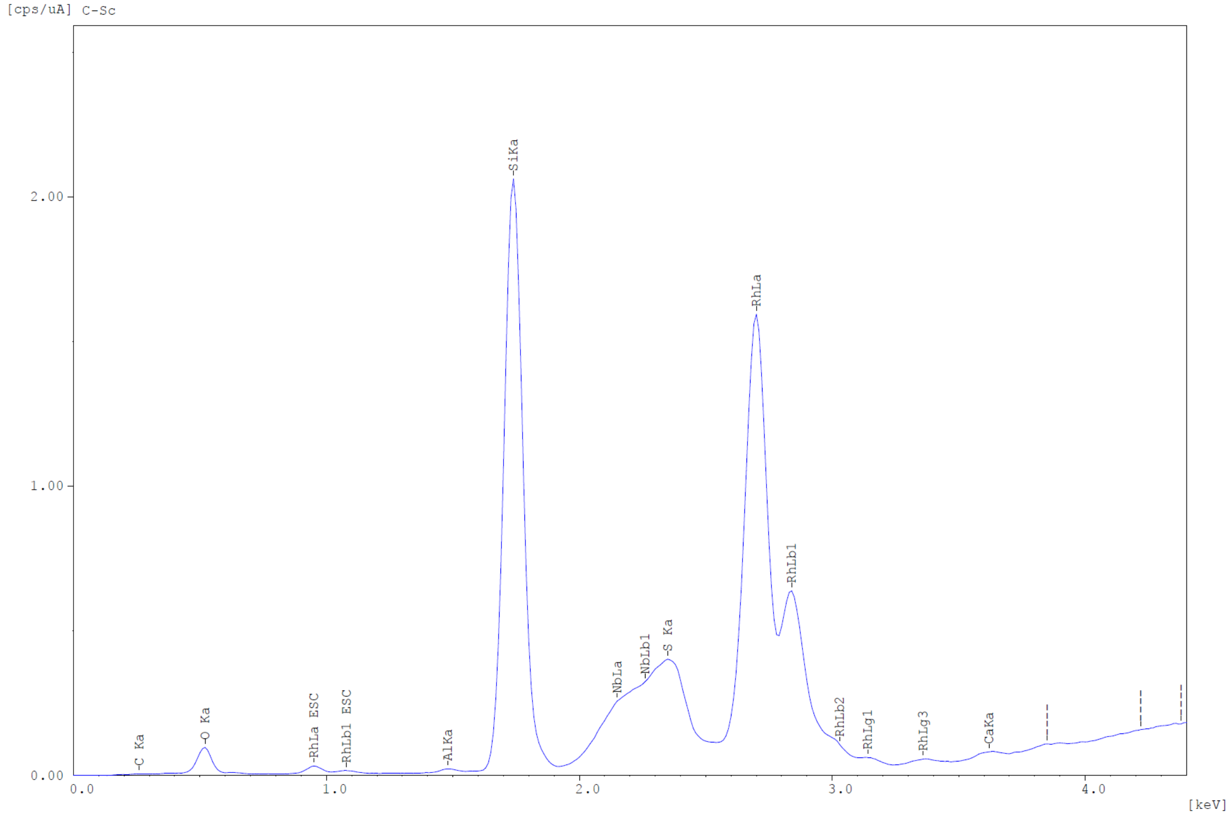

Fig. S-4.4.1. XRF/EDX of FAV - Transparent portion, inside.

Quantitative Result

Analyte	Result	[3-sigma]	Proc.-Calc.	Line	Int.(cps/uA)
C	54.324 %	[26.435]	Quan-FP	C Ka	0.0180
O	42.118 %	[0.742]	Quan-FP	O Ka	0.6105
Si	3.085 %	[0.011]	Quan-FP	SiKa	16.8398
S	0.270 %	[0.001]	Quan-FP	S Ka	4.8886
Cs	0.154 %	[0.012]	Quan-FP	CsLa	1.3245
Sc	0.026 %	[0.003]	Quan-FP	ScKa	0.9186
Al	0.023 %	[0.006]	Quan-FP	AlKa	0.0750

Sample : FAV OUT 2 Group : Miri Date : 2025-02-14 15:24:45
Operator: Comment : Comment 01/01

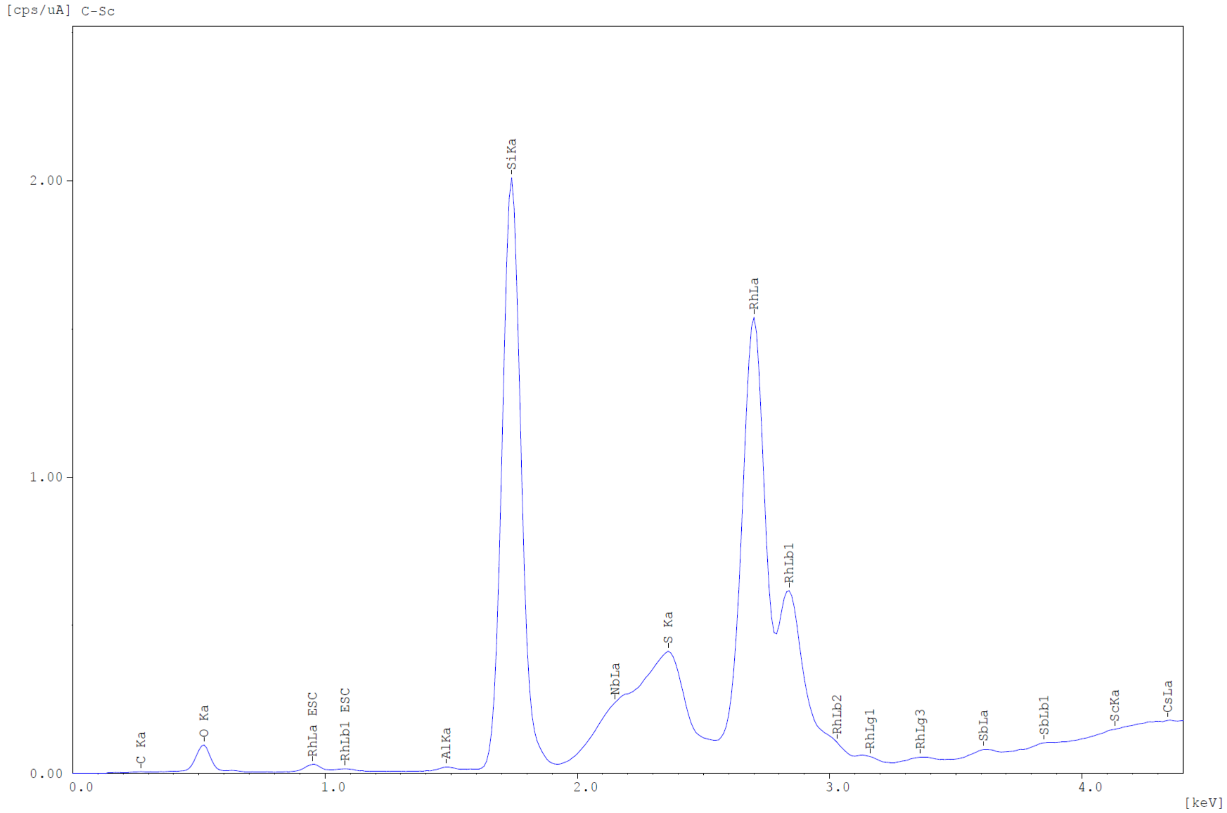

Fig. S-4.4.2. XRF/EDX of FAV - Transparent portion, outside.

Quantitative Result

Analyte	Result	[3-sigma]	Proc.-Calc.	Line	Int.(cps/uA)
O	53.869 %	[1.388]	Quan-FP	O Ka	0.4632
Si	43.789 %	[0.098]	Quan-FP	SiKa	67.5195
Al	1.459 %	[0.020]	Quan-FP	AlKa	1.6086
S	0.883 %	[0.010]	Quan-FP	S Ka	2.1802

Sample : JEM IN Group : Miri Date : 2025-02-13 14:56:32
Operator: Comment : Comment 01/01

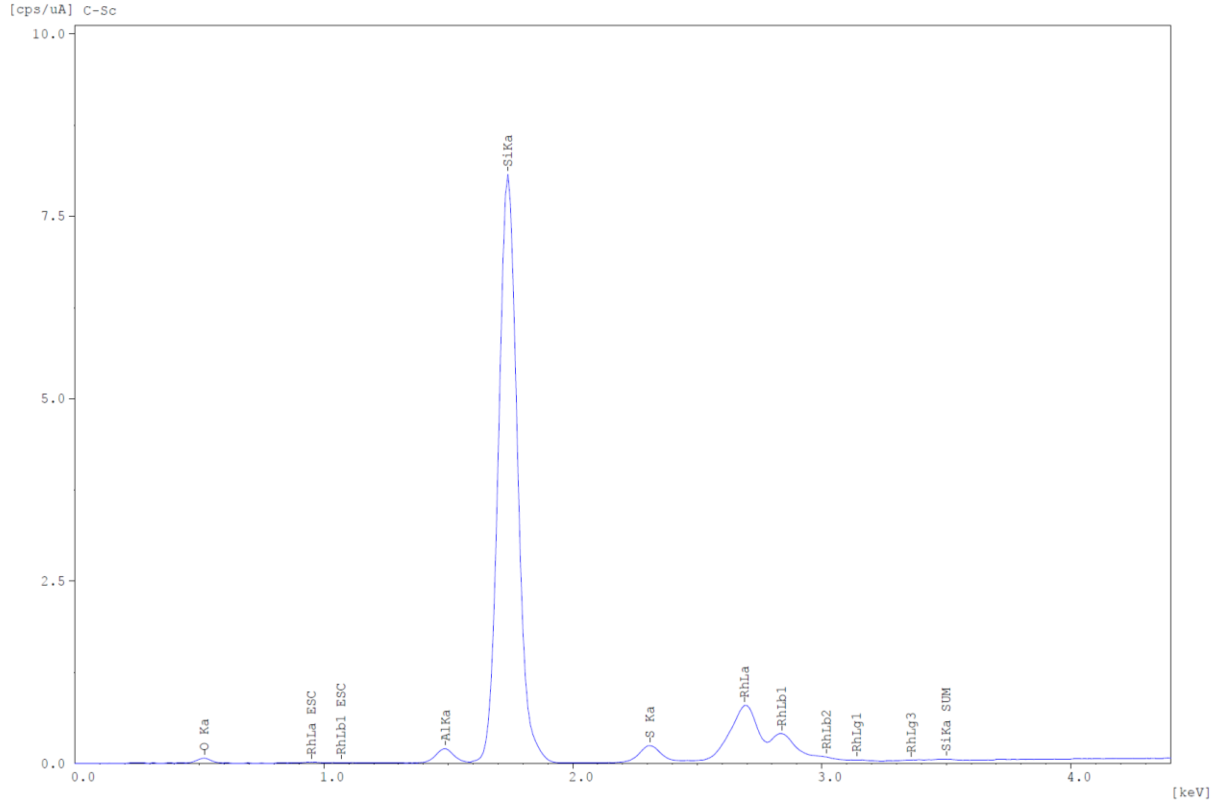

Fig. S-4.5.1. XRF/EDX of JEM - Transparent portion, inside.

Quantitative Result

Analyte	Result	[3-sigma]	Proc.-Calc.	Line	Int.(cps/uA)
O	53.175 %	[1.335]	Quan-FP	O Ka	0.4855
Si	44.423 %	[0.096]	Quan-FP	SiKa	73.2084
Al	1.484 %	[0.020]	Quan-FP	AlKa	1.7550
S	0.918 %	[0.010]	Quan-FP	S Ka	2.4032

Sample : JEM OUT
Operator:
Group : Miri
Comment : Comment
Date : 2025-02-13 15:44:01
01/01

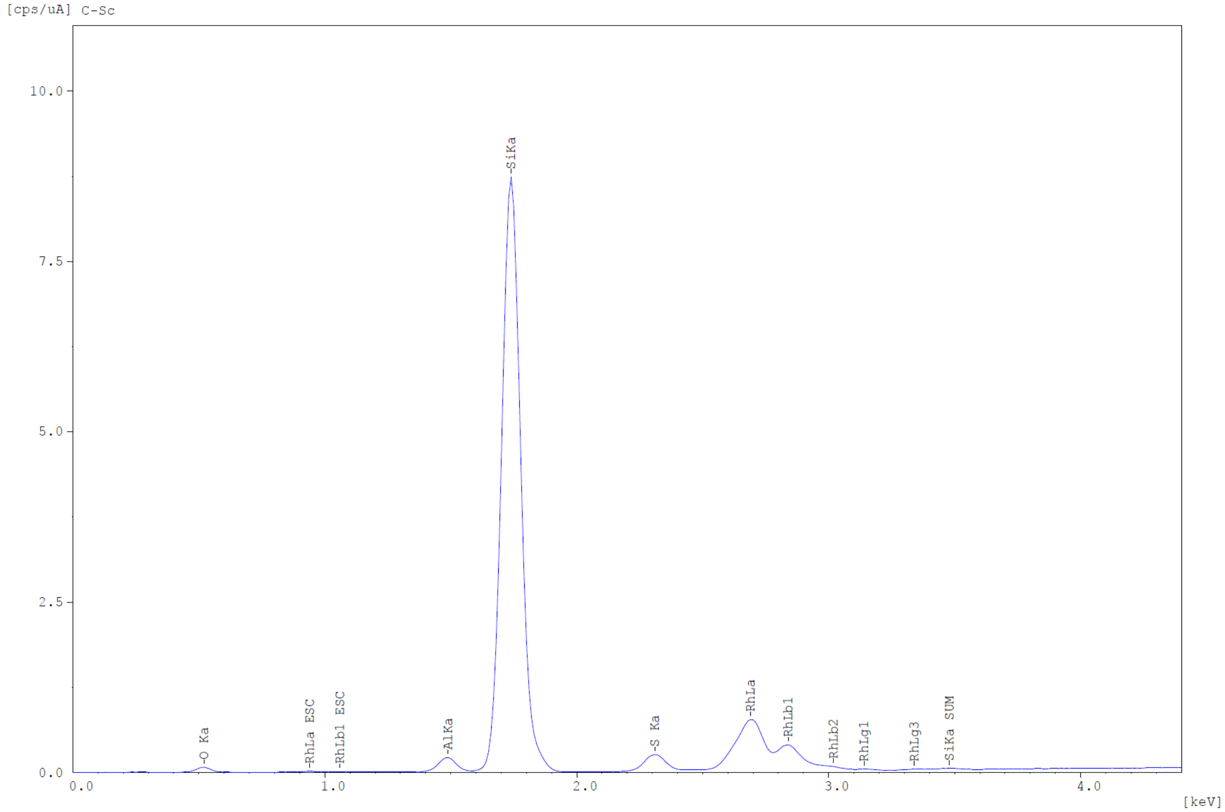

Fig. S-4.5.2. XRF/EDX of JEM - Transparent portion, outside.

Quantitative Result

Analyte	Result	[3-sigma]	Proc.-Calc.	Line	Int.(cps/uA)
C	99.858 %	[4.178]	Quan-FP	C Ka	0.0424
Si	0.083 %	[0.001]	Quan-FP	SiKa	0.6188
Al	0.018 %	[0.001]	Quan-FP	AlKa	0.0788
Mg	0.018 %	[0.002]	Quan-FP	MgKa	0.0319
S	0.009 %	[0.000]	Quan-FP	S Ka	0.2776
Ca	0.006 %	[0.000]	Quan-FP	CaKa	0.2232
P	0.006 %	[0.000]	Quan-FP	P Ka	0.0837

Sample : OPT IN   Group : Miri   Date : 2025-02-13 14:59:09
Operator: Comment : Comment   01/01

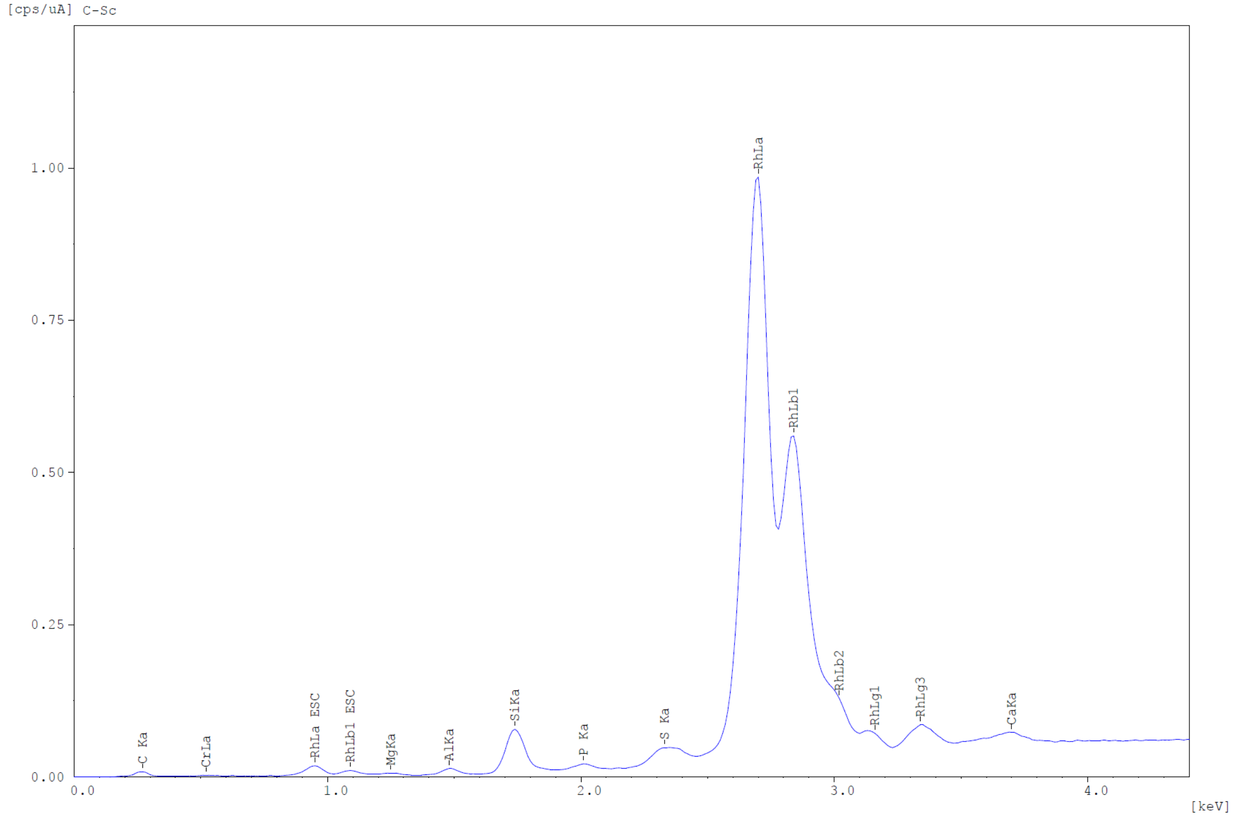

Fig. S-4.6.1. XRF/EDX of OPT - Transparent portion, inside.

Quantitative Result

Analyte	Result	[3-sigma]	Proc.-Calc.	Line	Int.(cps/uA)
C	98.672 %	[5.796]	Quan-FP	C Ka	0.0204
O	1.201 %	[0.267]	Quan-FP	O Ka	0.0053
Si	0.070 %	[0.001]	Quan-FP	SiKa	0.2527
Al	0.025 %	[0.001]	Quan-FP	AlKa	0.0510
Mg	0.016 %	[0.002]	Quan-FP	MgKa	0.0136
S	0.009 %	[0.000]	Quan-FP	S Ka	0.1326
P	0.005 %	[0.000]	Quan-FP	P Ka	0.0315
Ca	0.002 %	[0.000]	Quan-FP	CaKa	0.0427

Sample : OPT OUT Group : Miri Date : 2025-02-13 15:46:40
Operator: Comment : Comment 01/01

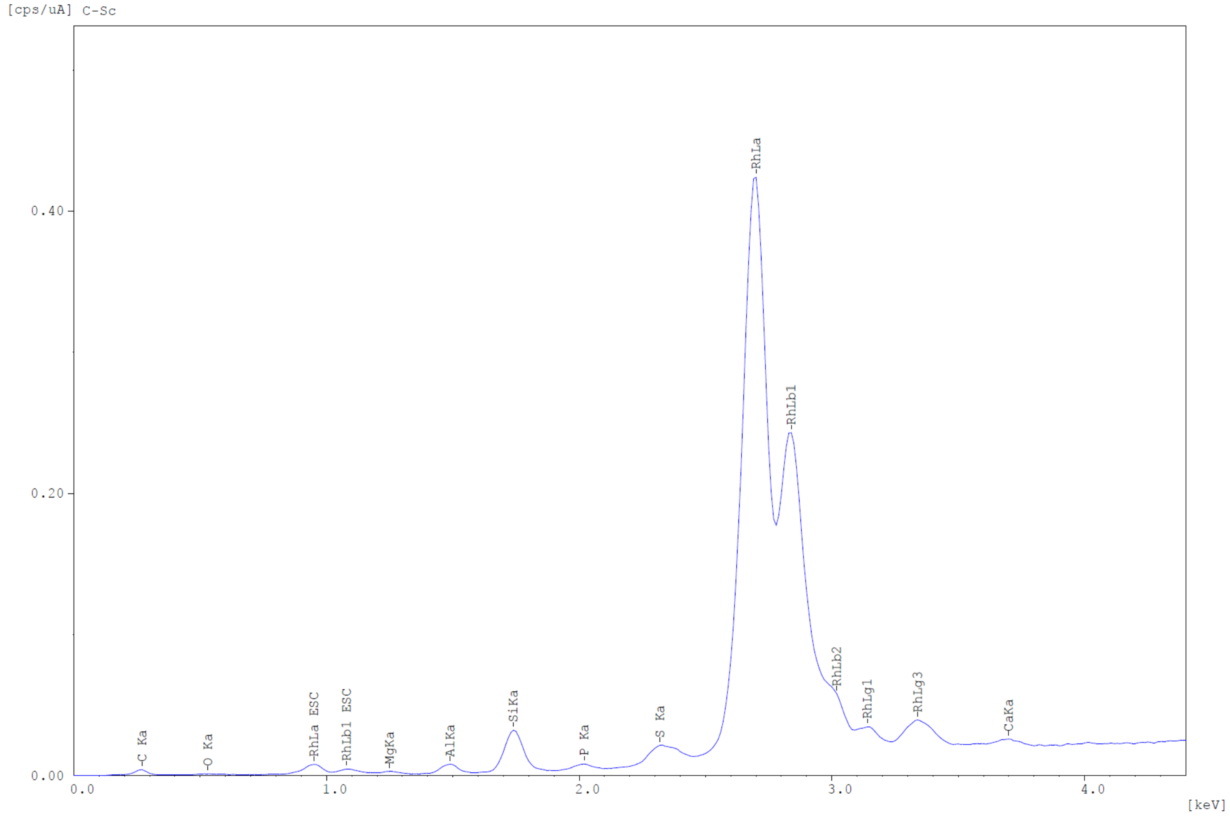

Fig. S-4.6.2. XRF/EDX of OPT - Transparent portion, outside.

Quantitative Result

Analyte	Result	[3-sigma]	Proc.-Calc.	Line	Int.(cps/uA)
Cl	99.702 %	[0.128]	Quan-FP	ClKa	727.8935
Al	0.178 %	[0.010]	Quan-FP	AlKa	0.7674
Si	0.088 %	[0.006]	Quan-FP	SiKa	0.6180
S	0.032 %	[0.003]	Quan-FP	S Ka	0.7241

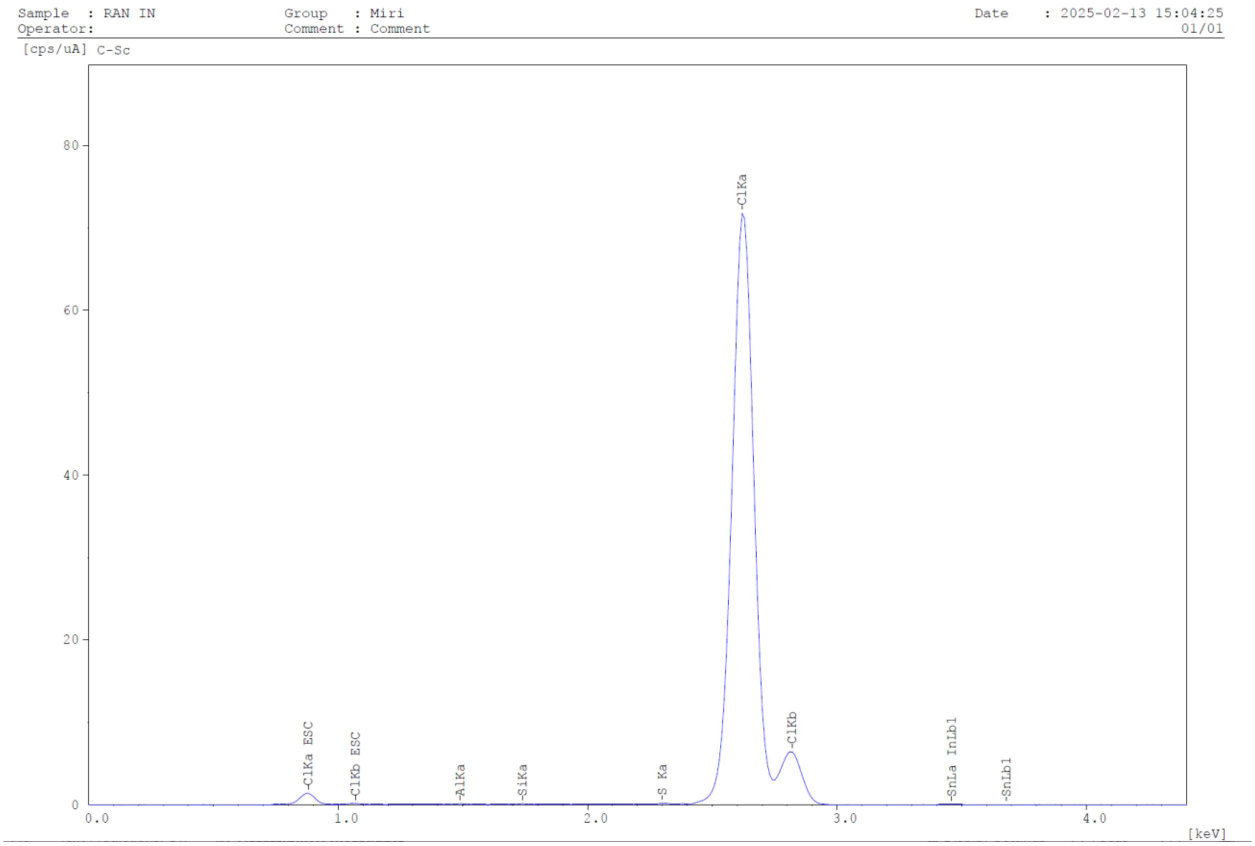

Fig. S-4.7.1. XRF/EDX of RAN - Transparent portion, inside.

Quantitative Result

Analyte	Result	[3-sigma]	Proc.-Calc.	Line	Int.(cps/uA)
Cl	99.675 %	[0.126]	Quan-FP	ClKa	646.5159
Al	0.199 %	[0.010]	Quan-FP	AlKa	0.7605
Si	0.091 %	[0.006]	Quan-FP	SiKa	0.5692
S	0.035 %	[0.003]	Quan-FP	S Ka	0.7017

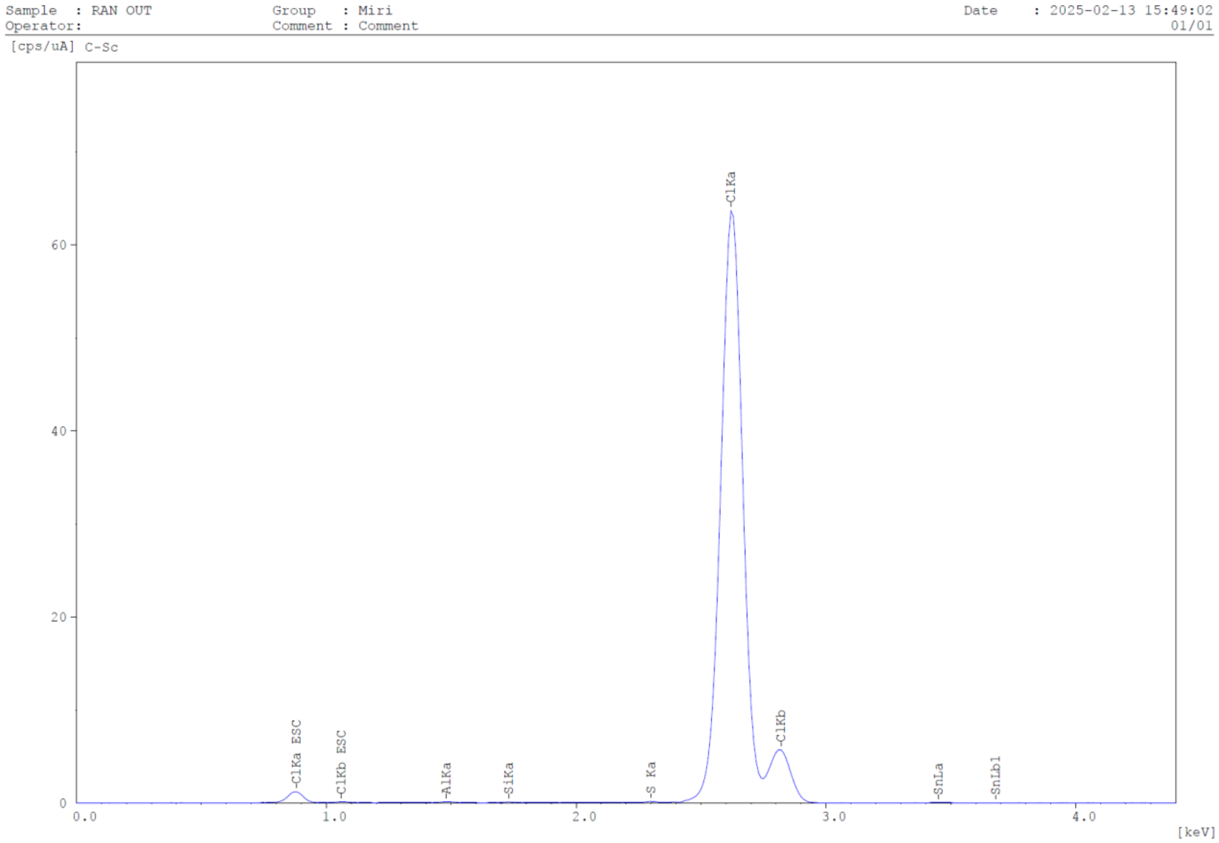

Fig. S-4.7.2. XRF/EDX of RAN - Transparent portion, outside.

Quantitative Result

Analyte	Result	[3-sigma]	Proc.-Calc.	Line	Int.(cps/uA)
Si	77.530 %	[0.104]	Quan-FP	SiKa	262.8430
O	21.959 %	[0.726]	Quan-FP	O Ka	0.3142
Al	0.486 %	[0.008]	Quan-FP	AlKa	1.3658
S	0.024 %	[0.003]	Quan-FP	S Ka	0.0914

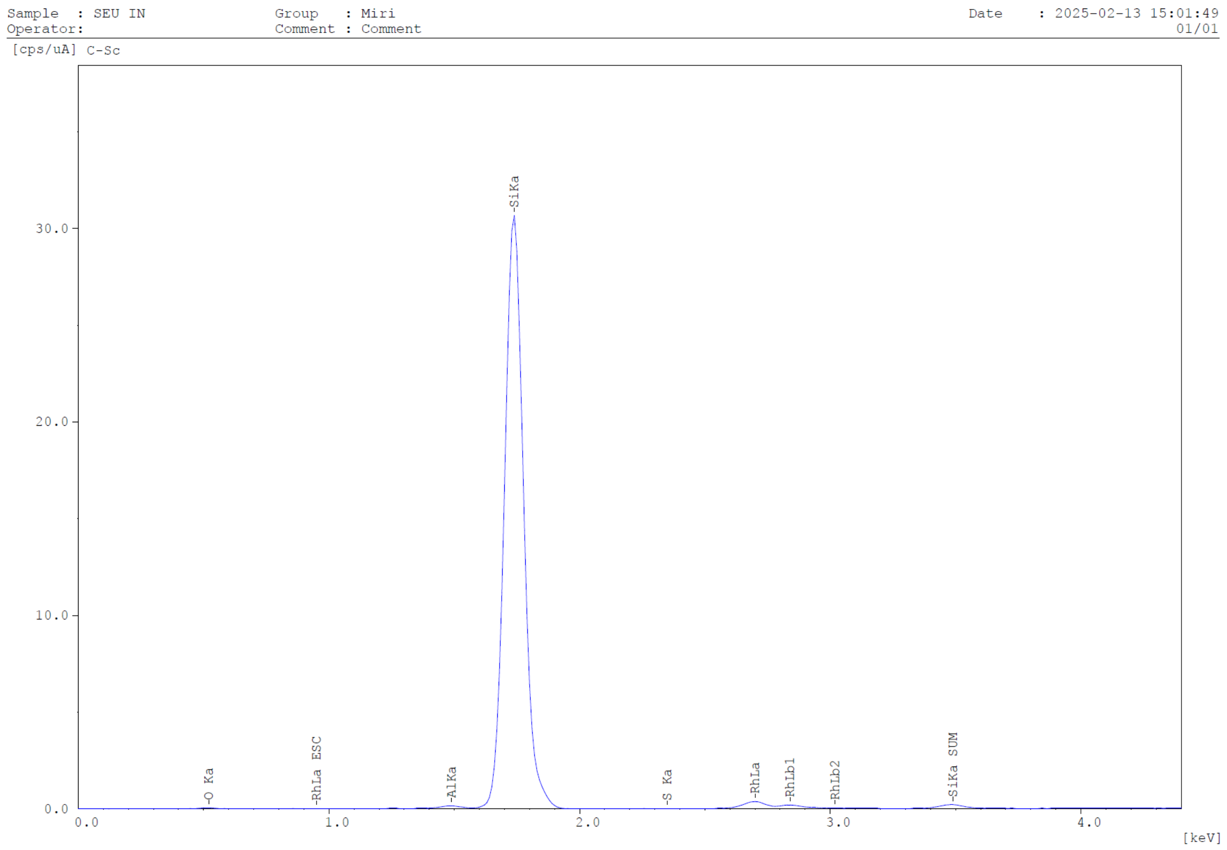

Fig. S-4.8.1. XRF/EDX of SEU - Transparent portion, inside.

Quantitative Result

Analyte	Result	[3-sigma]	Proc.-Calc.	Line	Int.(cps/uA)
Si	77.721 %	[0.102]	Quan-FP	SiKa	270.7891
O	21.798 %	[0.712]	Quan-FP	O Ka	0.3202
Al	0.481 %	[0.008]	Quan-FP	AlKa	1.3889

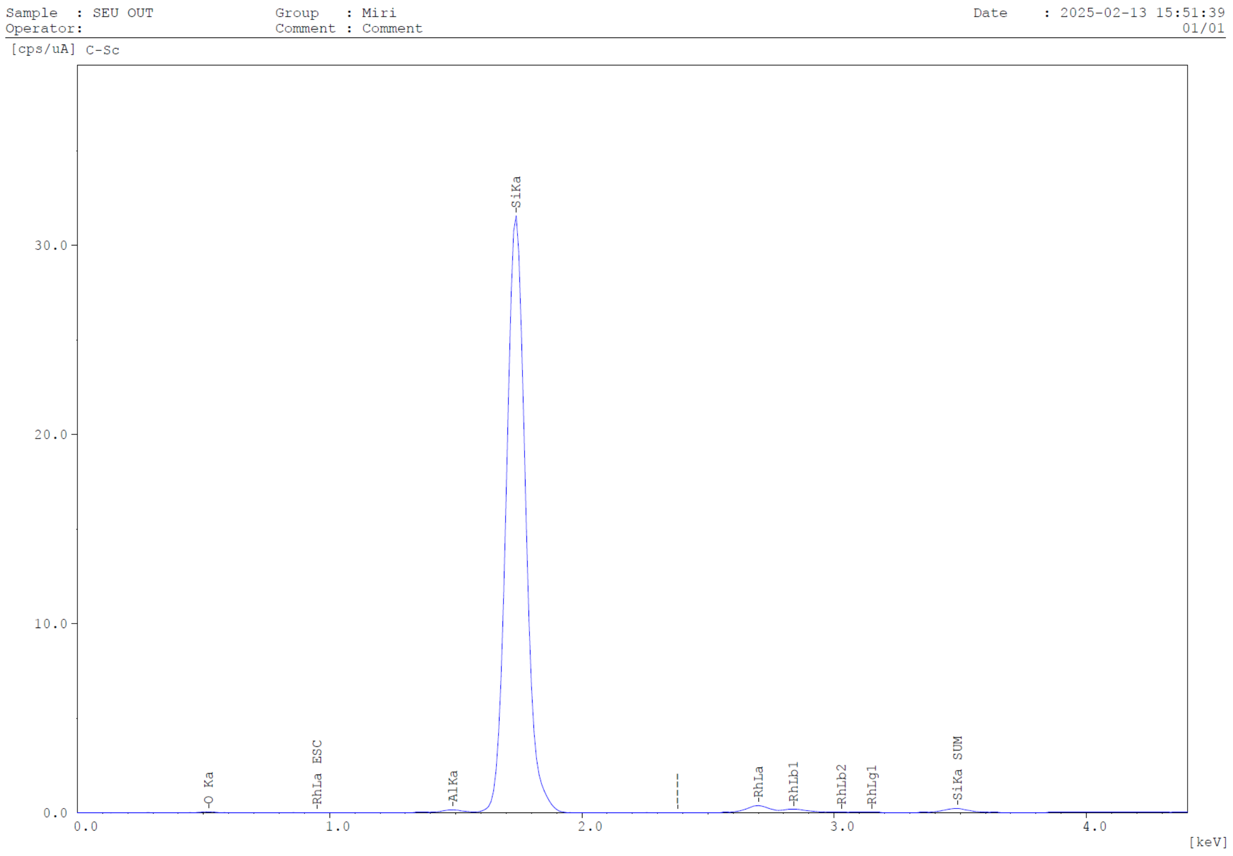

Fig. S-4.8.2. XRF/EDX of SEU - Transparent portion, outside.

Quantitative Result

Analyte	Result	[3-sigma]	Proc.-Calc.	Line	Int.(cps/uA)
C	71.435 %	[30.058]	Quan-FP	C Ka	0.0385
O	28.305 %	[0.519]	Quan-FP	O Ka	0.4844
S	0.193 %	[0.001]	Quan-FP	S Ka	6.4013
Si	0.041 %	[0.006]	Quan-FP	SiKa	0.3577
Sc	0.014 %	[0.002]	Quan-FP	ScKa	0.9260
Al	0.012 %	[0.006]	Quan-FP	AlKa	0.0620

Sample : SNC IN   Group : Miri   Date : 2025-02-13 15:07:02
Operator: Comment : Comment   01/01

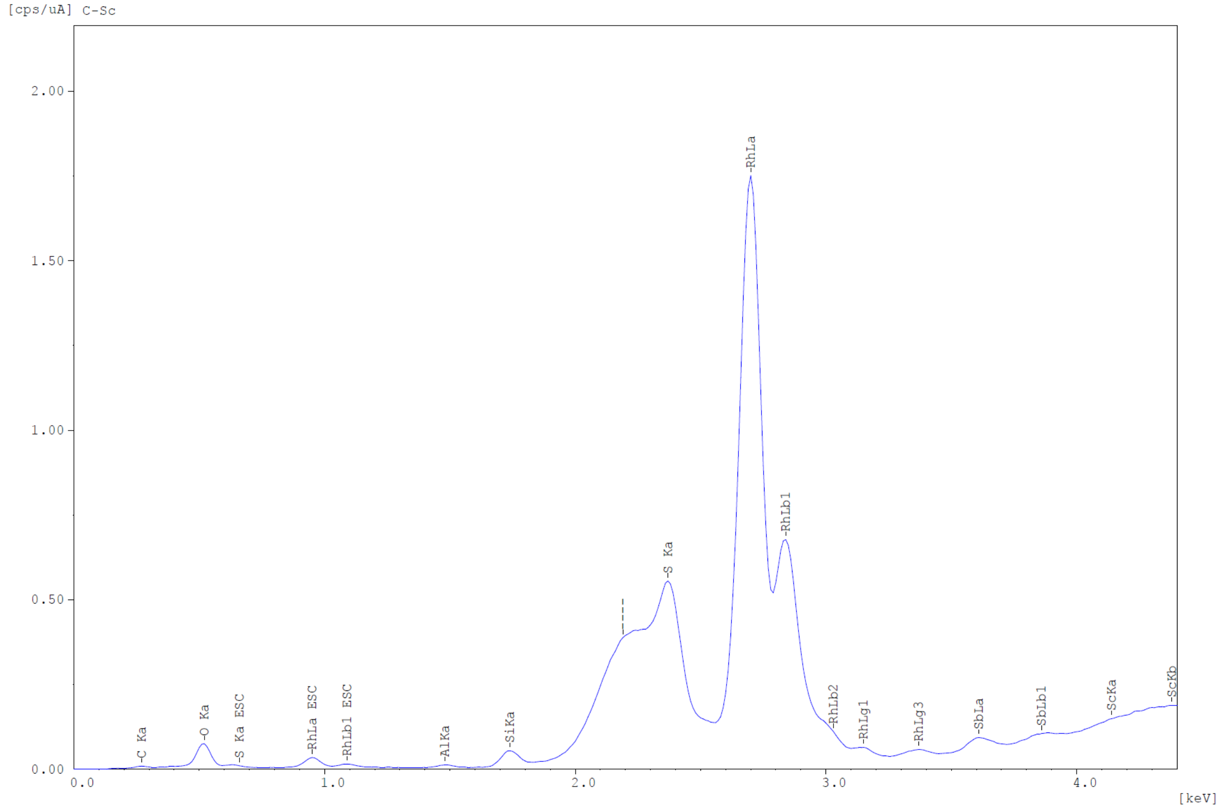

Fig. S-4.9.1. XRF/EDX of SNC - Transparent portion, inside.

Quantitative Result

Analyte	Result	[3-sigma]	Proc.-Calc.	Line	Int.(cps/uA)
C	77.201 %	[3.064]	Quan-FP	C Ka	0.0309
O	22.744 %	[0.421]	Quan-FP	O Ka	0.2643
Si	0.030 %	[0.001]	Quan-FP	SiKa	0.1983
Al	0.013 %	[0.001]	Quan-FP	AlKa	0.0481
Ca	0.012 %	[0.000]	Quan-FP	CaKa	0.3559

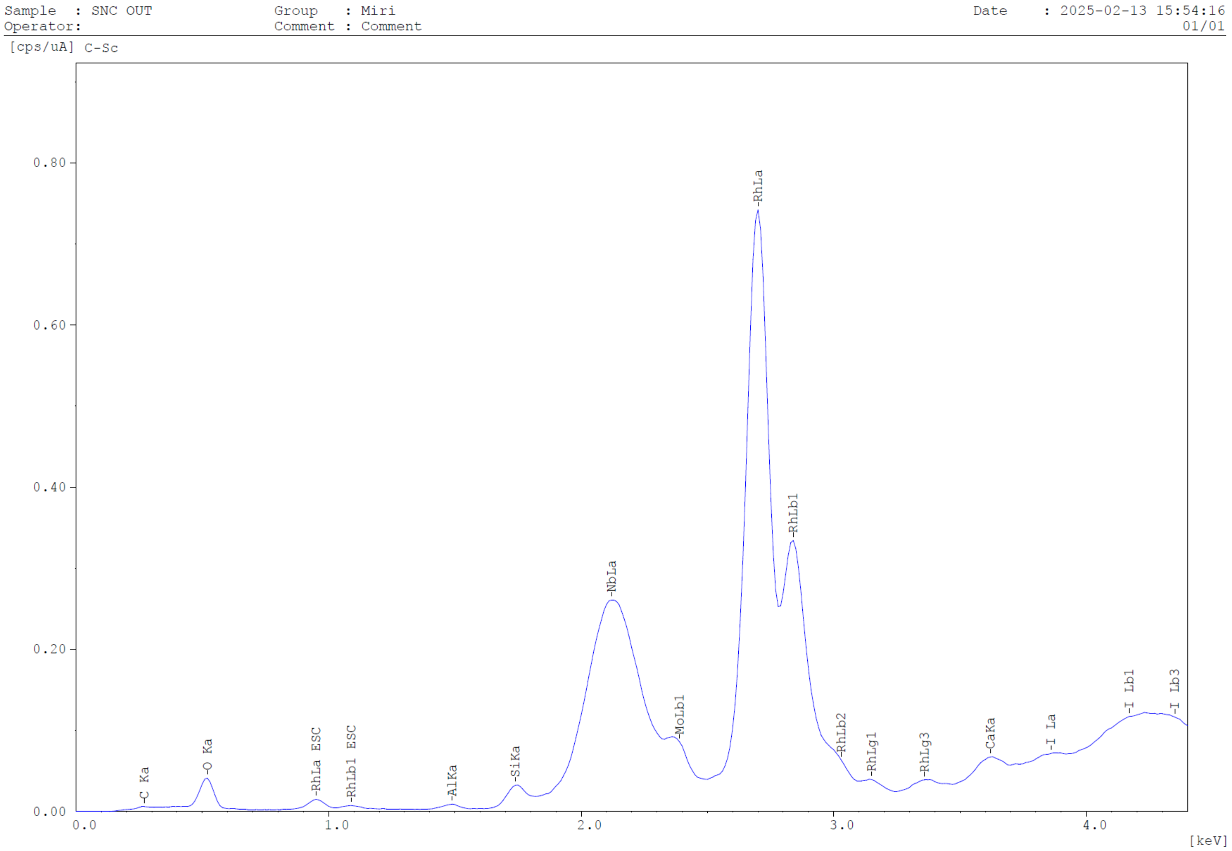

Fig. S-4.9.2. XRF/EDX of SNC - Transparent portion, outside.

Quantitative Result

Analyte	Result	[3-sigma]	Proc.-Calc.	Line	Int.(cps/uA)
C	68.135 %	[29.608]	Quan-FP	C Ka	0.0304
O	31.641 %	[0.595]	Quan-FP	O Ka	0.4680
S	0.173 %	[0.001]	Quan-FP	S Ka	4.6796
Si	0.028 %	[0.005]	Quan-FP	SiKa	0.1952
Sc	0.012 %	[0.003]	Quan-FP	ScKa	0.6374
Al	0.012 %	[0.005]	Quan-FP	AlKa	0.0469

Sample : STK IN 2 Group : Miri Date : 2025-02-14 15:27:21
Operator: Comment : Comment 01/01

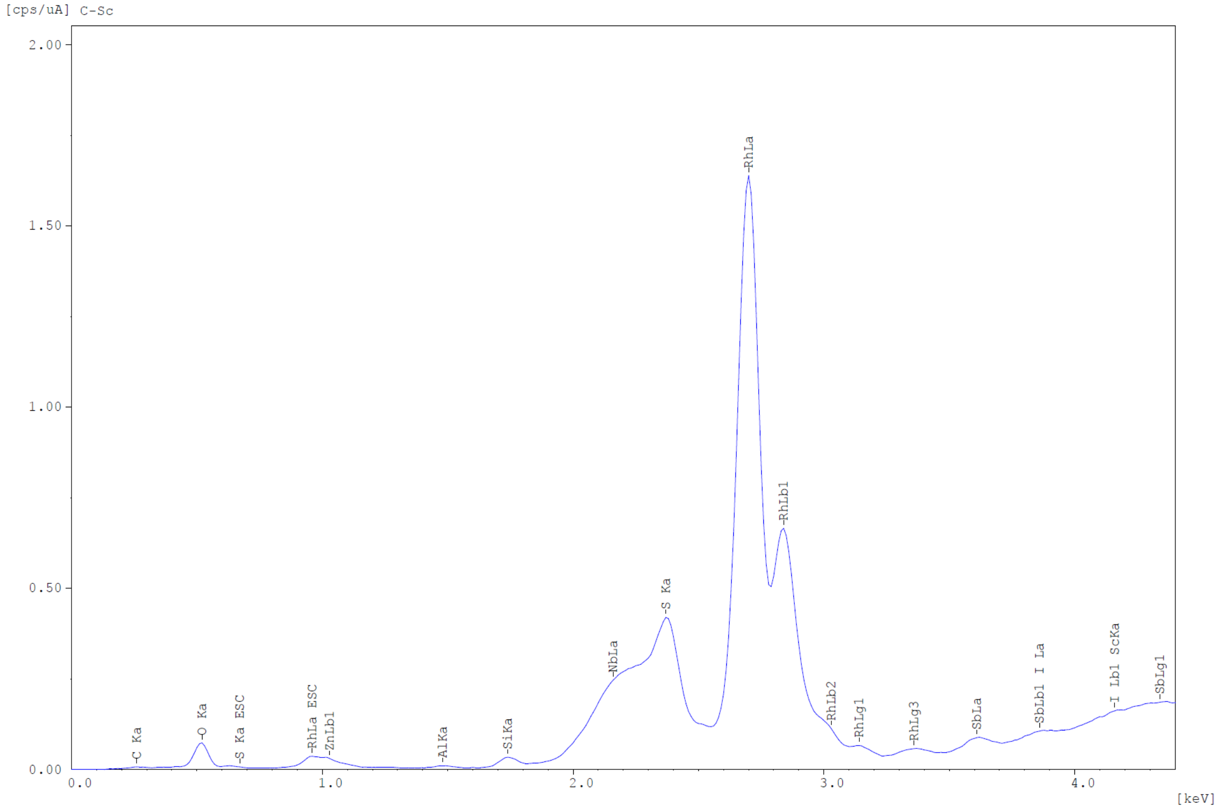

Fig. S-4.10.1. XRF/EDX of STK - Transparent portion, inside.

Quantitative Result

Analyte	Result	[3-sigma]	Proc.-Calc.	Line	Int.(cps/uA)
C	70.442 %	[29.540]	Quan-FP	C Ka	0.0347
O	29.294 %	[0.555]	Quan-FP	O Ka	0.4630
S	0.147 %	[0.001]	Quan-FP	S Ka	4.4088
Cs	0.090 %	[0.012]	Quan-FP	CsLa	1.3518
Si	0.016 %	[0.005]	Quan-FP	SiKa	0.1279
Al	0.010 %	[0.006]	Quan-FP	AlKa	0.0472

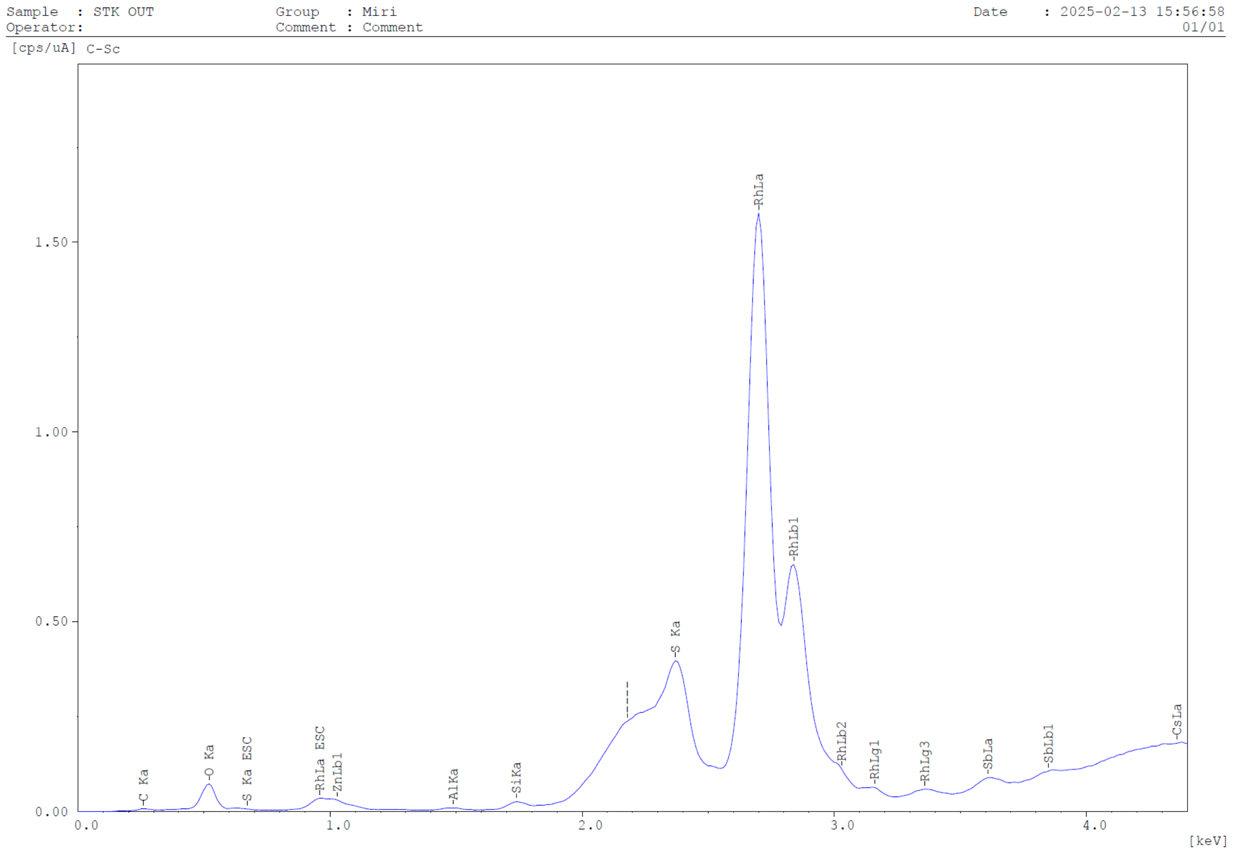

Fig. S-4.10.2. XRF/EDX of STK - Transparent portion, outside.

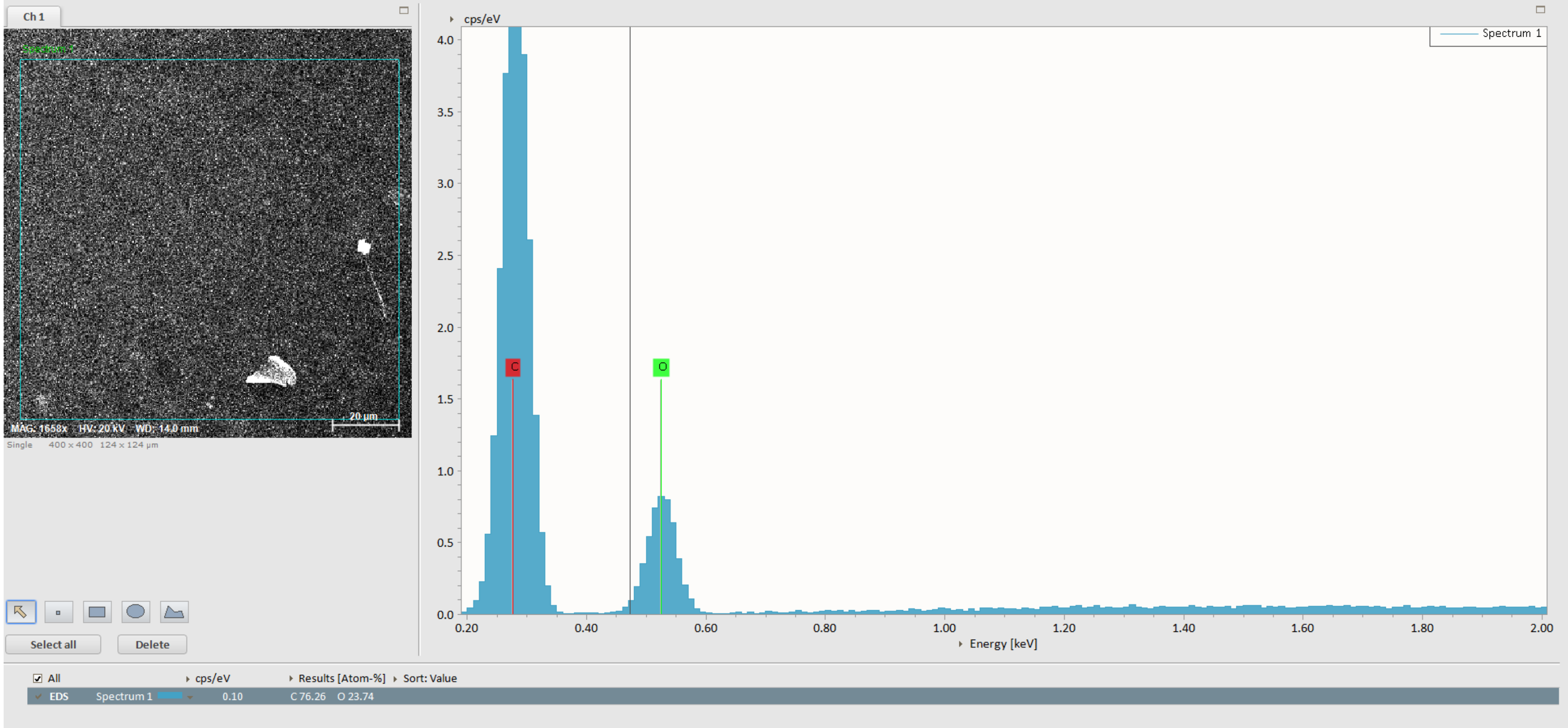

Fig. S-4.1.1 SEM/EDX diagram for BEC - Transparent portion, inside.

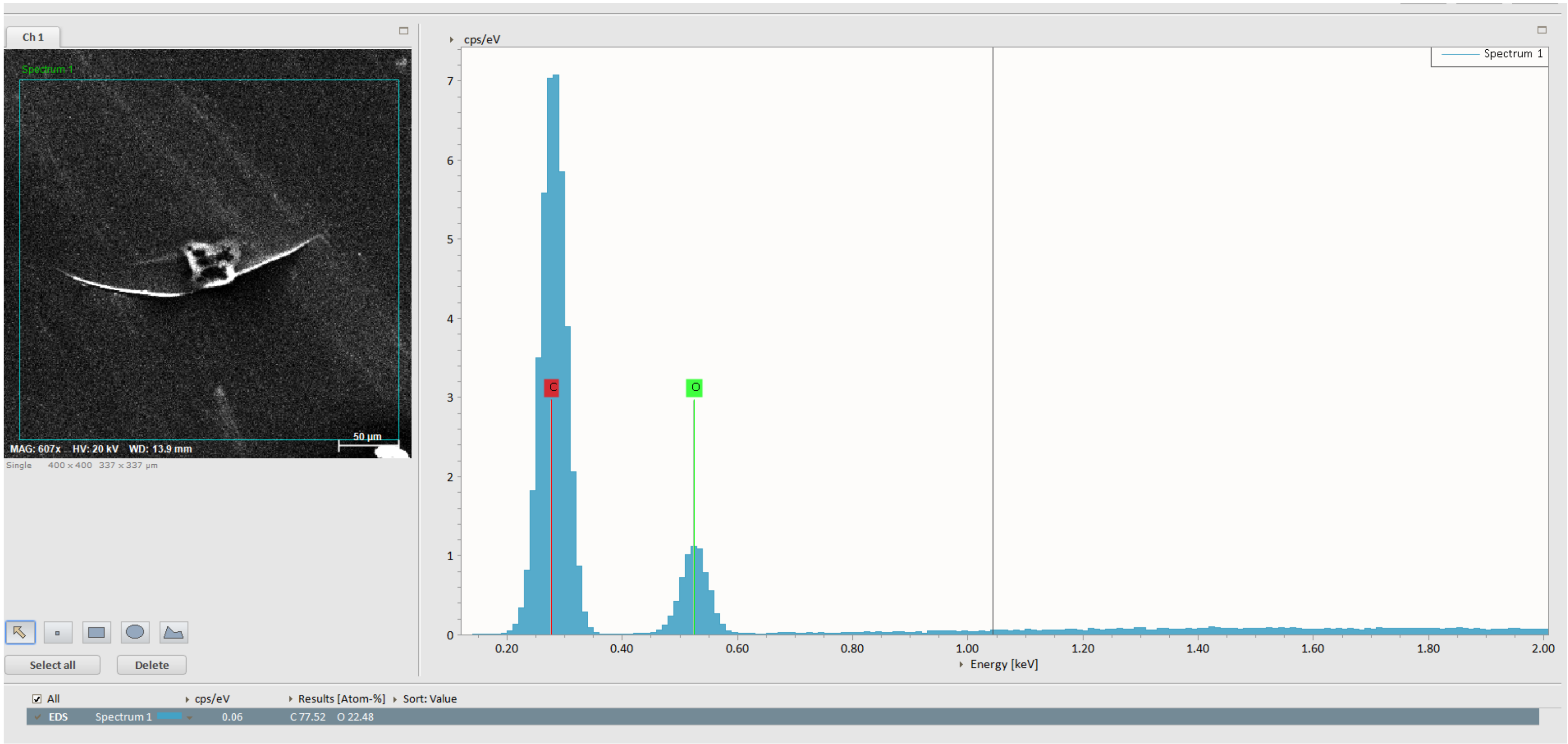

Fig. S-4.1.2. SEM/EDX diagram for BEC - Transparent portion, outside.

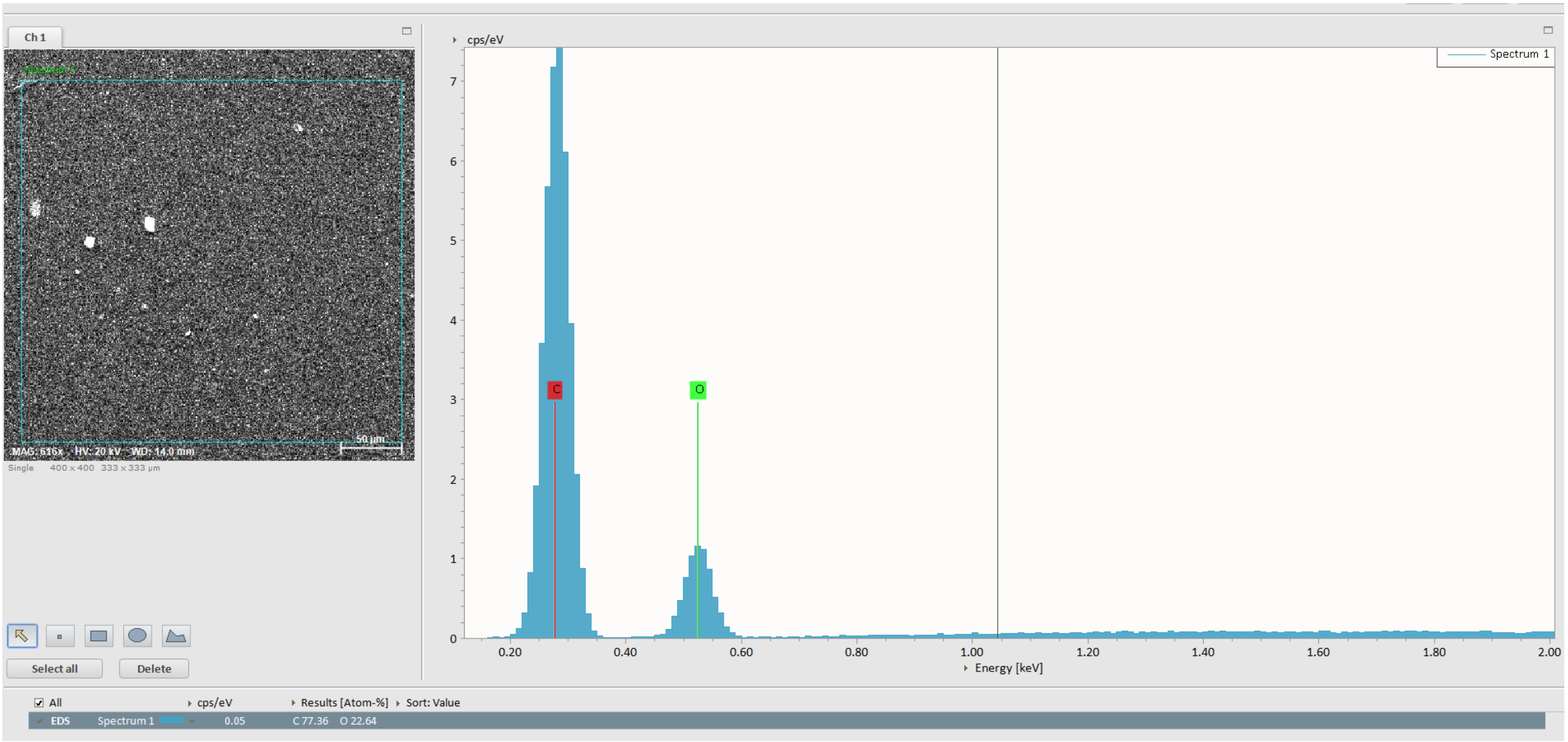

Fig. S-4.2.1. SEM/EDX diagram for BES - Transparent portion, inside.

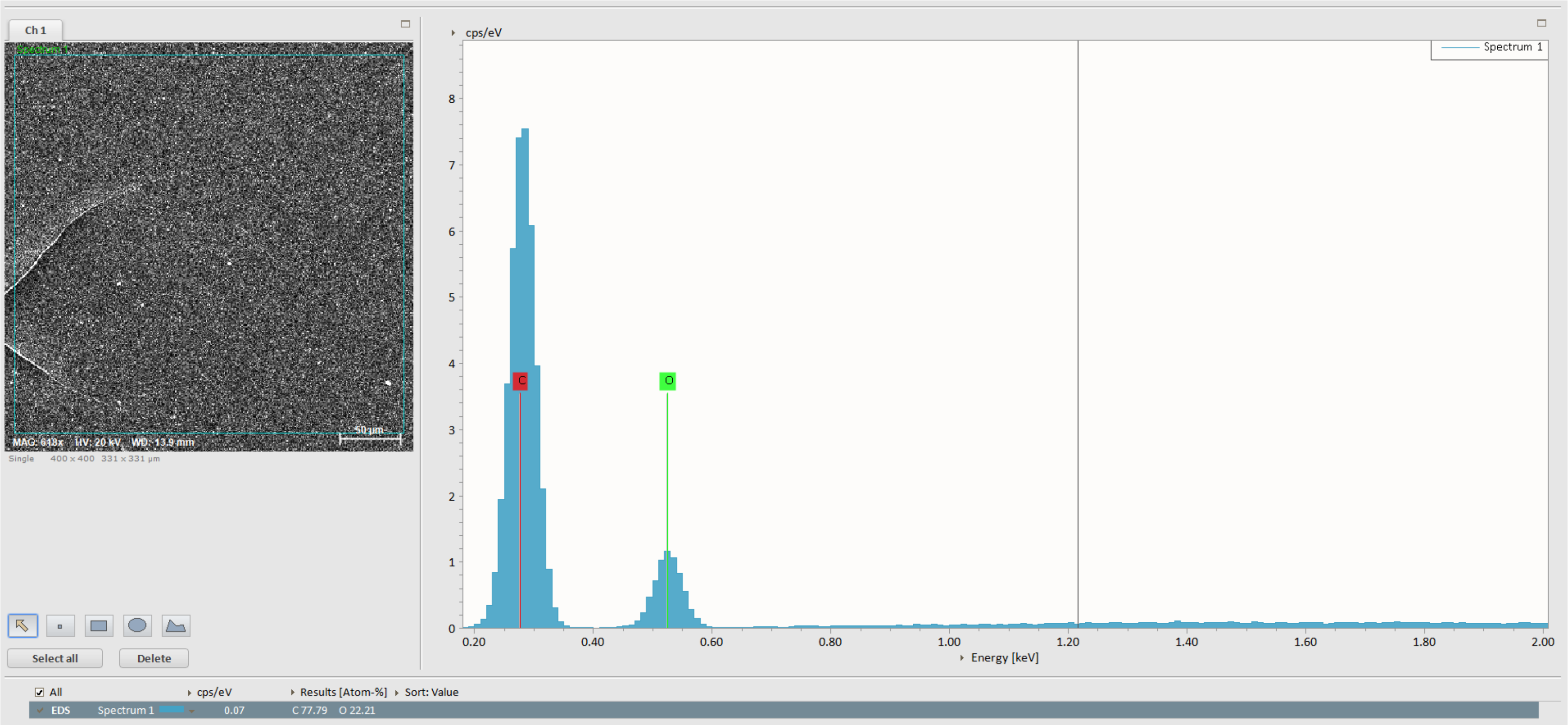

Fig. S-4.2.2. SEM/EDX diagram for BES - Transparent portion, outside.

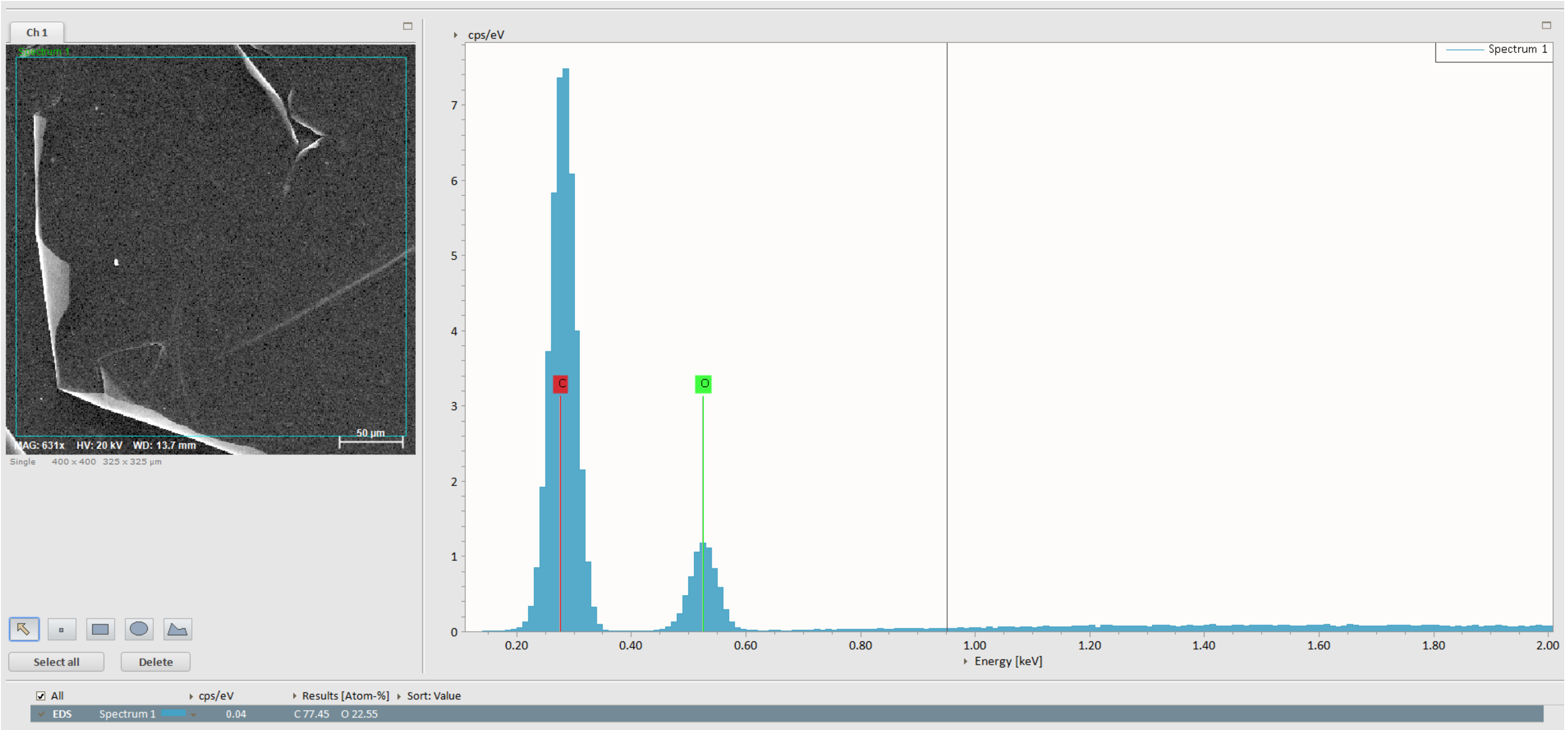

Fig. S-4.3.1. SEM/EDX diagram for CLM - Transparent portion, inside.

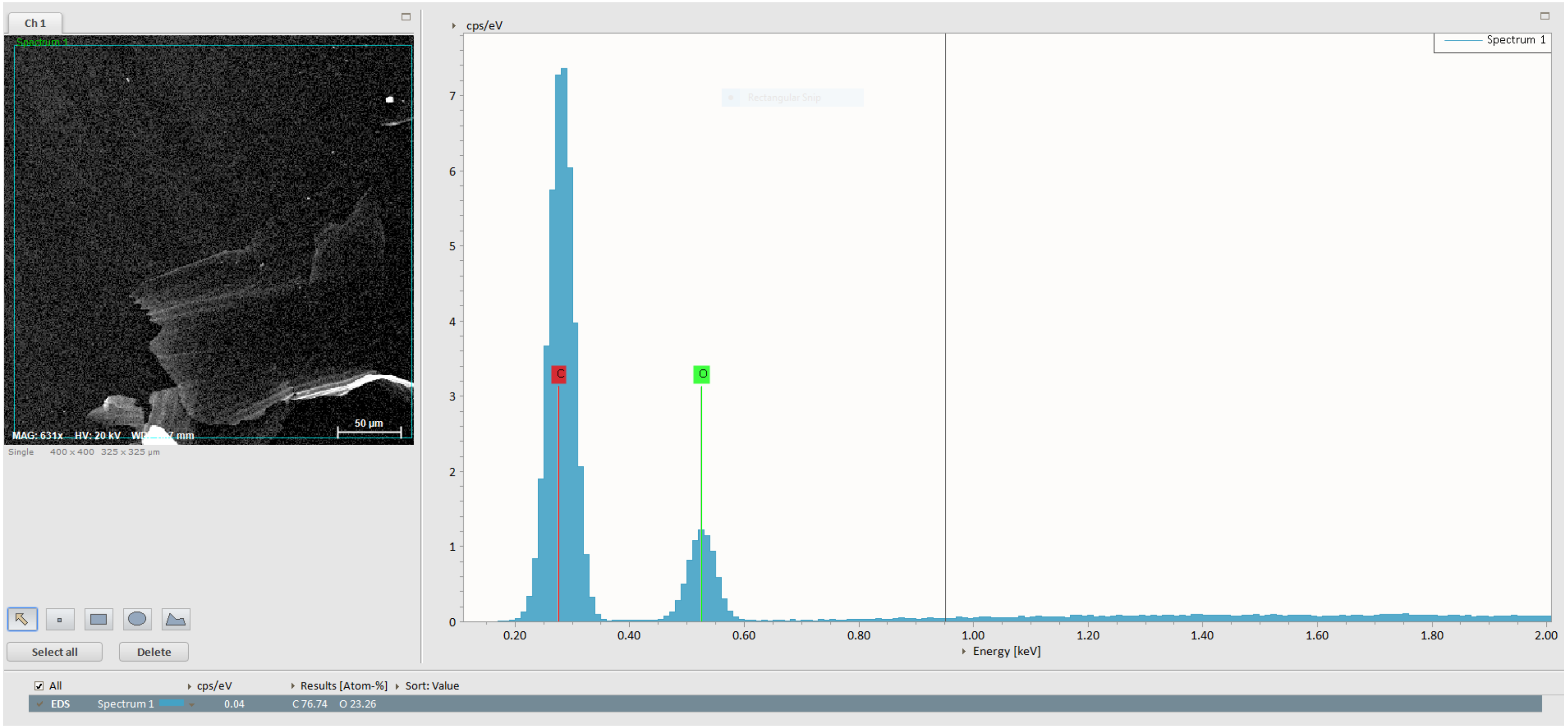

Fig. S-4.3.2 SEM/EDX diagram for CLM - Transparent portion, outside.

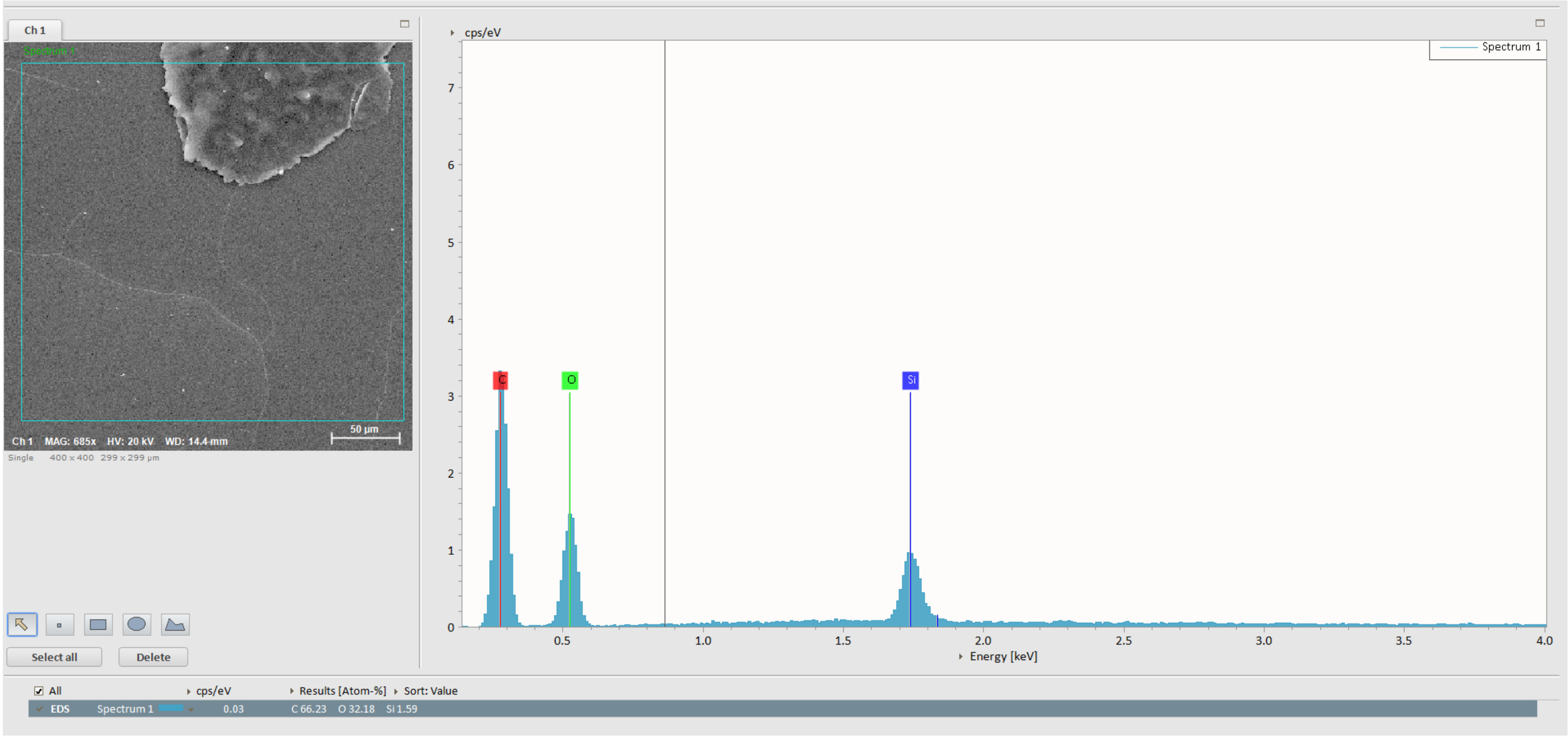

Fig. S-4.4.1. SEM/EDX diagram for FAV - Transparent portion, inside.

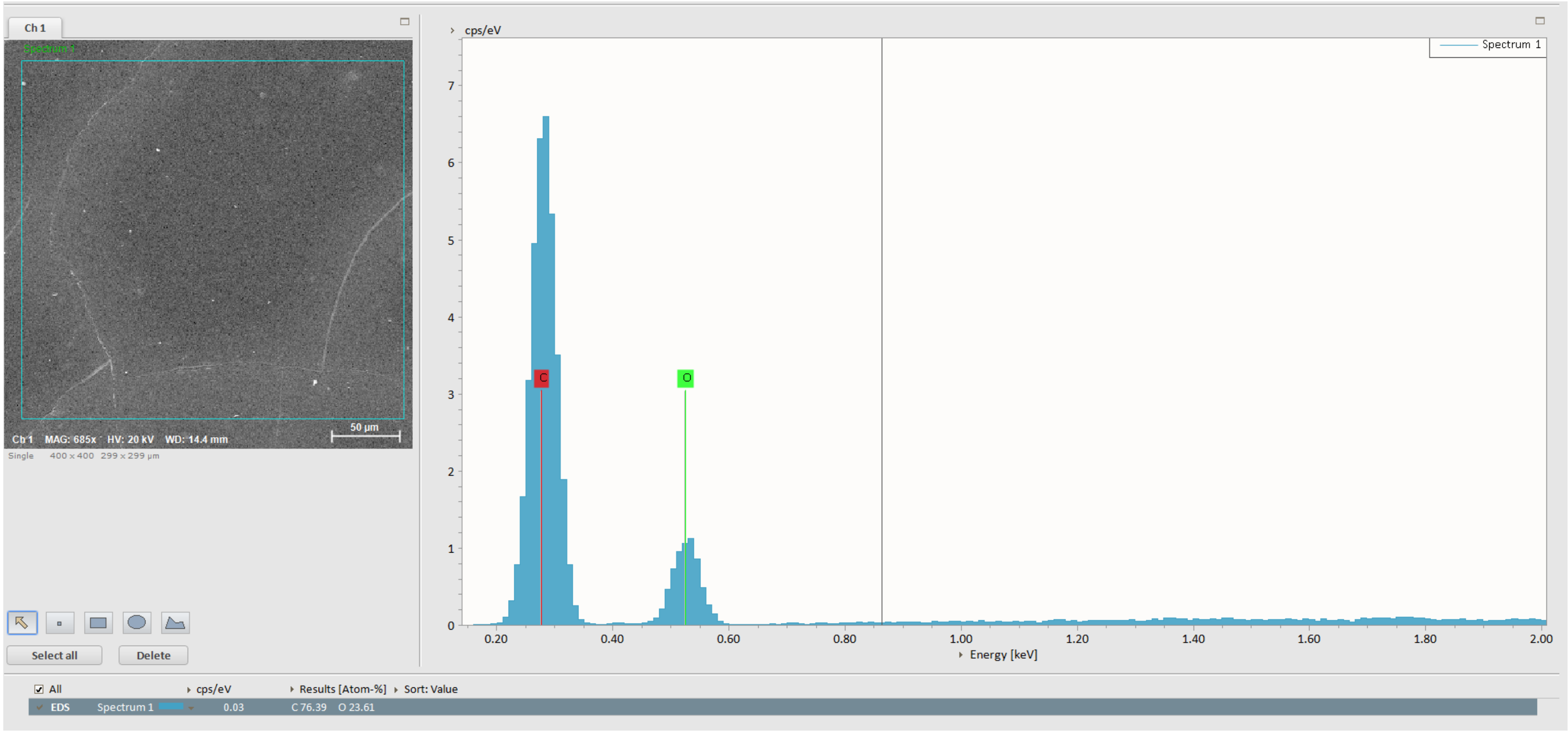

Fig. S-4.4.2. SEM/EDX diagram for FAV - Transparent portion, outside.

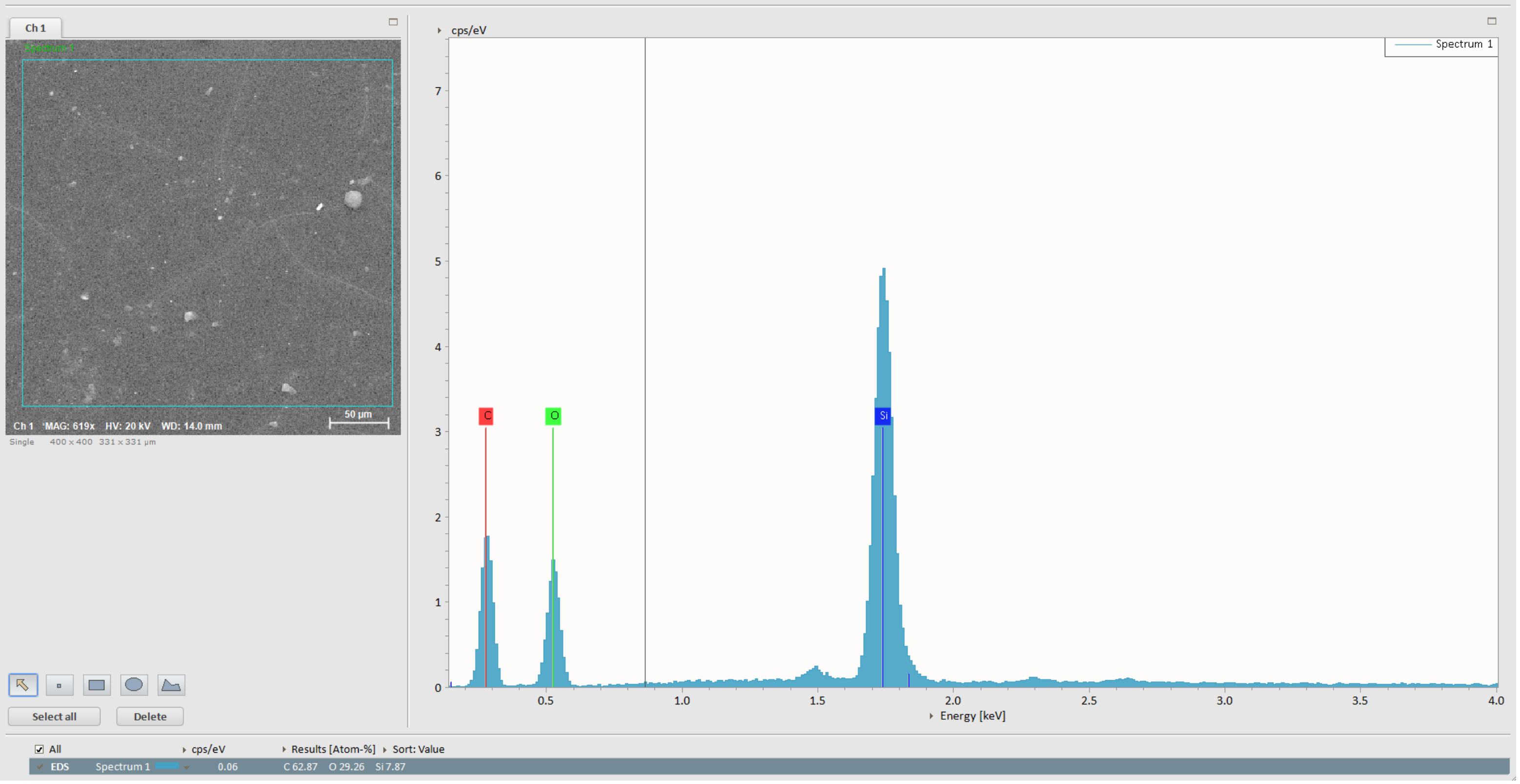

Fig. S-4.5.1. SEM/EDX diagram for JEM - Transparent portion, inside.

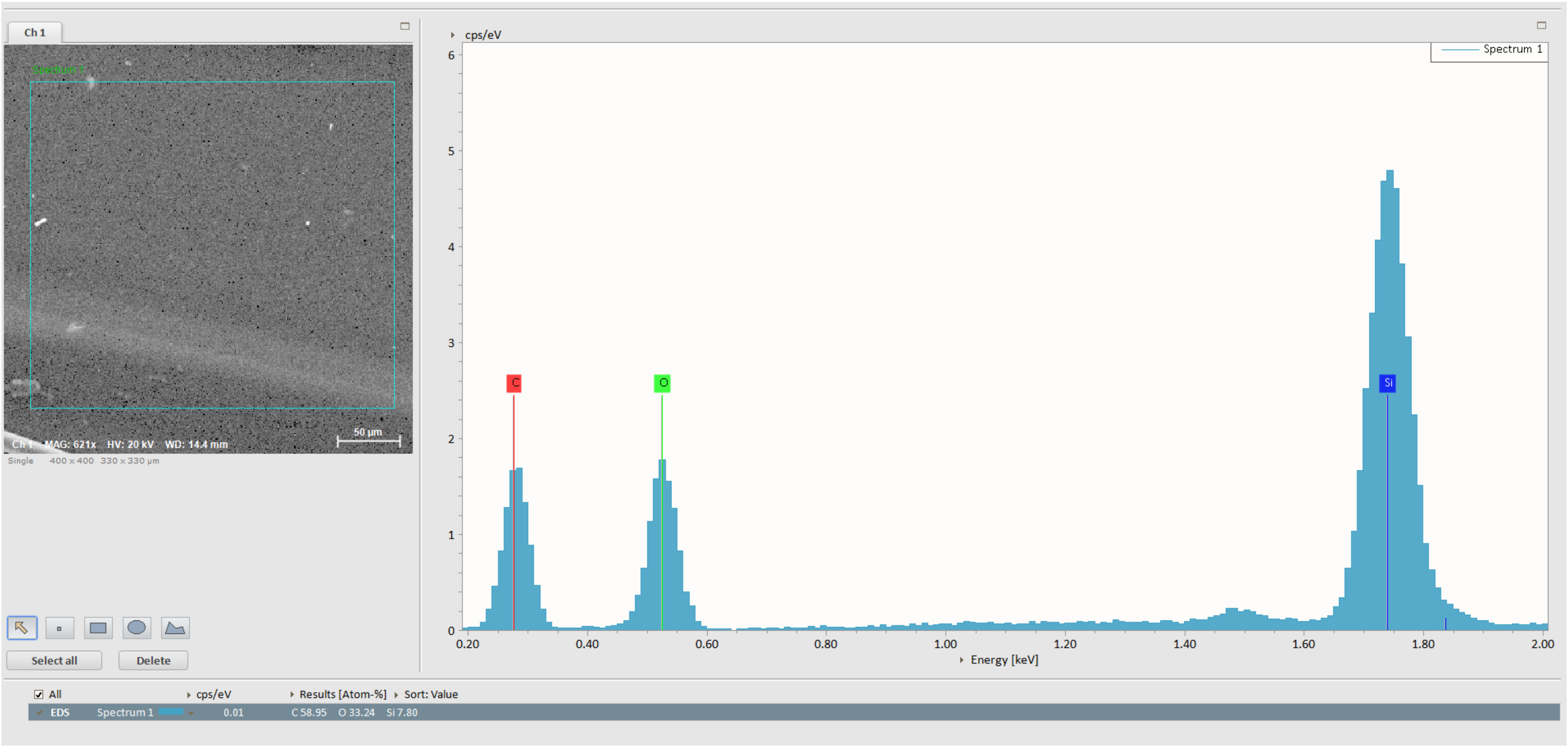

Fig. S-4.5.2 SEM/EDX diagram for JEM - Transparent portion, outside.

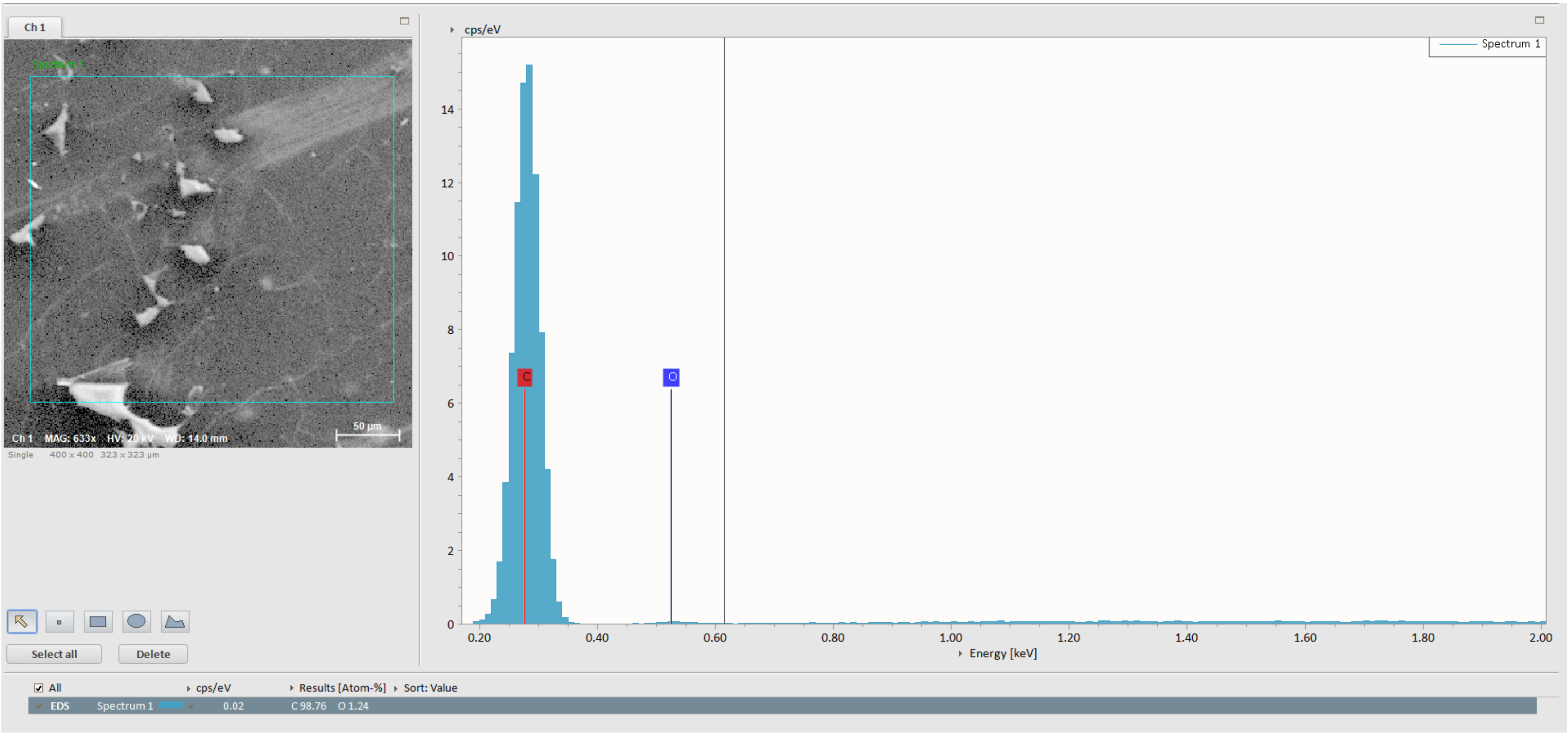

Fig. S-4.6.1. SEM/EDX diagram for OPT - Transparent portion, inside.

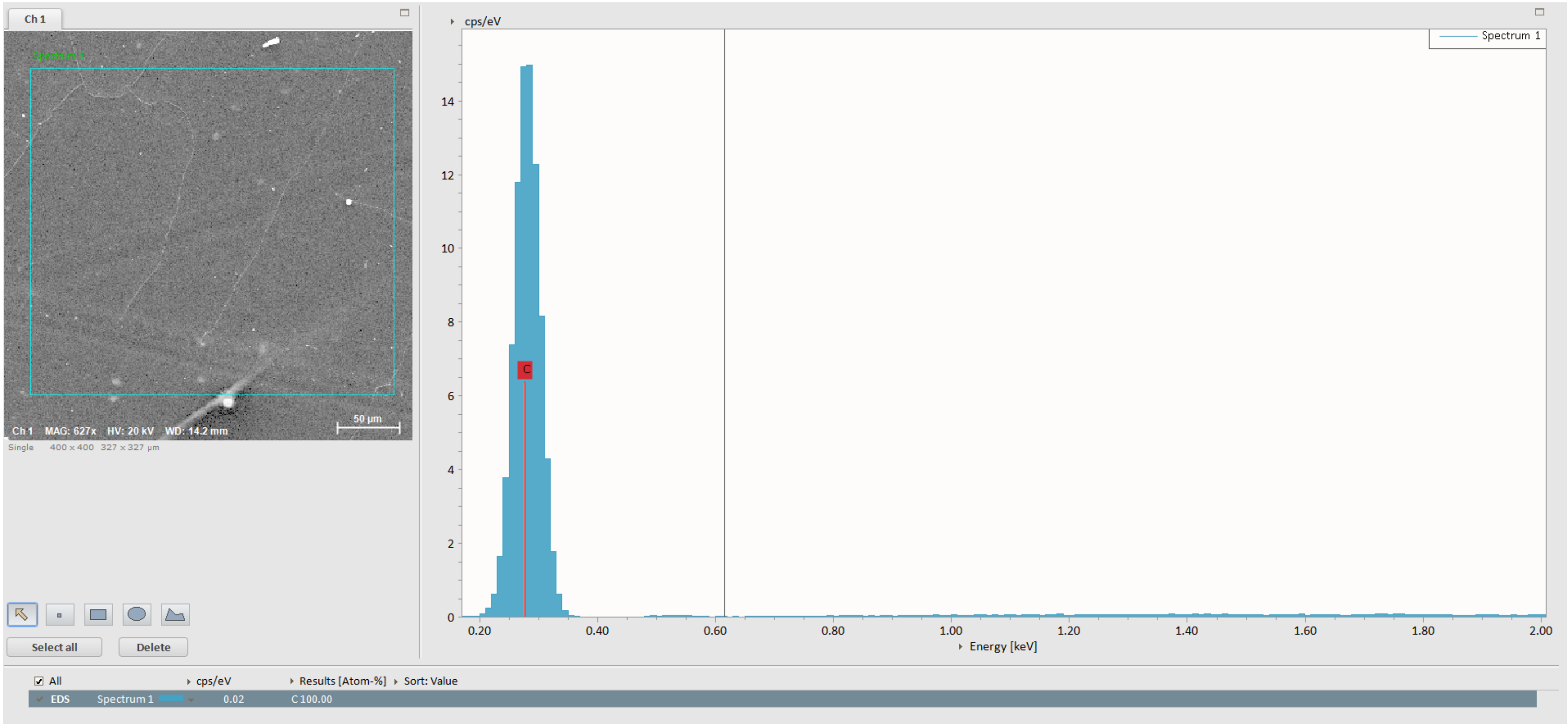

Fig. S-4.6.2. SEM/EDX diagram for OPT - Transparent portion, outside.

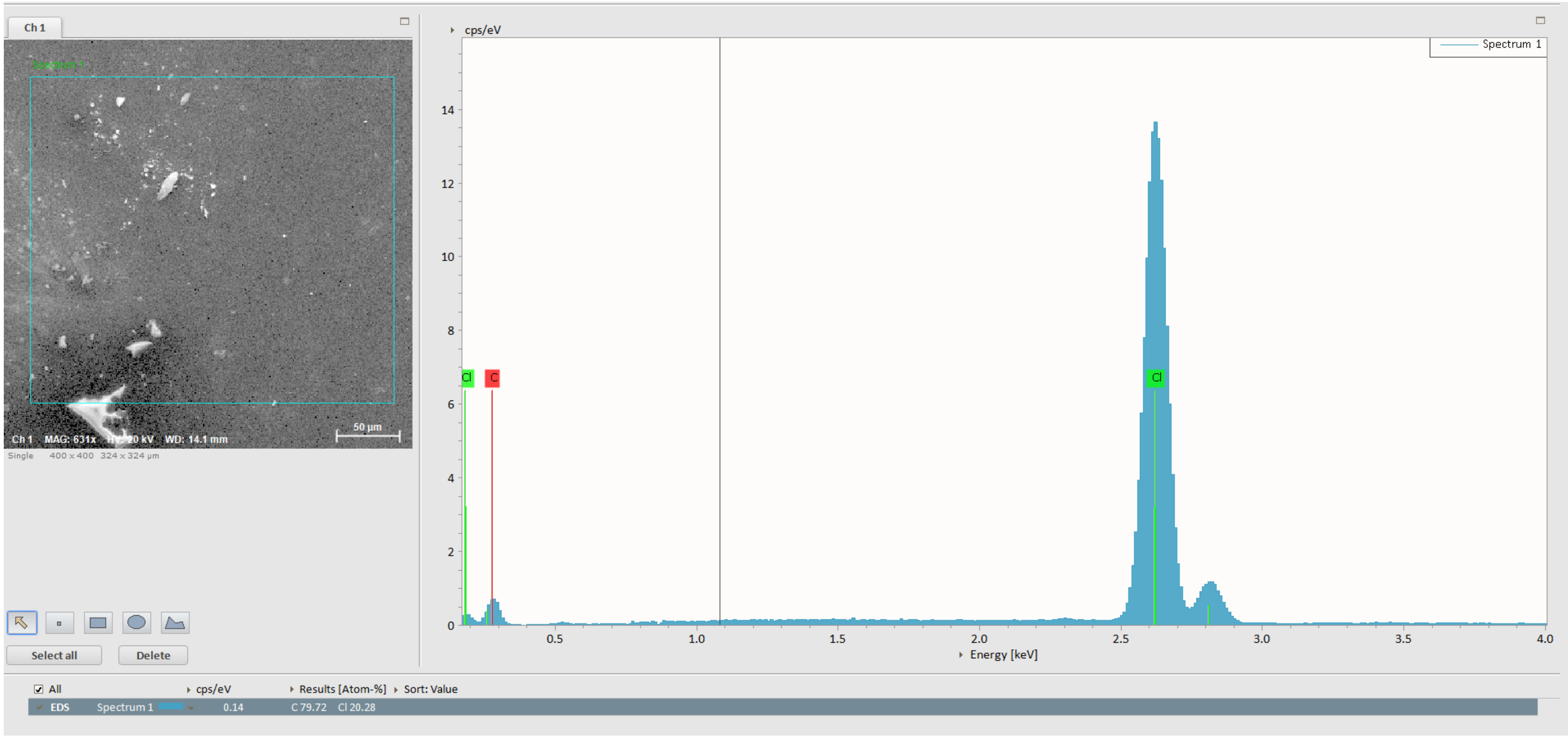

Fig. S-4.7.1. SEM/EDX diagram for RAN - Transparent portion, inside.

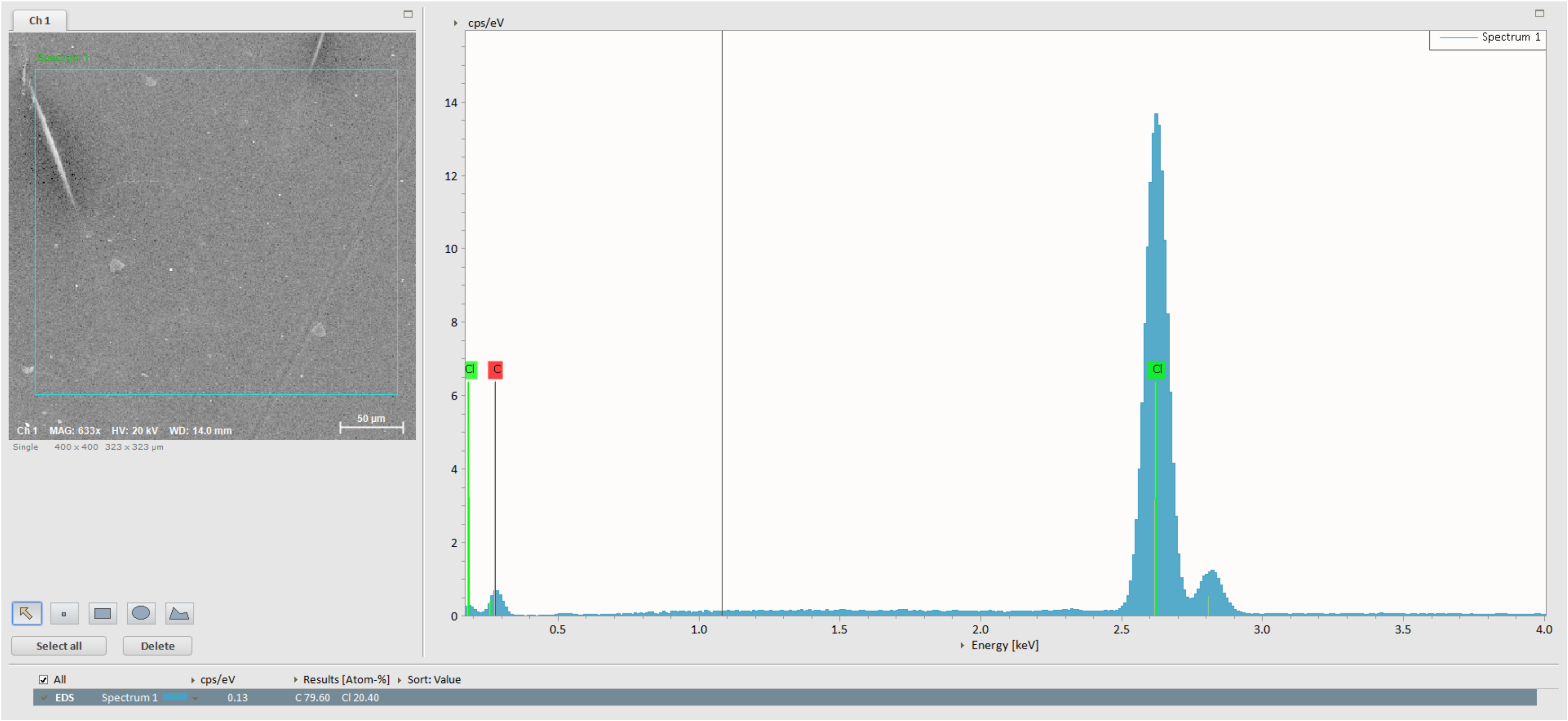

Fig. S-4.7.2. SEM/EDX diagram for RAN - Transparent portion, outside.

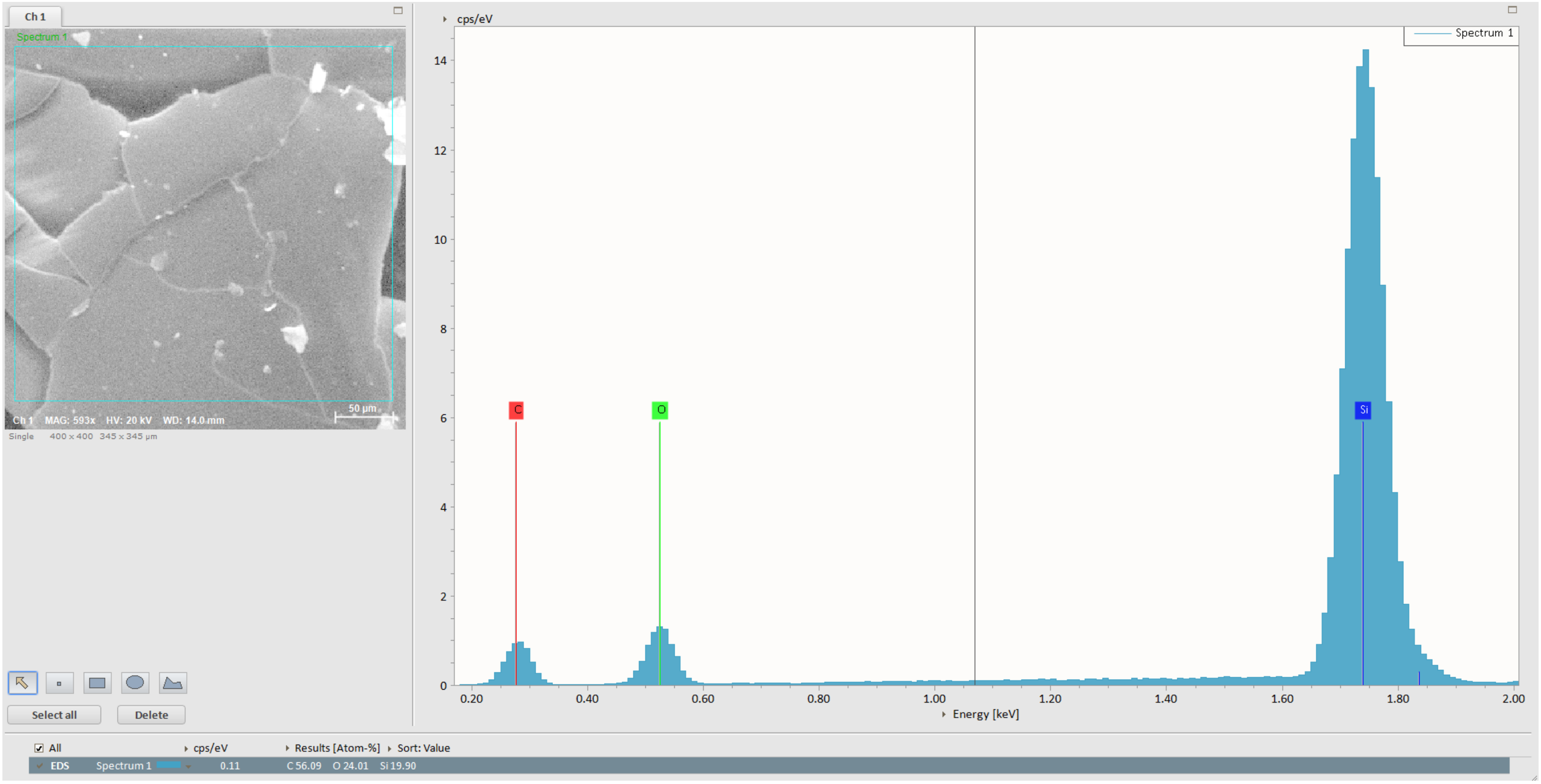

Fig. S-4.8.1. SEM/EDX diagram for SEU - Transparent portion, inside.

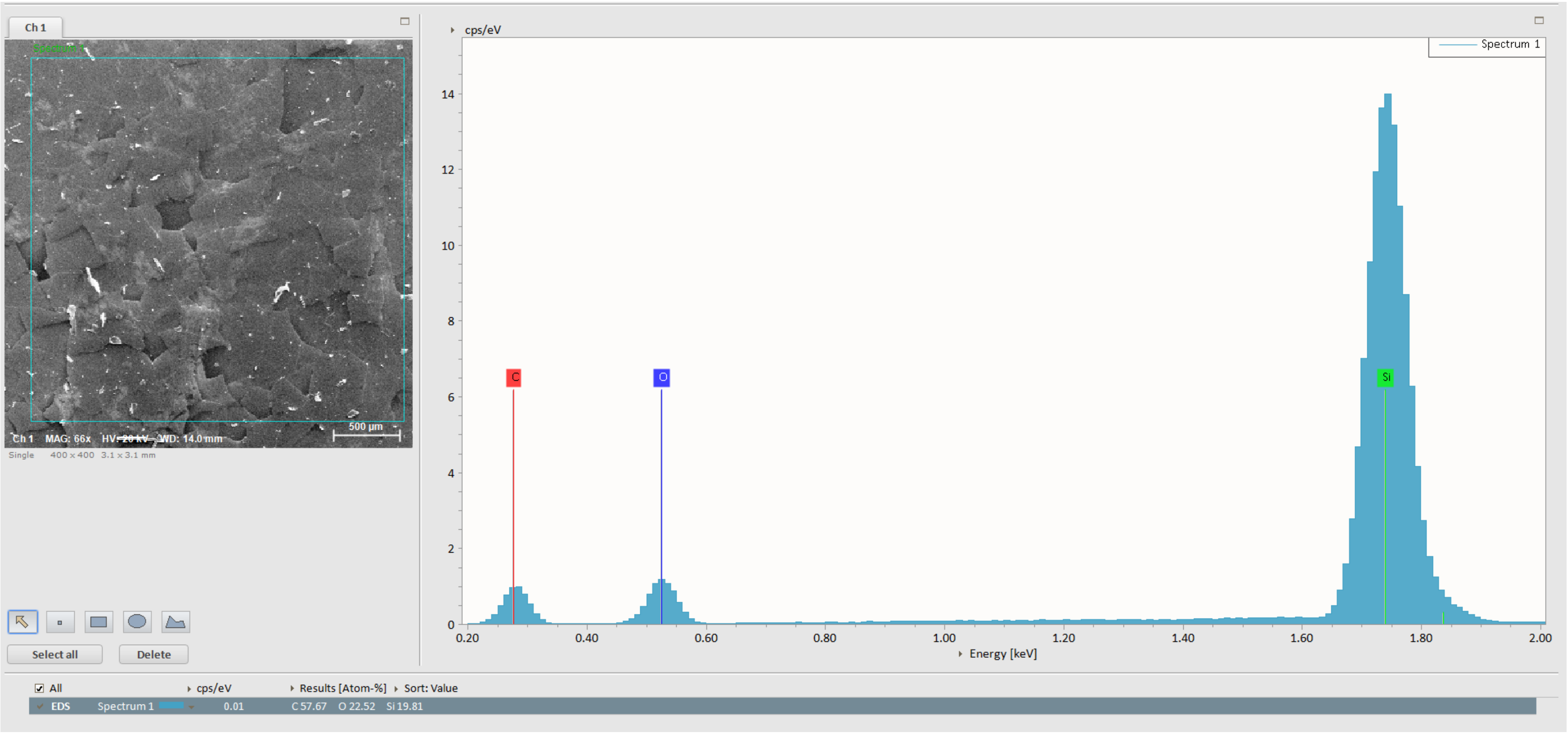

Fig. S-4.8.2. SEM/EDX diagram for SEU - Transparent portion, outside.

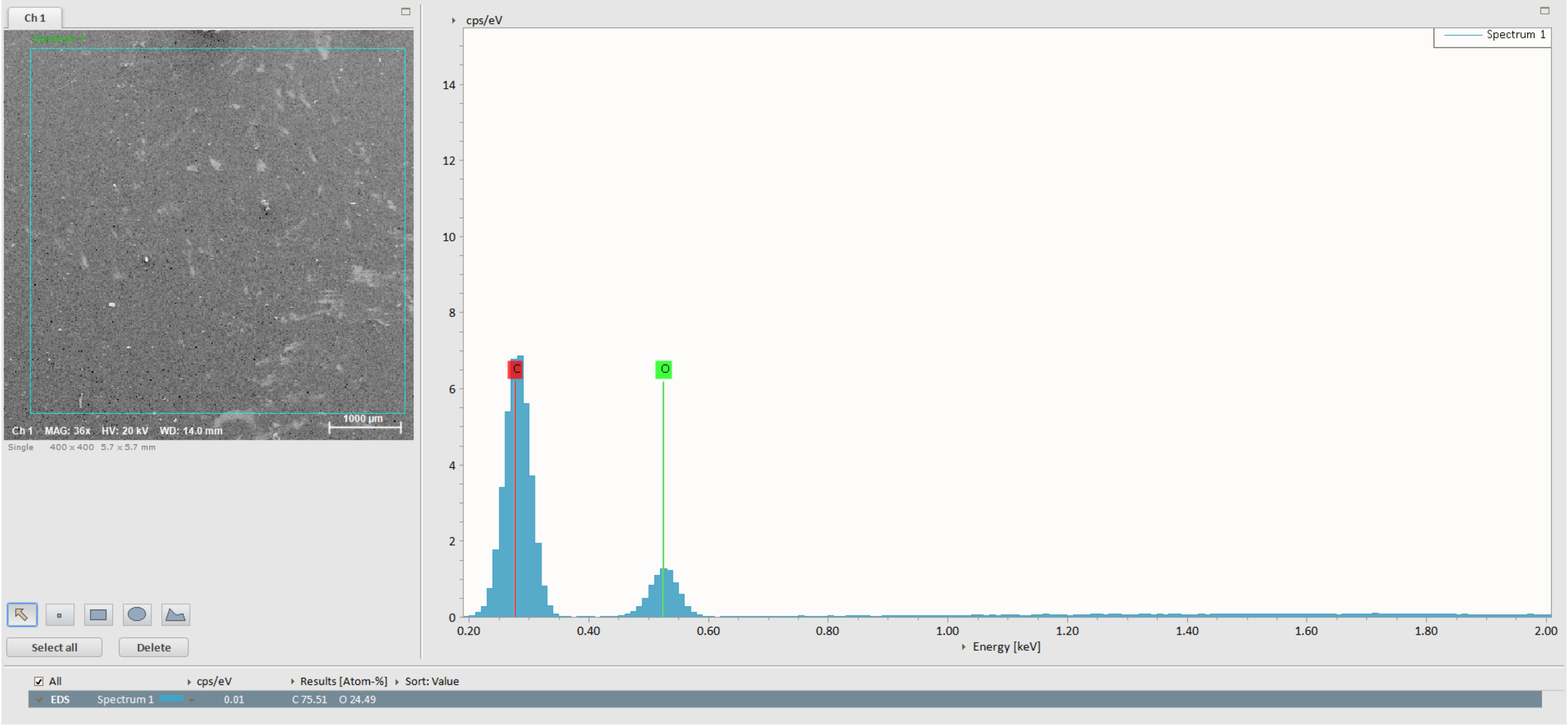

Fig. S-4.9.1 SEM/EDX diagram for SNC - Transparent portion, inside.

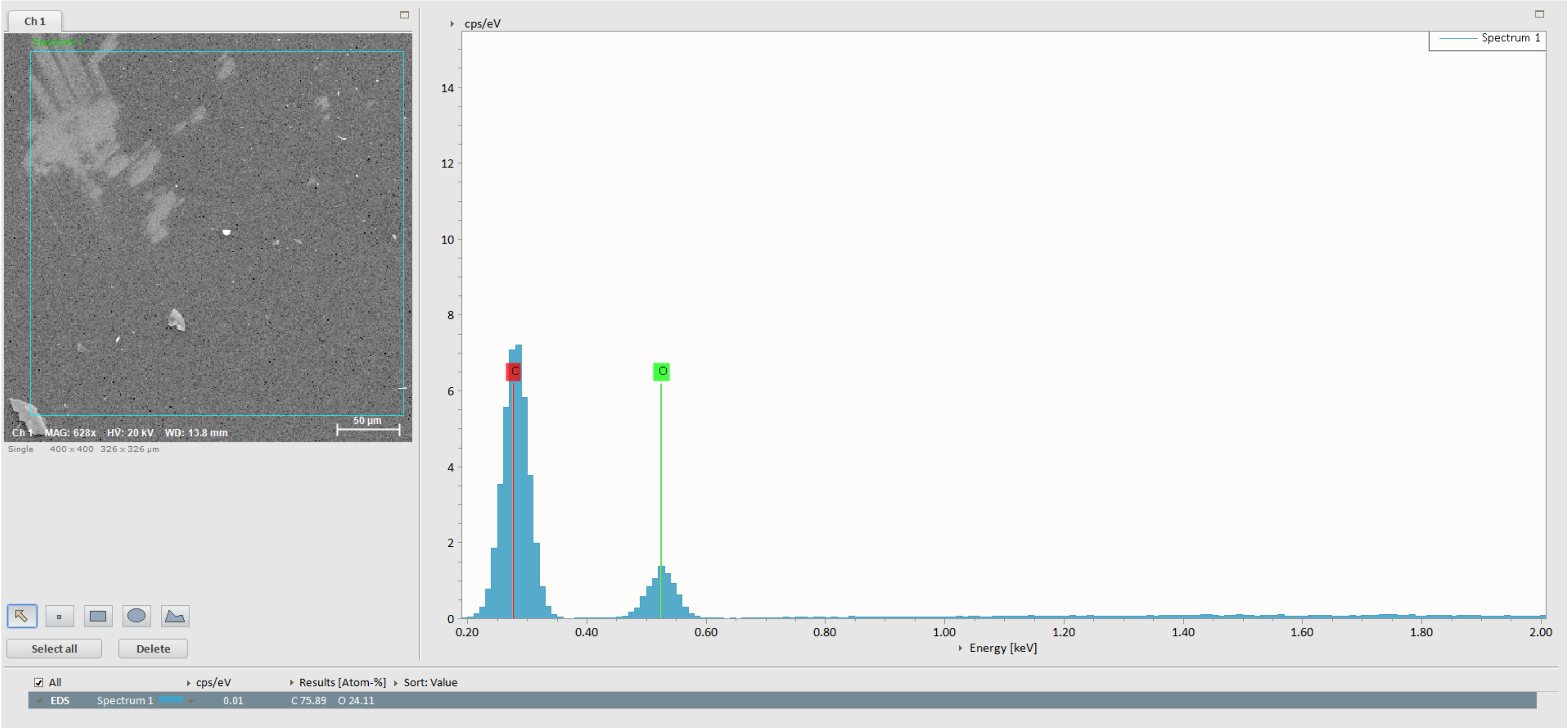

Fig. S-4.9.2. SEM/EDX diagram for SNC - Transparent portion, outside.

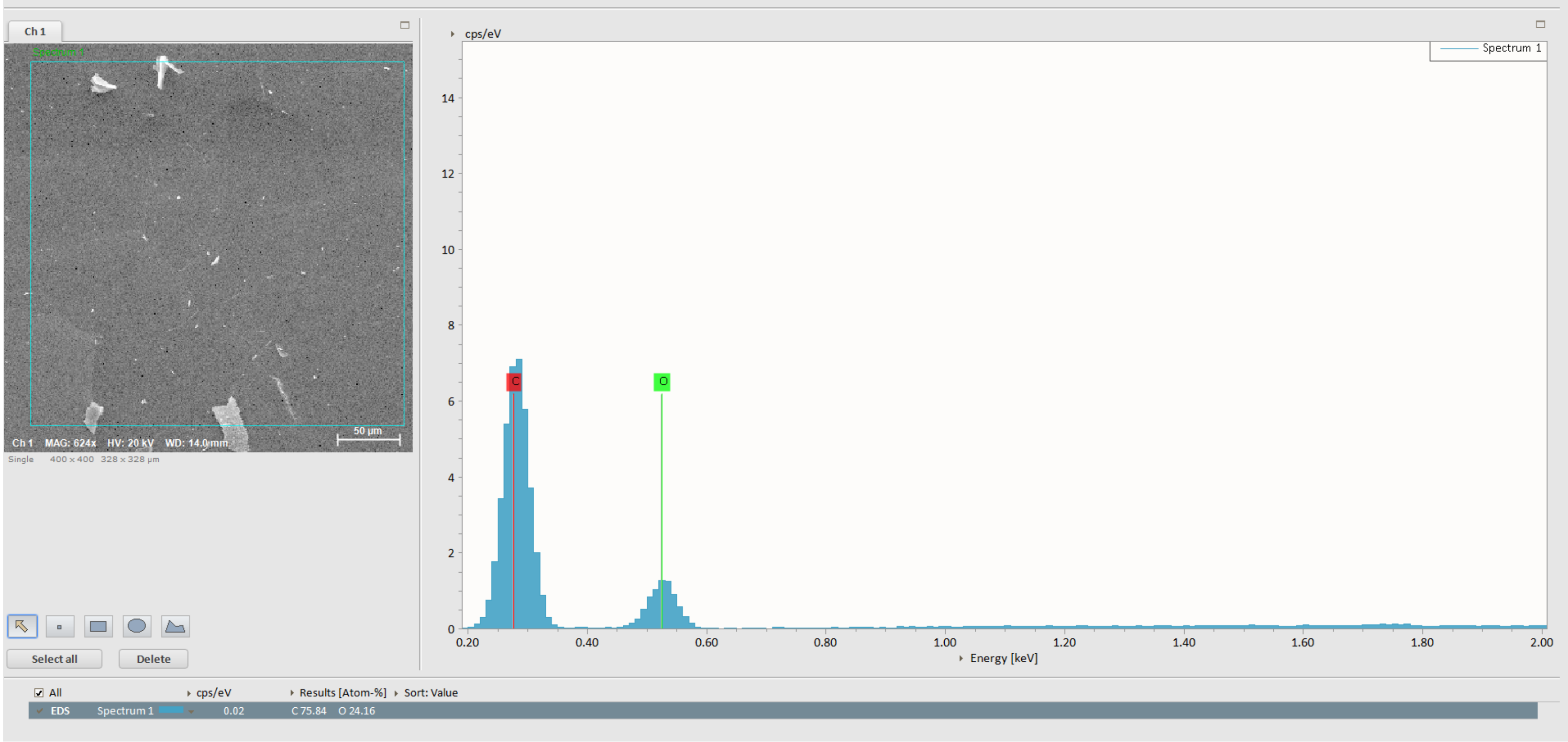

Fig. S-4.10.1. SEM/EDX diagram for STK - Transparent portion, inside.

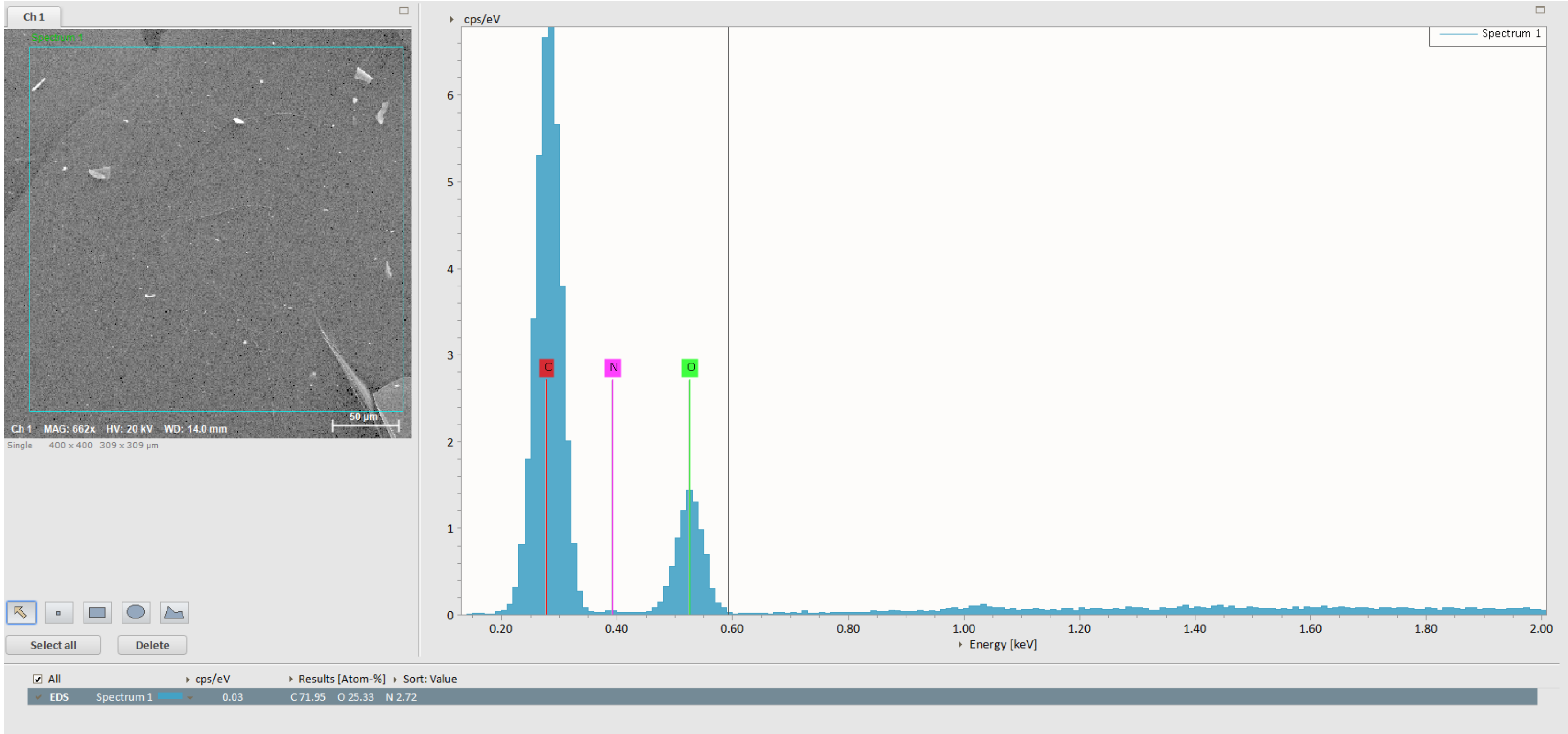

Fig. S-4.10.2. SEM/EDX diagram for STK - ransparent portion, outside.

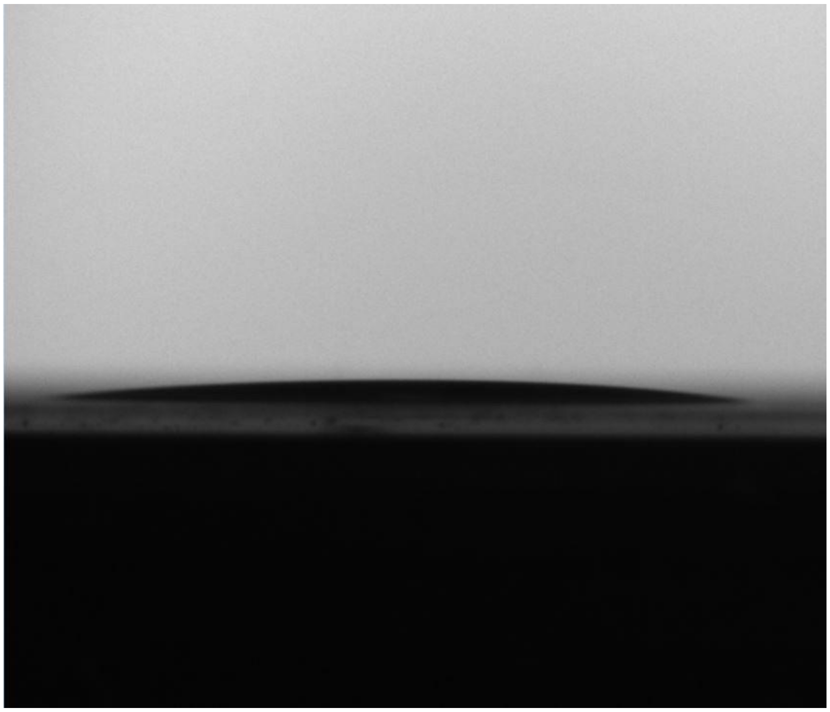

Fig. S-6. Photo of a water contact angle measurement of STK, - Transparent portion, inside.

(The measured value of less than 10° was relatively small and indicated a very high hydrophilicity).

Table S-1.1 Contact Angle Data – BEC and BES (CLM not included here, see main article)

Mask	Side	Left	Right	Mean	Height	Width
BEC	Outside	72.8	73.3	73.1	1.095	2.008
		75.5	75.5	75.5	1.19	2.006
		74.9	74.9	74.9	1.18	2.013
		71.5	71.6	71.5	1.034	1.976
		71.4	71.5	71.4	1.034	1.985
	Inside	72.7	72.1	72.4	1.129	2.161
		72.3	72	72.2	1.133	2.165
		80.4	79.5	79.9	1.607	2.285
		79.1	78.6	78.9	1.553	2.294
		70.5	71.6	71	1.072	2.168
BES	Outside	61.1	60.2	60.7	0.979	2.687
		58.8	60	59.4	0.95	2.5
		72.9	75.1	74	1.396	2.449
		52.9	49.1	51	0.91	3.164
		53.2	56.1	54.7	0.917	3.008
	Inside	56.3	54.9	55.6	0.891	2.873
		51.9	54.8	53.4	0.902	3.091
		61.7	59.6	60.6	1.121	3.057
		65.4	69.2	67.3	1.323	2.718
		75.6	75.1	75.4	1.445	2.425
CLM	(see main article)	*	*	*	*	*

Table S-1.2 Contact Angle Data – FAV

Mask	Side	Left	Right	Mean	Height	Width
FAV	Outside	72.2	72.3	72.3	1.65	3.196
		72	72.2	72.1	1.646	3.199
		72	72.1	72	1.642	3.2
		71.8	72.1	72	1.637	3.201
		71.8	72	71.9	1.635	3.203
	Inside	12	13.3	12.6	0.271	5.76
		11.8	13	12.4	0.281	5.944
		10.7	11.3	11	0.244	6.048
		10	12.6	11.3	0.237	5.919
		11.1	13.5	12.3	0.278	6.392
		11	12.3	11.6	0.233	6.26

Table S-1.3 Contact Angle Data – JEM and OPT

Mask	Side	Left	Right	Mean	Height	Width
JEM	Outside	23.6	20.7	22.2	0.347	3.857
		18	20.5	19.2	0.296	3.399
		17.5	20.7	19.1	0.29	3.394
		17.3	20.7	19	0.287	3.395
		17	20.6	18.8	0.283	3.391
	Inside	20.6	20.8	20.7	0.193	2.081
		19.2	19.2	19.2	0.175	2.052
		18.5	18.3	18.4	0.167	2.052
		17.5	17.6	17.6	0.156	2.034
		14.5	15.7	15.1	0.134	2.165
OPT	Outside	57.9	59	58.4	1.041	3.043
		52	53.9	52.9	0.829	2.794
		51.4	53.3	52.3	0.813	2.802
		57	55.1	56	0.906	2.925
		48.3	48.1	48.2	0.73	2.924
	Inside	54.9	58.9	56.9	0.999	3.372
		53.4	54.4	53.9	0.963	3.424
		47.5	51.2	49.3	0.924	3.403
		52.3	54.1	53.2	0.961	3.481
		52	53.7	52.9	0.954	3.479

Table S-1.4 Contact Angle Data – RAN and SEU

Mask	Side	Left	Right	Mean	Height	Width
RAN	Outside	75.8	77	76.4	1.501	2.435
		72.6	73.6	73.1	1.415	2.596
		78.3	79.1	78.7	1.532	2.336
		77.4	78.1	77.8	1.478	2.335
		79.8	76.2	78	1.498	2.317
	Inside	75	77.3	76.1	1.424	2.336
		78.2	78.4	78.3	1.423	2.191
		75.4	77.6	76.5	1.434	2.311
		72.1	73.3	72.7	1.335	2.466
		79.1	80	79.6	1.546	2.292
SEU	Outside	80.4	80.1	80.2	1.456	2.134
		91.9	92.4	92.1	1.952	1.878
		91.8	91.8	91.8	1.947	1.902
		88.6	88.6	88.6	1.844	2.005
		88.4	87.8	88.1	1.816	2.02
	Inside	87.4	87.2	87.3	1.674	1.91
		87.3	87.2	87.2	1.671	1.911
		87.3	87.2	87.2	1.669	1.911
		87.1	87.3	87.2	1.718	1.949
		87	87.2	87.1	1.718	1.951

Table S-1.4 Contact Angle Data – SNC and STK

Mask	Side	Left	Right	Mean	Height	Width
		53.9	55.8	54.8	0.984	3.501
		48.4	47.7	48	0.864	4.06
		55.6	53.3	54.4	0.882	2.71
	Inside	42.3	43.8	43.1	0.855	3.869
		44.3	42.2	43.3	0.797	3.492
		46.2	49.3	47.8	0.796	3.137
		43.8	41.8	42.8	0.706	3.308
		45.9	47.5	46.7	0.732	3.054
STK	Outside	67.7	67.4	67.5	1.068	2.312
		77.1	76.8	77	1.521	2.448
		72.7	72.7	72.7	1.351	2.482
		78	77.9	78	1.556	2.443
		77.4	77.2	77.3	1.595	2.564
		77	76.7	76.9	1.573	2.532
	Inside	10.2	10.9	10.6	0.164	3.936
		10.4	10	10.2	0.126	2.925
		9.8	10.4	10.1	0.118	2.847
		10.5	10.1	10.3	0.101	2.263
		9.6	9.4	9.5	0.094	2.231

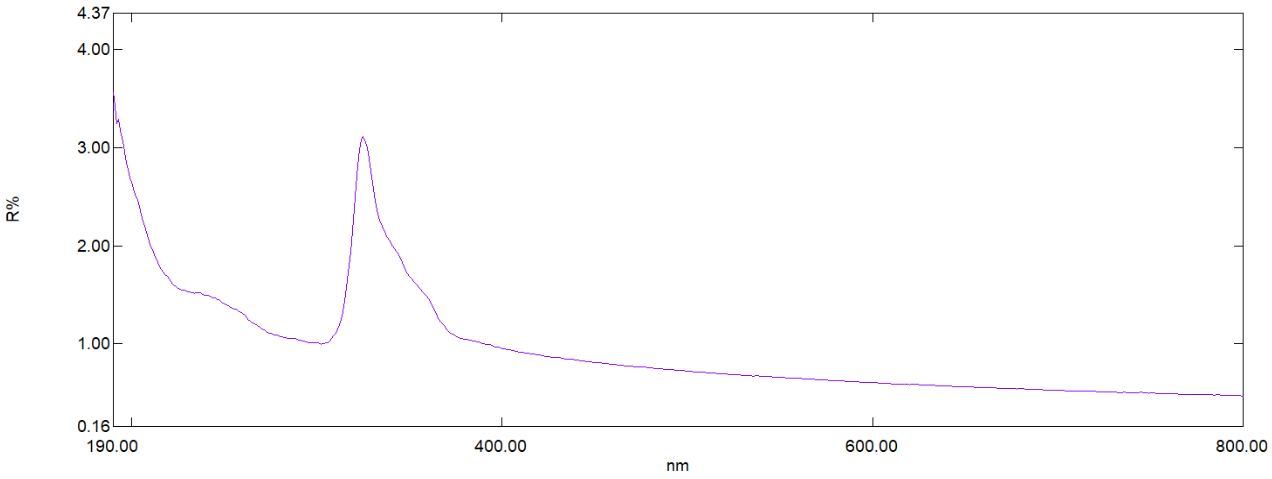

Fig. S-5.1. UV-Vis Reflectance Graph of BEC.

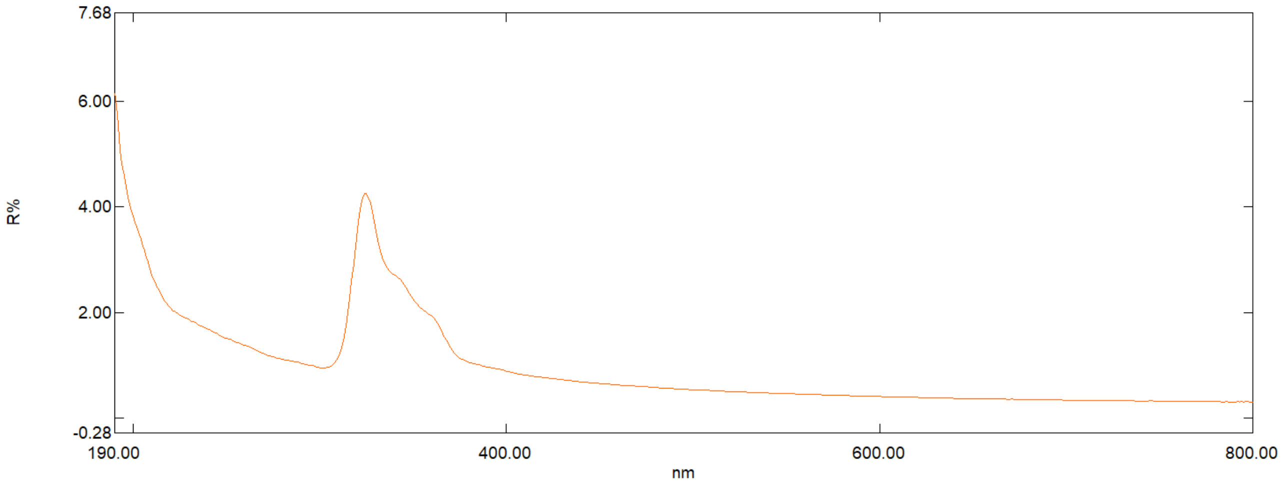

Fig. S-5.2 . UV-Vis Reflectance Graph of BES.

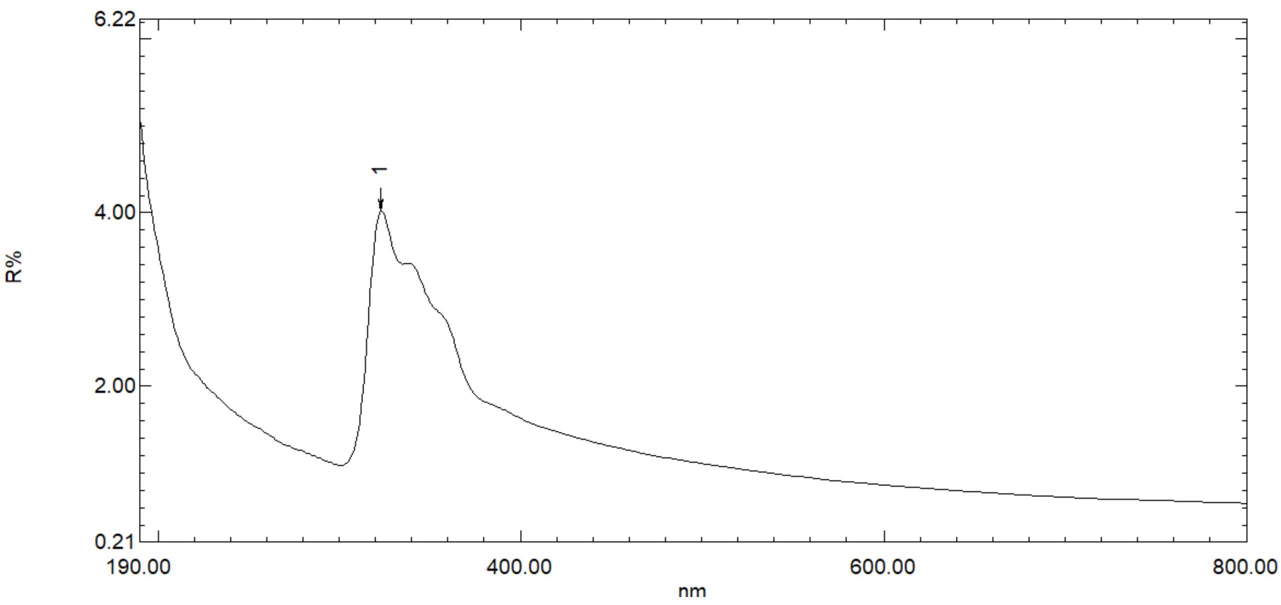

Fig. S-5.3. UV-Vis Reflectance Graph of CLM.

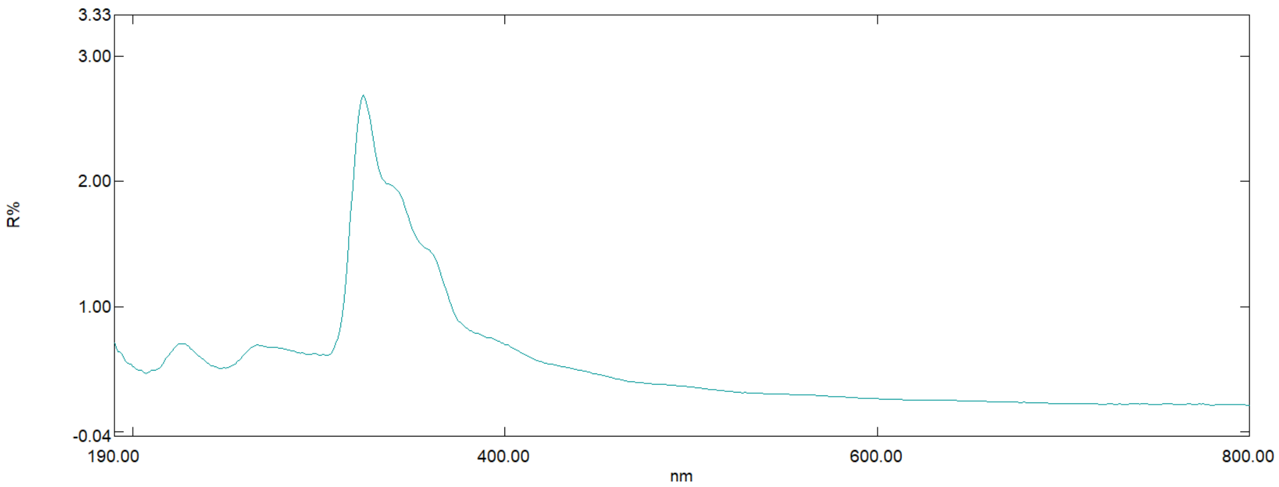

Fig. S-5.4. UV-Vis Reflectance Graph of FAV.

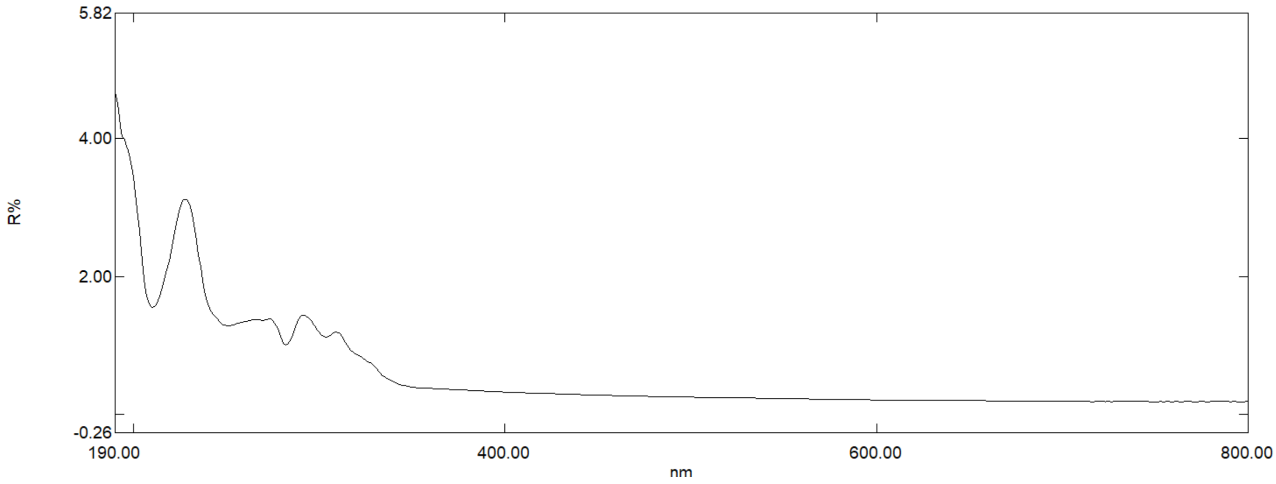

Fig. S-5.5. UV-Vis Reflectance Graph of JEM.

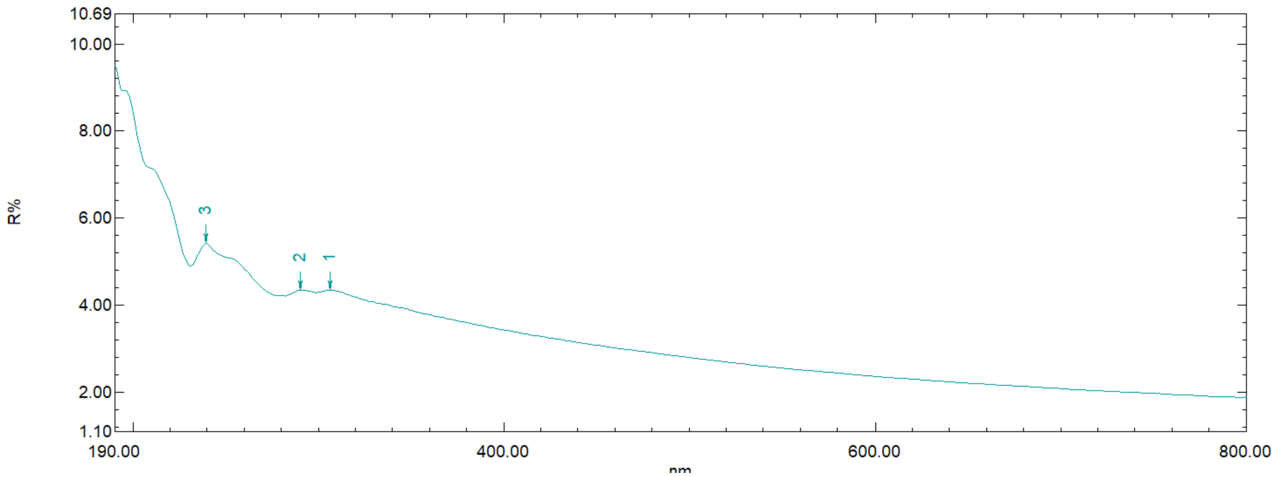

Fig. S-5.6. UV-Vis Reflectance Graph of OPT.

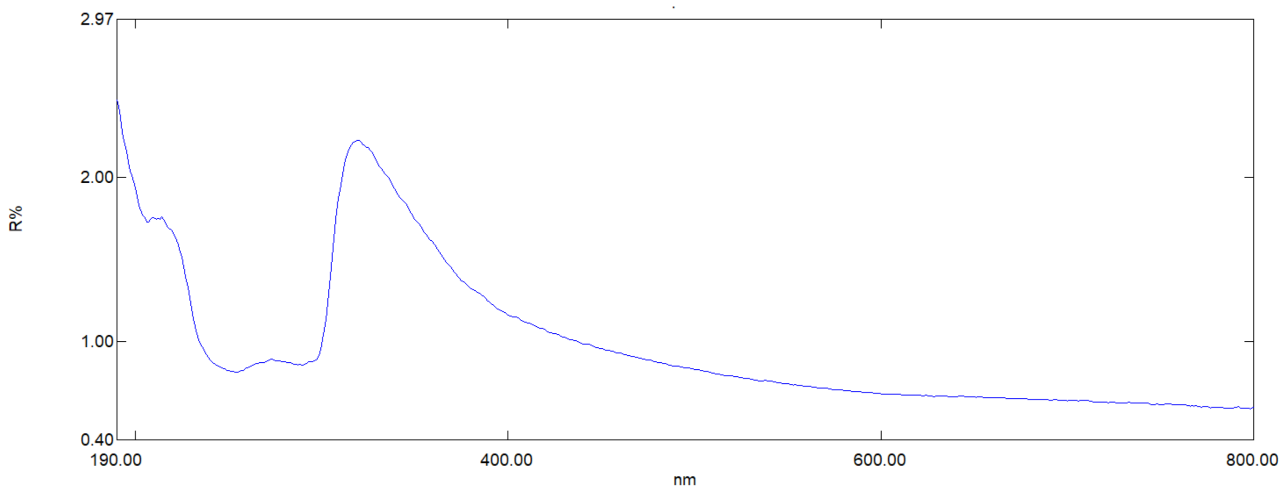

Fig. S-5.7. UV-Vis Reflectance Graph of RAN.

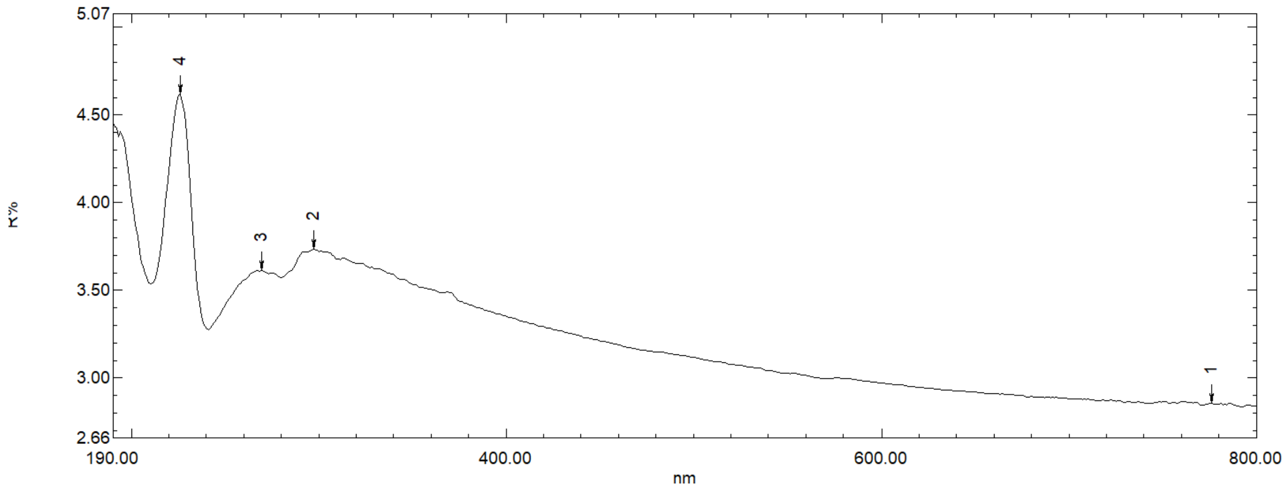

Fig. S-5.8. UV-Vis Reflectance Graph of SEU.

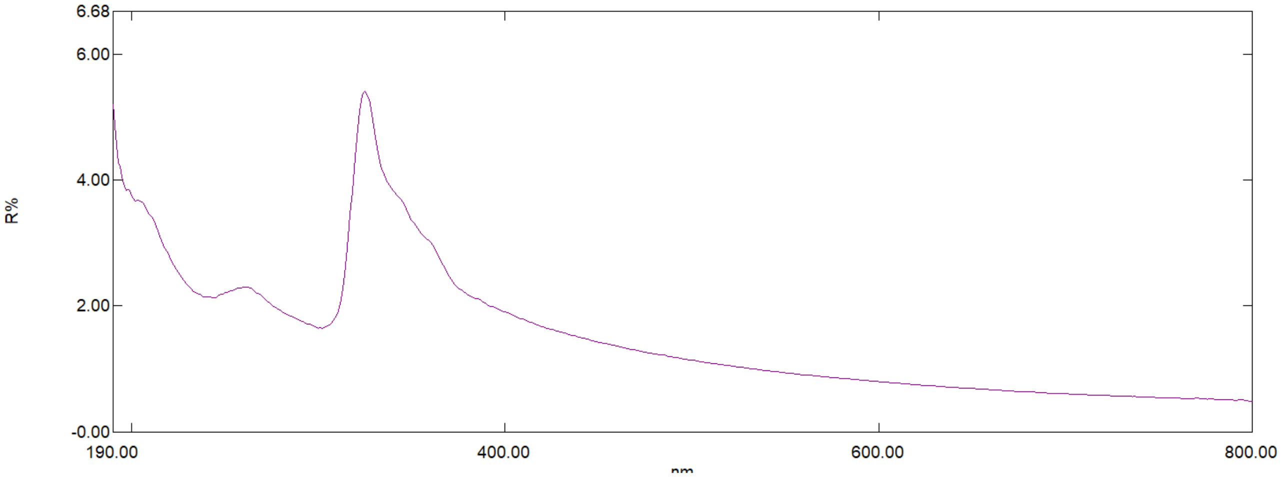

Fig. S-5.9. UV-Vis Reflectance Graph of SNC.

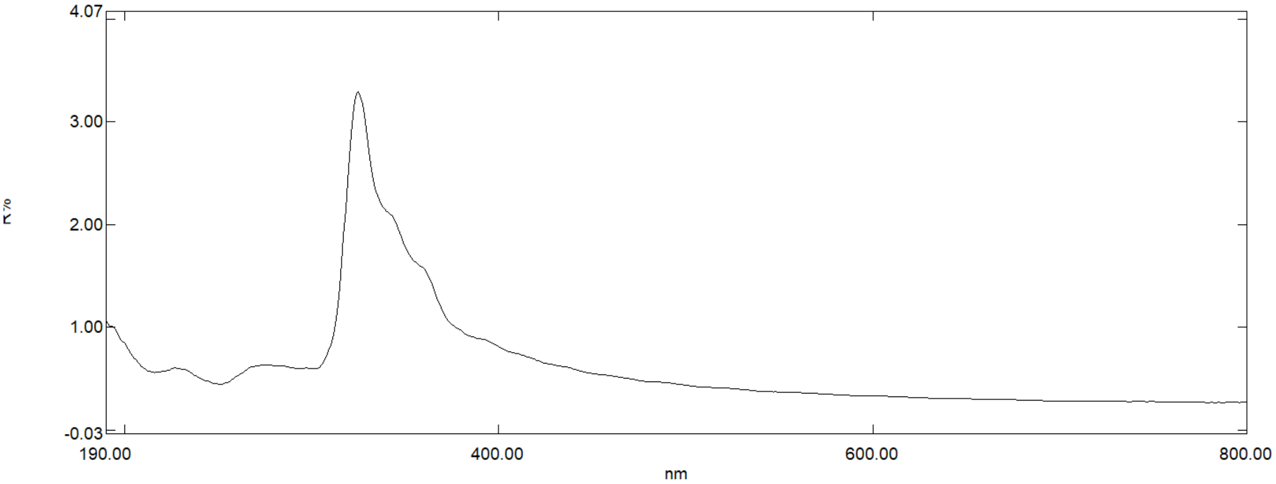

Fig. S-5.10. UV-Vis Reflectance Graph of STK.

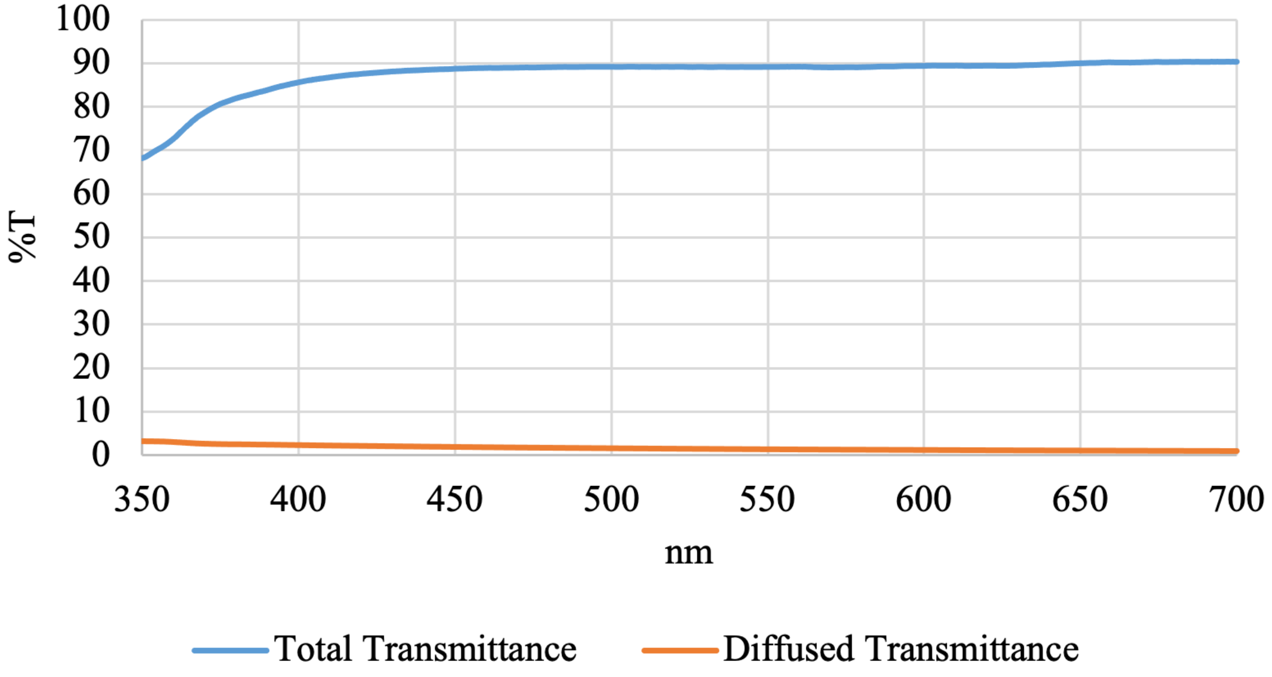

Fig. S-6.1. UV-Vis Transmittance Graph of BEC.

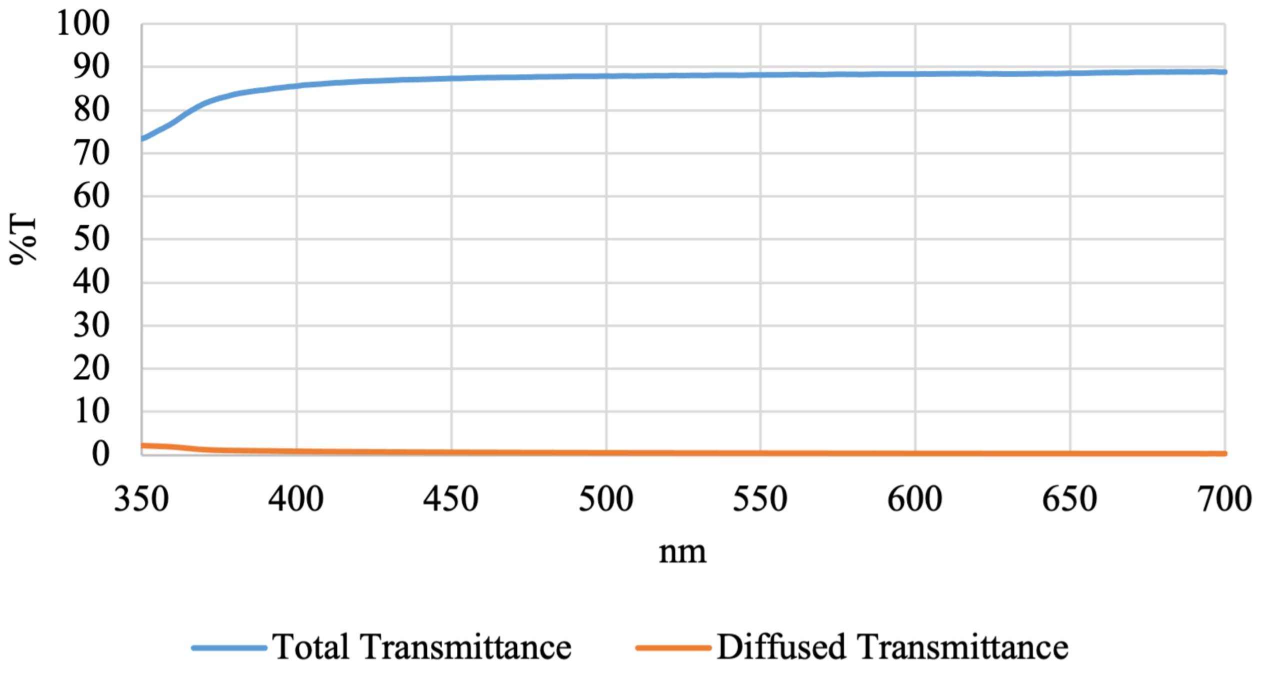

Fig. S-6.2. UV-Vis Transmittance Graph of BES.

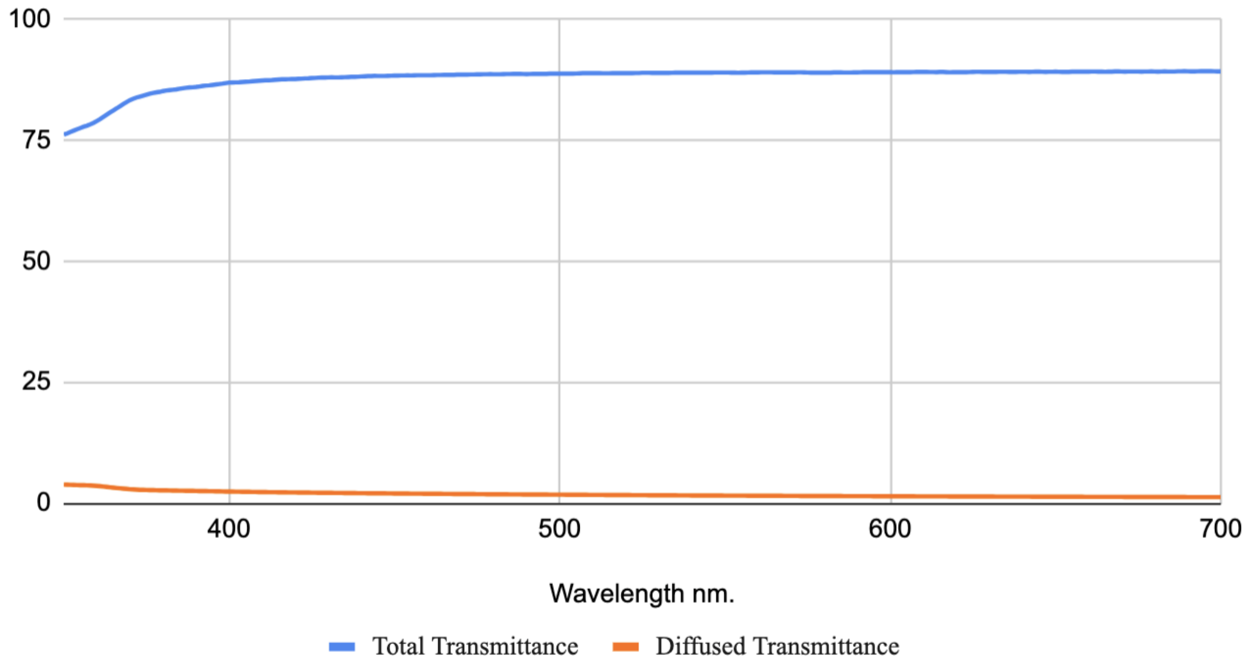

Fig. S-6.3. UV-Vis Transmittance Graph of CLM.

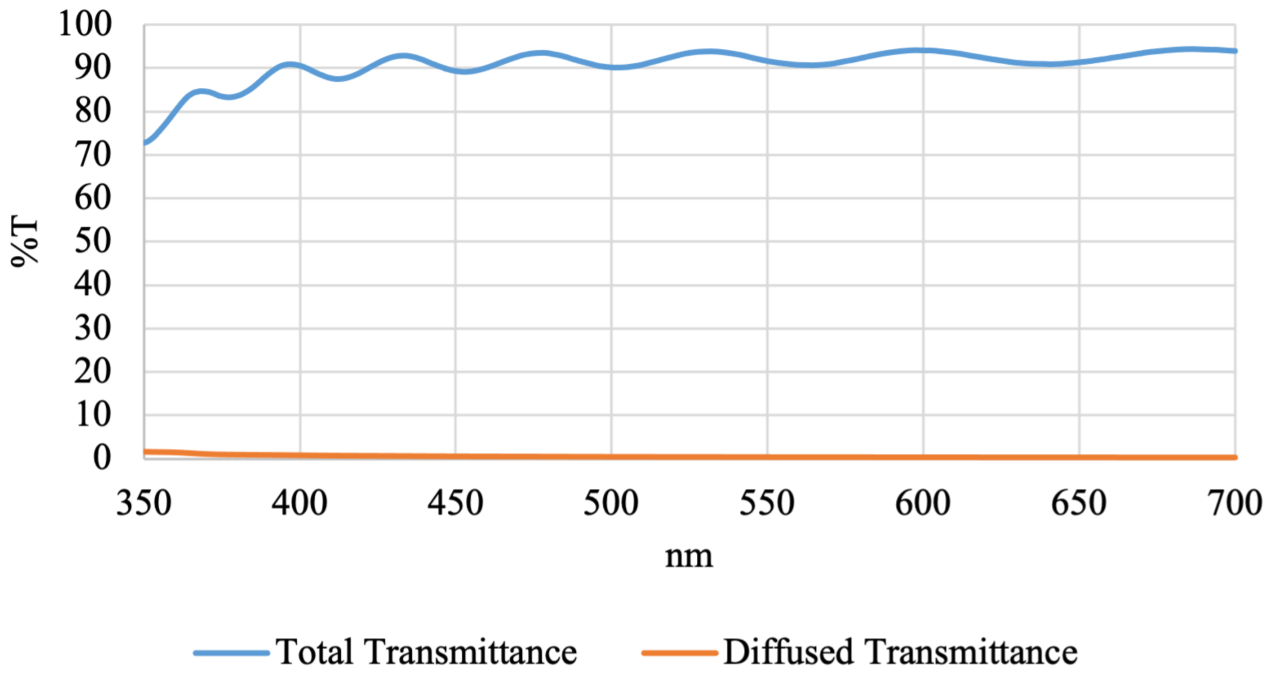

Fig. S-6.4. UV-Vis Transmittance Graph of FAV.

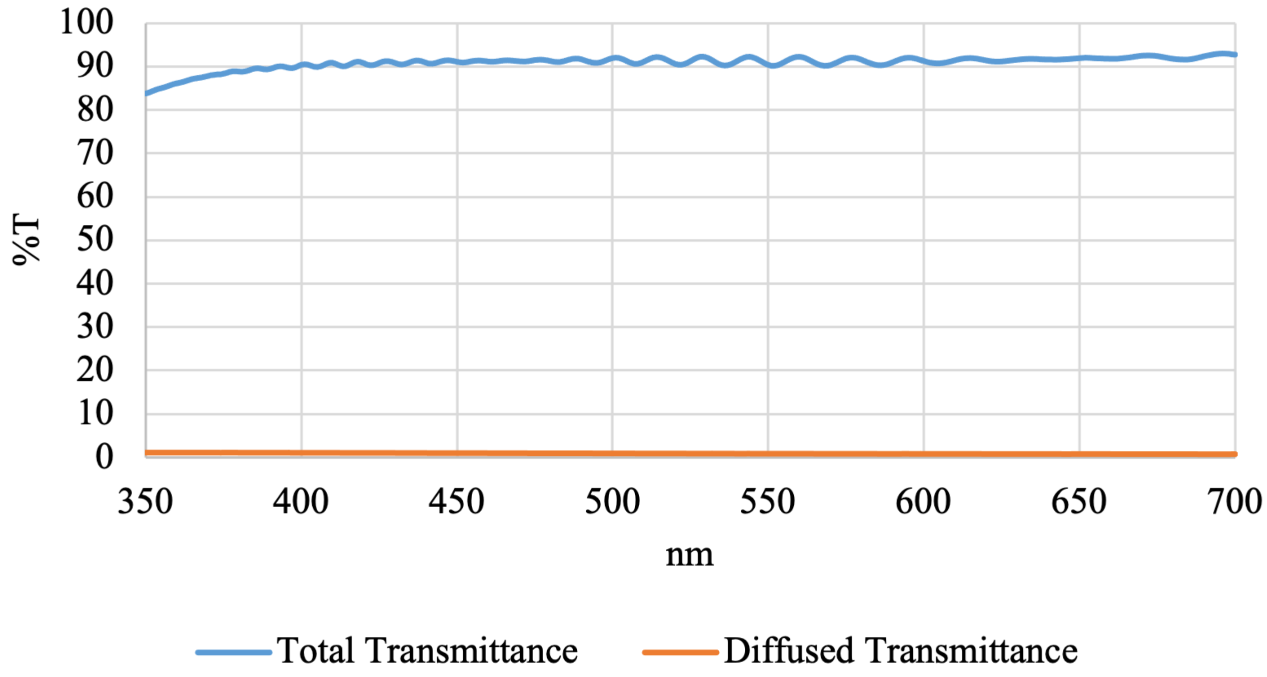

Fig. S-6.5. UV-Vis Transmittance Graph of JEM.

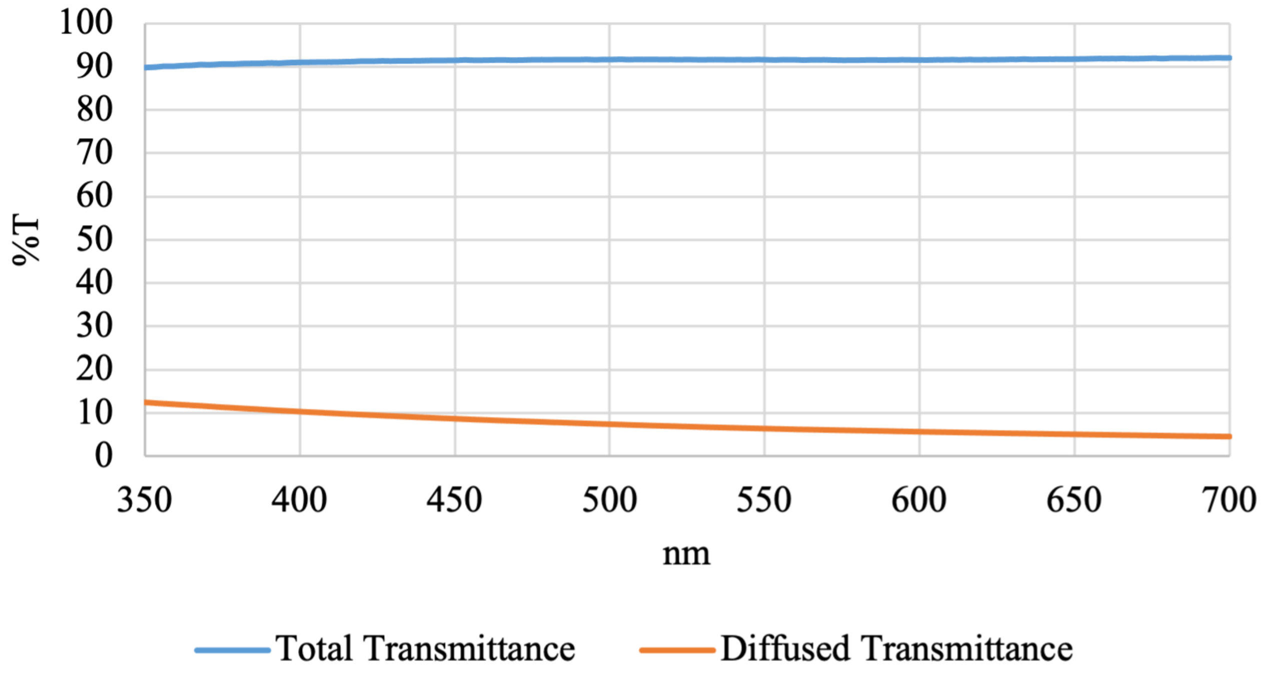

Fig. S-6.6. UV-Vis Transmittance Graph of OPT.

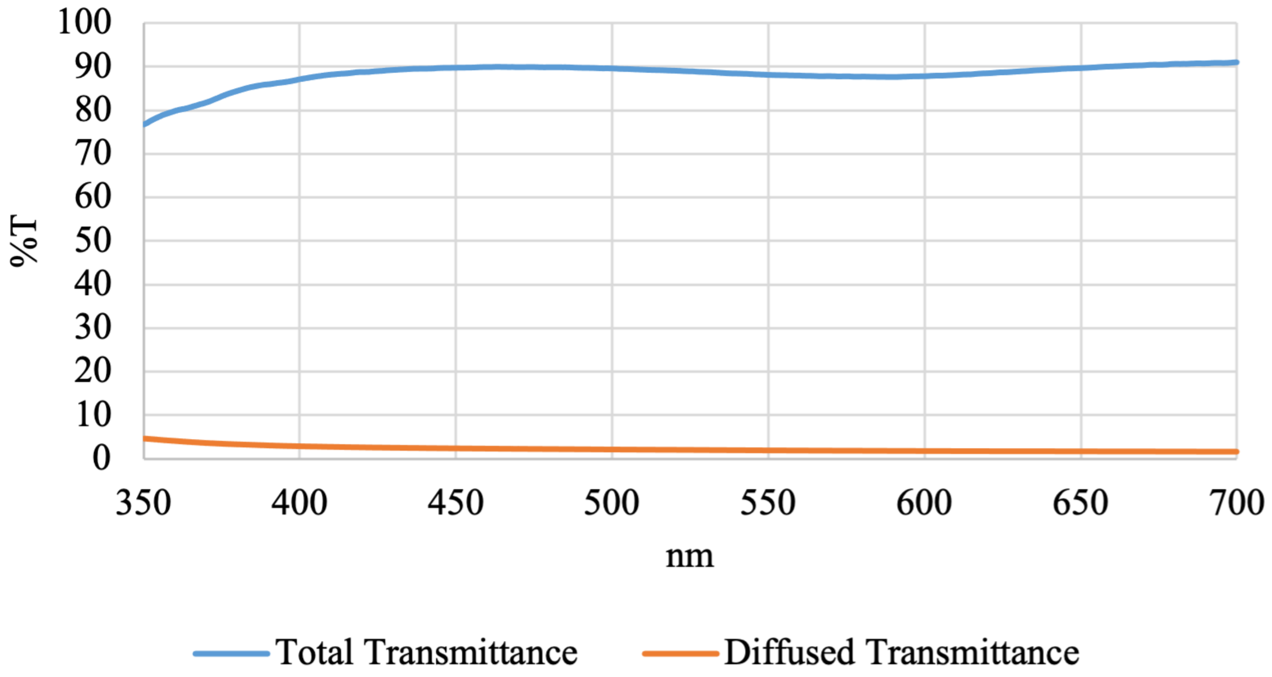

Fig. S-6.7. UV-Vis Transmittance Graph of RAN.

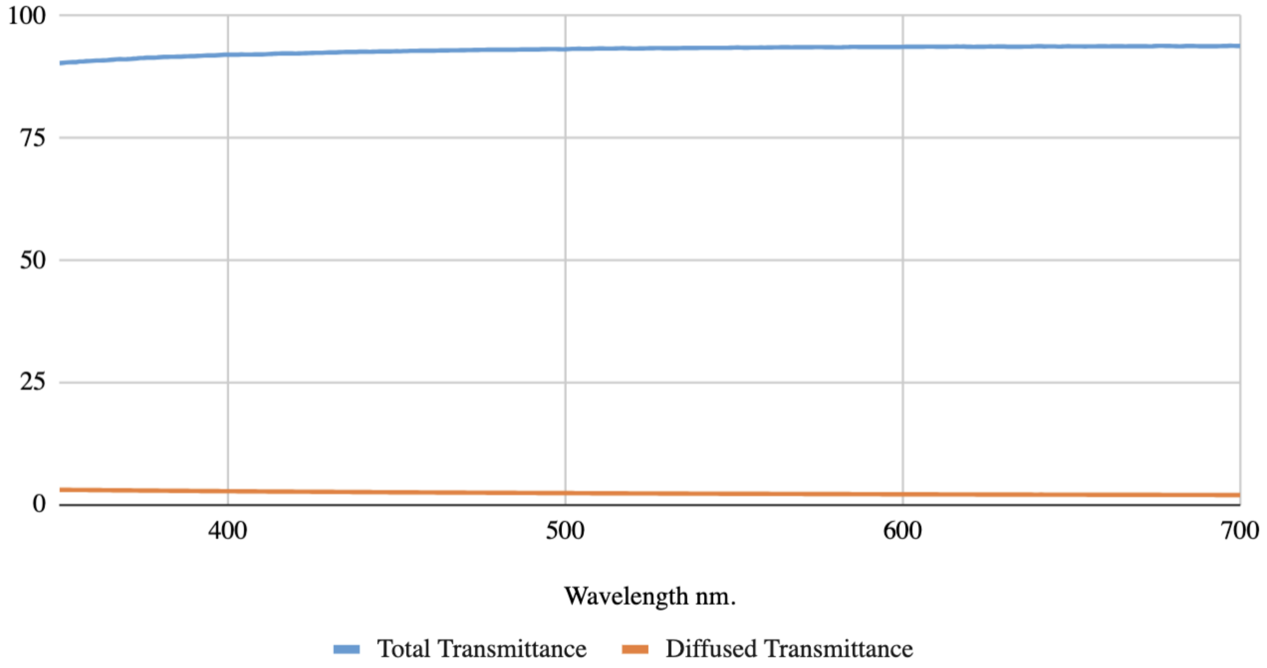

Fig. S-6.4. UV-Vis Transmittance Graph of SEU.

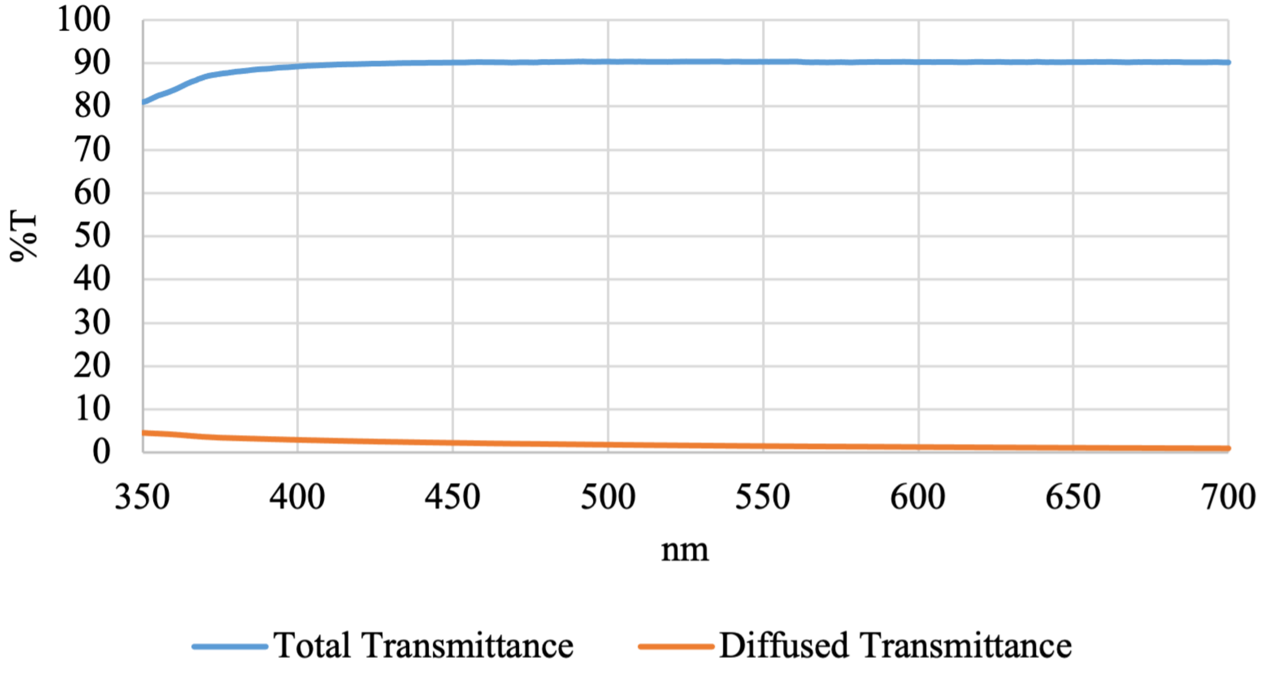

Fig. S-6.4. UV-Vis Transmittance Graph of SNC.

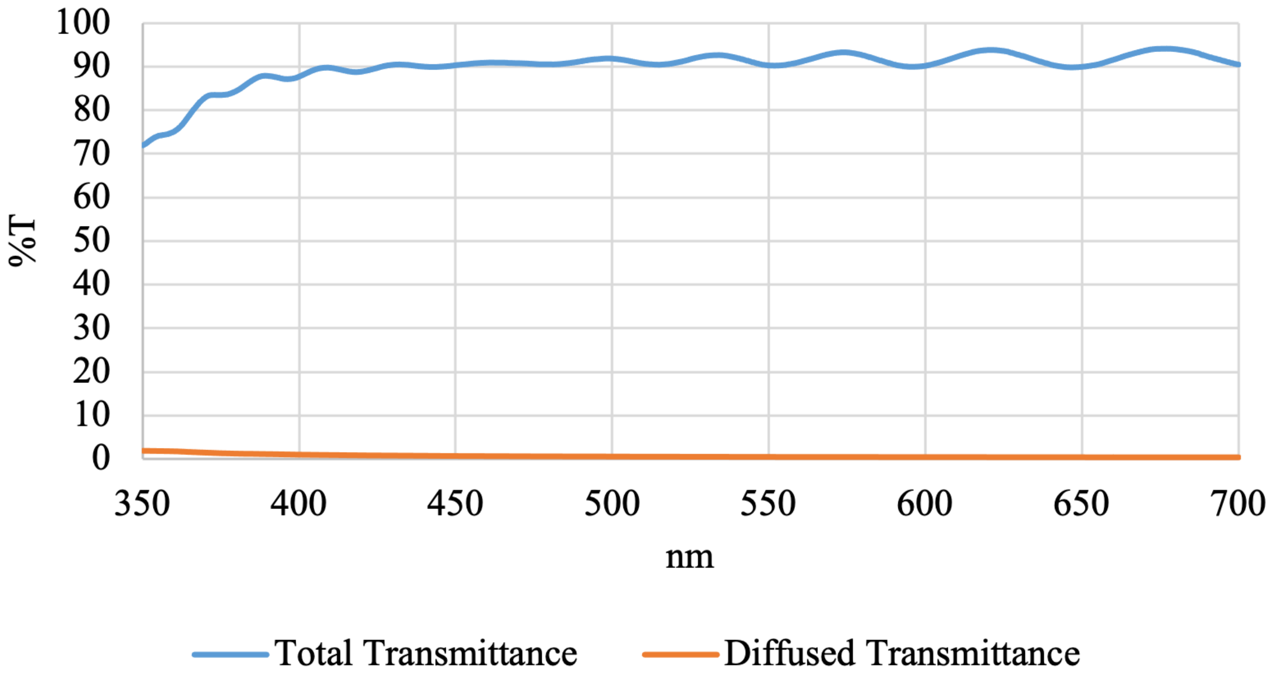

Fig. S-6.4. UV-Vis Transmittance Graph of STK.

Table S-2.1.1. Assessment of PROTECTION – Seal and Good Fit

Mask ID	**PROTECTION Seal (Presence and Quality)**	Rank	Weight	Pts per property
BEC	No	0	0.9	0
BES	No	0	0.9	0
CLM	No	0	0.9	0
FAV	No	0	0.9	0
JEM	Yes, less tight	6	0.9	5.4
OPT	No	0	0.9	0
RAN	No	0	0.9	0
SEU	Yes, tighter	9	0.9	8.1
SNC	No	0	0.9	0
STK	No	0	0.9	0

Comments: comparisons of mainly Boolean data; JEM has a rubber seal on the upper portion of mask, however not very tight; SEM provides sealing tapes on the sides and near the nose to create a tighter fit, enhanced by the soft silicon rubber of the main mask.

Relevance: 1.0 (a good seal guarantees protection from viruses)

Reliability: 0.9 (some uncertainty since tightness is estimated)

Table S-2.1.2. Assessment of PROTECTION – Major Gaps

Mask ID	**PROTECTION Major Gaps**	Rank	Weight	Pts per property
BEC	Top	2	0.9	1.8
BES	No	9	0.9	8.1
CLM	Sides	2	0.9	1.8
FAV	No	9	0.9	8.1
JEM	No	9	0.9	8.1
OPT	No	9	0.9	8.1
RAN	No	9	0.9	8.1
SEU	No	9	0.9	8.1
SNC	No	9	0.9	8.1
STK	No	9	0.9	8.1

Comments: comparison of mainly Boolean data; none of the masks had a very tight fit all over the face (therefore none received a 10); however, BEC, has a molded, unframed and therefore rigid piece of PET, which creates large gaps between mask and face; CLM has large gaps on the sides, because the PET just acts like a face shield.

Relevance: 1.0 (with increasing size of the gap the exposure to viruses increases)

Reliability: 0.9 (gaps were estimated)

Table S-2.1.3. Assessment of PROTECTION – Available Sizes

Mask ID	**PROTECTION Availability of Sizes**	Rank	Weight	Pts per property
BEC	1	0	0.8	0
BES	1	0	0.8	0
CLM	2	5	0.8	4.0
FAV	1	0	0.8	0
JEM	1	0	0.8	0
OPT	1	0	0.8	0
RAN	4	10	0.8	8
SEU	2	5	0.8	4.0
SNC	1	0	0.8	0
STK	1	0	0.8	0

Comments: adjusted, quantitative comparison (those masks that offer two sizes were given half of the available points); this property is not included again under Comfort to avoid overestimating its relevance.

Relevance: 1.0 (the protection depends much on the fit of the mask to the face, certainly an adult mask would not sufficiently protect a child, and vice versa)

Reliability: 0.8 (the few sizes will not guarantee a perfectly tight fit, some related descriptions on websites were not clear on availability of different sizes)

Table S-2.1.4. Assessment of PROTECTION – Breathable area

Mask ID	**PROTECTION Breathable Area (cm^2)**	Rank	Weight	Pts per property
BEC	293	10	0.9	9.0
BES	180	5.9	0.9	5.4
CLM	14	0	0.9	0
FAV	275	9.4	0.9	8.4
JEM	30	0.6	0.9	0.5
OPT	226	7.6	0.9	6.8
RAN	216	7.2	0.9	6.5
SEU	19	0.2	0.9	0.2
SNC	262	8.9	0.9	8.0
STK	243	8.2	0.9	7.4

Comments: quantitative comparison

Relevance: 1.0 (as the breathable area increases the filtration of viruses increases)

Reliability: 0.9 (some masks have efficient filters, position of breathable area is also important)

Table S-2.1.5. Assessment of PROTECTION – Breathable Material

Mask ID	**PROTECTION Material in breathable portion**	Rank	Weight	Pts per property
BEC	it-PP	10	0.8	8
BES	it-PP	10	0.8	8
CLM	PU Foam	7	0.8	5.6
FAV	it-PP	10	0.8	8
JEM	it-PP	10	0.8	8
OPT	it-PP	10	0.8	8
RAN	Cotton/PET	9	0.8	7.2
SEU	PLA	8	0.8	6.4
SNC	PET	8	0.8	6.4
STK	it-PP	10	0.8	8

Comments: iPP is known to be most efficient in filtering viruses, followed by cotton. Fiber made of PET and PLA also are efficient. PU appears to be the least efficient. Polymers were identified by FTIR spectroscopy.

Relevance: 1.0 (certain materials are more efficient in filtering out viruses, e.g. Ref. 5-9)

Reliability: 0.8 (some overlap between properties of materials)

Table S-2.1.6. Assessment of PROTECTION – Filter presence

Mask ID	**PROTECTION Specific Filter**	Rank	Weight	Pts per property
BEC	No	0	1.0	0
BES	No	0	1.0	0
CLM	No	0	1.0	0
FAV	No	0	1.0	0
JEM	Yes	10	1.0	10
OPT	No	0	1.0	0
RAN	No	0	1.0	0
SEU	Yes	10	1.0	10
SNC	No	0	1.0	0
STK	No	0	1.0	0

Comments: comparison of Boolean properties

Relevance: 1.0 (a dedicated filter is most efficient in filtering out viruses)

Reliability: 1.0 (filters could be clearly recognized)

Table S-2.1.7. Assessment of PROTECTION – Nose bridge material

Mask ID	PROTECTION **Tight seal around nose**	Rank	Weight	Pts per property
BEC	(iPP coated wire)	5	0.8	4.0
BES	Al strip	8	0.8	6.4
CLM	(PU foam)	5	0.8	4.0
FAV	PU foam	5	0.8	4.0
JEM	(SR)	7	0.8	5.6
OPT	PU foam	5	0.8	4.0
RAN	PVC coated wire	9	0.8	7.2
SEU	(Sil. adhes. strip)	10	0.8	8.0
SNC	Al strip	8	0.8	6.4
STK	(iPP coated wire)	9	0.8	7.2

Comments: For SEM, the silicon adhesive strip (Sil. adhes. strip) in combination with the silicon rubber appears most tight; coated wire can be well adjusted to the contours around the nose, followed by aluminum strip; PU foam creates some gaps; polymers were identified by FTIR spectroscopy, metals and wires visually; for BEC, solid part was placed on chin and existing nose bridge was not applied as such.

Relevance: 1.0 (tight seal around nose is critical in protection from viruses)

Reliability: 0.8 (some material properties more difficult to differentiate)

Table S-2.1.8. Assessment of PROTECTION – Types of Approvals and Subtotal

Mask ID	**PROTECTION Types of Approvals**	Rank	Weight	Pts per property	**Subtotal**
BEC	KF94	7	0.8	5.6	**28.4**
BES		4	0.8	3.2	**31.1**
CLM	FDA, CE	4	0.8	3.2	**18.6**
FAV	KN95	9	0.8	7.2	**35.7**
JEM		4	0.8	3.2	**40.8**
OPT	N95, NIOSH	10	0.8	8	**34.9**
RAN		4	0.8	3.2	**40.2**
SEU	Filter: N95	9	0.8	7.2	**52.0**
SNC	FDA	4	0.8	3.2	**32.1**
STK		4	0.8	3.2	**33.9**

Comments: As mentioned in the main text, only OPT and SEU (breathable filter) are N95 approved and therefore adheres to the highest standard, because the entire mask is not N95 approved; also SEU does not come with ear loops, which is required for an N95 mask (though it has two slits that could be used for extra ear loops); therefore SEU was ranked slightly lower than OPT; FAV is KN95 approved with the next higher standard, and BEC is KF94 approved with the next higher standard. The FDA and CE (European Conformity) are not conclusive; for any mask that did not have a specific standard the rounded, 50 % for the KF94 rank, or 4, was applied.

Relevance: 1.0 (the standards ensure better protection)

Reliability: 0.8 (some approvals were not clearly stated)

Table S-2.2.1. Assessment of VISIBILITY – Clear area around mouth (based on photos)

Mask ID	**VISIBILITY** **Clear area around mouth (based on photo)**	Rank	Weight	Pts per property
BEC	medium	2	0.8	1.6
BES	large	9	0.8	7.2
CLM	largest	10	0.8	8
FAV	smaller	5	0.8	4
JEM	medium	6	0.8	4.8
OPT	smaller	4	0.8	3.2
RAN	medium	8	0.8	6.4
SEU	larger	7	0.8	5.6
SNC	smallest	0	0.8	0
STK	small	3	0.8	2.4

Comments: comparison of ordinal data, sizes estimated from photos.

Relevance: 1.0 (large area around mouth is critical for lip reading)

Reliability: 0.8 (photos may cause some error due to size, angle, brightness)

Table S-2.2.2. Assessment of VISIBILITY – Transparent area (cm^2)

Mask ID	**VISIBILITY Transparent area (cm^2)**	Rank	Weight	Pts per property
BEC	151.5	7.9	1.0	7.9
BES	121.8	6.0	1.0	6.0
CLM	131.3	6.6	1.0	6.6
FAV	42.4	1.0	1.0	1.0
JEM	111.1	5.3	1.0	5.3
OPT	49.3	1.4	1.0	1.4
RAN	52.8	1.6	1.0	1.6
SEU	184.6	10	1.0	10
SNC	26.9	0	1.0	0
STK	47.6	1.3	1.0	1.3

Comments: quantitative comparison

Relevance: 1.0 (as the clear area increases, more visual clues can be communicated)

Reliability: 1.0 (very little error)

Table S-2.2.3. Assessment of VISIBILITY – Apparent distortion (based on photos)

Mask ID	**VISIBILITY Apparent Distortion (photo)**	Rank	Weight	Pts per property
BEC	highest	0	0.8	0
BES	low	9	0.8	7.2
CLM	high	3	0.8	2.4
FAV	medium	4	0.8	3.2
JEM	high	2	0.8	1.6
OPT	low	10	0.8	8.0
RAN	low	10	0.8	8.0
SEU	higher	0	0.8	0
SNC	low	9	0.8	7.2
STK	low	9	0.8	7.2

Comments: comparison of ordinal data, estimated from photos

Relevance: 1.0 (with increasing distortion the mask wearer looks less natural)

Reliability: 0.8 (photos may cause some error due to size, angle, brightness)

Table S-2.2.4. Assessment of VISIBILITY – Apparent reflections (photos)

Mask ID	**VISIBILITY Apparent Reflectance Photo**	Rank	Weight	Pts per property
BEC	high	0	0.8	0
BES	low	8	0.8	6.4
CLM	high	3	0.8	2.4
FAV	low	9	0.8	7.2
JEM	higher	2	0.8	1.6
OPT	low	10	0.8	8
RAN	low	10	0.8	8
SEU	highest	0	0.8	0
SNC	low	9	0.8	7.2
STK	low	9	0.8	7.2

Comments: comparison of ordinal terms, estimated from photos

Relevance: 1.0 (with increasing reflectance the critical area of the face of the mask wearer can be less clearly seen)

Reliability: 0.8 (photos may cause some error due to size, angle, brightness)

Table S-2.2.5. Assessment of VISIBILITY – Reflectance by UV-Vis

Mask ID	**VISIBILITY Reflectance UV-Vis (%)**	Rank	Weight	Pts per property
BEC	3.0	7.7	0.9	6.9
BES	4.3	3.5	0.9	3.2
CLM	4.0	4.5	0.9	4.1
FAV	2.7	8.7	0.9	7.8
JEM	3.1	7.4	0.9	6.7
OPT	5.4	0	0.9	0
RAN	2.3	10	0.9	9
SEU	4.6	2.6	0.9	2.3
SNC	5.4	0	0.9	0
STK	3.3	6.8	0.9	6.1

Comments: quantitative comparison, based on reflectance within the material

Relevance: 0.9 (slightly less relevant compared to apparent reflectance shown in Table S-2.2.6.)

Reliability: 1.0 (quantitative, with little measurement error, commonly estimated at 0.1 to 1 %)

Table S-2.2.6. Assessment of VISIBILITY – Haze by UV-Vis

Mask ID	**VISIBILITY Haze UV-Vis (%)**	Rank	Weight	Pts per property
BEC	1.817	8.3	1.0	8.3
BES	0.707	9.7	1.0	9.7
CLM	2.063	7.9	1.0	7.9
FAV	0.508	10	1.0	10
JEM	0.973	9.4	1.0	9.4
OPT	8.04	0	1.0	0
RAN	2.463	7.4	1.0	7.4
SEU	2.435	7.4	1.0	7.4
SNC	2.148	7.8	1.0	7.8
STK	0.726	9.7	1.0	9.7

Comments: quantitative comparison

Relevance: 1.0 (haze can significantly reduce visibility)

Reliability: 1.0 (little measurement error, typically estimated at below 5 %)

Table S-2.2.7. Assessment of VISIBILITY – Crystallinity from Density and DSC

Mask ID	**VISIBILITY Average Degree of Crystallinity**	Rank	Weight	Pts per property
BEC	0.0	10.0	0.81	8.1
BES	49.9	4.1	0.81	3.3
CLM	48.3	4.3	0.81	3.5
FAV	52.2	3.9	0.81	3.2
JEM	0.0	10.0	0.81	8.1
OPT	85.2	0.0	0.81	0.0
RAN	0.0	10.0	0.81	8.1
SEU	0.0	10.0	0.81	8.1
SNC	50.9	4.0	0.81	3.2
STK	50.0	4.1	0.81	3.3

Comments: quantitative comparison; values are averages of degrees of crystallinity obtained from DSC and density

Relevance: 0.9 (other factors besides crystallinity can also influence clarity, however, crystallinity having a dominating impact)

Reliability: 0.9 (quantitative with reasonably low error based on the two methods estimated at about 5 %)

Table S-2.2.8. Assessment of VISIBILITY – Contact Angle, Inside

Mask ID	**VISIBILITY Contact Angle, Inside (º)**	Rank	Weight	Pts per Property
BEC	74.9	1.6	0.9	1.4
BES	62.5	3.2	0.9	2.9
CLM	28	7.7	0.9	6.9
FAV	11.7	9.8	0.9	8.8
JEM	18.2	8.9	0.9	8.1
OPT	53.2	4.4	0.9	4.0
RAN	76.6	1.4	0.9	1.2
SEU	87.2	0.0	0.9	0.0
SNC	44.7	5.5	0.9	5.0
STK	10.1	10.0	0.9	9.0

Comments: quantitative comparison

Relevance: 1.0 (with decreasing contact angle the probability for fogging decreases)

Reliability: 0.9 (relatively low error, as indicated by low standard deviations in main text)

Table S-2.2.9. Assessment of VISIBILITY – Inside (anti-fog) coating

Mask ID	**VISIBILITY Inside (Anti-fog) coating, Applied & Uniformity**	Rank	Weight	Pts per property
BEC	Y, not uniform	7	0.8	5.6
BES	Y	10	0.8	8
CLM	Y, not uniform	7	0.8	5.6
FAV	Y	10	0.8	8
JEM	Y	10	0.8	8
OPT	Y	10	0.8	8
RAN	N	0	0.8	0
SEU	Y	10	0.8	8
SNC	Y	10	0.8	8
STK	Y	10	0.8	8

Comments: comparison of mostly Boolean properties, based on information from manufacturer and confirmed by EDX and contact angle measurements.

Relevance: 1.0 (with increasing fog the visibility of the transparent portion decreases and communication is impaired)

Reliability: 0.9 (differences in some antifog formulas)

Table S-2.2.10. Assessment of VISIBILITY – Outside (anti-scratch) coating and Subtotal

	**VISIBILITY** **Outside (Anti-scratch) coating**	Rank	Weight	Pts per property	**Subtotal**
BEC	N	0	0.72	0	**39.9**
BES	N	0	0.72	0	**53.9**
CLM	N	0	0.72	0	**47.4**
FAV	Y	10	0.72	7.2	**60.4**
JEM	Y	10	0.72	7.2	**60.7**
OPT	N	0	0.72	0	**32.6**
RAN	N	0	0.72	0	**49.7**
SEU	N	0	0.72	0	**41.4**
SNC	N	0	0.72	0	**38.4**
STK	Y	10	0.72	7.2	**61.4**

Comment: comparison of Boolean property; based on information from manufacturers and confirmed by EDX and contact angle measurements.

Relevance: 0.8 (less relevant for communicating than anti-fog coating)

Reliability: 0.9 (dependence on EDX and contact angle measurements)

Table S-2.3.1. Assessment of COMFORT – Flexibility from T_g

	**COMFORT Flexibility from Tg (ºC)**	Rank	Weight	Pts per property
BEC	67.9	4.6	0.81	3.7
BES	73.3	4.2	0.81	3.4
CLM	74.1	4.2	0.81	3.4
FAV	75	4.1	0.81	3.3
JEM	138	0	0.81	0.0
OPT	-14.9	10	0.81	8.1
RAN	77	4.0	0.81	3.2
SEU	0	9.0	0.81	7.3
SNC	72.9	4.3	0.81	3.5
STK	66.2	4.7	0.81	3.8

Comments: quantitative comparison

Relevance: 0.9 (as T_g increases the flexibility of the material decreases, important for jaw movements; other factors like crystallinity and molecular weight may also cause effects)

Reliability: 0.9 (since quantitative and measurement errors are low).

Table S-2.3.2. Assessment of COMFORT – Flexibility from Tensile Strength

	**COMFORT Flexibility based on Tensile Strength (MPa)**	Rank	Weight	Pts per property
BEC	51.7	7.5	0.8	5.8
BES	189	0	0.8	0.0
CLM	179	1	0.8	0.4
FAV	166	1.5	0.8	1.0
JEM	68.2	6.5	0.8	5.1
OPT	28.5	9	0.8	6.8
RAN	57.5	7	0.8	5.6
SEU	0.248	10	0.8	8.0
SNC	122	4	0.8	2.8
STK	148	2.5	0.8	1.8

Comments: quantitative comparison

Relevance: 1.0 (generally, as the strength of a polymer increases, the flexibility decreases, important for jaw movements)

Reliability: 0.8 (lower reliability because for JEM only four instead of regularly five dumbbells could be measured, and in one case (SNC) the specimens had to be cut in horizontal instead of vertical direction from the mask.)

Table S-2.3.3. Assessment of COMFORT – Flexibility from Elongation

Mask ID	**COMFORT Flexibility based on Elongation (%)**	Rank	Weight	Pts per property
BEC	371.3	5.4	0.8	4.3
BES	172.7	1.9	0.8	1.5
CLM	100.6	0.7	0.8	0.6
FAV	200	2.4	0.8	1.9
JEM	60.6	0	0.8	0.0
OPT	636.9	10	0.8	8.0
RAN	139.3	1.4	0.8	1.1
SEU	186.6	2.2	0.8	1.8
SNC	99	0.7	0.8	0.6
STK	170	1.9	0.8	1.5

Comments: quantitative comparison

Relevance: 1.0 (with increasing ability of the material to deform, jar movements become easier)

Reliability: 0.8 (lower reliability because for JEM only four instead of regularly five dumbbells could be measured, and in one case (SNC) the specimens had to be cut in horizontal instead of vertical direction from the mask.)

Table S-2.3.4. Assessment of COMFORT – Flexibility from Modulus

Mask ID	**COMFORT Flexibility based on Modulus (MPa)**	Rank	Weight	Pts per property
BEC	390	9.0	0.8	7.2
BES	4,060	0.0	0.8	0.0
CLM	3,570	1.2	0.8	1.0
FAV	3,450	1.5	0.8	1.2
JEM	2,230	4.5	0.8	3.6
OPT	1,000	7.5	0.8	6.0
RAN	2,040	5.0	0.8	4.0
SEU	0.391	10.0	0.8	8.0
SNC	3,190	2.1	0.8	1.7
STK	3,280	1.9	0.8	1.5

Comment: quantitative comparison

Relevance: 1.0 (as the modulus increases the mask feels stiffer)

Reliability: 0.8 (lower reliability because for JEM only four instead of regularly five dumbbells could be measured, and in one case (SNC) the specimens had to be cut in horizontal instead of vertical direction from the mask.)

Table S-2.3.5. Assessment of COMFORT – Mass

Mask ID	**COMFORT Mass (g)**	Rank	Weight	Pts per property
BEC	15.3	8.7	1.0	8.7
BES	8.75	9.6	1.0	9.6
CLM	9.07	9.6	1.0	9.6
FAV	8.72	9.6	1.0	9.6
JEM	78.31	0.0	1.0	0
OPT	7.36	9.8	1.0	9.8
RAN	21.45	7.9	1.0	7.9
SEU	45.68	4.5	1.0	4.5
SNC	5.94	10.0	1.0	10
STK	6.9	9.9	1.0	9.9

Comments: quantitative comparison

Relevance: 1.0 (with increasing mass, the mask feels less comfortable)

Reliability: 1.0 (high accuracy)

Table S-2.3.6. Assessment of COMFORT – Nose bridge material

Mask ID	**COMFORT Nose bridge Foam, less pressure on nose**	Rank	Weight	Pts per property
BEC	Coated wire	7	0.72	5.0
BES	Al strip	5	0.72	3.6
CLM	PU foam	10	0.72	7.2
FAV	PU foam	10	0.72	7.2
JEM	(SR)	9	0.72	6.5
OPT	PU foam	10	0.72	7.2
RAN	Coated wire	7	0.72	5.0
SEU	(Sil. adhes. strip)	9	0.72	6.5
SNC	Al strip	5	0.72	3.6
STK	Coated wire	7	0.72	5.0

Comments: The PU foam provides most comfort, then the silicon adhesive strip (Sil. adhes. strip), followed by the coated wire, and lastly the aluminum strip; PU foam was identified by FTIR spectroscopy, metals and wires visually; polymers were identified by FTIR spectroscopy, metals and wires visually; for BEC, solid part was placed on chin and existing nose bridge was not applied as such.

Relevance: 0.9 (as the stiffness of the nose bridge increases, it will feel less comfortable)

Reliability: 0.8 (effect of materials not highly distinguishable)

Table S-2.3.7. Assessment of COMFORT – Ear loops

Mask ID	**COMFORT Ear loops Softness based on polymer**	**Average Elongation**	Rank	Weight	Pts per property
BEC	Nylon 6	90	1.2	0.72	0.9
BES	PET	70	0	0.72	0
CLM	PET	70	0	0.72	0
FAV	Nylon 6	90	1.2	0.72	0.9
JEM	Silicon Rubber	500	10	0.72	7.2
OPT	NBR	200	4.0	0.72	2.9
RAN	PET	70	0	0.72	0
SEU	not included	100	2.0	0.72	1.4
SNC	PET	70	0	0.72	0
STK	Nylon 6	90	1.3	0.72	0.9

Comments: Generally, the more elastic, the more comfortable; materials were identified by FTIR spectroscopy; the elongation at break decreases generally in the following order: SR, NBR (nitrile rubber), Nylon and PET, with typical averages of 500, 200, 90 and 70 %, respectively, for the unreinforced polymers based on internet sources (www.matweb.com; www.curbellplastics.com). A manual test confirmed the different degrees of stretchability; with SEU there are only slits for ear loops, but no ear loops present, which does not fulfill one of the N95 requirements

Relevance: 0.9 (with increasing stiffness or roughness of the ear loop, comfort decreases; however, this is not as relevant as for the role of the ear loop for protection)

Reliability: 0.8 (elongations may vary with grade and purity of polymer)

Table S-2.3.8. Assessment of COMFORT – Ease of Assembly and Subtotal

Mask ID	**COMFORT Ease of Assembly**	Rank	Weight	Pts per property	**Subtotal**
BEC	most difficult	0	0.72	0	**35.7**
BES	not required	10	0.72	7.2	**25.3**
CLM	not required	10	0.72	7.2	**29.3**
FAV	not required	10	0.72	7.2	**32.3**
JEM	more difficult	2	0.72	1.4	**23.8**
OPT	not required	10	0.72	7.2	**56.0**
RAN	not required	10	0.72	7.2	**34.1**
SEU	difficult	4	0.72	2.9	**40.3**
SNC	not required	10	0.72	7.2	**29.3**
STK	not required	10	0.72	7.2	**31.7**

Comments: comparison of ordinal terms; BEC was exceptional having the clear face on top, and the breathable part on the bottom, it is the only mask that required an adhesive strip for the chin, which makes it difficult to get a tight fit on the face and it took the longest to assemble; JEM has two components for the ear loops, a simple rubber bands and a strip for the neck, which are not easy to get hooked, to change filters, filter caps must be removed; SEU required use of the strips (two for the sides and one above the nose) to create a tighter seal.

Relevance: 0.8 (preferably no need to assemble, as the assembly gets more complex, the user will feel less comfortable)

Reliability: 0.9 (may include a little degree of subjectivity)

Table S-2.4.1 – Assessment of SUSTAINABILITY – Reusability

Mask Type	**SUSTAINABILITY Reusability Manufacturer info**	Rank	Weight	Pts per property
BEC	Reusable	9	0.8	7.2
BES	10 times/80 h	6	0.8	4.8
CLM	Reusable	9	0.8	7.2
FAV	Reusable	9	0.8	7.2
JEM	Reusable	9	0.8	7.2
OPT	Reusable	9	0.8	7.2
RAN	Reusable	9	0.8	7.2
SEU	Washable	10	0.8	8
SNC	Disposable	0	0.8	0
STK	For a week	7	0.8	5.6

Comments: mostly ordinal properties, reusable is clearly better than disposable

Relevance: 1.0 (the more often the mask can be reused, the better; high impact on amount of plastic waste in the environment)

Reliability: 0.8 (to some degree not clearly distinguishable)

Table S-2.4.2 – Assessment of SUSTAINABILITY –Recyclability

Mask Type	**SUSTAINABILITY Recylability Type of Polymer**	Rank	Weight	Pts per property
BEC	PETG/iPP/PU	5	0.9	4.5
BES	PET /iPP	7	0.9	6.3
CLM	PET/PU	5	0.9	4.5
FAV	PET/iPP	7	0.9	6.3
JEM	PC/SiO_2/Nylon	1	0.9	0.9
OPT	iPP/iPP	8	0.9	7.2
RAN	PVC/iPP	6	0.9	5.4
SEU	SR/PLA	4	0.9	3.6
SNC	PET/PET	10	0.9	9
STK	PET/iPP	7	0.9	6.3

Comments: transparent / breathable materials shown; specifically recyclable polymers received higher ranking, PET is the most recycled within its group (between 15 and 30 %); average recycling rate of iPP is only about 5 % (see references 60 and 61); there is no specific recycle group for PETG, PC, Nylon, SR, PLA and PU; both OPT and SNC are mono-materials (i.e. contain the same material in the transparent and breathable portions); the easier the separation of different materials, the better; FAV and STK have major layers of coatings; for JEM, the rubber seal was glued to the transparent portion, which makes separation difficult.

Relevance: 1.0 (the higher the recycling rate of the polymer, the better; recyclability would greatly reduce negative impact on environment)

Reliability: 0.9 (some dependance on grades of a polymer)

Table S-2.4.3 – Assessment of SUSTAINABILITY – Renewability

Mask Type	**SUSTAINABILITY Renewability**	**kg plastic /L oil**	Rank	Weight	Pts per property
BEC	PETG	0.75	1.6	0.8	0
BES	PET	0.875	3.0	0.8	2.4
CLM	PET	0.875	3.0	0.8	2.4
FAV	PET	0.875	3.0	0.8	2.4
JEM	PC/SiO_2	0.735	1.4	0.8	1.1
OPT	it-PP	0.61	0.0	0.8	0
RAN	PVC	1.2	6.6	0.8	5.3
SEU	SR/PLA	1.5	10.0	0.8	8.0
SNC	PET	0.875	3.0	0.8	2.4
STK	PET	0.875	3.0	0.8	2.4

Comments: quantitative comparison; kg plastic/p L oil was estimated for PETG and SR/PLA (see references 62-65)

Relevance: 1.0 (the higher the use of fossil fuel based materials, the larger the contribution to climate change)

Reliability: 0.8 (data may not be current, but represent general differences between polymers)

Table S-2.4.4. – Assessment of SUSTAINABILITY – Biodegradability

Mask Type	**SUSTAINABILITY Biodegradability Type of Polymer**	Rank	Weight	Pts per property
BEC	PETG	3	0.8	2.4
BES	PET	2	0.8	1.6
CLM	PET	2	0.8	1.6
FAV	PET	2	0.8	1.6
JEM	PC/SiO_2	2	0.8	1.6
OPT	it-PP	1	0.8	0.8
RAN	PVC	1	0.8	0.8
SEU	SR/PLA	4	0.8	3.2
SNC	PET	2	0.8	1.6
STK	PET	2	0.8	1.6

Comments: comparison based on ordinal data, see references 66 and 67.

Relevance: 1.0 (the higher the degree of biodegradability, the lower the accumulation of waste and the better for the terrestrial and marine environment)

Reliability: 0.8 (some spread between biodegradability data, due to type (compost vs. marine environment)

Table S-2.4.5 – Assessment of SUSTAINABILITY – Mass

Mask Type	**SUSTAINABILITY Mass (g)**	Rank	Weight	Pts per property
BEC	15.3	8.7	1.0	8.7
BES	8.75	9.6	1.0	9.6
CLM	9.07	9.6	1.0	9.6
FAV	8.72	9.6	1.0	9.6
JEM	78.31	0.0	1.0	0
OPT	7.36	9.8	1.0	9.8
RAN	21.45	7.9	1.0	7.9
SEU	45.68	4.5	1.0	4.5
SNC	5.94	10.0	1.0	10
STK	6.9	9.9	1.0	9.9

Comments: quantitative comparison

Relevance: 1.0 (the lower the mass of a product for a given target application, the less waste is produced)

Reliability: 1.0 (highly accurate)

Table S-2.4.6 – Assessment of SUSTAINABILITY – Thickness

Mask Type	**SUSTAINABILITY Thickness mm**	Rank	Weight	Pts per property
BEC	0.441	7.6	1.0	7.6
BES	0.165	9.7	1.0	9.7
CLM	0.190	9.5	1.0	9.5
FAV	0.195	9.5	1.0	9.5
JEM	1.456	0.0	1.0	0.0
OPT	0.123	10.0	1.0	10.0
RAN	0.288	8.8	1.0	8.8
SEU	0.600	6.4	1.0	6.4
SNC	0.168	9.7	1.0	9.7
STK	0.190	9.5	1.0	9.5

Comments: quantitative comparison

Relevance: 1.0 (the thinner the product while meeting requirements for a target application, the less waste is produced)

Reliability: 1.0 (highly accurate, measured with a micrometer, reading 3 decimals for mm)

Table S-2.4.7 – Assessment of SUSTAINABILITY – Price and Subtotal

	SUSTAINABILITY				
Mask Type	**Price $/mask**	Rank	Weight	Pts per property	Subtotal
BEC	1	10	0.7	7	**37.4**
BES	7.5	10	0.7	7	**41.4**
CLM	7.5	10	0.7	7	**41.8**
FAV	3.5	10	0.7	7	**36.4**
JEM	35	5	0.7	3.5	**14.3**
OPT	1.5	10	0.7	7	**42.0**
RAN	5	10	0.7	7	**42.4**
SEU	66.5	0	0.7	0	**33.7**
SNC	1.25	10	0.7	7	**39.7**
STK	3.5	10	0.7	7	**42.3**

Comments: quantitative comparison

Relevance: 1.0 (the lower the price, the better regarding the economical aspect of sustainability)

Reliability: 0.7 (prices may change depending on manufacturer, seller and time)
